# Supplementary material for: Formal pyridine meta-azidation and its application for the synthesis of diazepines, ring-fused δ-carbolines and 1,2,3-triazolylpyridines
Source: Nat Commun. 2025 Oct 23;16:9395. doi: 10.1038/s41467-025-65313-x (PMC12549803; doi:10.1038/s41467-025-65313-x)
Supplement: Supplementary file 1 — Supplementary Information [file 41467_2025_65313_MOESM1_ESM.pdf]

Supplementary Information

Formal pyridine *meta*-azidation and its application for the synthesis of diazepines, ring-fused δ-carbolines and 1,2,3-triazolylpyridines

Shu-Min Guo,[+] Pengwei Xu,[+] Constantin G. Daniliuc, and Armido Studer*

Organisch-Chemisches Institut, Universität Münster, 48149 Münster, Germany.

Table of Contents

General Methods 3

Experimental Sections 5

General procedure for redox-neutral dearomatization 6

General procedure for *meta*-C–H azidation 21

General procedure for ring enlargement 35

General procedure for C–H insertion 42

Proposed mechanism for hydrolysis 45

Synthetic application 48

X-ray 50

NMR Spectra 53

References 137

General Methods

Experimental procedures, reagents and glassware

All commercially available chemicals were used without additional purification for the synthesis of substrates. All reactions were carried out under an atmosphere of argon in oven-dried glassware with magnetic stirring, unless otherwise indicated. Chemicals were used as obtained from the suppliers unless otherwise stated. Solvent compositions are given in (v/v).

Chromatography

Flash chromatography was performed with silica gel (40-63 μm grade) (Merck or VWR) using pressurized air. Analytical thin-layer chromatography was performed with commercial glass plates coated with 0.25 mm silica gel (E. Merck, Kieselgel 60 F_{254}). Compounds were visualized under UV-light at 254 nm or 366 nm and oxidized in standard $KMnO_4$ solution followed by heating if necessary.

NMR spectroscopy

Nuclear magnetic resonance spectra were recorded on a Bruker Avance II 300 (300 MHz), Bruker NEO 400 (400 MHz) or a DD2 500 (Agilent, 500 MHz) in deuterated chloroform (residual peaks ^{1}H δ 7.26 ppm, ^{13}C δ 77.16 ppm) unless otherwise noted.

Chemical shifts (δ) are reported in parts per million (ppm) relative to residual chloroform (s, 7.26 ppm). Proton decoupled Carbon-13 nuclear magnetic resonance (^{13}C{^{1}H} NMR) were acquired at 101 MHz on a Bruker NEO 400 spectrometer. Proton decoupled Fluorine-19 nuclear magnetic resonance (^{19}F{^{1}H} NMR) were acquired at 282 MHz on a Bruker AV300 spectrometer. Splitting patterns are designated as s, singlet; d, doublet; t, triplet; q, quartet; p, pentet; hept, heptet; dd, doublet of doublets; dt, doublet of triplets; ddd, doublet of doublets of doublets; tt, triplet of triplets; tq, triplet of quartets; qt, quartet of triplets; m, multiplet. The NMR data were recorded at 298 K or 223K.

Mass spectrometry

High resolution mass spectra were recorded by Dr. Matthias Letzel and his coworkers (Department of Chemistry, University of Münster) on a Thermo Fisher Scientific Exploris 120 Electrospray Orbitrap ESI mass spectrometer.

Infrared Spectroscopy

IR spectra were recorded on a FTIR-4600LE FTIR spectrometer (Jasco) and are reported in reciprocal centimeters (cm^{-1}), and the absorption bands are stated with their wave numbers ν with the intensities: *w* (weak), *m* (medium), *s* (strong) or *br* (broad).

Melting points

Melting points were obtained on a Büchi melting point M-565, and are uncorrected.

Photoreactions

Photoreactions were performed with a Kessil PR160L 456 nm lamp (40 W).

X-Ray diffraction

Data sets for compounds **34**, **43** and **48** were collected with a Bruker D8 Venture Photon III Diffractometer. Programs used: data collection: *APEX6* Version 2024.9-0 [1] (Bruker AXS Inc., **2024**); cell refinement: *SAINT* Version 8.41 (Bruker AXS Inc., **2024**); data reduction: *SAINT* Version 8.41 (Bruker AXS Inc., **2024**); absorption correction, *SADABS* Version 2016/2 (Bruker AXS Inc., **2024**); structure solution *SHELXT*-Version 2018-3 [2] (Sheldrick, G. M. *Acta Cryst.*, **2015**, *A71*, 3-8); structure refinement *SHELXL*- Version 2019-2 [3] (Sheldrick, G. M. *Acta Cryst.*, **2015**, *C71* (1), 3-8) and graphics, *XP* [4] (Version 5.1, Bruker AXS Inc., Madison, Wisconsin, USA, **1998**). *R*-values are given for observed reflections, and wR^2 values are given for all reflections.

Experimental Sections

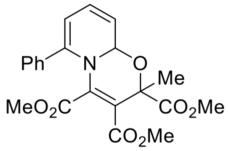

S1

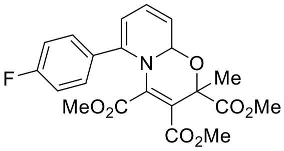

S2

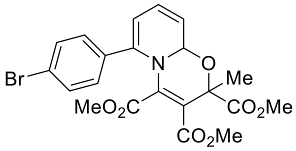

S3

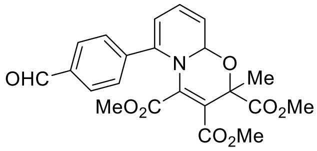

S4

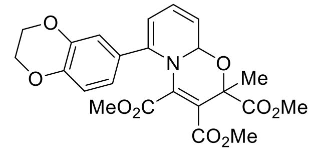

S5

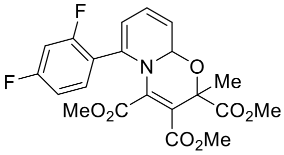

S6

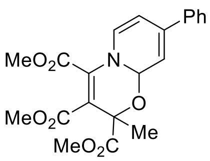

S7

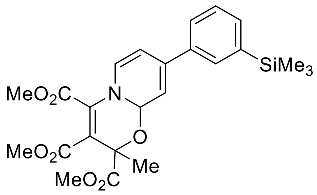

S8

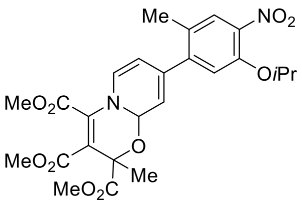

S9

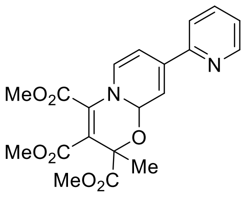

S10

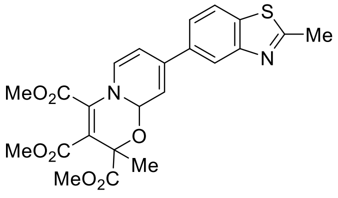

S11

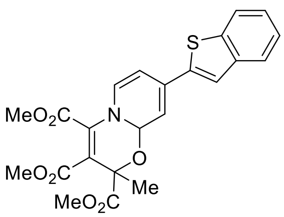

S12

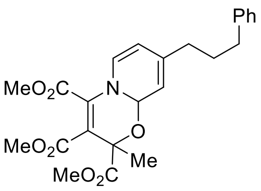

S13

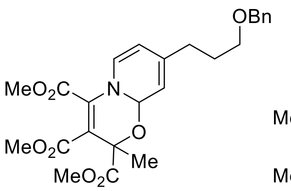

S14

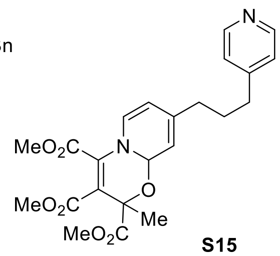

S15

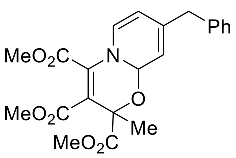

S16

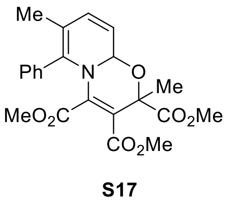

S17

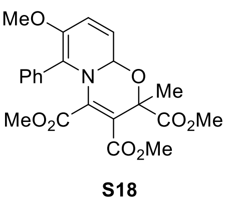

S18

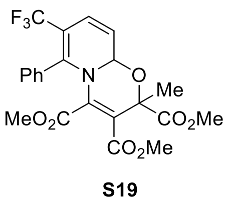

S19

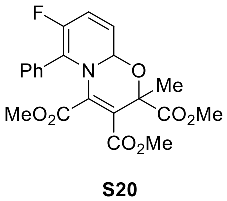

S20

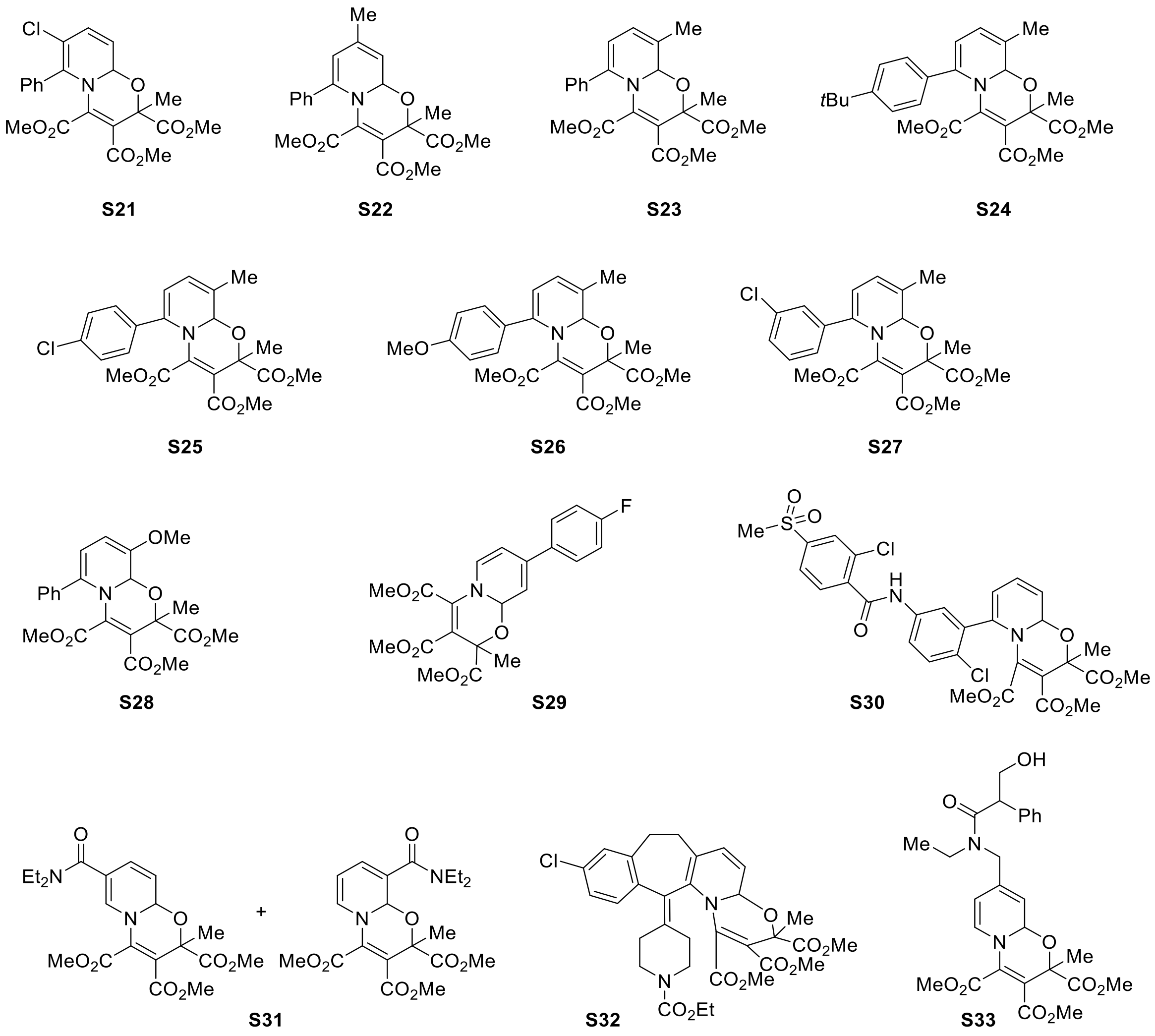

Figure S1. Overview of oxazino pyridines used in this study.

General procedure for redox-neutral dearomatization (GP1)

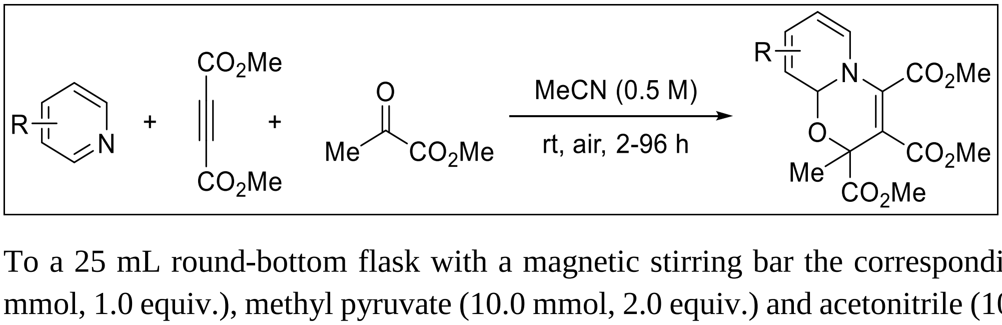

To a 25 mL round-bottom flask with a magnetic stirring bar the corresponding heteroarene (5.0 mmol, 1.0 equiv.), methyl pyruvate (10.0 mmol, 2.0 equiv.) and acetonitrile (10.0 mL, 0.5 M) were added under air atmosphere. Dimethyl acetylenedicarboxylate (10.0 mmol, 2.0 equiv.) was then added dropwise to the stirred reaction mixture. The reaction mixture was allowed to stir at room temperature for 2 to 96 h. After the reaction was complete, as monitored by TLC, the solvent was

removed with a rotary evaporator under reduced pressure and the residue was subjected to flash column chromatography over silica gel to give the corresponding dearomatized heteroarene product.

Substrates **S1-S6**, **S8-S11, S14-S16, S19, S24, S26-S30** (**Figure S1**) were synthesized according to known procedures.[1-5] The analytical data match the reported ones.

<u>Trimethyl 2-methyl-6-phenyl-2*H*,9a*H*-pyrido[2,1-*b*][1,3]oxazine-2,3,4-tricarboxylate (S1):</u> synthesized according to the **GP1** from 2-phenyl pyridine in 48 h.[1]

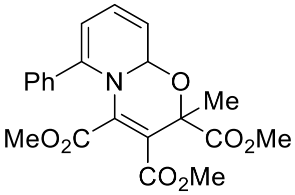

Yield: 83%. **^{1}H NMR** (400 MHz, $CDCl_3$, containing diastereomers, ratio = 1/1) δ 7.52 – 7.15 (m, 5H), 6.55 – 6.35 (m, 1H), 5.76 – 5.64 (m, 1H), 5.59 – 5.20 (m, 2H), 3.90 – 3.61 (m, 6H), 3.13 (s, 3H), 1.99 – 1.56 (m, 3H).

<u>Trimethyl 6-(4-fluorophenyl)-2-methyl-2*H*,9a*H*-pyrido[2,1-*b*][1,3]oxazine-2,3,4-tricarboxylate (S2):</u> synthesized according to the **GP1** from 2-(4-fluorophenyl)pyridine (0.52 g, 3.0 mmol) in 48 h.

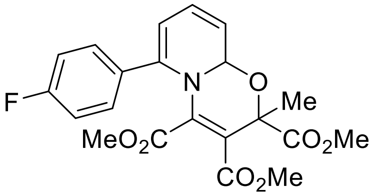

Yield: 80% (1.0 g, containing diasteroisomers, ratio = 1/1). Yellow foam. ***R*f** 0.40 (pentane/EtOAc 4/1); **^{1}H NMR** (300 MHz, $CDCl_3$, all isomers) δ 7.44 (s, 1H), 7.22 (t, *J* = 6.7 Hz, 1H), 7.02 (t, *J* = 8.8 Hz, 2H), 6.45 (ddd, *J* = 16.0, 9.7, 6.1 Hz, 1H), 5.71 (tt, *J* = 5.6, 4.2 Hz, 1H), 5.57 – 5.11 (m, 2H), 3.90 – 3.52 (m, 6H), 3.21 (s, 3H), 1.90 – 1.64 (m, 3H). **^{13}C NMR** (101 MHz, $CDCl_3$, all isomers) δ 171.1, 170.8, 170.1, 165.7, 165.2, 164.0(6), 164.0(4), 163.1, 162.7, 161.5(8), 161.5(7), 141.5, 140.6, 138.5, 137.7, 132.2(9), 132.2(6), 131.9(3), 131.9(0), 127.5, 127.4, 127.1, 126.5, 126.1, 124.9, 123.8, 122.9, 119.8, 115.7, 114.6, 113.8, 104.1, 104.0(7), 101.5, 81.5, 80.5, 78.7, 78.1, 77.5, 77.2, 76.8, 60.4, 53.5, 53.4, 53.2, 52.8, 52.4, 52.3, 52.2, 52.0, 51.8, 50.2, 31.7, 26.3, 24.6, 22.8, 21.0, 19.3, 18.5, 17.0, 14.2, 13.9. **^{19}F NMR** (282 MHz, $CDCl_3$, all isomers) δ -112.1, -112.4. **HRMS** (ESI): calcd. for $[C_{21}H_{21}NF_7]^+$, $[M+H]^+$: 418.1297; found: 418.1294. **IR** (neat, cm^{-1}) = 2952*w*, 2359*w*, 1736*s*, 1714*s*, 1604*m*, 1567*s*, 1506*s*, 1424*s*, 1266*s*, 1224*s*, 1120*s*, 1078*m*, 910*s*, 841*s*, 726*s*, 677*m*, 648*m*, 572*w*, 545*w*.

Trimethyl 6-(4-bromophenyl)-2-methyl-2*H*,9a*H*-pyrido[2,1-*b*][1,3]oxazine-2,3,4-tricarboxylate (S3): synthesized according to the **GP1** from 2-(4-bromophenyl)pyridine in 48 h.[4]

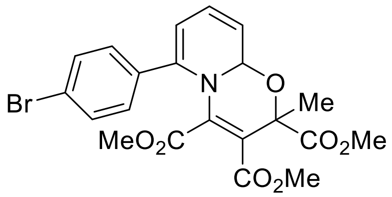

Yield: 92%. **^{1}H NMR** (400 MHz, $CDCl_3$, containing diasteroisomers, ratio = 1:1) 7.49 – 7.06 (m, 4H), 6.51 – 6.37 (m, 1H), 5.78 – 5.20 (m, 3H), 3.85 – 3.63 (m, 6H), 3.24 – 3.17 (m, 3H), 1.91 – 1.61 (m, 3H).

Trimethyl 6-(4-formylphenyl)-2-methyl-2*H*,9a*H*-pyrido[2,1-*b*][1,3]oxazine-2,3,4-tricarboxylate (S4): synthesized according to the **GP1** from 4-formylphenyl pyridine in 48 h.[1]

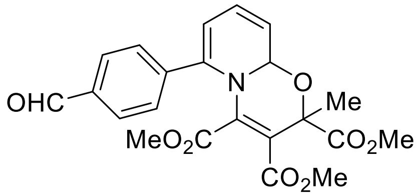

Yield: 96%. **^{1}H NMR** (400 MHz, $CDCl_3$, containing diasteroisomers, ratio = 1/1) 9.98 (s, 1H), 7.86 – 7.79 (m, 2H), 7.69 – 7.56 (m, 1H), 7.43 – 7.34 (m, 1H), 6.52 – 6.39 (m, 1H), 5.80 – 5.70 (m, 1H), 5.53 – 5.25 (m, 2H), 3.94 3.07 (m, 9H), 1.95 – 1.59 (m, 3H).

Trimethyl 6-(2,3-dihydrobenzo[*b*][1,4]dioxin-6-yl)-2-methyl-2*H*,9a*H*-pyrido[2,1-*b*][1,3]oxazine-2,3,4-tricarboxylate (S5): synthesized according to the **GP1** from 2-(2,3-dihydrobenzo[*b*][1,4]dioxin-6-yl)pyridine in 12 h.[4]

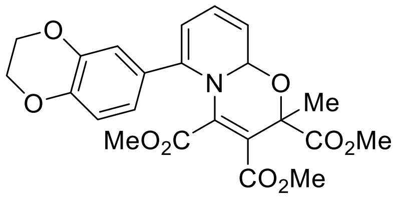

Yield: 34%. **^{1}H NMR** (400 MHz, $CDCl_3$, containing diasteroisomers, ratio = 1/1) δ 7.02 – 6.68 (m, 3H), 6.49 – 6.37 (m, 1H), 5.73 – 5.63 (m, 1H), 5.55 – 5.21 (m, 2H), 4.24 (d, J = 1.8 Hz, 3H), 3.86 – 3.73 (m, 3H), 3.69 – 3.64 (m, 3H), 3.29 – 3.26 (m, 3H), 1.91 – 1.62 (m, 3H).

Trimethyl 6-(2,4-difluorophenyl)-2-methyl-2H,9aH-pyrido[2,1-b][1,3]oxazine-2,3,4-tricarboxylate (S6): synthesized according to the **GP1** from 2-(2,4-difluorophenyl)pyridine in 48 h.[1]

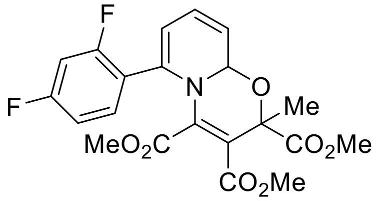

Yield: 94%. **^{1}H NMR** (400 MHz, $CDCl_3$, containing diastereoisomers, ratio = 1/1) δ 7.59 – 7.20 (m, 1H), 6.92 – 6.73 (m, 2H), 6.52 – 6.36 (m, 1H), 5.80 – 5.69 (m, 1H), 5.57 – 5.21 (m, 2H), 3.90 – 3.14 (m, 9H), 1.91 – 1.61 (m, 3H).

<u>Trimethyl 2-methyl-8-phenyl-2*H*,9a*H*-pyrido[2,1-*b*][1,3]oxazine-2,3,4-tricarboxylate (S7):</u> synthesized according to the **GP1** from 4-phenyl pyridine in 12 h.[1]

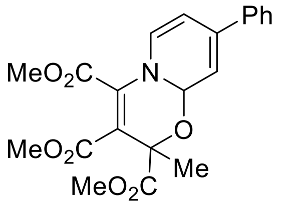

Yield: 98%. **^{1}H NMR** (400 MHz, $CDCl_3$, containing diasteroisomers, ratio = 4/1) δ 7.44 (ddd, *J* = 6.1, 3.4, 1.5 Hz, 2H), 7.40 – 7.29 (m, 3H), 6.45 – 6.37 (m, 1H), 5.97 – 5.73 (m, 2H), 5.65 – 5.58 (m, 1H), 3.95 (s, 3H), 3.80 – 3.69 (m, 6H), 1.78 (s, 3H).

<u>Trimethyl 2-methyl-8-(3-(trimethylsilyl)phenyl)-2*H*,9a*H*-pyrido[2,1-*b*][1,3]oxazine-2,3,4-tricarboxylate (S8):</u> synthesized according to the **GP1** from 4-(3-(trimethylsilyl)phenyl)pyridine (0.91 g, 4.0 mmol) in 12 h.

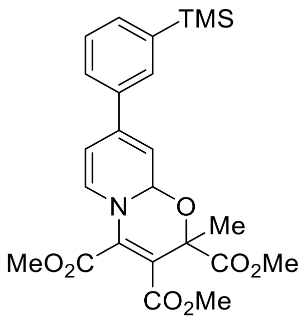

Yield: 43% (0.80 g, containing diasteroisomers, ratio = 4/1). Yellow foam. ***R*f** 0.70 (pentane/EtOAc 2/1); **^{1}H NMR** (400 MHz, $CDCl_3$, all isomers) δ 7.58 (s, 1H), 7.52 – 7.45 (m, 1H), 7.45 – 7.39 (m, 1H), 7.35 (t, *J* = 7.4 Hz, 1H), 6.53 – 5.51 (m, 4H), 3.95 (s, 3H), 3.78 (s, 3H), 3.74 (s, 3H), 1.80 (s, 2H), 1.78 (s, 1H), 0.27 (s, 9H). **^{13}C NMR** (101 MHz, $CDCl_3$, all isomers) δ 171.4, 170.7, 164.8, 163.7, 142.7, 142.2, 141.1, 137.9, 137.8, 133.5, 130.9, 128.1, 126.6, 126.1, 125.9, 113.7, 111.7, 111.4, 102.6, 102.4, 79.9, 79.1, 78.1, 77.0, 53.5, 53.3, 52.9, 52.2, 24.0, 23.9, -1.0. **HRMS** (ESI): calcd. for $[C_{24}H_{30}NO_7Si]^+$, $[M+H]^+$: 472.1786; found: 472.1782. **IR** (neat, cm^{-1}) = 2952*m*, 1739*s*, 1709*s*, 1657*w*, 1573*m*, 1424*m*, 1233*s*, 1201*s*, 1119*s*, 981*m*, 922*w*, 837*s*, 736*s*, 695*w*.

Trimethyl 8-(5-isopropoxy-2-methyl-4-nitrophenyl)-2-methyl-2*H*,9a*H*-pyrido[2,1-*b*][1,3]oxazine-2,3,4-tricarboxylate (S9): synthesized according to the **GP1** from 4-(2-isopropoxy-6-methyl 4-nitrophenyl)pyridine in 24 h.[4]

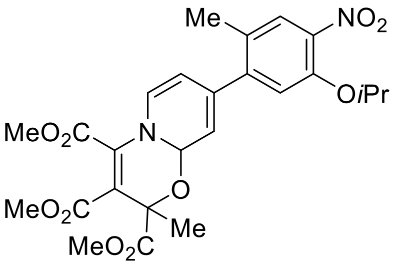

Yield: 94%. **^{1}H NMR** (600 MHz, $CDCl_3$, containing diasteroisomers, ratio = 5/1) δ 7.61 – 7.59 (m, 1H), 6.89 – 6.86 (m, 1H), 6.39 – 6.34 (m, 1H), 5.95 – 5.76 (m, 1H), 5.51 – 5.45 (m, 1H), 5.23 – 5.19 (m, 1H), 4.65 – 4.08 (m, 1H), 3.95 – 3.85 (m, 3H), 3.84 – 3.71 (m, 6H), 2.49 – 2.23 (m, 3H), 1.80 – 1.74 (m, 3H), 1.40 – 1.32 (m, 6H).

Trimethyl 2-methyl-8-(pyridin-2-yl)-2*H*,9a*H*-pyrido[2,1-*b*][1,3]oxazine-2,3,4-tricarboxylate (S10): synthesized according to the **GP1** from 4-(2-pyridinyl)pyridine in 48 h.[1]

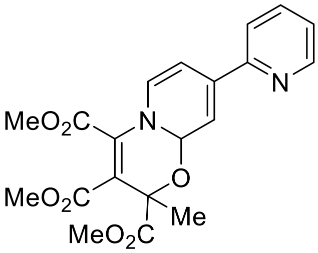

Yield: 92%. **^{1}H NMR** (400 MHz, $CDCl_3$, containing diasteroisomers, ratio = 4/1) δ 8.63 – 8.56 (m, 1H), 7.72 – 7.64 (m, 1H), 7.57 – 7.50 (m, 1H), 7.25 – 7.17 (m, 1H), 6.48 – 6.39 (m, 1H), 6.26 – 6.17 (m, 1H), 6.08 – 6.00 (m, 1H), 6.00 – 5.78 (m, 1H), 3.97 – 3.89 (m, 3H), 3.78 – 3.67 (m, 6H), 1.80 – 1.72 (m, 3H).

Trimethyl 2-methyl-8-(2-methylbenzo[*d*]thiazol-5-yl)-2*H*,9a*H*-pyrido[2,1-*b*][1,3]oxazine-2,3,4-tricarboxylate (S11): synthesized according to the **GP1** from 2-methyl-5-(pyridin-4-yl)benzo[*d*]thiazole (0.66 g, 2.9 mmol) in 12 h.

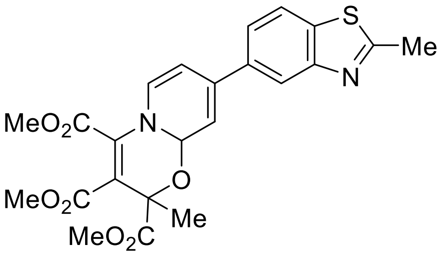

Yield: 46% (0.62 g, containing diasteroisomers, ratio = 2/1). Yellow foam. ***R*f** 0.20 (pentane/EtOAc 1/1); **^{1}H NMR** (300 MHz, $CDCl_3$, all isomers and inseparable unknown impurities not affecting the reaction) δ 7.17 – 6.97 (m, 2H), 6.68 – 6.25 (m, 2H), 5.97 – 5.29 (m, 3H), 3.98 – 3.30 (m, 12H), 2.02 – 1.73 (m, 3H). **^{13}C NMR** (101 MHz, $CDCl_3$, all isomers and inseparable unknown impurities not affecting the reaction) δ 171.2, 169.4, 165.4, 165.1, 164.7, 164.0, 163.6, 163.3, 142.4, 142.0, 141.3, 136.9, 136.5, 134.5, 134.2, 126.2, 125.3, 125.0, 124.5, 123.0, 122.4, 121.6, 120.9, 119.8, 113.9, 111.3, 111.1, 108.9, 108.2, 104.7, 104.0, 103.9, 101.7, 101.6, 79.6, 78.9, 77.5, 77.2, 76.8, 53.6, 53.5, 53.4, 53.0, 52.9, 52.7, 52.2, 27.6, 26.7, 24.8, 23.9, 22.7, 20.3. **HRMS** (ESI): calcd. for $[C_{23}H_{22}N_2O_7SNa]^+$, $[M+Na]^+$: 493.1040; found: 493.1041. **IR** (neat, cm^{-1}) = 2360*s*, 2341*s*, 1698*w*, 1260*s*, 1126*w*, 749*m*, 669*w*.

Trimethyl 8-(benzo[b]thiophen-2-yl)-2-methyl-2H,9aH-pyrido[2,1-b][1,3]oxazine-2,3,4-tricarboxylate (S12): synthesized according to the **GP1** from 4-(benzo[*b*]thiophen-2-yl)pyridine in 12 h.[3]

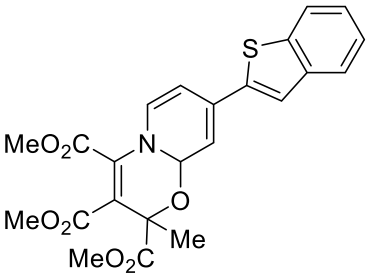

Yield: 60%. **^{1}H NMR** (400 MHz, $CDCl_3$, containing diasteroisomers, ratio = 4/1) δ 7.82 – 7.75 (m, 1H), 7.74 – 7.72 (m, 1H), 7.39 (s, 1H), 7.36 – 7.30 (m, 2H), 6.51 – 6.32 (m, 1H), 6.07 – 5.60 (m, 3H), 3.95 (d, *J* = 1.8 Hz, 3H), 3.80 – 3.73 (m, 6H), 1.80 – 1.78 (m, 3H).

Trimethyl 2-methyl-8-(3-phenylpropyl)-2*H*,9a*H*-pyrido[2,1-*b*][1,3]oxazine-2,3,4-tricarboxylate (S13): synthesized according to the **GP1** from 4-(3-phenylpropyl)pyridine (2.0 g, 10.0 mmol) in 12 h.

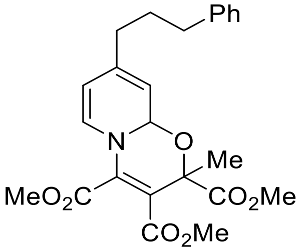

Yield: 84% (3.7 g, containing diasteroisomers, ratio = 4/1). Brown thick oil. ***R*f** 0.50 (pentane/EtOAc 4/1); **^{1}H NMR** (400 MHz, $CDCl_3$, all isomers) δ 7.31 – 7.15 (m, 5H), 6.24 (d, *J* = 7.5 Hz, 1H), 5.86 – 5.05 (m, 3H), 3.92 (s, 3H), 3.82 – 3.65 (m, 6H), 2.62 (t, *J* = 7.7 Hz, 2H), 2.18 (t, *J* = 7.8 Hz, 2H), 1.89 – 1.68 (m, 5H). **^{13}C NMR** (101 MHz, $CDCl_3$, all isomers) δ 171.4, 170.8, 164.9, 164.8, 163.8, 142.8, 142.4, 142.0, 138.5, 138.2, 128.5, 128.4, 125.9, 125.3, 125.1, 113.1,

112.2, 111.3, 111.1, 103.7, 103.6, 79.7, 78.9, 77.8, 76.7, 53.4, 53.2, 52.8, 52.0, 35.3, 34.7, 29.6, 24.0, 23.8. **HRMS** (ESI): calcd. for $[C_{24}H_{28}NO_7]^+$, $[M+H]^+$: 442.1860; found: 442.1860. **IR** (neat, cm^{-1}) = 2950*m*, 1739*s*, 1705*s*, 1667*w*, 1574*s*, 1434*m*, 1263*s*, 1234*s*, 1117*m*, 1003*w*, 982*m*, 922*w*, 846*w*, 782*m*, 736*s*, 700*s*, 664*w*.

Trimethyl 8-(3-(benzyloxy)propyl)-2-methyl-2*H*,9a*H*-pyrido[2,1-*b*][1,3]oxazine-2,3,4-tricarboxylate (S14): synthesized according to the **GP1** from 4-(3-(benzyloxy)propyl)pyridine (1.35 g, 5.9 mmol) in 12 h.

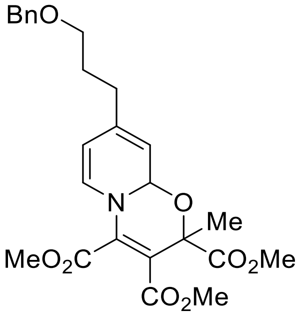

Yield: 64% (1.78 g, containing diasteroisomers, ratio = 5/1). Brown thick oil. ***R*f** 0.60 (pentane/EtOAc 4/1); **^{1}H NMR** (300 MHz, $CDCl_3$, all isomers) δ 7.37 – 7.27 (m, 5H), 6.25 – 6.21 (m, 1H), 5.74 – 5.55 (m, 1H), 5.37 – 5.23 (m, 0H), 5.16 (dd, *J* = 7.6, 1.7 Hz, 1H), 4.49 (s, 2H), 3.92 (s, 3H), 3.75 – 3.7.0 (m, 6H), 3.47 (t, *J* = 6.2 Hz, 2H), 2.24 (t, *J* = 7.6 Hz, 2H), 1.86 – 1.68 (m, 5H). **^{13}C NMR** (101 MHz, $CDCl_3$, major isomer) δ 171.4, 164.8, 163.8, 142.5, 138.6, 138.0, 128.5, 127.8, 127.7, 125.2, 113.1, 111.4, 103.6, 79.7, 73.0, 69.4, 53.4, 52.8, 52.1, 31.8, 28.2, 24.0. **HRMS** (ESI): calcd. for $[C_{25}H_{29}NNaO_8]^+$, $[M+Na]^+$: 494.1785; found: 494.1786. **IR** (neat, cm^{-1}) = 2950*m*, 2859*br*, 1740*s*, 1707*s*, 1668*w*, 1577*s*, 1452*m*, 1265*s*, 1239*s*, 1121*m*, 1072*w*, 982*w*, 848*w*, 748*s*, 699*m*.

Trimethyl 2-methyl-8-(3-(pyridin-4-yl)propyl)-2*H*,9a*H*-pyrido[2,1-*b*][1,3]oxazine-2,3,4-tricarboxylate (S15): synthesized according to the **GP1** from 1,3-di(pyridin-4-yl)propane with 1.0 equiv. DMAD and MP in 48 h.[1]

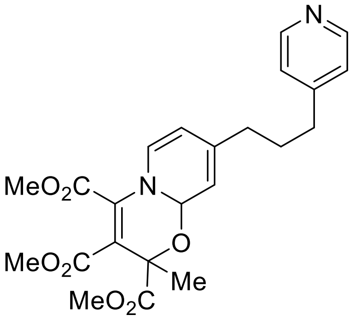

Yield: 56%. **^{1}H NMR** (400 MHz, $CDCl_3$, containing diasteroisomers, ratio = 4/1) δ 8.51 – 8.43 (m, 2H), 7.12 – 7.06 (m, 2H), 6.28 – 6.21 (m, 1H), 5.78 – 5.54 (m, 1H), 5.35 – 5.29 (m, 1H), 5.15

– 5.09 (m, 1H), 3.94 – 3.84 (m, 3H), 3.77 – 3.65 (m, 6H), 2.61 (t, *J* = 7.7 Hz, 2H), 2.17 (t, *J* = 7.6 Hz, 2H), 1.86 – 1.76 (m, 2H), 1.76 – 1.67 (m, 3H).

Trimethyl 8-benzyl-2-methyl-2*H*,9a*H*-pyrido[2,1-*b*][1,3]oxazine-2,3,4-tricarboxylate (S16): synthesized according to the **GP1** from 4-benzylpyridine in 12 h.[4]

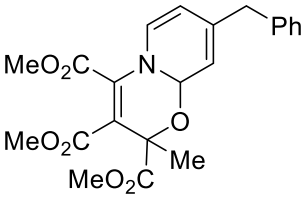

Yield: 98%. **^{1}H NMR** (400 MHz, $CDCl_3$, containing diasteroisomers, ratio = 4/1) δ 7.38 – 7.13 (m, 5H), 6.26 – 5.07 (m, 4H), 3.92 – 3.86 (m, 3H), 3.75 – 3.67 (m, 6H), 3.52 – 3.40 (m, 2H), 1.77 – 1.69 (m, 3H).

Trimethyl 2,7-dimethyl-6-phenyl-2*H*,9a*H*-pyrido[2,1-*b*][1,3]oxazine-2,3,4-tricarboxylate (S17): synthesized according to the **GP1** from 3-methyl-2-phenylpyridine in 48 h.[1]

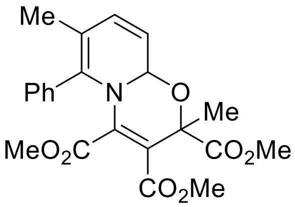

Yield: 85%. **^{1}H NMR** (400 MHz, $CDCl_3$, containing diasteroisomers, ratio = 2/1) δ 7.60 – 7.28 (m, 5H), 6.43 – 6.30 (m, 1H), 5.80 – 5.70 (m, 1H), 5.49 – 5.15 (m, 1H), 3.86 – 3.13 (m, 9H), 2.37 – 1.85 (m, 3H), 1.66 – 1.57 (m, 3H).

Trimethyl 7-methoxy-2-methyl-6-phenyl-2*H*,9a*H*-pyrido[2,1-*b*][1,3]oxazine-2,3,4-tricarboxylate (S18): synthesized according to the **GP1** from 3-methoxy-2-phenylpyridine in 48 h.[3]

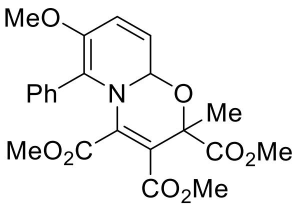

Yield: 84%. Yellow foam. ***R*f** 0.20 (pentane/EtOAc 1/1); **^{1}H NMR** (400 MHz, $CDCl_3$, containing diasteroisomers, ratio = 5/1) δ 7.59 – 7.46 (m, 2H), 7.38 – 7.19 (m, 3H), 6.55 – 4.88 (m, 3H), 3.99 (s, CH_3 major), 3.94 (s, CH_3 minor), 3.72 (s, CH_3 minor), 3.62 (s, CH_3 major), 3.61 (s, CH_3 minor), 3.58 (s, CH_3 major), 3.34 (s, CH_3 minor), 3.15 (s, CH_3 major), 1.89 (s, CH_3 minor), 3.86 (s, CH_3 major).

Trimethyl 2-methyl-6-phenyl-7-(trifluoromethyl)-2*H*,9a*H*-pyrido[2,1-*b*][1,3]oxazine-2,3,4-tricarboxylate (S19): synthesized according to the **GP1** from 3-trifluoromethyl-2-phenylpyridine (1.1 g, 5.0 mmol) in 48 h.

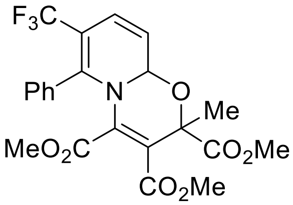

Yield: 69% (1.60 g, containing diasteroisomers, ratio = 3/2). Yellow foam. ***R*f** 0.50 (pentane/EtOAc 3/1); **^{1}H NMR** (300 MHz, $CDCl_3$, all isomers) δ 7.49 – 7.10 (m, 5H), 6.70 – 6.43 (m, 1H), 5.90 – 5.14 (m, 2H), 3.95 – 3.49 (m, 6H), 3.21 – 3.20 (m, 3H), 1.89 – 1.60 (m, 3H). **^{13}C NMR** (101 MHz, $CDCl_3$, all isomers) δ 170.5, 169.5, 165.1, 164.7, 162.8, 162.4, 142.5, 142.2, 142.1, 141.4(9), 141.4(5), 140.6, 139.7, 132.2, 132.1, 132.0(2), 131.9(9), 131.6, 131.5, 129.9, 129.8(7), 129.8, 129.3, 128.8, 128.6, 128.4, 128.2, 127.8(9), 127.8(5), 127.7, 127.5, 125.6, 125.5, 124.4, 124.3(6), 124.3, 123.9, 123.8(5), 123.8, 123.7(6), 122.9, 122.8, 120.1, 118.7, 114.2, 113.4, 108.4, 105.7, 105.4, 105.3, 105.1, 105.0, 104.8, 80.6, 79.6, 79.0, 78.4, 53.7, 53.4(4), 53.3(9), 53.1, 52.7(4), 52.7(3), 52.6(1), 52.5(8), 32.1, 29.8, 24.5, 23.0, 22.8, 22.5, 14.3. **^{19}F NMR** (376 MHz, $CDCl_3$, all isomers) δ -55.2, -55.4. **HRMS** (ESI): calcd. for $[C_{22}H_{20}NO_7F_3Na]^+$, $[M+Na]^+$: 490.1084; found: 490.1087. **IR** (neat, cm^{-1}) = 2953*w*, 1730*s*, 1570*m*, 1309*s*, 1265*s*, 1235*s*, 1241*s*, 1152*s*, 1112*s*, 1072*s*, 978*m*, 933*s*, 737*s*, 701*s*.

Trimethyl 7-fluoro-2-methyl-6-phenyl-2*H*,9a*H*-pyrido[2,1-*b*][1,3]oxazine-2,3,4-tricarboxylate (S20): synthesized according to the **GP1** from 3-fluoro-2-phenylpyridine (0.87 g, 5.0 mmol) in 48 h.

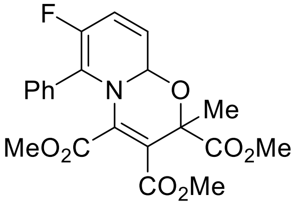

S17

Yield: 86% (1.80 g, containing diasteroisomers, ratio = 4/1). Yellow foam. ***R*f** 0.40 (pentane/EtOAc 4/1); **^{1}H NMR** (300 MHz, $CDCl_3$, all isomers) δ 7.58 – 7.30 (m, 5H), 6.69 – 6.19 (m, 1H), 5.84 – 5.58 (m, 1H), 5.54 – 5.08 (m, 1H), 4.02 – 3.74 (m, 3H), 3.69 – 3.52 (m, 3H), 3.35 – 2.96 (m, 3H), 1.97 – 1.47 (m, 3H). **^{13}C NMR** (101 MHz, $CDCl_3$, all isomers) δ 171.1, 170.8, 170.0, 169.7, 165.6, 165.1, 165.0, 164.1, 163.0, 162.7, 152.8, 150.2, 144.1, 141.8(4), 141.7(9), 140.8, 140.4, 136.3(4), 136.3(1), 129.6, 129.4, 129.2, 129.1, 129.0, 128.5, 128.4, 128.3, 127.4(8), 127.4(5), 126.9, 126.8, 126.3(8), 126.3(5), 124.1(7), 124.0, 123.6, 123.5, 123.2(2), 123.1(7), 123.1(3), 122.9(3), 122.9(0), 122.8(8), 122.8(5), 122.7, 122.4, 120.4, 120.3, 116.7(2), 116.6(5), 115.9, 115.8, 112.1, 101.0, 100.8, 100.6, 100.4, 98.3(1), 98.2(5), 97.9(4), 97.9, 87.2, 87.0, 80.3, 79.4, 78.8, 78.2, 77.5, 77.2,

76.8, 74.6, 60.4, 53.4(4), 53.4(1), 53.2, 52.8, 52.3, 52.2(6), 52.2, 52.1(4), 52.0, 51.9, 51.8, 51.7, 50.1, 39.7, 31.9, 29.7(0), 29.6(5), 29.5, 29.4, 26.3, 24.5, 23.3, 22.8, 22.7, 21.0, 14.2, 14.1. **^{19}F NMR** (376 MHz, $CDCl_3$, all isomers) δ -151.0, -151.7. **HRMS** (ESI): calcd. for $[C_{21}H_{20}NO_7FNa]^+$, $[M+Na]^+$: 440.1116; found: 440.1111. **IR** (neat, cm^{-1}) = 2952*w*, 2360*m*, 2342*m*, 1735*s*, 1714*s*, 1581*m*, 1427*s*, 1238*s*, 1223*s*, 1121*s*, 956*m*, 911*s*, 729*s*, 698*s*, 660*m*.

Trimethyl 7-chloro-2-methyl-6-phenyl-2*H*,9a*H*-pyrido[2,1-*b*][1,3]oxazine-2,3,4-tricarboxylate (S21): synthesized according to the **GP1** from 3-chloro-2-phenylpyridine (1.90 g, 10.0 mmol) in 48 h.

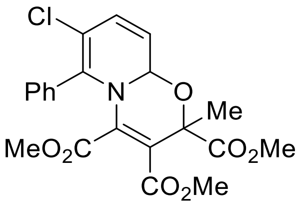

Yield: 67% (2.90 g, containing diasteroisomers, ratio = 2/1). Yellow solid. ***R*f** 0.30 (pentane/EtOAc 4/1); **^{1}H NMR** (300 MHz, $CDCl_3$, all isomers) δ 7.55 – 7.03 (m, 5H), 6.62 – 6.15 (m, 1H), 5.86 – 5.13 (m, 2H), 3.97 – 3.56 (m, 6H), 3.16 (s, 3H), 1.96 – 1.69 (m, 3H). **^{13}C NMR** (101 MHz, $CDCl_3$, all isomers) δ 170.8, 170.0, 168.9, 165.4, 165.1, 163.1, 162.7, 141.7, 134.7, 132.7, 132.4, 130.3, 129.7, 129.4, 129.3, 128.7, 128.3, 127.9, 126.3, 124.1, 121.3, 118.7, 115.9, 115.1, 109.2, 100.8, 80.4, 79.5, 78.9, 78.3, 53.3, 52.9, 52.5, 52.3, 24.5, 23.0, 22.8. **HRMS** (ESI): calcd. for $[C_{21}H_{21}NO_7ClNa]^+$, $[M+Na]^+$: 456.0821; found: 456.0821. **IR** (neat, cm^{-1}) = 2859*w*, 1744*s*, 1716*s*, 1275*s*, 1237*s*, 1209*m*, 1120*m*, 764*s*, 749*s*, 700*w*. **Mp**: 158.2 – 159.2 °C.

Trimethyl 2,8-dimethyl-6-phenyl-2*H*,9a*H*-pyrido[2,1-*b*][1,3]oxazine-2,3,4-tricarboxylate (S22): synthesized according to the **GP1** from 4-methyl-2-phenylpyridine (0.68 g, 4.0 mmol) in 48 h.

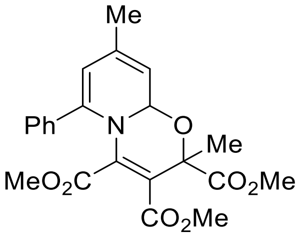

Yield: 42% (0.70 g, containing diasteroisomers, ratio = 2/1). Yellow foam. ***R*f** 0.20 (pentane/EtOAc 4/1); **^{1}H NMR** (400 MHz, $CDCl_3$, all isomers) δ 7.49 – 7.17 (m, 5H), 5.57 – 5.07 (m, 3H), 3.85 – 3.75 (m, 3H), 3.66 – 3.65 (m, 3H), 3.13 (s, 3H), 1.96 – 1.62 (m, 6H). **^{13}C NMR** (101 MHz, $CDCl_3$, all isomers) δ 171.2, 170.4, 166.0, 165.6, 163.2, 162.9, 149.6, 141.6, 140.7, 139.0, 138.3, 136.6, 136.2, 135.9, 135.8(8), 128.7, 128.6, 128.5(6), 128.4, 127.1, 124.5, 123.5, 110.6, 109.9, 107.8, 82.2, 81.2, 78.7, 78.1, 53.3, 52.9, 52.5, 52.3, 52.2, 29.8, 24.8, 22.9, 21.3(4), 21.2(7). **HRMS** (ESI): calcd. for $[C_{22}H_{24}NO_7]^+$, $[M+H]^+$: 414.1547; found: 414.1544. **IR** (neat, cm^{-1}) = 2952*w*, 1735*s*,

1631*w*, 1579*w*, 1434*s*, 1374*w*, 1235*s*, 1203*s*, 1121*m*, 1069*w*, 979*w*, 847*w*, 766*m*, 736*m*, 700*m*, 673*w*.

Trimethyl 2,9-dimethyl-6-phenyl-2*H*,9a*H*-pyrido[2,1-*b*][1,3]oxazine-2,3,4-tricarboxylate (S23): synthesized according to the **GP1** from 5-methyl-2-phenylpyridine in 48 h.[3]

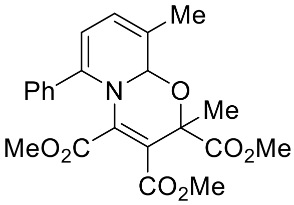

Yield: 73%. **^{1}H NMR** (400 MHz, $CDCl_3$, containing diasteroisomers, ratio = 1.2/1) δ 7.48 – 7.38 (m, 1H), 7.35 – 7.27 (m, 3H), 7.25 – 7.20 (m, 1H), 6.30 – 6.09 (m, 1H), 5.37 – 5.08 (m, 2H), 3.85 – 3.77 (m, 3H), 3.66 – 3.65 (m, 3H), 3.13 (s, 3H), 2.02 – 1.95 (m, 3H), 1.92 – 1.66 (m, 3H).

Trimethyl 6-(4-(*tert*-butyl)phenyl)-2,9-dimethyl-2*H*,9a*H*-pyrido[2,1-*b*][1,3]oxazine-2,3,4-tricarboxylate (S24): synthesized according to the **GP1** from 2-(4-(*tert*-butyl)phenyl)-5-methylpyridine (0.90 g, 4.0 mmol) in 60 h.

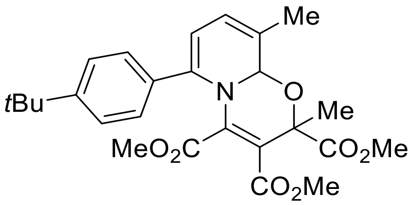

Yield: 89% (1.68 g, containing diasteroisomers, ratio = 2/1). Brown thick oil. ***R*f** 0.50 (pentane/EtOAc 4/1); **^{1}H NMR** (400 MHz, $CDCl_3$, all isomers) δ 7.38 – 7.15 (m, 4H), 6.20 – 6.12 (m, , 1H), 5.43 – 4.99 (m, 2H), 3.86 – 3.77 (m, 3H), 3.66 – 3.65 (m, 3H), 3.09 (s, 3H), 1.97 – 1.95 (m, 3H), 1.92 – 1.66 (m, 3H), 1.30 (s, 9H). **^{13}C NMR** (101 MHz, $CDCl_3$, all isomers) δ 171.3, 170.6, 166.2, 165.7, 163.4, 163.1, 151.6, 151.5, 142.1, 141.4, 137.0, 136.3, 133.4, 133.1, 128.4, 126.6, 126.0, 125.6, 125.3, 123.7, 123.6, 123.2, 122.8, 122.8, 122.6, 104.2, 104.2, 85.3, 84.5, 78.9, 78.5, 53.3, 52.7, 52.4, 52.3, 52.2, 52.2, 34.8, 34.8, 31.4, 31.4, 29.9, 24.7, 22.7, 19.0, 18.8. **HRMS** (ESI): calcd. for $[C_{26}H_{32}NO_7]^+$, $[M+H]^+$: 470.2173; found: 470.2166. **IR** (neat, cm^{-1}) = 2954*m*, 1732*s*, 1605*w*, 1508*w*, 1435*m*, 1363*w*, 1260*s*, 1203*s*, 1124*m*, 1075*w*, 1021*w*, 981*w*, 838*w*, 735*m*, 703*w*.

Trimethyl 6-(4-chlorophenyl)-2,9-dimethyl-2*H*,9a*H*-pyrido[2,1-*b*][1,3]oxazine-2,3,4-tricarboxylate (S25): synthesized according to the **GP1** from 2-(4-chlorophenyl)-5-methylpyridine (0.80 g, 3.9 mmol) in 60 h.

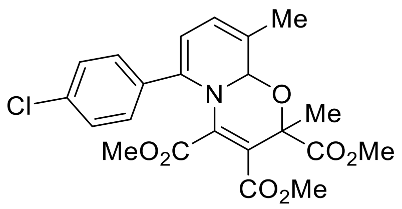

Yield: 93% (1.62 g, containing diasteroisomers, ratio = 1/1). Orange foam. ***R*f** 0.30 (pentane/EtOAc 4/1); **^{1}H NMR** (400 MHz, $CDCl_3$, all isomers) δ 7.41 – 7.33 (m, 1H), 7.29 (d, *J* = 8.8 Hz, 2H), 7.16 (d, *J* = 8.0 Hz, 1H), 6.18 – 6.10 (m, 1H), 5.51 – 4.89 (m, 2H), 3.84 – 3.77 (m, 3H), 3.67 – 3.66 (m, 3H), 3.28 – 3.18 (m, 3H), 1.99 – 1.92 (m, 3H), 1.90 – 1.65 (m, 3H). **^{13}C NMR** (101 MHz, $CDCl_3$, all isomers) δ 171.2, 170.4, 165.9, 165.6, 163.3(1), 162.9(5), 141.2, 140.8, 135.8, 135.2, 134.8, 134.6, 134.5, 130.0, 128.5(8), 128.5(8), 124.5, 124.4, 123.6, 123.0, 122.3, 104.8, 85.1, 84.3, 79.0, 78.6, 53.3, 52.8, 52.5, 52.3(7), 52.3(5), 52.3, 29.8, 24.7, 22.6, 19.0, 18.8. **HRMS** (ESI): calcd. for $[C_{22}H_{22}N\ O_7Na]^+$, $[M+Na]^+$: 470.0977; found: 470.0974. **IR** (neat, cm^{-1}) = 2952*w*, 1737*s*, 1581*m*, 1488*w*, 1434*m*, 1373*w*, 1234*s*, 1207*s*, 1175*m*, 1124*s*, 1075*m*, 1014*w*, 979*w*, 835*m*, 735*s*, 673*w*.

Trimethyl 6-(4-methoxyphenyl)-2,9-dimethyl-2*H*,9a*H*-pyrido[2,1-*b*][1,3]oxazine-2,3,4-tricarboxylate (S26): synthesized according to the **GP1** from 2-(4-methoxyphenyl)-5-methylpyridine (0.80 g, 4.0 mmol) in 60 h.

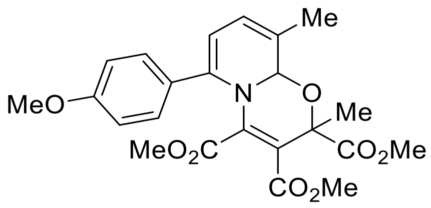

Yield: 45% (0.8 g, containing diasteroisomers, ratio = 2/1). Yellow foam. ***R*f** 0.20 (pentane/EtOAc 4/1); **^{1}H NMR** (400 MHz, $CDCl_3$, all isomers) δ 7.40 – 7.08 (m, 2H), 6.86 – 6.76 (m, 2H), 6.18 – 6.11 (m, 1H), 5.49 – 4.94 (m, 2H), 3.89 – 3.53 (m, 6H), 3.66 – 3.65 (m, 3H), 3.19 (s, 3H), 1.97 – 1.63 (m, 6H). **^{13}C NMR** (101 MHz, $CDCl_3$, all isomers) δ 171.3, 170.5, 166.1, 165.7, 163.3, 162.9(5), 159.9, 142.0, 141.4, 136.8, 136.1, 130.1, 128.7, 128.4, 125.5, 123.8, 123.6, 123.3, 123.0, 122.6, 122.5(8), 117.0, 114.4, 113.7, 104.1, 104.0, 85.3, 84.5, 78.9, 78.5, 55.5, 55.4, 53.3, 52.7, 52.4, 52.3(3), 52.2(9), 52.2, 29.8, 24.7, 22.7, 21.2, 19.0, 18.8. **HRMS** (ESI): calcd. for $[C_{23}H_{25}NO_7Na]^+$, $[M+Na]^+$: 466.1472; found: 466.1461. **IR** (neat, cm^{-1}) = 2953*w*, 1733*s*, 1604*m*, 1510*w*, 1435*m*, 1340*w*, 1256*s*, 1173*s*, 1123*m*, 1074*w*, 1026*w*, 981*w*, 836*w*, 733*s*, 701*w*.

Trimethyl 6-(3-chlorophenyl)-2,9-dimethyl-2*H*,9a*H*-pyrido[2,1-*b*][1,3]oxazine-2,3,4-tricarboxylate (S27): synthesized according to the **GP1** from 2-(3-chlorophenyl)-5-methylpyridine (0.82 g, 4.0 mmol) in 60 h.

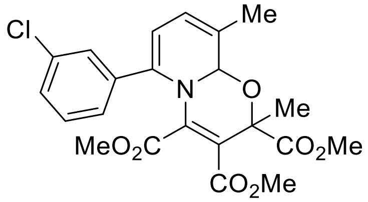

Yield: 50% (0.9 g, containing diasteroisomers, ratio = 1/1). Yellow foam. ***R*f** 0.50 (pentane/EtOAc 4/1); **^{1}H NMR** (400 MHz, $CDCl_3$, all isomers) δ 7.55 – 6.88 (m, 4H), 6.26 – 6.10 (m, 1H), 5.38 – 4.91 (m, 2H), 3.88 – 3.77 (m, 3H), 3.68 – 3.67 (m, 3H), 3.21 (s, 3H), 2.05 – 1.63 (m, 6H). **^{13}C NMR** (101 MHz, $CDCl_3$, all isomers) δ 171.1, 170.2, 165.8, 165.4, 163.1, 162.8, 141.2, 140.7, 137.9, 137.5, 135.4, 134.8, 134.2, 134.0, 129.7, 129.5, 128.5, 128.3, 126.5, 124.7, 124.0, 123.8, 123.3, 122.7, 122.1, 105.0, 104.9, 85.0, 84.1, 78.9, 78.5, 53.1, 52.6, 52.4, 52.2(2), 52.1(9), 29.7, 24.5, 22.5, 18.9, 18.7. **HRMS** (ESI): calcd. for $[C_{22}H_{22}NO_7ClNa]^+$, $[M+Na]^+$: 470.0977; found: 470.0967. **IR** (neat, cm^{-1}) = 2953*w*, 1737*s*, 1595*w*, 1434*m*, 1376*w*, 1249*s*, 1203*s*, 1125*s*, 1074*m*, 978*w*, 783*w*, 738*m*, 699*w*.

Trimethyl 9-methoxy-2-methyl-6-phenyl-2*H*,9a*H*-pyrido[2,1-*b*][1,3]oxazine-2,3,4-tricarboxylate (S28): synthesized according to the **GP1** from 5-methoxy-2-phenylpyridine in 60 h.[4]

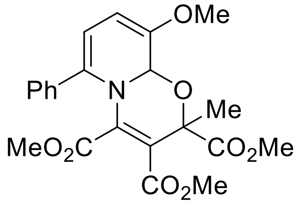

Yield: 89%. **^{1}H NMR** (400 MHz, $CDCl_3$, containing diasteroisomers, ratio = 1.3/1) δ 7.45 – 7.18 (m, 5H), 5.60 – 5.10 (m, 3H), 3.84 – 3.75 (m, 3H), 3.71 – 3.69 (m, 3H), 3.65 – 3.62 (m, 3H), 3.12 (s, 3H), 1.95 – 1.64 (m, 3H).

Trimethyl 8-(4-fluorophenyl)-2-methyl-2*H*,9a*H*-pyrido[2,1-*b*][1,3]oxazine-2,3,4-tricarboxylate (S29): synthesized according to the **GP1** from 4-(4-fluorophenyl)pyridine in 16 h.[4]

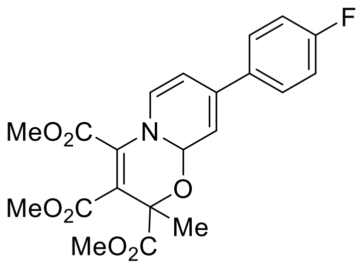

Yield: 85%. **^{1}H NMR** (500 MHz, $CDCl_3$, containing diastereoisomers, ratio = 4.7/1) δ 7.44 – 7.38 (m, 2H), 7.08 – 7.02 (m, 2H), 6.44 – 6.38 (m, 1H), 5.94 – 5.52 (m, 3H), 3.96 – 3.93 (m, 3H), 3.78 – 3.72 (m, 6H), 1.80 – 1.75 (m, 3H).

Trimethyl 6-(2-chloro-5-(2-chloro-4-(methylsulfonyl)benzamido)phenyl)-2-methyl-2*H*,9a*H*-pyrido[2,1-*b*][1,3]oxazine-2,3,4-tricarboxylate (S30): synthesized according to the **GP1** from vismodegib in 48 h.[1]

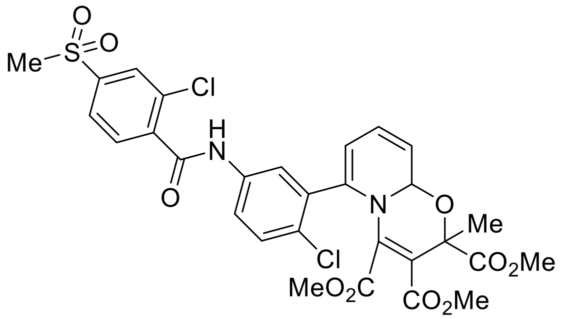

Yield: 87%. **^{1}H NMR** (400 MHz, $CDCl_3$, containing diasteroisomers and rotamers, ratio = 8/4/2/1) δ 8.67 – 8.39 (m, 1H), 8.11 7.31 (m, 6H), 6.56 – 6.36 (m, 1H), 5.79 – 5.22 (m, 3H), 3.84 – 3.57 (m, 6H), 3.42 – 3.23 (m, 3H), 3.12 3.02 (m, 3H), 1.92 – 1.59 (m, 3H).

Trimethyl 7-(diethylcarbamoyl)-2-methyl-2*H*,9a*H*-pyrido[2,1-*b*][1,3]oxazine-2,3,4-tricarboxylate and trimethyl 9-(diethylcarbamoyl)-2-methyl-2*H*,9a*H*-pyrido[2,1-*b*][1,3]oxazine-2,3,4-tricarboxylate (S31): synthesized according to the **GP1** from nikethamide in 48 h.[1]

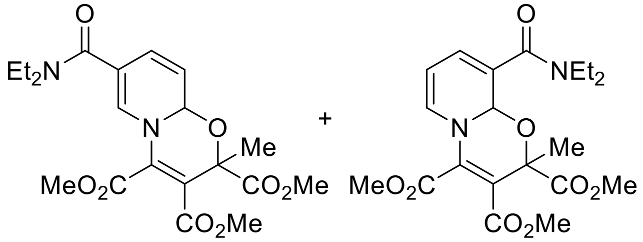

Yield: 99%. **^{1}H NMR** (400 MHz, $CDCl_3$, containing regioisomers and diasteroisomers, ratio = 6/3/2/1) δ 6.72 – 5.21 (m, 4H), 3.93 – 3.85 (m, 3H), 3.78 – 3.66 (m, 6H), 3.54 – 3.27 (m, 4H), 1.75 – 1.65 (m, 3H), 1.20 – 1.08 (m, 6H).

Trimethyl 10-chloro-13-(1-(ethoxycarbonyl)piperidin-4-ylidene)-3-methyl-4a,7,8,13-tetrahydro-3*H*-benzo[4',5']cyclohepta[1',2':5,6]pyrido[2,1-*b*][1,3]oxazine-1,2,3-tricarboxylate (S32): synthesized according to the **GP1** from loratadine in 12 h.[1]

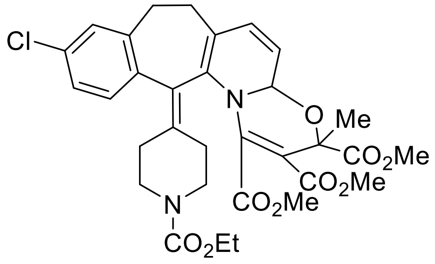

Yield: 97%. **^{1}H NMR** (400 MHz, $CDCl_3$, containing diasteroisomers, ratio = 2/1) δ 7.36 – 7.02 (m, 3H), 6.34 – 6.20 (m, 1H), 5.84 – 5.35 (m, 2H), 4.31 – 3.76 (m, 15H), 3.26 – 2.19 (m, 8H), 2.17 – 1.90 (m, 3H), 1.37 (t, J = 7.1 Hz, 3H).

<u>Trimethyl 8-((*N*-ethyl-3-hydroxy-2-phenylpropanamido)methyl)-2-methyl-2*H*,9a*H*-pyrido[2,1-*b*][1,3]oxazine-2,3,4-tricarboxylate (S33):</u> synthesized according to the **GP1** from tropicamide in 48 h.[1]

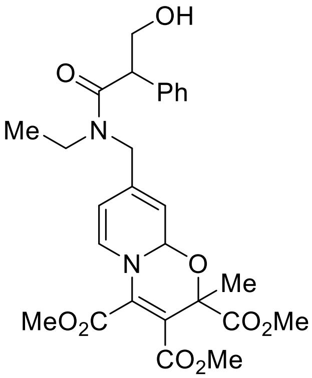

Yield: 96%. **^{1}H NMR** (400 MHz, $CDCl_3$, containing diastereomers and rotamers, ratio = 5/4/4/4//3/2/1/1) δ 7.36 – 7.08 (m, 5H), 6.32 – 6.16 (m, 1H), 5.85 – 4.71 (m, 3H), 4.40 – 3.44 (m, 16H), 3.31 – 2.92 (m, 2H), 2.00 – 1.66 (m, 3H), 1.13 – 0.81 (m, 3H).

General procedure for *meta*-C–H azidation (GP2):

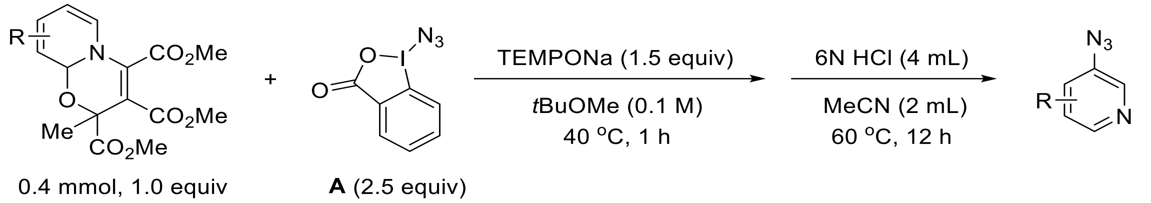

Failed cases:

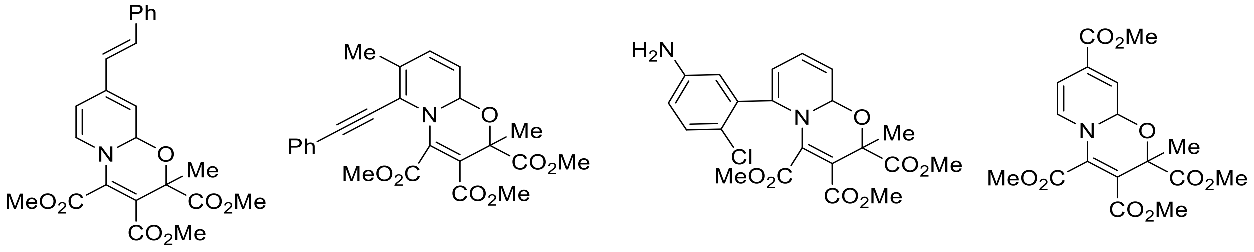

messy | decomposed | pyridine starting material recovered

Preparation of TEMPONa solution: TEMPONa was prepared according to a literature known procedure.[6] An oven dried Schlenk tube was charged with freshly cleaned (with dry pentane) elemental sodium metal (0.44 g, 19 mmol, 1.4 equiv.) which was then melted to sodium mirror using a heating gun at 200 °C. The Schlenk tube was cooled to rt, and dry THF (16 mL), TEMPO (2.12 g, 13.6 mmol, 1 equiv.) and naphthalene (170 mg, 0.70 mmol, 0.1 equiv.) were added under argon atmosphere. The reaction mixture was stirred at room temperature until a blueblack colour persisted (generally 1-2 h). TEMPONa solution can be stored under argon for several days in the fridge without any significant decomposition.

***meta*-C–H azidation of pyridines:** To an oven-dried 20 mL Schlenk tube, dearomatized pyridine (0.4 mmol, 1.0 equiv.) and 1-azido-1λ^3-benzo[*d*][1,2]iodaoxol-3(1*H*)-one **A** (1.0 mmol, 2.5 equiv.) were added. The vial was sealed with a septum and put under vacuum, followed by flushing with Ar gas 3 times. *Tert*-butyl methylether (4.0 mL, 0.1 M) was added to the mixture under Ar protection. The reaction mixture was heated with a water bath at 40 °C. Freshly prepared TEMPONa-solution (0.71 mL, 1.5 equiv., 0.85 M in THF) was added dropwise via syringe over the period of 30 min. Then, the reaction mixture was stirred at 40 °C for another 30 min.

Caution: For safety reasons, the reaction was carried out behind an anti-blast shield.[7]

The reaction was filtered and washed with EA (5 mL x 3). The filtrate was combined and the organic solvent was removed with a rotary evaporator under reduced pressure to give a colloidal residue.

Note: for lager scales, the foam-like residue was easily obtained under vacuum drying for a few minutes.

MeCN (2.0 mL) and 6N HCl (4.0 mL) were then added to the residue and stirred at 60 °C for 12 h. Then, the reaction mixture was basified with saturated Na_2CO_3 aqueous solution and extracted with DCM (50 mL x 3). The combined organic phase was dried over $MgSO_4$ and filtered. The solvent was removed under reduced pressure and the residue was purified by column chromatography on silica gel to give pure product.

5-Azido-2-phenylpyridine (1): synthesized according to **GP2** from **S1** (160.0 mg, 400.0 μmol) run for 1 h at 40 °C.

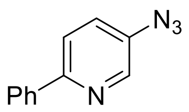

Yield: 68% (53.0 mg). Brown solid. R_f 0.40 (pentane/EtOAc 20/1); **^{1}H NMR** (300 MHz, $CDCl_3$) δ 8.43 (dd, *J* = 2.8, 0.8 Hz, 1H), 8.17 – 7.88 (m, 2H), 7.74 (dd, *J* = 8.4, 0.8 Hz, 1H), 7.59 – 7.34 (m, 4H). **^{13}C NMR** (101 MHz, $CDCl_3$) δ 154.2, 141.2, 138.6, 135.7, 129.1, 129.0, 126.8(1), 126.7(8), 121.1. **HRMS** (ESI): calcd. for $[C_{11}H_9N_4]^+$, $[M+H]^+$: 197.0822; found: 197.0824. **IR** (neat, cm^{-1}) = 2123*s*, 2096*s*, 1560*w*, 1473*m*, 1446*m*, 1389*m*, 1307*m*, 1290*s*, 1230*w*, 1151*w*, 1015*w*, 830*m*, 778*s*, 692*s*. **Mp:** 57.7 – 58.7 °C.

For 5.0 mmol scale: **S1** (2.0 g, 5.0 mmol), **A** (3.60 g, 12.5 mmol), TEMPONa in THF (9.0 mL, 7.5 mmol), *t*BuOMe (40 mL), 40 °C, 1h. 5-Azido-2-phenylpyridine (**1**) was obtioned with 784 mg, 80%.

5-Azido-2-(4-fluorophenyl)pyridine (2): synthesized according to **GP2** from **S2** (167.0 mg, 400.0 μmol) run for 1 h at 40 °C.

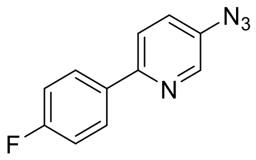

Yield: 50% (43.0 mg). Brown semisolid. R_f 0.50 (pentane/EtOAc 9/1); **^{1}H NMR** (400 MHz, $CDCl_3$) δ 8.41 (d, *J* = 2.8 Hz, 1H), 8.14 – 7.80 (m, 2H), 7.68 (d, *J* = 8.4 Hz, 1H), 7.41 (ddd, *J* = 8.4, 2.8, 0.8 Hz, 1H), 7.15 (t, *J* = 8.4 Hz, 2H). **^{13}C NMR** (101 MHz, $CDCl_3$) δ 163.7 (d, $^1J_{C–F}$ = 248.8 Hz), 153.2, 141.2, 135.7, 134.8 (d, $^4J_{C–F}$ = 3.4 Hz), 128.6 (d, $^3J_{C–F}$ = 8.3 Hz), 126.9, 120.7, 115.9 (d, $^2J_{C–F}$ = 21.7 Hz). **^{19}F NMR** (282 MHz, $CDCl_3$) δ -112.9. **HRMS** (ESI): calcd. for $[C_{11}H_8FN_4]^+$, $[M+H]^+$: 215.0728; found: 215.0726. **IR** (neat, cm^{-1}) = 2105*s*, 1601*m*, 1512*m*, 1473*s*, 1415*w*, 1387*w*, 1288*s*, 1224*m*, 1160*w*, 1014*w*, 824*s*, 756*m*, 718*w*, 490*w*.

5-Azido-2-(4-bromophenyl)pyridine (3): synthesized according to **GP2** from **S3** (192.0 mg, 400.0 μmol) run for 1 h at 40 °C.

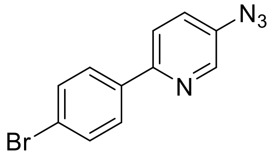

Yield: 59% (65.0 mg). Crystalline light brown solid. R_f 0.70 (pentane/EtOAc 9/1); **^{1}H NMR** (400 MHz, $CDCl_3$) δ 8.41 (d, *J* = 2.8 Hz, 1H), 7.83 (dd, *J* = 8.8, 2.0 Hz, 2H), 7.69 (dd, *J* = 8.8, 2.0 Hz, 1H), 7.61 – 7.55 (m, 2H), 7.41 (dd, *J* = 8.4, 2.8 Hz, 1H). **^{13}C NMR** (101 MHz, $CDCl_3$) δ 152.9, 141.2, 137.4, 136.1, 132.1, 128.3, 126.9, 123.6, 120.8. **HRMS** (ESI): calcd. for $[C_{11}H_8BrN_4]^+$, $[M+H]^+$: 274.9927; found: 274.9927. **IR** (neat, cm^{-1}) = 2103*s*, 1470*m*, 1406*w*, 1314*m*, 1287*m*, 1152*w*, 817*s*, 750*m*. **Mp:** 91.3 – 92.3 °C.

<u>4-(5-Azidopyridin-2-yl)benzaldehyde (4):</u> synthesized according to **GP2** from **S4** (171.0 mg, 400.0 μmol) run for 1 h at 40 ^{o}C.

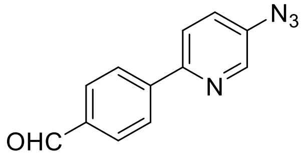

Yield: 51% (46.0 mg). Light brown solid. R_f 0.70 (pentane/EtOAc 4/1); **^{1}H NMR** (400 MHz, $CDCl_3$) δ 10.06 (s, 1H), 8.45 (dd, *J* = 2.8, 0.8 Hz, 1H), 8.14 (d, *J* = 8.4 Hz, 2H), 7.97 (d, *J* = 8.4 Hz, 2H), 7.80 (dd, *J* = 8.8, 0.8 Hz, 1H), 7.45 (dd, *J* = 8.8, 2.8 Hz, 1H). **^{13}C NMR** (101 MHz, $CDCl_3$) δ 192.0, 152.4, 144.0, 141.5, 136.8, 136.5, 130.3, 127.2, 126.8, 121.7. **HRMS** (ESI): calcd. for $[C_{12}H_8N_4ONa]^+$, $[M+Na]^+$: 247.0590; found: 247.0591. **IR** (neat, cm^{-1}) = 3017*w*, 2867*w*, 2429*w*, 2100*s*, 1684*s*, 1604*m*, 1575*m*, 1469*s*, 1391*m*, 1296*s*, 1227*m*, 1211*s*, 1169*s*, 1010*m*, 849*m*, 816*s*, 751*m*, 675*w*, 643*w*. **Mp:** 93.5 – 94.5 °C.

<u>3-Azido-2-(2,3-dihydrobenzo[*b*][1,4]dioxin-6-yl)pyridine and 5-azido-2-(2,3-dihydrobenzo[*b*][1,4]dioxin-6-yl)pyridine (5):</u> synthesized according to **GP2** from **S5** (400.0 μmol) run for 1 h at 40 ^{o}C.

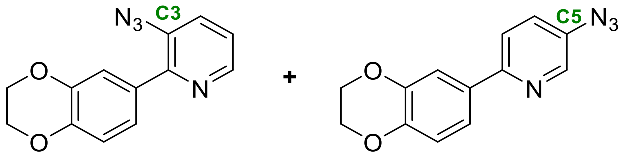

Yield: 50% (51.0 mg). Dark brown sticky oil. R_f 0.30 (pentane/EtOAc 9/1); **^{1}H NMR** (300 MHz, $CDCl_3$, containing regio-isomers: C3/C5 = 1:1) δ 8.36 – 8.28 (m, 1H), 7.62 – 7.34 (m, 3H), 7.26 – 7.20 (m, 1H), 6.93 (d, *J* = 8.4 Hz, 1H), 4.28 (s, 4H). **^{13}C NMR** (101 MHz, $CDCl_3$, containing regio-isomers: C3/C5 = 1:1) δ 153.6, 152.3, 144.7, 144.5, 143.9, 143.2, 140.9, z139.0, 136.1, 135.1, 132.2, 130.8, 130.3, 127.9, 126.8, 122.9, 120.4, 120.0, 118.7, 117.7, 117.1, 115.8, 64.6(9), 64.6(8), 64.4(9), 64.4(6). **HRMS** (ESI): calcd. for $[C_{13}H_{10}N_4O_2Na]^+$, $[M+Na]^+$: 277.0696; found: 277.0695.

IR (neat, cm^{-1}) = 2929*w*, 2933*w*, 2876*w*, 2108*s*, 1584*m*, 1508*m*, 1472*s*, 1444*s*, 1385*m*, 1281*s*, 1247*m*, 1126*m*, 1065*s*, 896*m*, 878*m*, 816*m*, 747*m*, 720*w*, 530*w*.

5-Azido-2-(2,4-difluorophenyl)pyridine (6): synthesized according to **GP2** from **S6** (175.0 mg, 400.0 μmol) run for 1 h at 40 °C.

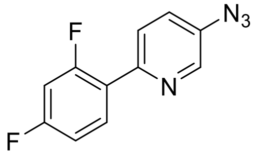

Yield: 43% (40.0 mg). White powder. R_f 0.70 (pentane/EtOAc 9/1); **^{1}H NMR** (300 MHz, $CDCl_3$) δ 8.43 (d, *J* = 2.7 Hz, 1H), 8.06 – 7.93 (m, 1H), 7.77 (dd, *J* = 8.4, 2.1 Hz, 1H), 7.41 (dd, *J* = 8.4, 2.7 Hz, 1H), 7.04 – 6.96 (m, 1H), 6.91 (ddd, *J* = 11.4, 8.7, 2.7 Hz, 1H). **^{13}C NMR** (101 MHz, $CDCl_3$) δ 163.3 (dd, $^1J_{C–F}$ = 251.3 Hz, $^3J_{C–F}$ = 12.1 Hz), 160.7 (dd, $^1J_{C–F}$ = 252.4, $^3J_{C–F}$ = 11.9 Hz), 149.1 (d, $^4J_{C–F}$ = 2.7 Hz), 141.3, 136.0, 132.0 (dd, $^3J_{C–F}$ = 9.6 Hz, $^4J_{C–F}$ = 4.4 Hz), 126.5, 124.7 (d, $^3J_{C–F}$ = 10.4 Hz), 123.0 (dd, $^3J_{C–F}$ = 11.6 Hz, $^4J_{C–F}$ = 3.9 Hz), 112.1 (dd, $^2J_{C–F}$ = 21.1 Hz, $^4J_{C–F}$ = 3.7 Hz), 104.6 (dd, $^2J_{C–F}$ = 27.1, 25.4 Hz). **^{19}F NMR** (282 MHz, $CDCl_3$) δ -109.0 (d, *J* = 8.6 Hz), -112.7 (d, *J* = 8.6 Hz). **HRMS** (ESI): calcd. for $[C_{11}H_7N_4F_2]^+$, $[M+H]^+$: 233.0633; found: 233.0633. **IR** (neat, cm^{-1}) = 2133*s*, 2107*s*, 1597*m*, 1508*m*, 1481*s*, 1428*m*, 1297*s*, 1265*m*, 1100*m*, 968*m*, 842*s*, 809*s*, 754*s*. **Mp:** 86.5 – 87.5 °C.

3-Azido-4-phenylpyridine (7): synthesized according to **GP2** from **S7** (160.0 mg, 400.0 μmol) run for 1 h at 40 °C.

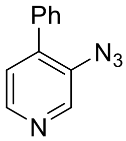

Yield: 56% (44.0 mg). Orange oil. R_f 0.60 (pentane/EtOAc 9/1); **^{1}H NMR** (400 MHz, $CDCl_3$) δ 8.60 (s, 1H), 8.45 (d, *J* = 4.8 Hz, 1H), 7.58 – 7.41 (m, 5H), 7.28 (d, *J* = 4.8 Hz, 1H). **^{13}C NMR** (101 MHz, $CDCl_3$) δ 144.7, 140.1, 139.1, 134.0, 132.6, 127.7, 127.5, 127.2, 123.4. **HRMS** (ESI): calcd. for $[C_{11}H_9N_4]^+$, $[M+H]^+$: 197.0822; found: 197.0822. **IR** (neat, cm^{-1}) = 3033*br*, 2100*s*, 1585m, 1504*w*, 1476*m*, 1409*m*, 1308*s*, 1297*s*, 1262*m*, 1183*w*, 1041*w*, 835*m*, 773*m*, 737*m*, 696*s*, 672*m*, 617*m*, 582*w*, 549*w*..

For 5.0 mmol scale: **S7** (2.0 g, 5.0 mmol), **A** (3.60 g, 12.5 mmol), TEMPONa in THF (9.0 mL, 7.5 mmol), *t*BuOMe (50 mL), 40 °C, 1h. 3-Azido-4-phenylpyridine (**6**) was obtioned with 712 mg, 73%.

3-Azido-4-(3-(trimethylsilyl)phenyl)pyridine (8): synthesized according to **GP2** from **S8** (189.0 mg, 400.0 μmol) run for 1 h at 40 °C.

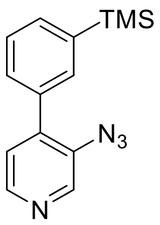

Yield: 54% (58.0 mg). Yellow oil. R_f 0.30 (pentane/EtOAc 8/1); **^{1}H NMR** (400 MHz, $CDCl_3$) δ 8.59 (d, *J* = 0.8 Hz, 1H), 8.44 (d, *J* = 4.8 Hz, 1H), 7.71 – 7.54 (m, 2H), 7.52 – 7.39 (m, 2H), 7.27 (dd, *J* = 4.8, 0.8 Hz, 1H), 0.31 (s, 9H). **^{13}C NMR** (101 MHz, $CDCl_3$) δ 146.2, 141.6, 141.3, 141.0, 134.9, 134.2, 134.0, 133.8, 129.5, 127.9, 125.0, -1.0. **HRMS** (ESI): calcd. for $[C_{14}H_{17}N_4Si]^+$, $[M+H]^+$: 269.1217; found: 269.1217. **IR** (neat, cm^{-1}) = 2954*w*, 2110*s*, 1584*w*, 1492*w*, 1413*w*, 1388*w*, 1307*m*, 1249*m*, 1119*w*, 1052*w*, 858*s*, 838*s*, 796*w*, 748*m*, 694*w*, 622*w*.

3-Azido-4-(5-isopropoxy-2-methyl-4-nitrophenyl)pyridine (9): synthesized according to **GP2** from **S9** (207.0 mg, 400.0 μmol) run for 1 h at 40 °C.

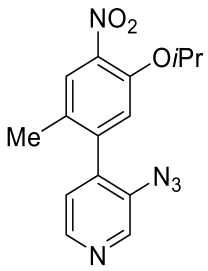

Yield: 36% (45.0 mg). Dark brown solid. R_f 0.20 (pentane/EtOAc 3/1); **^{1}H NMR** (400 MHz, $CDCl_3$) δ 8.62 (s, 1H), 8.48 (d, *J* = 4.8 Hz, 1H), 7.91 – 7.61 (m, 1H), 7.11 (dd, *J* = 4.8, 0.8 Hz, 1H), 6.81 (s, 1H), 4.60 (p, *J* = 6.1 Hz, 1H), 2.10 (s, 3H), 1.38 (d, *J* = 6.1 Hz, 6H). **^{13}C NMR** (101 MHz, $CDCl_3$) δ 149.1, 146.1, 141.3, 140.8(4), 140.7(9), 139.0, 134.9, 128.4, 126.8, 124.7, 117.4, 73.2, 22.0, 18.9. **HRMS** (ESI): calcd. for $[C_{15}H_{16}N_5O_3]^+$, $[M+H]^+$: 314.1248; found: 314.1247. **IR** (neat, cm^{-1}) = 2979*w*, 2929*w*, 2127*s*, 2107*s*, 1617*w*, 1570*w*, 1519*s*, 1482*w*, 1348*m*, 1308*s*, 1274*m*, 1223*m*, 1106*m*, 981*w*, 930*m*, 840*m*, 782*m*, 764*m*, 679*w*, 649*w*. **Mp:** 108.3 – 109.2 °C.

3'-Azido-2,4'-bipyridine (10): synthesized according to **GP2** from **S10** (160.0 mg, 400.0 μmol) run for 1 h at 40 °C.

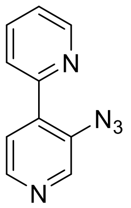

Yield: 53% (42.0 mg). Brown liquid. R_f 0.20 (pentane/EtOAc 2/1); **^{1}H NMR** (400 MHz, $CDCl_3$) δ 8.74 (dd, *J* = 4.8, 2.0 Hz, 1H), 8.61 (d, *J* = 2.0 Hz, 1H), 8.48 (dd, *J* = 5.2, 2.0 Hz, 1H), 7.80 (q, *J* = 2.4 Hz, 2H), 7.68 (dd, *J* = 5.2, 2.0 Hz, 1H), 7.33 (ddt, *J* = 7.2, 4.8, 2.4 Hz, 1H). **^{13}C NMR** (101

MHz, $CDCl_3$) δ 153.1, 150.1, 146.3, 141.8, 138.5, 136.4, 133.9, 125.0, 124.7, 123.6. **HRMS** (ESI): calcd. for $[C_{10}H_8N_5]^+$, $[M+H]^+$: 198.0774; found: 198.0771. **IR** (neat, cm^{-1}) = 2104*s*, 1582*m*, 1571*m*, 1496*w*, 1462*m*, 1433*m*, 1411*m*, 1319*m*, 1289*s*, 1218*w*, 1049*w*, 842*w*, 791*m*, 746*s*, 675*w*, 616*w*.

5-(3-Azidopyridin-4-yl)-2-methylbenzo[*d*]thiazole (11): synthesized according to **GP2** from **S11** (188.0 mg, 400.0 μmol) run for 1 h at 40 °C.

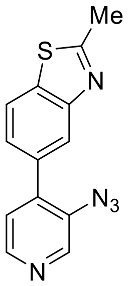

Yield: 42% (45.0 mg). Brown oil. R_f 0.20 (pentane/EtOAc 1/1); **^{1}H NMR** (300 MHz, $CDCl_3$) δ 8.62 (s, 1H), 8.47 (d, *J* = 4.8 Hz, 1H), 8.09 (d, *J* = 1.8 Hz, 1H), 7.92 (d, *J* = 8.4 Hz, 1H), 7.49 (dd, *J* = 8.4, 1.8 Hz, 1H), 7.34 (d, *J* = 4.8 Hz, 1H), 2.88 (s, 3H). **^{13}C NMR** (101 MHz, $CDCl_3$) δ 168.4, 153.7, 146.3, 141.7, 140.1, 136.5, 134.3, 133.5, 125.6, 125.0, 123.1, 121.6, 20.4. **HRMS** (ESI): calcd. for $[C_{13}H_{10}N_5S]^+$, $[M+H]^+$: 268.0651; found: 268.0651. **IR** (neat, cm^{-1}) = 3010*w*, 2350*s*, 2310*s*, 2115*br*, 1747*w*, 1508*s*, 1276*s*, 946*m*, 750*s*, 618*w*.

3-Azido-4-(benzo[*b*]thiophen-2-yl)pyridine (12): synthesized according to **GP2** from **S12** (182.0 mg, 400.0 μmol) run for 1 h at 40 °C.

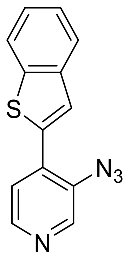

Yield: 30% (30.0 mg). Light brown solid. R_f 0.70 (pentane/EtOAc 1/1); **^{1}H NMR** (400 MHz, $CDCl_3$) δ 8.62 (s, 1H), 8.41 (d, *J* = 5.2 Hz, 1H), 7.98 (d, *J* = 0.8 Hz, 1H), 7.90 – 7.75 (m, 2H), 7.55 (d, *J* = 5.2 Hz, 1H), 7.45 – 7.36 (m, 2H). **^{13}C NMR** (101 MHz, $CDCl_3$) δ 146.1, 142.0, 140.4, 139.8, 136.4, 133.0, 132.5, 126.2, 125.7, 124.9, 124.5, 123.1, 122.2. **HRMS** (ESI): calcd. for $[C_{13}H_9N_4S]^+$, $[M+H]^+$: 253.0542; found: 253.0543. **IR** (neat, cm^{-1}) = 3056*w*, 2922*w*, 2126*s*, 2103*s*, 1581*s*, 1542*w*, 1483*w*, 1415*m*, 1308*s*, 1234*w*, 1174*w*, 1062*w*, 822*m*, 747*s*, 724*m*, 706*w*. **Mp:** Decomposed at 112.7 °C.

3-Azido-4-(3-phenylpropyl)pyridine (13): synthesized according to **GP2** from **S13** (177.0 mg, 400.0 μmol) run for 1 h at 40 °C.

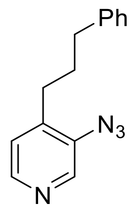

Yield: 61% (58.0 mg). Brown oil. R_f 0.50 (pentane/EtOAc 4/1); **^{1}H NMR** (400 MHz, $CDCl_3$) δ 8.44 (d, *J* = 1.6 Hz, 1H), 8.28 (dd, *J* = 4.8, 1.6 Hz, 1H), 7.30 (t, *J* = 7.2 Hz, 2H), 7.24 – 7.13 (m, 3H), 7.07 (dd, *J* = 4.8, 1.2 Hz, 1H), 2.67 (t, *J* = 7.6 Hz, 2H), 2.61 (t, *J* = 8.2, 7.6 Hz, 2H), 2.01 – 1.82 (m, 2H). **^{13}C NMR** (101 MHz, $CDCl_3$) δ 145.9, 142.1, 141.6, 140.4, 135.5, 128.5, 128.5, 126.1, 124.6, 35.6, 30.7, 30.2. **HRMS** (ESI): calcd. for $[C_{14}H_{15}N_4]^+$, $[M+H]^+$: 239.1291; found: 239.1290. **IR** (neat, cm^{-1}) = 3026*w*, 2930*w*, 2113*s*, 1588*m*, 1493*m*, 1411*m*, 1297*s*, 827*m*, 744*s*, 698*s*, 672*w*, 608*w*.

3-Azido-4-(3-(benzyloxy)propyl)pyridine (14): synthesized according to **GP2** from **S14** (189.0 mg, 400.0 μmol) run for 1 h at 40 °C.

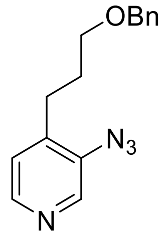

Yield: 56% (60.0 mg). Brown oil. R_f 0.20 (pentane/EtOAc 4/1); **^{1}H NMR** (400 MHz, $CDCl_3$) δ 8.43 (s, 1H), 8.26 (d, *J* = 4.8 Hz, 1H), 7.40 – 7.26 (m, 5H), 7.06 (d, *J* = 4.8 Hz, 1H), 4.51 (s, 2H), 3.48 (t, *J* = 6.4 Hz, 2H), 2.68 (t, *J* = 7.6 Hz, 2H), 1.99 – 1.77 (m, 2H). **^{13}C NMR** (101 MHz, $CDCl_3$) δ 145.9, 141.9, 140.4, 138.5, 135.6, 128.6, 127.9, 127.8, 124.9, 73.1, 69.3, 29.2, 27.5. **HRMS** (ESI): calcd. for $[C_{15}H_{17}N_4O]^+$, $[M+H]^+$: 269.1397; found: 269.1397. **IR** (neat, cm^{-1}) = 2926*w*, 2856*w*, 2117*s*, 1588*w*, 1492*m*, 1411*m*, 1293*s*, 1100*s*, 1076*s*, 825*m*, 735*s*, 697*s*, 672*w*, 601*w*.

3'-Azido-2,4'-bipyridine (15): synthesized according to **GP2** from **S15** (177.0 mg, 400.0 μmol) run for 1 h at 40 °C.

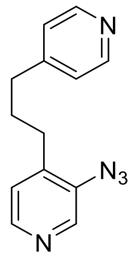

Yield: 37% (35.0 mg). Brown oil. R_f 0.20 (pentane/EtOAc 1/2); **^{1}H NMR** (300 MHz, $CDCl_3$) δ 8.53 – 8.47 (m, 2H), 8.44 (s, 1H), 8.28 (d, *J* = 4.8 Hz, 1H), 7.15 – 7.08 (m, 2H), 7.06 (d, *J* = 4.8 Hz, 1H), 2.62 (dt, *J* = 15.3, 7.8 Hz, 4H), 2.17 – 1.73 (m, 2H). **^{13}C NMR** (101 MHz, $CDCl_3$) δ 150.5,

149.9, 146.0, 141.4, 140.5, 135.6, 124.6, 123.9, 34.8, 30.2, 29.6. **HRMS** (ESI): calcd. for $[C_{13}H_{13}N_5Na]^+$, $[M+Na]^+$: 262.1063; found: 262.1063. **IR** (neat, cm^{-1}) = 2927*w*, 2115*s*, 1601*m*, 1589*m*, 1492*m*, 1291*s*, 825*m*, 792*m*, 754*w*, 672*w*, 609*w*.

3-Azido-4-benzylpyridine (16): synthesized according to **GP2** from **S16** (165.0 mg, 400.0 μmol) run for 1 h at 40 °C.

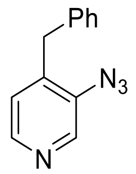

Yield: 43% (36.0 mg). Brown oil. R_f 0.30 (pentane/EtOAc 9/1); **^{1}H NMR** (400 MHz, $CDCl_3$) δ 8.40 (s, 1H), 8.20 (d, *J* = 4.8 Hz, 1H), 7.31 – 7.21 (m, 2H), 7.20 – 7.13 (m, 1H), 7.12 – 7.07 (m, 2H), 6.93 (d, *J* = 4.8 Hz, 1H), 3.84 (s, 2H). **^{13}C NMR** (101 MHz, $CDCl_3$) δ 145.7, 140.5, 140.1, 137.8, 135.2, 128.7, 128.5, 126.5, 124.7, 36.0. **HRMS** (ESI): calcd. for $[C_{12}H_{11}N_4]^+$, $[M+H]^+$: 211.0978; found: 211.0938. **IR** (neat, cm^{-1}) = 3029*w*, 2116*s*, 1584*m*, 1492*m*, 1401*m*, 1300*s*, 1049*w*, 840*m*, 735*m*, 696*s*, 672*w*, 621*m*, 566*w*.

5-Azido-3-methyl-2-phenylpyridine (17): synthesized according to **GP2** from **S17** (165.0 mg, 400.0 μmol) run for 1 h at 40 °C.

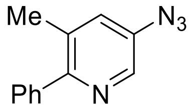

Yield: 49% (41.0 mg). Brown oil. R_f 0.60 (pentane/EtOAc 9/1); **^{1}H NMR** (400 MHz, $CDCl_3$) δ 8.17 (d, *J* = 2.4 Hz, 1H), 7.42 – 7.37 (m, 2H), 7.36 – 7.31 (m, 2H), 7.31 – 7.26 (m, 1H), 7.14 (d, *J* = 2.4 Hz, 1H), 2.26 (s, 3H). **^{13}C NMR** (101 MHz, $CDCl_3$) δ 155.3, 139.6, 138.2, 135.3, 131.9, 128.9, 128.1, 128.0(3), 127.9(7), 20.1. **HRMS** (ESI): calcd. for $[C_{12}H_{10}N_4Na]^+$, $[M+Na]^+$: 233.0798; found: 233.0797. **IR** (neat, cm^{-1}) = 2310*m*, 2114*s*, 1462*m*, 1308*m*, 1017*m*, 749*s*, 698*m*.

5-Azido-3-methoxy-2-phenylpyridine (18): synthesized according to **GP2** from **S18** (172.0 mg, 400.0 μmol) run for 1 h at 40 °C.

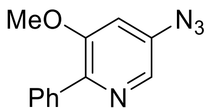

Yield: 22% (20.0 mg). Pale yellow oil. R_f 0.70 (pentane/EtOAc 9/1); **^{1}H NMR** (400 MHz, $CDCl_3$) δ 8.11 (d, *J* = 2.2 Hz, 1H), 7.96 – 7.78 (m, 2H), 7.51 – 7.40 (m, 2H), 7.40 – 7.33 (m, 1H), 6.91 (d, *J* = 2.2 Hz, 1H), 3.88 (s, 3H). **^{13}C NMR** (101 MHz, $CDCl_3$) δ 154.2, 145.0, 137.1, 136.5, 132.3, 129.3, 128.5, 128.2, 109.2, 77.5, 77.2, 76.8, 55.8. **HRMS** (ESI): calcd. for $[C_{12}H_{11}N_4O]^+$, $[M+H]^+$: 227.0927; found: 227.0927. **IR** (neat, cm^{-1}) = 2923*m*, 2360*s*, 2341*s*, 2113*m*, 1684*w*, 1541*w*, 1457*w*, 742*w*, 669*w*.

5-Azido-2-phenyl-3-(trifluoromethyl)pyridine (19): synthesized according to **GP2** from **S19** (374.0 mg, 800.0 µmol) run for 1 h at 40 °C.

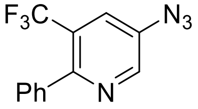

Yield: 26% (55.0 mg). Brown oil. R_f 0.80 (pentane/EtOAc 9/1); **^{1}H NMR** (400 MHz, $CDCl_3$) δ 8.58 (d, J = 2.5 Hz, 1H), 7.70 (d, J = 2.6 Hz, 1H), 7.56 – 7.38 (m, 5H). **^{13}C NMR** (101 MHz, $CDCl_3$) 154.5, 143.0, 138.3, 135.9, 129.0, 128.7, 128.1, 127.1, 125.7, 125.4, 124.8, 124.7(6), 124.7, 124.6, 124.3, 121.6. **^{19}F NMR** (282 MHz, $CDCl_3$) δ -57.7. **HRMS** (ESI): calcd. for $[C_{12}H_{18}N_4F_3]^+$, $[M+H]^+$: 265.0696; found: 265.0697. **IR** (neat, cm^{-1}) = 2360*m*, 2341*m*, 2117*s*, 1456*m*, 1324*m*, 1165*m*, 1135*m*, 753*m*, 699*w*.

5-Azido-3-fluoro-2-phenylpyridine (20): synthesized according to **GP2** from **S20** (167.0 mg, 400.0 µmol) run for 1 h at 40 °C.

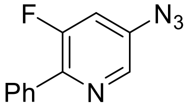

Yield: 48% (41.0 mg). Pale yellow oil. R_f 0.70 (pentane/EtOAc 9/1); **^{1}H NMR** (400 MHz, $CDCl_3$) δ 8.39 – 8.29 (m, 1H), 7.95 (dd, J = 6.8, 1.6 Hz, 2H), 7.56 – 7.37 (m, 3H), 7.17 (dd, J = 11.2, 2.2 Hz, 1H). **^{13}C NMR** (101 MHz, $CDCl_3$) δ 157.5 (d, $^1J_{C–F}$ = 264.1 Hz), 142.7 (d, $^2J_{C–F}$ = 10.9 Hz), 141.4, 137.0 (d, $^3J_{C–F}$ = 4.4 Hz), 136.8 (d, $^3J_{C–F}$ = 4.6 Hz), 134.8 (d, $^3J_{C–F}$ = 5.8 Hz), 132.7, 129.4, 128.7, 128.6, 114.6 (d, $^2J_{C–F}$ = 23.9 Hz). **^{19}F NMR** (282 MHz, $CDCl_3$) δ -120.8. **HRMS** (ESI): calcd. for $[C_{11}H_8N_4F]^+$, $[M+H]^+$: 215.0728; found: 215.0727. **IR** (neat, cm^{-1}) = 2113*s*, 1602*w*, 1463*m*, 1446*m*, 1412*m*, 1320*m*, 1230*m*, 1172*w*, 1118*w*, 782*w*, 730*m*, 693*m*.

5-Azido-3-chloro-2-phenylpyridine (21): synthesized according to **GP2** from **S21** (174.0 mg, 400.0 µmol) run for 1 h at 40 °C.

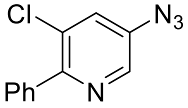

Yield: 51% (47.0 mg). Brown oil. R_f 0.80 (pentane/EtOAc 9/1); **^{1}H NMR** (400 MHz, $CDCl_3$) δ 8.35 (d, J = 2.4 Hz, 1H), 7.72 (dd, J = 8.0, 2.0 Hz, 2H), 7.56 – 7.39 (m, 4H). **^{13}C NMR** (101 MHz, $CDCl_3$) δ 152.9, 139.0, 137.4, 136.4, 130.4, 129.3, 128.9, 128.1, 127.8. **HRMS** (ESI): calcd. for $[C_{11}H_8N_4Cl]^+$, $[M+H]^+$: 231.0432; found: 231.0432. **IR** (neat, cm^{-1}) = 2111*s*, 1578*m*, 1442*s*, 1388*m*, 1281*m*, 1204*w*, 1082*w*, 888*m*, 735*m*, 696*s*.

5-Azido-4-methyl-2-phenylpyridine (22): synthesized according to **GP2** from **S22** (166.0 mg, 400.0 µmol) run for 1 h at 40 °C.

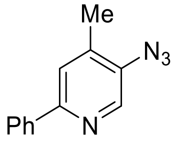

Yield: 42% (35.0 mg). Light brown oil. R_f 0.50 (pentane/EtOAc 20/1); **^{1}H NMR** (300 MHz, $CDCl_3$) δ 8.48 (s, 1H), 8.07 – 7.88 (m, 2H), 7.53 (s, 1H), 7.50 – 7.33 (m, 3H), 2.28 (s, 3H). **^{13}C NMR** (101 MHz, $CDCl_3$) δ 153.8, 140.0, 138.8(2), 138.7(8), 134.7, 128.9(5), 128.8(9), 126.8, 122.5, 17.2. **HRMS** (ESI): calcd. for $[C_{12}H_{10}N_4Na]^+$, $[M+Na]^+$: 233.0798; found: 233.0799. **IR** (neat, cm^{-1}) = 2924*w*, 2122*s*, 2089*s*, 1478*m*, 1303*m*, 1272*w*, 777*w*, 735*w*, 693*m*.

<u>3-Azido-5-methyl-2-phenylpyridine (23):</u> synthesized according to **GP2** from **S23** (165.0 mg, 400.0 μmol) run for 1 h at 40 °C.

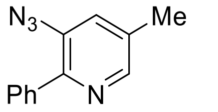

Yield: 45% (38.0 mg). Brown oil. R_f 0.40 (pentane/EtOAc 9/1); **^{1}H NMR** (400 MHz, $CDCl_3$) δ 8.32 (dd, J = 2.0, 0.8 Hz, 1H), 7.83 – 7.73 (m, 2H), 7.48 – 7.43 (m, 2H), 7.43 – 7.38 (m, 1H), 7.36 (dd, J = 2.0, 0.8 Hz, 1H), 2.42 (s, 3H). **^{13}C NMR** (101 MHz, $CDCl_3$) δ 147.8, 146.5, 137.3, 133.8, 133.3, 129.3, 128.7, 128.2, 127.1, 18.1. **HRMS** (ESI): calcd. for $[C_{12}H_{10}N_4Na]^+$, $[M+Na]^+$: 233.0798; found: 233.0797. **IR** (neat, cm^{-1}) = 2107*s*, 2063*w*, 1456*m*, 1397*m*, 1300*w*, 1241*w*, 1217*w*, 739*m*, 695*m*.

<u>3-Azido-2-(4-(*tert*-butyl)phenyl)-5-methylpyridine (24):</u> synthesized according to **GP2** from **S24** (188.0 mg, 400.0 μmol) run for 1 h at 40 °C.

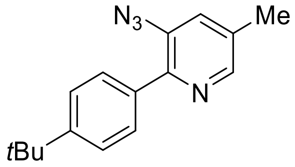

Yield: 43% (46.0 mg). Yellow oil. R_f 0.70 (pentane/EtOAc 9/1); **^{1}H NMR** (400 MHz, $CDCl_3$) δ 8.33 (d, J = 0.8 Hz, 1H), 7.75 (d, J = 8.4 Hz, 2H), 7.50 (d, J = 8.4 Hz, 2H), 7.37 (d, J = 0.8 Hz, 1H), 2.43 (s, 3H), 1.38 (s, 9H). **^{13}C NMR** (101 MHz, $CDCl_3$) δ 151.4, 147.4, 146.1, 134.1, 133.2, 132.6, 128.7, 126.6, 124.8, 34.4, 31.1, 17.8. **HRMS** (ESI): calcd. for $[C_{16}H_{18}N_4Na]^+$, $[M+Na]^+$: 289.1424; found: 289.1420. **IR** (neat, cm^{-1}) = 2962*m*, 2108*s*, 1457*m*, 1393*w*, 1300*m*, 1218*w*, 1013*w*, 840*w*, 768*m*..

<u>3-Azido-2-(4-chlorophenyl)-5-methylpyridine (25):</u> synthesized according to **GP2** from **S25** (179.0 mg, 400.0 μmol) run for 1 h at 40 °C.

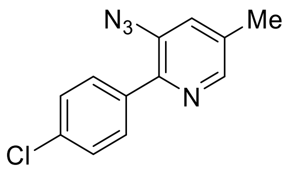

Yield: 41% (40.0 mg). White solid. R_f 0.30 (pentane/EtOAc 9/1); **^{1}H NMR** (400 MHz, $CDCl_3$) δ 8.31 (d, *J* = 0.8 Hz, 1H), 7.76 (d, *J* = 8.4 Hz, 2H), 7.42 (d, *J* = 8.4 Hz, 2H), 7.36 (d, *J* = 0.8 Hz, 1H), 2.42 (s, 3H). **^{13}C NMR** (101 MHz, $CDCl_3$) δ 146.5, 146.3, 135.7, 134.7, 133.7, 133.6, 130.7, 128.3, 127.1, 18.1. **HRMS** (ESI): calcd. for $[C_{12}H_9N_4ClNa]^+$, $[M+Na]^+$: 267.0408; found: 267.0408. **IR** (neat, cm^{-1}) = 2113*s*, 1461*m*, 1392*w*, 1295*m*, 1220*w*, 762*m*. **Mp:** 100.2 – 101.2 °C.

3-Azido-2-(4-methoxyphenyl)-5-methylpyridine (26): synthesized according to **GP2** from **S26** (178.0 mg, 400.0 μmol) run for 1 h at 40 °C.

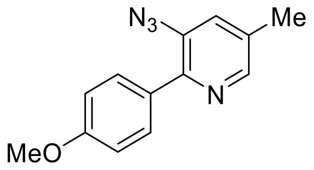

Yield: 43% (41.0 mg). Yellow solid. R_f 0.10 (pentane/EtOAc 9/1); **^{1}H NMR** (400 MHz, $CDCl_3$) δ 8.30 (d, *J* = 0.8 Hz, 1H), 7.77 (d, *J* = 8.8 Hz, 2H), 7.34 (d, *J* = 0.8 Hz, 1H), 6.99 (d, *J* = 8.8 Hz, 2H), 3.86 (s, 3H), 2.41 (s, 3H). **^{13}C NMR** (101 MHz, $CDCl_3$) δ 159.9, 147.3, 146.3, 133.2, 132.5, 130.6, 129.7, 126.9, 113.5, 55.3, 17.9. **HRMS** (ESI): calcd. for $[C_{13}H_{13}N_4O]^+$, $[M+H]^+$: 241.1084; found: 241.1078. **IR** (neat, cm^{-1}) = 2932*br*, 2103*s*, 1606*m*, 1511*m*, 1455*s*, 1395*m*, 1294*s*, 1245*s*, 1117*s*, 1109*m*, 1023*m*, 835*s*, 767*m*, 634*w*. **Mp:** 73.9 – 74.7 °C.

3-Azido-2-(3-chlorophenyl)-5-methylpyridine (27): synthesized according to **GP2** from **S27** (179.0 mg, 400.0 μmol) run for 1 h at 40 °C.

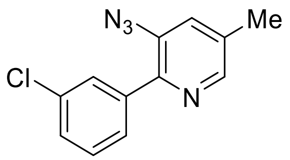

Yield: 38% (37.0 mg). White solid. R_f 0.30 (pentane/EtOAc 9/1); **^{1}H NMR** (400 MHz, $CDCl_3$) δ 8.31 (d, *J* = 0.8 Hz, 1H), 7.81 (d, *J* = 1.2 Hz, 1H), 7.74 – 7.65 (m, 1H), 7.42 – 7.30 (m, 3H), 2.43 (s, 8H). **^{13}C NMR** (101 MHz, $CDCl_3$) δ 146.6, 146.1, 139.1, 134.2, 134.0(0), 133.9(8), 129.5, 129.4, 128.7, 127.6, 127.2, 18.2. **HRMS** (ESI): calcd. for $[C_{12}H_{10}N_4Cl]^+$, $[M+H]^+$: 245.0589; found: 245.0583. **IR** (neat, cm^{-1}) = 2113*s*, 1594*w*, 1452*m*, 1395*w*, 1308*m*, 751*s*, 689*w*. **Mp:** Decomposed at 172.3 °C.

3-Azido-5-methoxy-2-phenylpyridine (28): synthesized according to **GP2** from **S28** (172.0 mg, 400.0 μmol) run for 1 h at 40 °C.

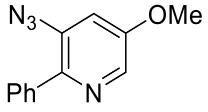

Yield: 51% (46.0 mg). White solid. R_f 0.30 (pentane/EtOAc 4/1); **^{1}H NMR** (300 MHz, $CDCl_3$) δ 8.21 (d, *J* = 2.4 Hz, 1H), 7.82 – 7.66 (m, 2H), 7.57 – 7.33 (m, 3H), 7.05 (d, *J* = 2.4 Hz, 1H), 3.93 (s, 3H). **^{13}C NMR** (101 MHz, $CDCl_3$) δ 155.6, 143.2, 137.2, 134.6, 133.6, 129.2, 128.4, 128.2, 111.6, 56.1. **HRMS** (ESI): calcd. for $[C_{12}H_{11}N_4O]^+$, $[M+H]^+$: 227.0927; found: 227.0923. **IR** (neat, cm^{-1}) = 2938*w*, 2109*s*, 1588*m*, 1464*m*, 1435*m*, 1307*m*, 1244*s*, 1208*s*, 1045*m*, 867*w*, 735*m*, 696*m*, 560*w*. **Mp:** 68.1 – 68.7 °C.

3-Azido-4-(4-fluorophenyl)pyridine (29): synthesized according to **GP2** from **S29** (167.0 mg, 400.0 μmol) run for 1 h at 40 °C.

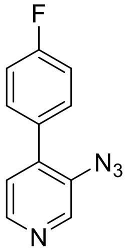

Yield: 47% (40.0 mg). White powder. R_f 0.30 (pentane/EtOAc 4/1); **^{1}H NMR** (300 MHz, $CDCl_3$) δ 8.61 (s, 1H), 8.46 (d, *J* = 4.8 Hz, 1H), 7.61 – 7.42 (m, 2H), 7.33 – 7.23 (m, 1H), 7.23 – 7.13 (m, 2H). **^{13}C NMR** (101 MHz, $CDCl_3$) δ 163.1 (d, $^1J_{C–F}$ = 249.4 Hz), 146.2, 141.6, 139.4, 134.0, 131.4 (d, $^4J_{C–F}$ = 3.4 Hz), 131.0 (d, $^3J_{C–F}$ = 8.3 Hz), 124.7, 115.7 (d, $^2J_{C–F}$ = 21.7 Hz). **^{19}F NMR** (282 MHz, $CDCl_3$) δ -112.2. **HRMS** (ESI): calcd. for $[C_{11}H_8N_4F]^+$, $[M+H]^+$: 215.0728; found: 215.0727. **IR** (neat, cm^{-1}) = 3041*w*, 2120*s*, 1591*m*, 1516*m*, 1485*s*, 1316*m*, 1296*s*, 1223*s*, 1159*m*, 822*s*, 813*s*, 755*w*, 729*m*, 571*m*, 548*m*. **Mp:** 79.8 – 80.8 °C.

***N*-(3-(5-Azidopyridin-2-yl)-4-chlorophenyl)-2-chloro-4-(methylsulfonyl)benzamide (30):** synthesized according to **GP2** from **S30** (200.0 mg, 300.0 μmol) run for 1 h at 40 °C.

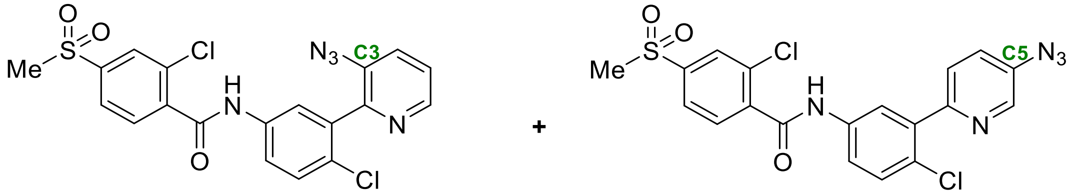

Yield: 47% (65.0 mg). Colorless film. R_f 0.40 (pentane/EtOAc 1/2); **^{1}H NMR** (400 MHz, $CDCl_3$, containing regio-isomers: C3/C5 = 1:2) δ 8.79 – 8.56 (s, 1H), 8.26 (t, *J* = 1.9 Hz, 1H), 7.92 (s, 1H), 7.85 – 7.60 (m, 4H), 7.57 – 7.35 (m, 2H), 3.06 (s, 3H). **^{13}C NMR** (101 MHz, $CDCl_3$, containing regio-isomers: C3/C5 = 1:2) δ 163.4, 163.2, 152.3, 151.2, 143.2, 143.1, 140.7, 140.4, 140.3, 138.8, 138.5, 137.9, 137.5, 136.5(9), 136.5(7), 136.3, 132.3(8), 132.3(6), 132.3, 131.1, 131.0, 130.8, 130.4, 129.4, 129.3, 129.2, 128.0, 127.2, 126.1(1), 126.0(8), 126.0(5), 125.8, 123.0, 122.3, 122.0,

121.8, 44.5(4), 44.5(1). **HRMS** (ESI): calcd. for $[C_{19}H_{13}N_5O_3SCl_2Na]^+$, $[M+Na]^+$: 484.0008; found: 484.0007. **IR** (neat, cm^{-1}) = 2925*w*, 2115*s*, 1669*s*, 1583*m*, 1536*s*, 1454*s*, 1376*m*, 1302*s*, 1152*s*, 1097*m*, 960*m*, 887*m*, 804*m*, 733*s*, 701*m*, 596*w*, 553*m*.

5-Azido-*N*,*N*-diethylnicotinamide (31): synthesized according to **GP2** from **S31** (169.0 mg, 400.0 μmol) run for 1 h at 40 °C.

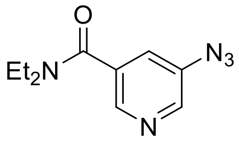

Yield: 29% (25.0 mg). Brown oil. R_f 0.10 (pentane/EtOAc 4/1); **^{1}H NMR** (400 MHz, $CDCl_3$) δ 8.39 (d, *J* = 1.6 Hz, 1H), 8.38 (d, *J* = 2.8 Hz, 1H), 7.39 (dd, *J* = 2.8, 1.6 Hz, 1H), 3.62 – 3.28 (m, 2H), 3.26 – 3.17 (m, 2H), 1.30 – 1.20 (m, 3H), 1.20 – 1.07 (m, 3H). **^{13}C NMR** (101 MHz, $CDCl_3$) δ 167.6, 143.2, 141.9, 137.5, 133.8, 124.4, 43.6, 39.8, 14.5, 13.0. **HRMS** (ESI): calcd. for $[C_{10}H_{13}N_5O]^+$, $[M+Na]^+$: 242.1012; found: 242.1006. **IR** (neat, cm^{-1}) = 2974*w*, 2934*w*, 2109*s*, 1630*s*, 1460*w*, 1423*m*, 1318*m*, 1300*m*, 1278*m*, 1220*w*, 1100*w*, 889*w*, 834*w*, 753*m*, 705*w*, 665*w*.

Ethyl 4-(3-azido-8-chloro-5,6-dihydro-11*H*-benzo[5,6]cyclohepta[1,2-*b*]pyridin-11-ylidene)piperidine-1-carboxylate (32): synthesized according to **GP2** from **S32** (188.0 mg, 300.0 μmol) run for 1 h at 40 °C.

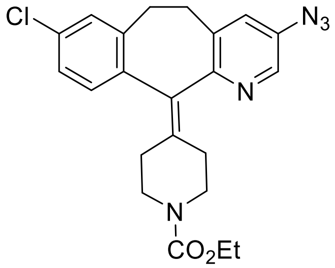

Yield: 36% (46.0 mg). Yellow foam. R_f 0.40 (pentane/EtOAc 4/1); **^{1}H NMR** (400 MHz, $CDCl_3$) δ 8.14 (d, *J* = 2.4 Hz, 1H), 7.20 – 7.11 (m, 2H), 7.12 – 7.05 (m, 2H), 4.13 (q, *J* = 7.2 Hz, 2H), 3.88 – 3.66 (m, 2H), 3.43 – 3.29 (m, 2H), 3.14 (ddd, *J* = 13.2, 8.8, 4.4 Hz, 2H), 2.92 – 2.73 (m, 2H), 2.47 (ddd, *J* = 14.1, 9.3, 4.6 Hz, 1H), 2.41 – 2.22 (m, 3H), 1.24 (t, *J* = 7.2 Hz, 3H). **^{13}C NMR** (101 MHz, $CDCl_3$) δ 155.6, 153.6, 139.5, 138.4, 138.1, 137.8, 135.6, 134.6, 133.4, 133.2, 130.6, 129.0, 127.4, 126.5, 61.5, 44.9, 44.9, 31.8, 31.5, 30.9, 30.7, 14.8. **HRMS** (ESI): calcd. for $[C_{22}H_{22}N_5O_2ClNa]^+$, $[M+Na]^+$: 446.1354; found: 446.1349. **IR** (neat, cm^{-1}) = 2950*w*, 2111*s*, 1696*s*, 1442*s*, 1304*w*, 1278*w*, 1228*m*, 1117*w*, 997*w*.

For 1.0 mmol scale: S32 (627 mg, 1.00 mmol), **A** (723 mg, 2.50 mmol), TEMPONa in THF (1.8 mL, 1.5 mmol), *t*BuOMe (10 mL), 40 °C, 1h. Ethyl 4-(3-azido-8-chloro-5,6-dihydro-11*H*-benzo[5,6]cyclohepta[1,2-*b*]pyridin-11-ylidene)piperidine-1-carboxylate (**32**) was obtained with 201 mg, 47%.

***N*-((3-azidopyridin-4-yl)methyl)-*N*-ethyl-3-hydroxy-2-phenylpropanamide (33):** synthesized according to the **GP2** from **S33** (159.0 mg, 300.0 μmol) run for 1 h at 40 °C.

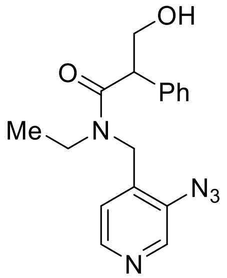

Yield: 27% (26.0 mg). Pale yellow film. R_f 0.30 (EtOAc); **^{1}H NMR** (300 MHz, $CDCl_3$, containing rotamers and isomers) δ 8.88 – 8.09 (m, 2H), 7.43 – 7.24 (m, 4H), 7.27 – 7.11 (m, 1H), 7.04 – 7.82 (m, 1H), 4.84 – 4.10 (m, 2H), 4.17 – 3.92 (m, 1H), 3.87 – 3.53 (m, 2H), 3.41 – 2.60 (m, 3H), 1.17 – 0.79 (m, 3H). **^{13}C NMR** (101 MHz, $CDCl_3$, containing rotamers and isomers) δ 173.6, 172.9(9), 172.9(5), 168.1, 150.7, 146.4, 146.2, 140.3, 140.2, 137.4, 137.3, 137.0, 136.4, 136.2, 135.8, 135.0, 134.6(7), 129.3(0), 129.2(7), 129.0, 128.6, 128.5, 128.4, 128.2, 128.1, 128.0, 127.9(7), 127.9, 127.6, 122.5, 122.3, 121.4, 87.6, 77.5, 77.2, 76.8, 67.6, 66.0, 66.0(1), 65.3, 54.4, 52.2, 52.1, 48.5, 45.5, 43.4, 42.7, 41.6, 39.1, 34.6, 29.8, 14.8, 13.6, 12.6, 12.5. **HRMS** (ESI): calcd. for $[C_{17}H_{19}N_5O_2Na]^+$, $[M+Na]^+$: 348.1431; found: 348.1431. **IR** (neat, cm^{-1}) = 2928*w*, 2360*s*, 2341*s*, 2124*s*, 1635*s*, 1456*s*, 1415*s*, 1311*s*, 1281*s*, 1051*s*, 751*m*, 701*s*.

General procedure for ring enlargement (GP3):

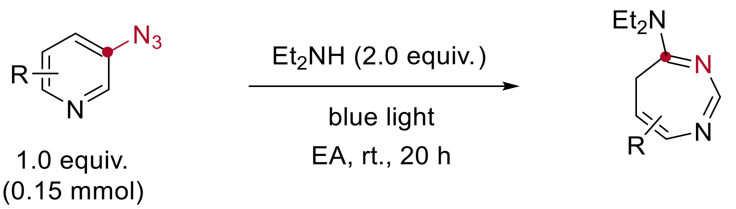

To an oven-dried 5 mL Schlenk tube, pyridinyl azide (0.15 mmol, 1.0 equiv.) obtained with **GP2** was dissolved in 3.0 mL dry ethyl acetate, and diethyl amine (0.3 mmol, 2.0 equiv.) was added under Ar atmosphere. The vial was then sealed and put under vacuum, followed by flushing with Ar gas 3 times. The tube was placed in a photoreactor, stirred and irradiated for 20 h. The temperature was maintained below 30 °C using a fan. After the completion of the reaction, as monitored by TLC, the solvent was removed under reduced pressure. The residue was purified by column chromatography (EtOAc/MeOH) on silica gel, to give the desired product. The concentrated product was then dissolved in DCM then filtered with Nylon membrane filter (0.2 μm). DCM was removed under reduced pressure to give pure product.

<u>*N*,*N*-diethyl-7-phenyl-5*H*-1,3-diazepin-4-amine (34):</u> synthesized according to **GP3** from **1** (30.0 mg, 0.150 mmol).

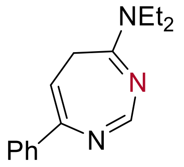

Yield: 54% (19.0 mg). Brown solid. ***R*f** 0.20 (EtOAc/MeOH 4/1); **^{1}H NMR** (500 MHz, CD_2Cl_2, -50 °C) δ 7.94 (s, 1H), 7.74 – 7.48 (m, 2H), 7.32 (dd, *J* = 7.1, 1.2 Hz, 2H), 7.29 – 7.18 (m, 1H), 5.44 (t, *J* = 7.4 Hz, 1H), 3.69 (dd, *J* = 12.6, 8.6 Hz, 1H), 3.43 – 3.11 (m, 4H), 1.93 (dd, *J* = 12.6, 6.3 Hz, 1H), 1.15 (t, *J* = 7.1 Hz, 3H), 1.03 (t, *J* = 7.1 Hz, 3H). **^{13}C NMR** (126 MHz, CD_2Cl_2, -60 °C) δ 153.2, 152.2, 147.9, 138.6, 127.9, 127.4, 126.0, 102.6, 43.5, 43.1, 31.0, 13.2, 11.7. **HRMS** (ESI): calcd. for $[C_{15}H_{20}N_3]^+$, $[M+H]^+$: 242.1652; found: 242.1644. **Mp**: 106.7 – 107.7 °C. **IR** (neat, cm^{-1}) = 2976*m*, 1593*w*, 1560*s*, 1498*s*, 1470*s*, 1360*m*, 1241*m*, 1148*m*, 1078*w*, 872*w*, 766*s*, 696*m*.

<u>7-(4-Bromophenyl)-*N*,*N*-diethyl-5*H*-1,3-diazepin-4-amine (35):</u> synthesized according to **GP3** from **3** (41.0 mg, 0.150 mmol).

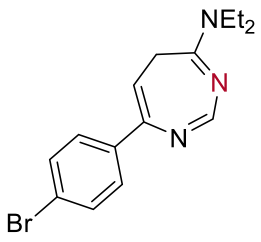

Yield: 56% (27.0 mg). Brown thick oil. ***R*f** 0.15 (EtOAc/MeOH 4/1); **^{1}H NMR** (500 MHz, $CDCl_3$, -50 °C) δ 8.07 (s, 1H), 7.51 (d, *J* = 8.3 Hz, 2H), 7.44 (d, *J* = 8.3 Hz, 2H), 5.41 (t, *J* = 7.4 Hz, 1H), 3.74 (dd, *J* = 12.6, 8.6 Hz, 1H), 3.43 – 3.30 (m, 4H), 1.99 (dd, *J* = 12.6, 6.3 Hz, 1H), 1.22 (t, *J* = 7.1 Hz, 3H), 1.09 (t, *J* = 7.1 Hz, 3H). **^{13}C NMR** (126 MHz, $CDCl_3$, -50 °C) δ 153.6, 152.4, 147.3, 137.3, 131.2, 128.2, 121.9, 102.7, 44.0, 43.4, 31.4, 13.8, 12.3. **HRMS** (ESI): calcd. for $[C_{15}H_{19}N_3Br]^+$, $[M+H]^+$: 320.0757; found: 320.0754. **IR** (neat, cm^{-1}) = 2979*w*, 1594*w*, 1564*s*, 1499*s*, 1276*m*, 1260*m*, 1149*w*, 1010*w*, 810*w*, 764*s*, 750*s*, 688*w*.

4-(4-(Diethylamino)-5*H*-1,3-diazepin-7-yl)benzaldehyde (36): synthesized according to **GP3** from **4** (34.0 mg, 0.150 mmol).

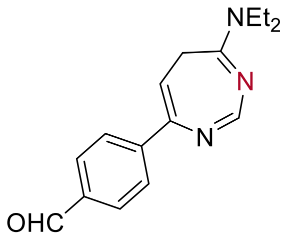

Yield: 60% (24.0 mg). Brown thick oil. ***R*f** 0.20 (EtOAc/MeOH 4/1); **^{1}H NMR** (500 MHz, $CDCl_3$, -40 °C) δ 9.98 (s, 1H), 8.08 (s, 1H), 7.84 (q, *J* = 8.4 Hz, 4H), 5.57 (t, *J* = 7.4 Hz, 1H), 3.85 – 3.68 (m, 1H), 3.52 – 3.23 (m, 4H), 2.41 – 1.92 (m, 1H), 1.24 (t, *J* = 7.2 Hz, 3H), 1.10 (t, *J* = 7.2 Hz, 3H). **^{13}C NMR** (126 MHz, $CDCl_3$, -50 °C) δ 193.0, 153.9, 152.3, 147.5, 144.8, 134.9, 130.1, 127.0, 105.0, 44.0, 43.4, 31.5, 13.8, 12.3. **HRMS** (ESI): calcd. for $[C_{16}H_{20}N_3O]^+$, $[M+H]^+$: 270.1601; found: 270.1600. **IR** (neat, cm^{-1}) = 2988*w*, 1705*s*, 1566*s*, 1508*m*, 1276*s*, 1260*s*, 750*s*, 650*w*.

***N*,*N*-diethyl-6-phenyl-4*H*-1,3-diazepin-7-amine (37):** synthesized according to **GP3** from **7** (30.0 mg, 0.150 mmol).

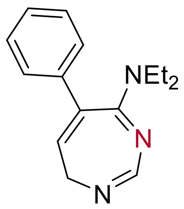

Yield: 58% (21.0 mg). Dark brown solid. ***R*f** 0.10 (EtOAc/MeOH 4/1); **^{1}H NMR** (400 MHz, $CDCl_3$) δ 7.37 (s, 1H), 7.21 – 7.02 (m, 5H), 7.02 – 6.95 (m, 1H), 5.25 (d, *J* = 9.2 Hz, 1H), 5.19 (ddd, *J* = 9.2, 6.8, 1.0 Hz, 1H), 3.74 – 3.35 (m, 4H), 1.27 (t, *J* = 7.2 Hz, 3H), 1.17 (t, *J* = 7.2 Hz, 3H). **^{13}C NMR** (101 MHz, $CDCl_3$) δ 153.7, 151.5, 141.0, 135.0, 128.1, 126.9, 126.9, 108.2, 46.6, 44.9, 44.2, 14.0, 12.4. **HRMS** (ESI): calcd. for $[C_{15}H_{20}N_3]^+$, $[M+H]^+$: 242.1652; found: 242.1643. **Mp**: 73.0 – 74.0 °C. **IR** (neat, cm^{-1}) = 2976*w*, 1597*w*, 1554*s*, 1448*m*, 1359*m*, 1251*m*, 1146*w*, 744*s*, 696*w*.

***N*,*N*-diethyl-6-(4-fluorophenyl)-4*H*-1,3-diazepin-7-amine (38):** synthesized according to **GP3** from **6** (32.0 mg, 0.150 mmol).

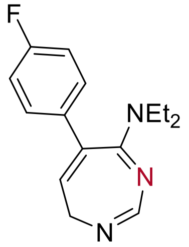

Yield: 51% (20.0 mg). Brown solid. ***R*f** 0.10 (EtOAc/MeOH 4/1); **^{1}H NMR** (500 MHz, $CDCl_3$) δ 7.41 (s, 1H), 7.08 – 6.96 (m, 3H), 6.86 (t, *J* = 8.6 Hz, 2H), 5.25 (d, *J* = 5.2 Hz, 2H), 3.77 – 3.33 (m, 4H), 1.30 (t, *J* = 7.2 Hz, 3H), 1.17 (t, *J* = 7.0 Hz, 3H). **^{13}C{^{19}F} NMR** (126 MHz, $CDCl_3$) δ 161.7, 153.9, 150.7, 139.6, 130.1, 128.4, 115.0, 108.2, 46.1, 45.2, 44.4, 13.8, 12.3. **^{19}F NMR** (470 MHz, $CDCl_3$) δ -115.5. **HRMS** (ESI): calcd. for $[C_{15}H_{19}N_3F]^+$, $[M+H]^+$: 260.1558; found: 260.1557. **Mp**: 52.8 – 53.6 °C. **IR** (neat, cm^{-1}) = 2977*w*, 2933*w*, 1658*w*, 1556*s*, 1507*s*, 1456*s*, 1382*m*, 1360*m*, 1223*s*, 1159*m*, 1079*w*, 819*m*, 791*w*, 750*m*.

***N*,*N*-diethyl-6-(5-isopropoxy-2-methyl-4-nitrophenyl)-4*H*-1,3-diazepin-7-amine (39):** synthesized according to **GP3** from **9** (31.0 mg, 0.100 mmol).

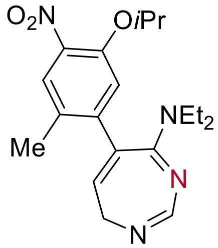

Yield: 22% (8.0 mg). Brown solid. ***R*f** 0.10 (EtOAc/MeOH 4/1); **^{1}H NMR** (400 MHz, $CDCl_3$) δ 7.53 (s, 1H), 7.42 (s, 1H), 7.15 (d, *J* = 7.0 Hz, 1H), 6.68 (s, 1H), 5.27 (d, *J* = 9.2 Hz, 1H), 5.19 (dd, *J* = 9.2, 6.8 Hz, 1H), 4.52 (hept, *J* = 6.0 Hz, 1H), 3.86 (dd, *J* = 14.8, 7.2 Hz, 1H), 3.55 (dd, *J* = 13.6, 7.0 Hz, 1H), 3.43 (t, *J* = 9.0 Hz, 2H), 2.33 (s, 3H), 1.33 (s, 2H), 1.30 (d, *J* = 6.0 Hz, 3H), 1.22 (dt, *J* = 22.4, 7.2 Hz, 12H). **^{13}C NMR** (101 MHz, $CDCl_3$) δ 150.3, 149.6, 149.3, 140.1, 139.3, 137.2, 128.1, 127.7, 116.6, 106.7, 73.2, 46.9, 45.1, 44.4, 22.2, 22.1, 19.5, 12.5, 11.9. **HRMS** (ESI): calcd. for $[C_{19}H_{26}N_4O_3Na]^+$, $[M+Na]^+$: 381.1897; found: 381.1892. **Mp**: 63.2 – 64.2 °C. **IR** (neat, cm^{-1}) = 2979*m*, 1552*s*, 1518*s*, 1489*s*, 1457*s*, 1355*s*, 1276*s*, 1256*s*, 1105*m*, 765*s*, 750*s*.

***N*,*N*-diethyl-6-(3-(trimethylsilyl)phenyl)-4*H*-1,3-diazepin-7-amine (40):** synthesized according to **GP3** from **8** (40.0 mg, 0.150 mmol).

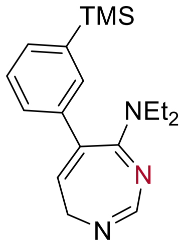

Yield: 23% (11.0 mg). Brown sticky oil. ***R*f** 0.10 (EtOAc/MeOH 4/1); **^{1}H NMR** (400 MHz, $CDCl_3$) δ 7.42 (s, 1H), 7.30 (dd, *J* = 7.2, 1.2 Hz, 1H), 7.22 (dd, *J* = 2.0, 1.0 Hz, 1H), 7.17 (t, *J* = 7.5 Hz, 1H), 7.10 – 7.03 (m, 1H), 7.00 (dd, *J* = 6.2, 1.6 Hz, 1H), 5.33 – 5.16 (m, 2H), 3.67 – 3.40 (m, 4H), 1.35 – 1.13 (m, 6H), 0.21 (s, 9H). **^{13}C NMR** (101 MHz, $CDCl_3$) δ 154.1, 151.4, 140.3, 140.3, 134.1, 132.0, 131.7, 127.5, 127.4, 108.5, 46.8, 45.1, 44.4, 14.0, 12.4, -1.1. **HRMS** (ESI): calcd. for $[C_{18}H_{27}N_3SiNa]^+$, $[M+Na]^+$: 336.1867; found: 336.1858. **IR** (neat, cm^{-1}) = 2954*m*, 1670*m*, 1596*s*, 1555*s*, 1491*m*, 1360*m*, 1248*s*, 837*s*, 751*s*, 691*w*.

N,N-diethyl-6-methyl-7-phenyl-5H-1,3-diazepin-4-amine (41): synthesized according to **GP3** from **17** (32.0 mg, 0.150 mmol).

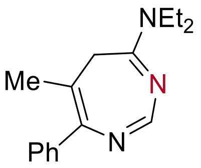

Yield: 65% (25.0 mg). Brown sticky oil. ***R*f** 0.20 (EtOAc/MeOH 4/1); **^{1}H NMR** (500 MHz, $CDCl_3$, -50 °C) δ 7.91 (s, 1H), 7.39 – 7.23 (m, 5H), 3.69 (ddd, *J* = 14.6, 11.6, 6.9 Hz, 2H), 3.59 (d, *J* = 12.4 Hz, 1H), 3.23 (ddt, *J* = 16.9, 14.0, 7.1 Hz, 2H), 2.10 (d, *J* = 12.3 Hz, 1H), 1.92 (s, 3H), 1.28 (t, *J* = 7.1 Hz, 3H), 1.08 (t, *J* = 7.1 Hz, 3H). **^{13}C NMR** (126 MHz, $CDCl_3$, -50 °C) δ 152.7, 151.7, 144.1, 139.4, 129.1, 127.9, 127.1, 113.3, 43.7, 43.4, 37.4, 21.4, 15.7, 12.4. **HRMS** (ESI): calcd. for $[C_{16}H_{22}N_3]^+$, $[M+H]^+$: 256.1808; found: 256.1809. **IR** (neat, cm^{-1}) = 2978*m*, 1670*s*, 1564*s*, 1447*s*, 1361*s*, 1259*m*, 751*s*, 700*s*.

N,N-diethyl-6-methoxy-7-phenyl-5H-1,3-diazepin-4-amine (42): synthesized according to **GP3** from **18** (10.0 mg, 0.044 mmol).

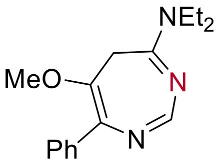

Yield: 67% (8.0 mg). Pale yellow film. ***R*f** 0.20 (EtOAc/MeOH 4/1); **^{1}H NMR** (500 MHz, $CDCl_3$, -50 °C) δ 7.99 (s, 1H), 7.55 (d, *J* = 7.7 Hz, 2H), 7.38 (t, *J* = 7.5 Hz, 2H), 7.29 (t, *J* = 7.3 Hz, 1H), 4.06 (d, *J* = 13.1 Hz, 1H), 3.64 (d, *J* = 1.4 Hz, 3H), 3.54 – 3.29 (m, 4H), 1.88 (d, *J* = 13.0 Hz, 1H), 1.28 (t, *J* = 7.2 Hz, 3H), 1.12 (t, *J* = 7.0 Hz, 3H). **^{13}C NMR** (101 MHz, $CDCl_3$, rt, containing resonance isomers) δ 150.1, 150.0, 137.2, 132.9, 132.7, 129.9, 129.1, 128.1, 127.5, 59.1, 44.1, 43.7, 35.0, 32.1, 29.9, 29.7, 29.5, 22.8, 15.3, 14.3, 12.4. **HRMS** (ESI): calcd. for $[C_{16}H_{22}N_3O]^+$, $[M+H]^+$: 272.1757; found: 272.1755. **IR** (neat, cm^{-1}) = 2927*w*, 2360*s*, 2341*s*, 1558*m*, 1507*m*, 1457*w*, 913*w*, 747*m*, 669*w*.

6-Chloro-*N*,*N*-diethyl-7-phenyl-2*H*-1,4-diazepin-3-amine (43) and 6-Chloro-*N*,*N*-diethyl-7-phenyl-5*H*-1,3-diazepin-4-amine (44): synthesized according to **GP3** from **21** (35.0 mg, 0.150 mmol).

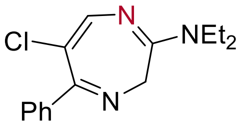

43: Yield: 46% (18.0 mg). brown solid. ***R*f** 0.90 (EtOAc/MeOH 4/1); **^{1}H NMR** (500 MHz, $CDCl_3$, -50 °C) δ 7.68 (s, 1H), 7.65 – 7.60 (m, 2H), 7.43 – 7.35 (m, 3H), 5.26 (d, *J* = 9.8 Hz, 1H), 3.72 – 3.61 (m, 2H), 3.34 – 3.14 (m, 2H), 3.15 (d, *J* = 9.8 Hz, 1H), 1.31 (t, *J* = 7.2 Hz, 3H), 1.09 (t, *J* = 7.0 Hz, 3H). **^{13}C NMR** (126 MHz, $CDCl_3$, -50 °C) δ 168.8, 149.9, 148.6, 138.2, 129.7, 129.7, 128.1, 113.6, 52.8, 43.8, 31.0, 15.1, 12.7. **HRMS** (ESI): calcd. for $[C_{15}H_{18}N_3ClNa]^+$, $[M+Na]^+$: 298.1082; found: 298.1081. **Mp**: 104.5 – 105.5 °C. **IR** (neat, cm^{-1}) = 2975*w*, 1585*m*, 1563*s*, 1492*w*, 1455*s*, 1373*m*, 1359*m*, 1249*m*, 1157*w*, 1081*m*, 1065*m*, 1033*s*, 872*w*, 774*m*, 698*s*, 658*w*.

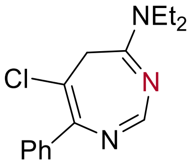

44: Yield: 17% (7.0 mg). Brown thick oil. ***R*f** 0.60 (EtOAc/MeOH 4/1); **^{1}H NMR** (500 MHz, $CDCl_3$, -50 °C) δ 7.97 (s, 1H), 7.53 – 7.47 (m, 2H), 7.43 – 7.35 (m, 2H), 7.35 – 7.29 (m, 1H), 3.98 (d, *J* = 13.6 Hz, 1H), 3.79 – 3.54 (m, 2H), 3.41 – 3.31 (m, 2H), 2.49 (d, *J* = 13.6 Hz, 1H), 1.32 (t, *J* = 7.2 Hz, 3H), 1.14 (t, *J* = 7.2 Hz, 3H). **^{13}C NMR** (126 MHz, $CDCl_3$, -50 °C) δ 152.2, 151.3, 145.8, 138.3, 129.2, 128.0(1), 127.9(7), 104.2, 44.0, 43.9, 40.7, 15.6, 12.4. **HRMS** (ESI): calcd. for $[C_{15}H_{19}N_3Cl]^+$, $[M+H]^+$: 276.1262; found: 276.1254. **IR** (neat, cm^{-1}) = 2983*br*, 1705*m*, 1680*s*, 1631*s*, 1583*s*, 1448*s*, 1361*m*, 766*s*, 750*s*, 700*s*.

***N*,*N*-diethyl-6-fluoro-7-phenyl-2*H*-1,4-diazepin-3-amine (45) and 5-fluoro-6-phenylpyridin-3-amine (46):** synthesized according to **GP3** from **20** (32.0 mg, 0.150 mmol).

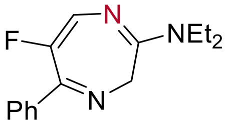

45: Yield: 31% (12.0 mg). brown film. ***R*f** 0.50 (EtOAc/MeOH 4/1); **^{1}H NMR** (500 MHz, $CDCl_3$, -50 °C) δ 8.03 (s, 1H), 7.67 (d, *J* = 7.6 Hz, 2H), 7.41 (t, *J* = 7.6 Hz, 2H), 7.31 (t, *J* = 7.4 Hz, 1H), 4.39 – 3.73 (m, 1H), 3.56 – 3.38 (m, 4H), 2.42 – 2.23 (m, 1H), 1.25 (t, *J* = 7.2 Hz, 3H), 1.16 (t, *J* = 7.0 Hz, 3H). **^{13}C{^{19}F} NMR** (126 MHz, $CDCl_3$, -50 °C) δ 151.6, 150.3, 135.8, 135.1, 129.1, 128.3(9), 128.3(6), 128.0, 44.3, 43.6, 34.4, 15.1, 12.4. **^{19}F NMR** (376 MHz, $CDCl_3$) δ -115.5. **HRMS** (ESI): calcd. for $[C_{15}H_{18}N_3FNa]^+$, $[M+Na]^+$: 282.1377; found: 282.1368. **IR** (neat, cm^{-1}) = 2979*w*, 1568*s*, 1508*s*, 1361*w*, 1250*w*, 749*s*, 697*m*.

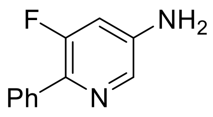

46: Yield: 28% (8.0 mg). brown film. ***R*f** 0.70 (EtOAc/MeOH 4/1); **^{1}H NMR** (400 MHz, $CDCl_3$) δ 8.01 (dd, *J* = 2.4, 1.6 Hz, 1H), 7.88 (dt, *J* = 8.2, 1.6 Hz, 2H), 7.49 – 7.40 (m, 2H), 7.37 – 7.29 (m, 1H), 6.78 (dd, *J* = 12.4, 2.4 Hz, 1H), 3.87 (s, 2H). **^{13}C NMR** (101 MHz, $CDCl_3$) δ 158.11 (d, $^{1}J_{C–F}$ = 259.5 Hz), 143.30 (d, $^{3}J_{C–F}$ = 6.1 Hz), 136.24 (d, $^{2}J_{C–F}$ = 11.5 Hz), 135.92 (d, $^{3}J_{C–F}$ = 5.7 Hz), 133.22 (d, $^{3}J_{C–F}$ = 3.8 Hz), 128.48, 128.15, 128.10, 109.51 (d, $^{2}J_{C–F}$ = 23.8 Hz). **^{19}F NMR** (376 MHz, $CDCl_3$) δ -123.5. **HRMS** (ESI): calcd. for $[C_{11}H_9N_2FNa]^+$, $[M+Na]^+$: 211.0642; found: 211.0635. **IR** (neat, cm^{-1}) = 3336*m*, 3210*m*, 1626*s*, 1606*s*, 1475*s*, 1426*s*, 1338*m*, 1258*m*, 1155*s*, 750*s*, 730*s*, 697*s*, 587*w*, 565*w*.

<u>*N*,*N*-diethyl-7-phenyl-6-(trifluoromethyl)-2*H*-1,4-diazepin-3-amine (47):</u> synthesized according to **GP3** from **19** (35.0 mg, 0.132 mmol).

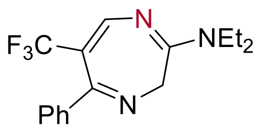

Yield: 51% (21.0 mg). White film. ***R*f** 0.20 (pentane/EtOAc 3/1); **^{1}H NMR** (500 MHz, $CDCl_3$) δ 7.91 (d, *J* = 1.2 Hz, 1H), 7.63 – 7.45 (m, 2H), 7.44 – 7.29 (m, 3H), 5.30 (d, *J* = 9.8 Hz, 1H), 3.83 – 3.60 (m, 2H), 3.35 – 3.21 (m, 2H), 3.08 (d, *J* = 9.9 Hz, 1H), 1.33 (t, *J* = 7.2 Hz, 3H), 1.09 (t, *J* = 7.2 Hz, 3H). **^{13}C{F} NMR** (126 MHz, $CDCl_3$) δ 168.0, 151.0(3), 150.9(9), 139.4, 129.5, 129.1, 128.0, 125.5, 112.5, 52.8, 44.0, 43.9, 15.0, 12.6. **^{19}F NMR** (376 MHz, $CDCl_3$) δ -53.1. **HRMS** (ESI): calcd. for $[C_{16}H_{18}N_3F_3Na]^+$, $[M+Na]^+$: 332.1345; found: 332.1345. **IR** (neat, cm^{-1}) = 2929*w*, 2360*m*, 2341*w*, 1570*s*, 1472*s*, 1287*s*, 1100*s*, 1065*s*, 1035*m*, 741*w*, 703*m*.

<u>7-(2,4-Difluorophenyl)-*N*,*N*-diethyl-5*H*-1,3-diazepin-4-amine (54):</u> synthesized according to **GP3** from **6** (35.0 mg, 0.150 mmol).

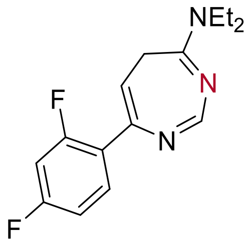

Yield: 58% (24.0 mg). Brown thick oil. ***R*f** 0.30 (EtOAc/MeOH 4/1); **^{1}H NMR** (500 MHz, $CDCl_3$, -50 °C) δ 8.03 (s, 1H), 7.66 – 7.46 (m, 1H), 6.87 (td, *J* = 8.3, 2.6 Hz, 1H), 6.83 – 6.76 (m, 1H), 5.45 (t, *J* = 7.5 Hz, 1H), 3.75 (dd, *J* = 12.6, 8.6 Hz, 1H), 3.49 – 3.19 (m, 4H), 2.00 (dd, *J* = 12.6, 6.3 Hz, 1H), 1.22 (t, *J* = 7.1 Hz, 3H), 1.10 (t, *J* = 7.1 Hz, 3H). **^{13}C{^{19}F} NMR** (126 MHz, $CDCl_3$, -50 °C) δ 161.8, 159.8, 153.8, 152.2, 142.7, 131.9, 122.8, 111.1, 107.1, 104.1, 43.9, 43.4, 31.6, 13.8, 12.2. **^{19}F NMR** (470 MHz, $CDCl_3$, -50 °C) δ -111.0, -111.2 (q, *J* = 9.5 Hz). **HRMS** (ESI): calcd. for

[$C_{15}H_{18}N_3F_2$]$^+$, [M+H]$^+$: 278.1463; found: 278.1456. **IR** (neat, cm^{-1}) = **IR** (neat, cm^{-1}) = 2979*m*, 1680*m*, 1594*s*, 1564*s*, 1500*s*, 1473*m*, 1423*m*, 1379*m*, 1264*s*, 1140*m*, 1099*s*, 967*m*, 849*m*, 750*s*.

Ethyl 4-(9-chloro-4-(diethylamino)-6,7-dihydrobenzo[5,6]cyclohepta[1,2-*d*][1,3]diazepin-12(5*H*)-ylidene)piperidine-1-carboxylate (55): synthesized according to **GP3** from **32** (64.0 mg, 0.150 mmol).

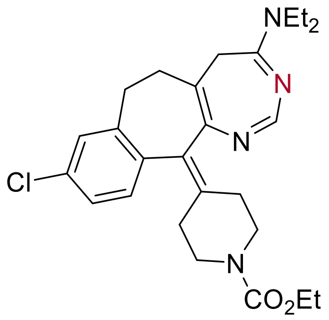

Yield: 53% (37.0 mg). Yellow foam. ***R*f** 0.30 (EtOAc/MeOH 4/1); **^{1}H NMR** (500 MHz, $CDCl_3$, -50 °C) δ 7.85 (s, 1H), 7.23 – 7.02 (m, 3H), 4.10 – 3.99 (m, 2H), 3.91 – 3.50 (m, 4H), 3.35 – 3.03 (m, 4H), 3.01 – 2.79 (m, 2H), 2.70 – 2.51 (m, 2H), 2.51 – 2.00 (m, 4H), 1.99 – 1.77 (m, 2H), 1.36 – 0.90 (m, 9H). **^{13}C NMR** (126 MHz, $CDCl_3$, -50 °C) δ 155.3, 154.1, 154.0, 152.0, 139.9, 133.1, 132.2, 132.0, 129.2, 128.2, 127.6, 126.2, 126.1, 61.5, 61.4, 44.4, 37.1, 35.0, 31.0, 30.8, 30.7, 30.5, 15.6, 14.7, 12.1, 11.9. **HRMS** (ESI): calcd. for [$C_{26}H_{34}N_4O_2Cl$]$^+$, [M+H]$^+$: 469.2365; found: 469.2359. **IR** (neat, cm^{-1}) = **IR** (neat, cm^{-1}) = 2977*w*, 1691*s*, 1558*s*, 1507*w*, 1473*s*, 1433*s*, 1356*w*, 1226*s*, 1116*m*, 992*w*, 766*s*.

General procedure for C–H insertion (GP4):

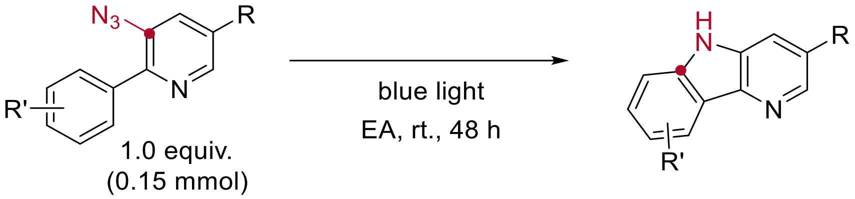

To an oven-dried 5 mL Schlenk tube, pyridinyl azide (0.15 mmol, 1.0 equiv.) obtained with **GP2** was dissolved in 3.0 mL dry ethyl acetate under Ar atmosphere. The tube was then placed in a photoreactor, stirred and irradiated for 48 h. The temperature was maintained below 30 °C using a fan. After the completion of the reaction, as monitored by TLC, the solvent was removed under reduced pressure. The residue was was purified by column chromatography (pentane/EtOAc) on silica gel to give the pure product.

Note: These carboline product shows poor solubility in DMSO. The IR spectrum could not be measured properly with our device here.

3-Methoxy-5*H*-pyrido[3,2-*b*]indole (48): synthesized according to **GP4** from **28** (34.0 mg, 0.150 mmol).

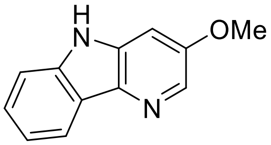

Yield: 77% (23.0 mg). Orange solid. ***R*f** 0.30 (pentane/EtOAc 2/1); **^{1}H NMR** (400 MHz, DMSO-*d*6) δ 11.30 (s, 1H), 8.18 (d, *J* = 2.4 Hz, 1H), 8.06 (dd, *J* = 7.8, 1.0 Hz, 1H), 7.51 (dt, *J* = 8.2, 1.0 Hz, 2H), 7.43 – 7.37 (m, 2H), 7.20 (td, *J* = 7.4, 1.0 Hz, 1H), 3.91 (s, 3H). **^{13}C NMR** (101 MHz, DMSO-*d*6) δ 154.2, 140.4, 134.9, 133.9, 131.7, 125.8, 121.8, 119.4, 119.1, 111.5, 101.2, 55.7. **HRMS** (ESI): calcd. for $[C_{12}H_{11}N_2O]^+$, $[M+H]^+$: 199.0866; found: 199.0866. **Mp**: 210.8 – 211.5 °C.

3-Methyl-5*H*-pyrido[3,2-*b*]indole (49): synthesized according to **GP4** from **23** (32.0 mg, 0.150 mmol).

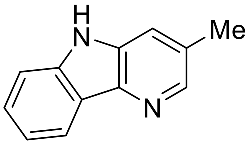

Yield: 73% (20.0 mg). Yellow solid. ***R*f** 0.10 (pentane/EtOAc 4/1); **^{1}H NMR** (400 MHz, DMSO-*d*6) δ 11.31 (s, 1H), 8.30 (d, *J* = 1.8 Hz, 1H), 8.13 (d, *J* = 7.8 Hz, 1H), 7.67 (d, *J* = 1.0 Hz, 1H), 7.52 (d, *J* = 8.2 Hz, 1H), 7.45 (ddd, *J* = 8.2, 6.8, 1.2 Hz, 1H), 7.21 (ddd, *J* = 7.8, 6.8, 1.0 Hz, 1H), 2.47 (s, 3H). **^{13}C NMR** (101 MHz, DMSO-*d*6) δ 142.2, 140.4, 139.2, 133.1, 129.6, 126.8, 121.6, 119.7,

119.2, 117.9, 111.6, 18.6. **HRMS** (ESI): calcd. for $[C_{12}H_{11}N_2]^+$, $[M+H]^+$: 183.0917; found: 183.0917. **Mp**: 252.4 – 253.4 °C.

7-(*Tert*-butyl)-3-methyl-5*H*-pyrido[3,2-*b*]indole (50): synthesized according to **GP4** from **24** (40.0 mg, 0.150 mmol).

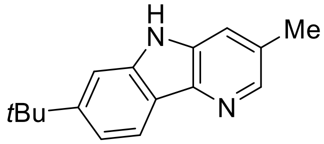

Yield: 63% (23.0 mg). Orange solid. ***R*f** 0.45 (pentane/EtOAc 2/1); **^{1}H NMR** (400 MHz, DMSO-*d*6) δ 11.13 (s, 1H), 8.26 (d, *J* = 1.8 Hz, 1H), 8.03 (d, *J* = 8.3 Hz, 1H), 7.63 (s, 1H), 7.46 (d, *J* = 1.6 Hz, 1H), 7.29 (dd, *J* = 8.3, 1.7 Hz, 1H), 2.46 (s, 3H), 1.38 (s, 9H). **^{13}C NMR** (101 MHz, DMSO-*d*6) δ 149.9, 141.9, 140.8, 139.2, 133.2, 129.0, 119.3, 117.6, 117.3, 107.7, 34.9, 31.5, 18.6 (one Ar-C signal is missing). **HRMS** (ESI): calcd. for $[C_{16}H_{18}N_2Na]^+$, $[M+Na]^+$: 261.1362; found: 261.1362. **Mp**: 204.1 – 205.1 °C.

7-Methoxy-3-methyl-5*H*-pyrido[3,2-*b*]indole (51): synthesized according to **GP4** from **26** (36.0 mg, 0.150 mmol).

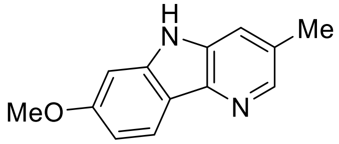

Yield: 69% (22.0 mg). Yellow solid. ***R*f** 0.20 (pentane/EtOAc 2/1); **^{1}H NMR** (400 MHz, DMSO-*d*6) δ 11.14 (s, 1H), 8.40 – 8.10 (m, 1H), 7.98 (d, *J* = 8.6 Hz, 1H), 7.59 (dd, *J* = 1.8, 1.0 Hz, 1H), 6.99 (d, *J* = 2.2 Hz, 1H), 6.82 (dd, *J* = 8.6, 2.2 Hz, 1H), 3.85 (s, 3H), 2.44 (s, 3H). **^{13}C NMR** (101 MHz, DMSO-*d*6) δ 159.5, 142.0, 141.7, 139.6, 133.0, 128.1, 120.6, 117.4, 115.4, 108.7, 94.8, 55.3, 18.5. **HRMS** (ESI): calcd. for $[C_{13}H_{13}N_2O]^+$, $[M+H]^+$: 213.1022; found: 213.1022. **Mp**: 215.3 – 216.2 °C.

7-Chloro-3-methyl-5H-pyrido[3,2-b]indole (52): synthesized according to **GP4** from **25** (37.0 mg, 0.150 mmol), 8 mg starting material was recovered after the reaction.

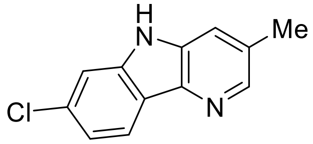

Yield: 55% (18.0 mg). Yellow solid. ***R*f** 0.30 (pentane/EtOAc 2/1); **^{1}H NMR** (400 MHz, DMSO-*d*6) δ 11.44 (s, 1H), 8.33 (d, *J* = 1.8 Hz, 1H), 8.11 (d, *J* = 8.2 Hz, 1H), 7.71 (d, *J* = 1.0 Hz, 1H), 7.58 (d, *J* = 1.8 Hz, 1H), 7.22 (dd, *J* = 8.2, 1.8 Hz, 1H), 2.47 (s, 3H). **^{13}C NMR** (101 MHz, DMSO-*d*6) δ 142.8, 140.9, 138.4, 133.5, 131.1, 130.1, 121.0, 120.5, 119.5, 118.3, 111.3, 18.6. **HRMS** (ESI): calcd. for $[C_{12}H_8N_2Cl]^+$, $[M-H]^+$: 215.0382; found: 215.0380. **Mp**: 303.7 – 304.4 °C.

8-chloro-3-methyl-5*H*-pyrido[3,2-*b*]indole and 6-chloro-3-methyl-5*H*-pyrido[3,2-*b*]indole (53): synthesized according to **GP4** from **27** (37.0 mg, 0.150 mmol).

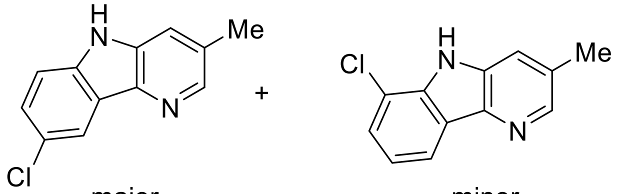

major minor

Yield: 55% (18.0 mg). Yellow solid. ***R*f** 0.40 (pentane/EtOAc 2/1); **^{1}H NMR** (400 MHz, DMSO-d_6, containing regio-isomers: major/minor 1.2:1) δ 11.64 (s, NH, minor), 11.48 (s, NH, major), 8.37 (d, *J* = 1.8 Hz, CH, minor), 8.34 (d, *J* = 1.8 Hz, CH, major), 8.13 – 8.05 (m, 1H), 7.72 (s, 1H), 7.57 (s, 1H, CH, minor), 7.55 (s, 1H, CH, major), 7.46 (dd, *J* = 8.6, 2.2 Hz, CH, major), 7.23 (t, *J* = 7.7 Hz, CH, major), 2.50 (s, CH_3, minor), 2.48 (s, CH_3, minor). **^{13}C NMR** (101 MHz, DMSO-d_6, containing regio-isomers: major/minor 1.2:1) δ 143.3, 142.9, 138.9, 138.7, 138.1, 137.1, 133.8, 133.5, 130.6, 130.5, 126.6, 126.1, 123.8, 123.6, 122.8, 120.3, 118.8, 118.6, 118.5, 118.3, 115.9, 113.2, 18.6(0), 18.5(9). **HRMS** (ESI): calcd. for $[C_{12}H_{10}N_2Cl]^+$, $[M+H]^+$: 217.0527; found: 217.0527.

Proposed mechanism for hydrolysis

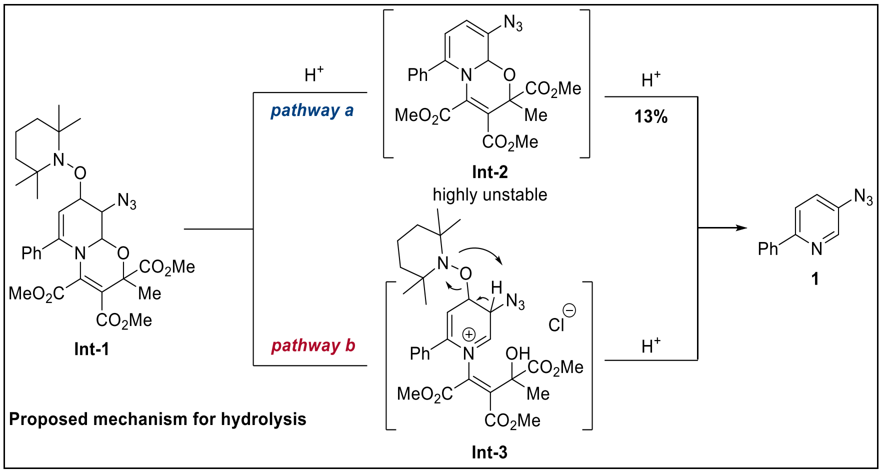

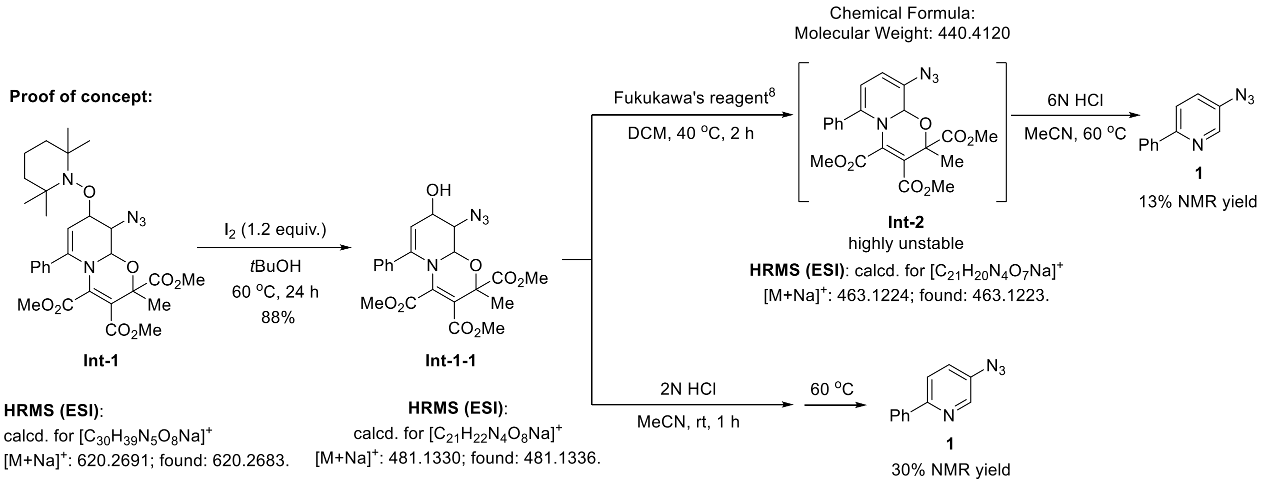

Figure S2. Proposed mechanism for hydrolysis step

<u>Trimethyl 9-azido-2-methyl-6-phenyl-8-((2,2,6,6-tetramethylpiperidin-1-yl)oxy)-9,9a-dihydro-2*H*,8*H*-pyrido[2,1-*b*][1,3]oxazine-2,3,4-tricarboxylate (Int-1):</u> synthesized according to **GP2** from **S1**.

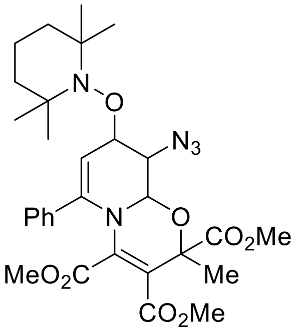

Yellow foam. ***R*f** 0.30 (pentane/EtOAc 4/1); **^{1}H NMR** (300 MHz, $CDCl_3$, containing diasteroisomers) δ 7.46 – 7.21 (m, 5H), 6.03 – 4.19 (m, 3H), 3.95 – 3.69 (m, 4H), 3.68 – 2.90 (m, 6H), 2.00 – 1.64 (m, 3H), 1.64 – 1.33 (m, 6H), 1.29 – 1.02 (m, 12H). **^{13}C NMR** (101 MHz, $CDCl_3$, containing diasteroisomers) δ 171.0, 170.2, 165.7, 165.5, 165.2, 165.2, 163.3, 163.1, 163.0, 142.1, 141.6, 141.1, 139.9, 139.6, 137.0, 136.6, 135.6, 135.5, 135.4(1), 135.3(6), 129.0, 128.9, 128.7, 128.6, 128.2, 128.1, 128.0, 127.9, 113.7, 113.5, 113.2, 111.5, 111.1, 109.4, 109.3, 83.6, 82.6, 80.7, 80.2, 79.0, 78.7(4), 78.7(1), 78.6, 78.0, 77.7, 77.4(9), 77.4(6), 77.2, 77.1, 76.9, 76.8, 73.6(2), 73.6(0), 65.1, 65.0, 61.8, 61.2, 60.9, 60.3, 59.4, 53.1, 53.0, 52.8, 52.7, 52.2, 52.1, 52.0, 51.9(5), 51.9, 51.8, 51.7, 40.5, 40.2, 24.8, 24.1, 22.3, 21.7, 17.1, 14.1. **HRMS** (ESI): calcd. for $[C_{30}H_{39}N_5O_8Na]^+$, $[M+Na]^+$: 620.2691; found: 620.2683. **IR** (neat, cm^{-1}) = 2948*m*, 2109*s*, 1742*s*, 1711*s*, 1591*s*, 1435*m*, 1375*m*, 1243*s*, 1203*s*, 1179*s*, 1125*s*, 1069*m*, 1012*m*, 750*s*, 700*m*.

Table S1. Condition optimization for hydrolysis

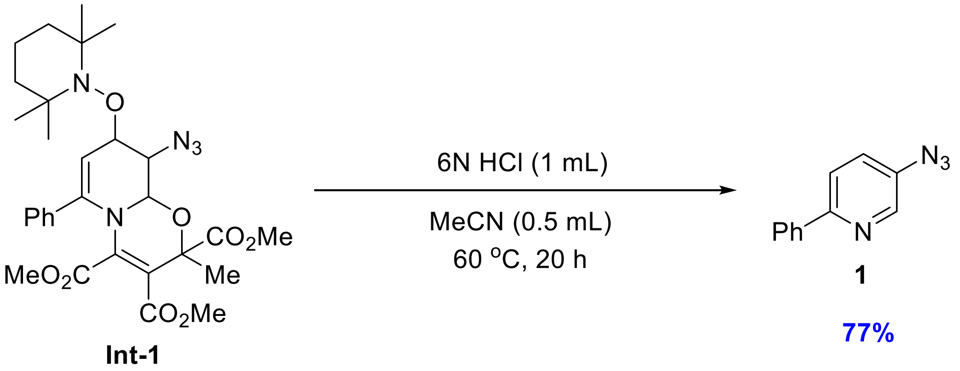

Entry	Variation from above	Yield of **1** (%)
1	2N HCl	46%
2	2N HCl, 40 °C	44%
3	2N HCl, 80 °C	40%
4	4N HCl	55%
5	12N HCl	64%
6	2 h	64%
7	1 mL HCl, 1 mL MeCN	57%
8	TMSOTf (2.5 equiv) as additive	66%
9	BF_3OEt_2 (2.5 equiv) as additive	66%
10	0.5 mL MeOH	72%
11	0.5 mL *t*BuOMe	64%
12	0.5 mL DCM	34%
13	0.5 mL EA	65%
14	0.5 mL THF	60%

Synthetic application

5-(4-(Phenoxymethyl)-1*H*-1,2,3-triazol-1-yl)-2-phenylpyridine (56):

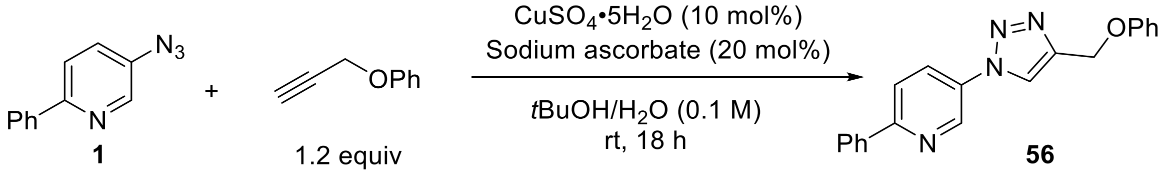

According to a literature reported procedure[9], to a 10 mL flask, **1** (0.10 mmol, 1.0 equiv.), (prop-2-yn-1-yloxy)benzene (0.12 mmol, 1.2 equiv.), $CuSO_4{\bullet}5H_2O$ (0.01 mmol, 10 mol%) and sodium ascorbate (0.02 mmol, 20 mol%) were added and dissolved in 2 mL of *t*BuOH:H_2O (1:1) solvent. The reaction mixture was allowed to stir for 18 h at room temperature. After completion of the reaction, as monitored by TLC, water was added and the mixture was extracted with ethylacetate (5 mL x 3). The combined organic phase was dried over $MgSO_4$ and filtered. The solvent was removed under reduced pressure and the residue was purified by column chromatography on silica gel to give a white soild **56**.

Yield: 88% (29.0 mg). White solid. R_f 0.20 (pentane/EtOAc 9/1); **^{1}H NMR** (300 MHz, $CDCl_3$) δ 9.05 (dd, *J* = 2.7, 0.9 Hz, 1H), 8.20 (dd, *J* = 8.7, 2.7 Hz, 1H), 8.14 (s, 1H), 8.05 (dd, *J* = 8.1, 1.8 Hz, 2H), 7.92 (dd, *J* = 8.7, 0.9 Hz, 1H), 7.58 – 7.43 (m, 3H), 7.39 – 7.29 (m, 2H), 7.10 – 6.88 (m, 3H), 5.34 (s, 2H). **^{13}C NMR** (101 MHz, $CDCl_3$) δ 158.2, 158.0, 145.8, 141.4, 138.0, 132.3, 129.9, 129.8, 129.1(2), 129.0(7), 127.2, 121.6, 121.0, 120.9, 114.9, 62.1. **HRMS** (ESI): calcd. for $[C_{20}H_{16}N_4ONa]^+$, $[M+Na]^+$: 351.1216; found: 351.1216. **IR** (neat, cm^{-1}) = 3065*br*, 2350*m*, 2309*m*, 1747*m*, 1601*s*, 1248*s*, 1173*w*, 1047*s*, 834*m*, 750*s*, 733*s*, 688*s*. **Mp**: 172.8 – 173.8 °C.

5-(5-Methyl-4-phenyl-1*H*-1,2,3-triazol-1-yl)-2-phenylpyridine (57) and 1-(5-Phenyl-1-(6-phenylpyridin-3-yl)-1*H*-1,2,3-triazol-4-yl)ethan-1-one (58):

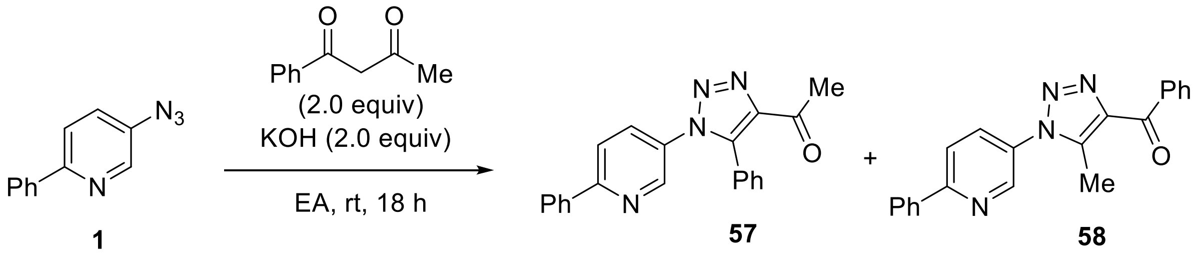

To a 10 mL flask, **1** (0.10 mmol, 1.0 equiv.), 1-phenylbutane-1,3-dione (0.20 mmol, 2.0 equiv.), and KOH (0.20 mmol, 2.0 equiv.) were added followed by 2 mL of dry ethylacetate. The reaction mixture was allowed to stir for 18 h at room temperature. After the completion of the reaction, as monitored by TLC, the solvent was removed under reduced pressure and the residue was was purified by column chromatography on silica gel to give **57** and **58** as a white soild.

Analysis for **57**: Yield: 68% (23.0 mg). Light yellow solid. R_f 0.10 (pentane/EtOAc 9/1); **^{1}H NMR** (400 MHz, $CDCl_3$) δ 8.60 (dd, *J* = 2.6, 0.8 Hz, 1H), 8.10 – 7.94 (m, 2H), 7.78 (dd, *J* = 8.4, 0.8 Hz, 1H), 7.69 (dd, *J* = 8.4, 2.6 Hz, 1H), 7.51 – 7.36 (m, 6H), 7.35 – 7.30 (m, 2H), 2.78 (s, 3H). **^{13}C NMR** (101 MHz, $CDCl_3$) δ 192.8, 158.3, 145.6, 143.9, 139.5, 137.8, 133.1, 131.3, 130.5, 130.3, 130.1, 129.1, 128.9, 127.2, 125.3, 120.5, 28.5. **HRMS** (ESI): calcd. for $[C_{21}H_{16}N_4O]^+$, $[M]^+$: 340.1319; found: 340.1315. **IR** (neat, cm^{-1}) = 3064*w*, 2309*w*, 1690*s*, 1480*s*, 1450*w*, 1418*w*, 1370*m*, 1294*m*, 1199*m*, 990*m*, 951*m*, 844*w*, 767*m*, 738*s*, 693*s*, 586*w*. **Mp**: 146.6 – 147.6 °C.

Analysis for **58**: Yield: 22% (8.0 mg). White solid. R_f 0.15 (pentane/EtOAc 9/1); **^{1}H NMR** (400 MHz, $CDCl_3$) δ 8.87 (dd, *J* = 2.4, 0.8 Hz, 1H), 8.52 – 8.26 (m, 2H), 8.09 (dd, *J* = 8.2, 1.6 Hz, 2H), 8.02 – 7.87 (m, 2H), 7.67 – 7.60 (m, 1H), 7.58 – 7.48 (m, 5H), 2.77 (s, 3H). **^{13}C NMR** (101 MHz, $CDCl_3$) δ 187.5, 159.1, 145.8, 144.0, 140.3, 137.9, 137.4, 133.6, 133.3, 130.9, 130.8, 130.2, 129.2, 128.5, 127.4, 121.0, 10.8. **HRMS** (ESI): calcd. for $[C_{21}H_{17}N_4O]^+$, $[M+H]^+$: 341.1397; found: 341.1395. **IR** (neat, cm^{-1}) = 3064*w*, 1647*s*, 1486*s*, 1447*m*, 1419*m*, 1361*m*, 1255*s*, 917*s*, 738*s*, 692*s*, 656*w*. **Mp**: 171.6 – 172.6 °C.

6-Phenylpyridin-3-amine (59):

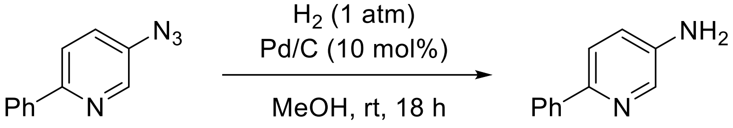

1 **59**

To a 5 mL flask, **1** (0.10 mmol, 1.0 equiv.), Pd/C (0.10 mmol, 10 mol%) and MeOH (1 mL) were added. The reaction tube was exchanged with H_2 for three times. The reaction mixture was allowed to stir for 18 h at room temperature. After completion of the reaction, as monitored by TLC, the solvent was removed under reduced pressure and the residue was purified by column chromatography on silica gel to give a white soild **59**. The analytical data match the reported one.[7]

Yield: 95% (16.0 mg). Colorless soild. R_f 0.10 (pentane/EtOAc 4/1); **^{1}H NMR** (300 MHz, $CDCl_3$) 8.18 (dd, *J* = 2.8, 0.8 Hz, 1H), 7.97 – 7.83 (m, 2H), 7.54 (dd, *J* = 8.5, 0.8 Hz, 1H), 7.46 – 7.37 (m, 2H), 7.36 – 7.28 (m, 1H), 7.05 (dd, *J* = 8.5, 2.8 Hz, 1H), 3.75 (bs, 2H).

X-ray

X-ray crystal structure analysis of 34 (stu10836): A pale yellow, prism shaped specimen of $C_{15}H_{19}N_3$, approximate dimensions 0.08×0.081×0.132 mm^3, was used for the X-ray crystallographic analysis. The crystals were crystallised from DCM and toluene. The X-ray intensity data of stu10836 were measured on a Bruker D8 VENTURE KAPPA diffractometer system equipped with a microfocus sealed tube (λ = 0.71073 Å) and a multilayer mirror monochromator. A total of 2566 frames were collected. The total exposure time was 15.62 hours. The frames were integrated with the SAINT V8.41 package using a narrow-frame algorithm. The integration of the data using a monoclinic unit cell yielded a total of 40175 reflections to a maximum θ angle of 27.52° (0.77 Å resolution), of which 2948 were independent (average redundancy 13.63, completeness = 100.0%, Rint = 5.89%, Rsig = 2.46%) and 2563 (86.9%) were greater than $2\sigma(F^2)$. The final cell constants of a = 11.9884(10) Å, b = 8.6961(6) Å, c = 12.2665(9) Å, volume = 1278.14(17) $Å^3$, are based upon the refinement of the XYZ-centroids of 9961 reflections above 20 $\sigma(I)$ with 2.34° < 2θ < 27.49°. Data were corrected for absorption effects using the Multi-Scan method in SADABS 2016/2. The calculated minimum and maximum transmission coefficients (based on crystal size) are 0.990 and 0.994. The structure was solved by SHELXT 2018/2 and refined using the SHELXL-2019/2 Software, in the space group $P2_1/n$ (14), with Z = 4 for the formula unit $C_{15}H_{19}N_3$. The final anisotropic full-matrix least-squares refinement on F^2 with 165 variables against 2948 data points converged at R_1 = 3.89%, for the observed data and wR_2 = 10.01% for all data. The goodness-of-fit on F^2 was 1.05. The largest peak in the final difference electron density synthesis was 0.29 $e^-/Å^3$ and the deepest hole was −0.21 $e^-/Å^3$ with an RMS deviation of 0.040 $e^-/Å^3$. On the basis of the final model, the calculated density was 1.25 g/cm^3 and $F(000)$, 520 e^-. CCDC number: 2468722.

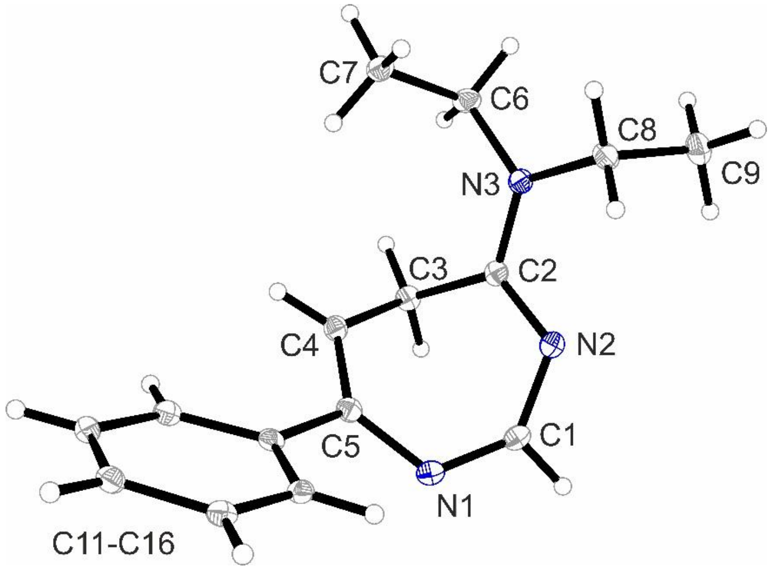

Figure S3: Crystal structure of compound **34**. (Thermal ellipsoids are shown at 30% probability.)

X-ray crystal structure analysis of 43 (stu10845): A colourless, prism shaped specimen of $C_{15}H_{18}ClN_3$, approximate dimensions 0.047×0.105×0.154 mm^3, was used for the X-ray crystallographic analysis. The crystals were crystallised from deuterated trichloromethane. The X-ray intensity data of stu10845 were measured on a Bruker D8 VENTURE KAPPA diffractometer system equipped with a microfocus sealed tube (λ = 0.71073 Å) and a multilayer mirror monochromator. The specimen was held at 100(2) K during the measurement with an Oxford Cryostream 1000 low temperature device. A total of 2910 frames were collected. The total exposure time was 14.37 hours. The frames were integrated with the SAINT V8.41 package using a narrow-frame algorithm. The integration of the data using a monoclinic unit cell yielded a total of 43275 reflections to a maximum θ angle of 27.51° (0.77 Å resolution), of which 3254 were independent (average redundancy 13.30, completeness = 99.9%, Rint = 3.28%, Rsig = 1.53%) and 2975 (91.4%) were greater than $2\sigma(F^2)$. The final cell constants of a = 6.4095(3) Å, b = 9.7903(4) Å, c = 22.7445(9) Å, volume = 1417.62(10) $Å^3$, are based upon the refinement of the XYZ-centroids of 9896 reflections above 20 $\sigma(I)$ with 2.75° < 2θ < 27.49°. Data were corrected for absorption effects using the Multi-Scan method in SADABS 2016/2. The calculated minimum and maximum transmission coefficients (based on crystal size) are 0.961 and 0.988. The structure was solved by SHELXT 2018/2 and refined using the SHELXL-2019/2 Software, in the space group $P2_1/c$ (14), with Z = 4 for the formula unit $C_{15}H_{18}ClN_3$. The final anisotropic full-matrix least-squares refinement on F^2 with 174 variables against 3254 data points converged at R_1 = 3.07%, for the observed data and wR_2 = 8.34% for all data. The goodness-of-fit on F^2 was 1.03. The largest peak in the final difference electron density synthesis was 0.30 $e^-/Å^3$ and the deepest hole was −0.22 $e^-/Å^3$ with an RMS deviation of 0.042 $e^-/Å^3$. On the basis of the final model, the calculated density was 1.29 g/cm^3 and $F(000)$, 584 e^-. CCDC number: 2468723.

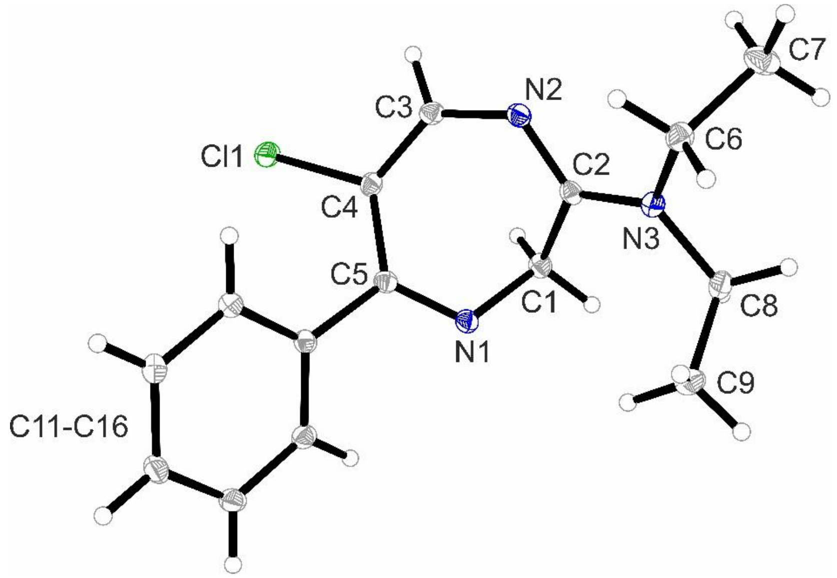

Figure S4: Crystal structure of compound **43**. (Thermal ellipsoids are shown at 30% probability.)

X-ray crystal structure analysis of 48 (stu10792): A colourless, plate shaped specimen of $C_{12}H_{10}N_2O$, approximate dimensions 0.04×0.118×0.127 mm^3, was used for the X-ray crystallographic analysis. The crystals were crystallised from DCM, methanol and pentane. The X-ray intensity data of stu10792 were measured on a Bruker D8 VENTURE KAPPA diffractometer system equipped with a microfocus sealed tube (λ = 0.71073 Å) and a multilayer mirror monochromator. A total of 2139 frames were collected. The total exposure time was 17.70 hours. The frames were integrated with the SAINT V8.41 package using a narrow-frame algorithm. The integration of the data using an orthorhombic unit cell yielded a total of 106874 reflections to a maximum θ angle of 27.47° (0.77 Å resolution), of which 4439 were independent (average redundancy 24.08, completeness = 99.8%, Rint = 10.05%, Rsig = 3.64%) and 3380 (76.1%) were greater than $2\sigma(F^2)$. The final cell constants of a = 42.353(7) Å, b = 7.4990(10) Å, c = 12.2421(16) Å, volume = 3888.2(9) $Å^3$, are based upon the refinement of the XYZ-centroids of 9995 reflections above 20 $\sigma(I)$ with 2.72° < 2θ < 26.88°. Data were corrected for absorption effects using the Multi-Scan method in SADABS 2016/2. The calculated minimum and maximum transmission coefficients (based on crystal size) are 0.989 and 0.996. The structure was solved by SHELXT 2018/2 and refined using the SHELXL-2019/2 Software, in the space group *Pccn* (56), with Z = 16 for the formula unit $C_{12}H_{10}N_2O$. The final anisotropic full-matrix least-squares refinement on F^2 with 281 variables against 4439 data points 2 and 2 restraints converged at R_1 = 4.55%, for the observed data and wR_2 = 12.34% for all data. The goodness-of-fit on F^2 was 1.02. The largest peak in the final difference electron density synthesis was 0.26 $e^-/Å^3$ and the deepest hole was −0.23 $e^-/Å^3$ with an RMS deviation of 0.046 $e^-/Å^3$. On the basis of the final model, the calculated density was 1.35 g/cm^3 and $F(000)$, 1664 e^-. The hydrogens at N1A and N1B atoms were refined freely, but with N-H distance restraints (DFIX). CCDC number: 2468724.

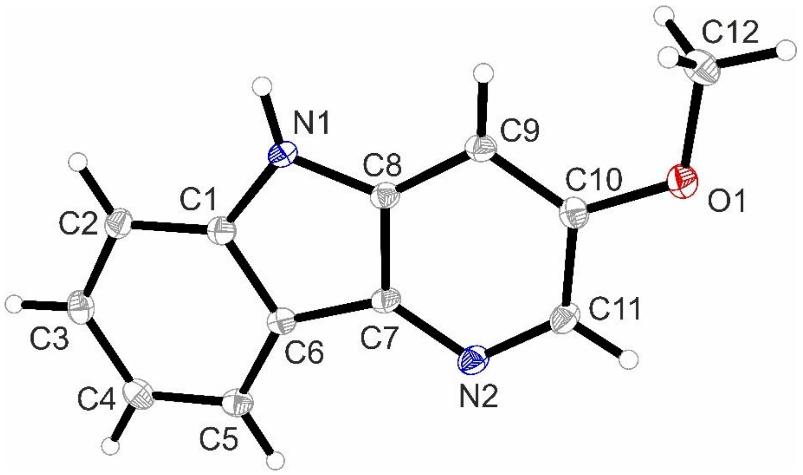

Figure S5: Crystal structure of compound **48**. Only one molecule (molecule named with suffix A) of two found in the asymmetric unit is shown. Thermal ellipsoids are shown at 30% probability.

NMR Spectra

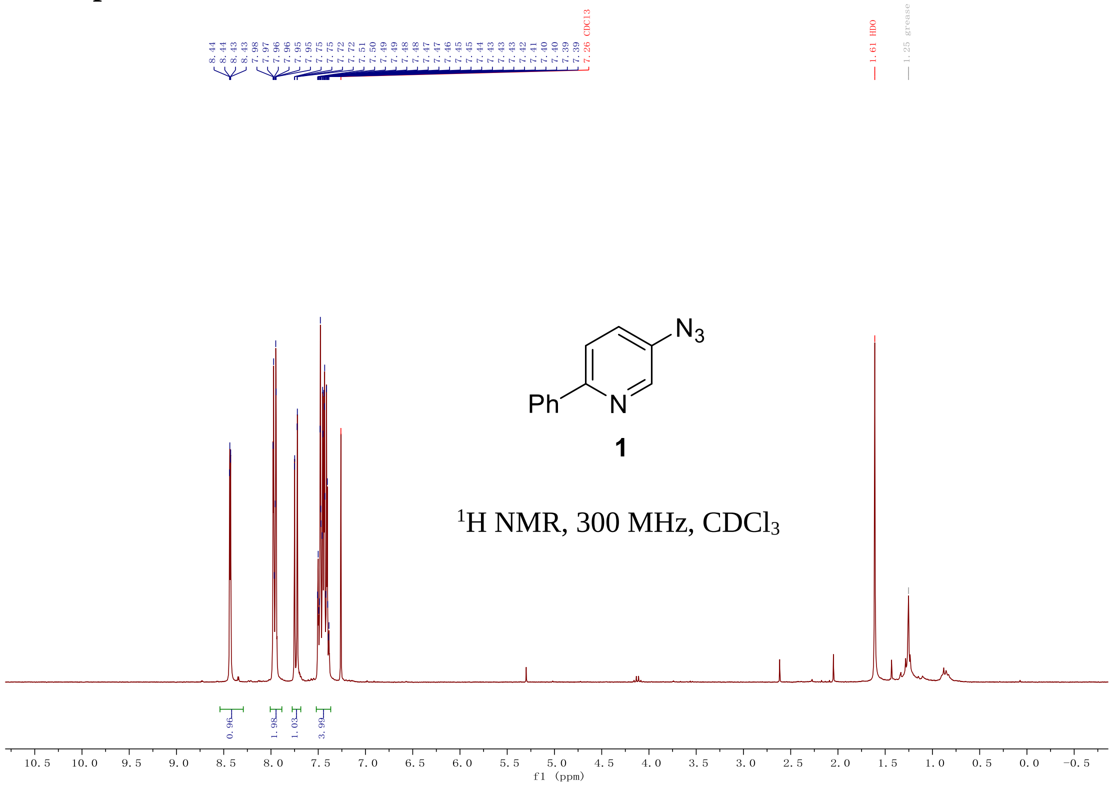

Ph
N
N3
1
1H NMR, 300 MHz, CDCl3
f1 (ppm)

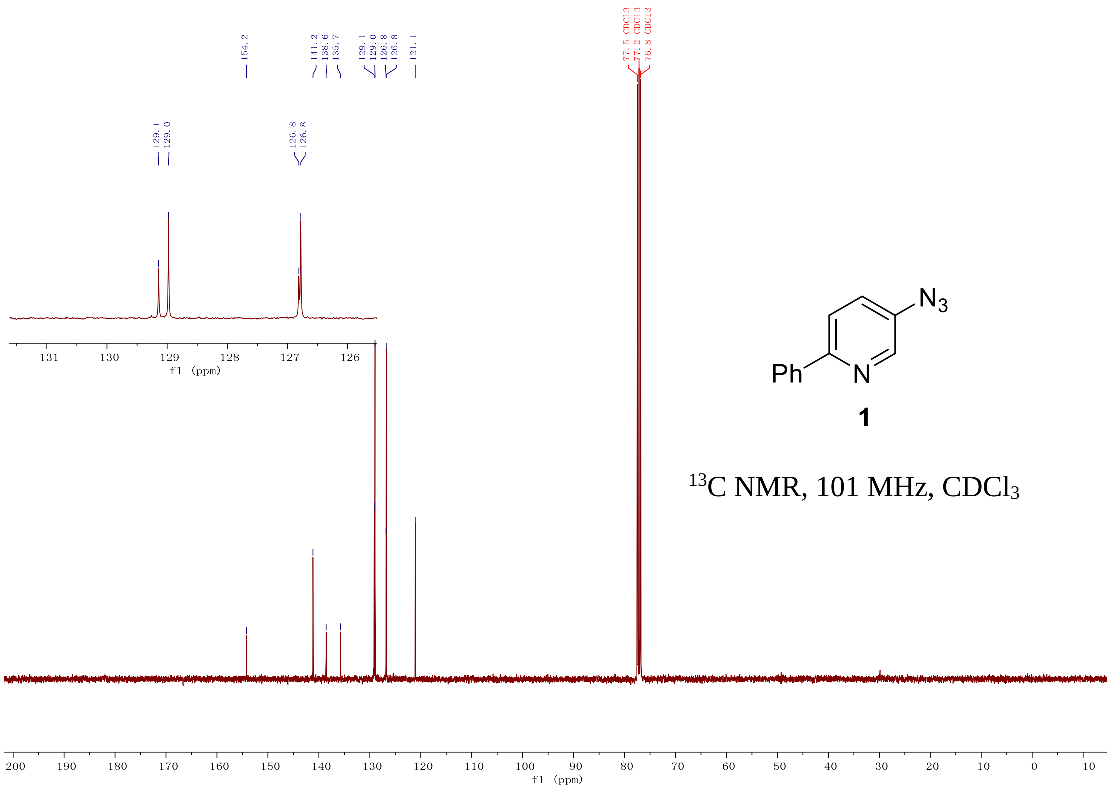

Ph
N
N3
1
13C NMR, 101 MHz, CDCl3
f1 (ppm)

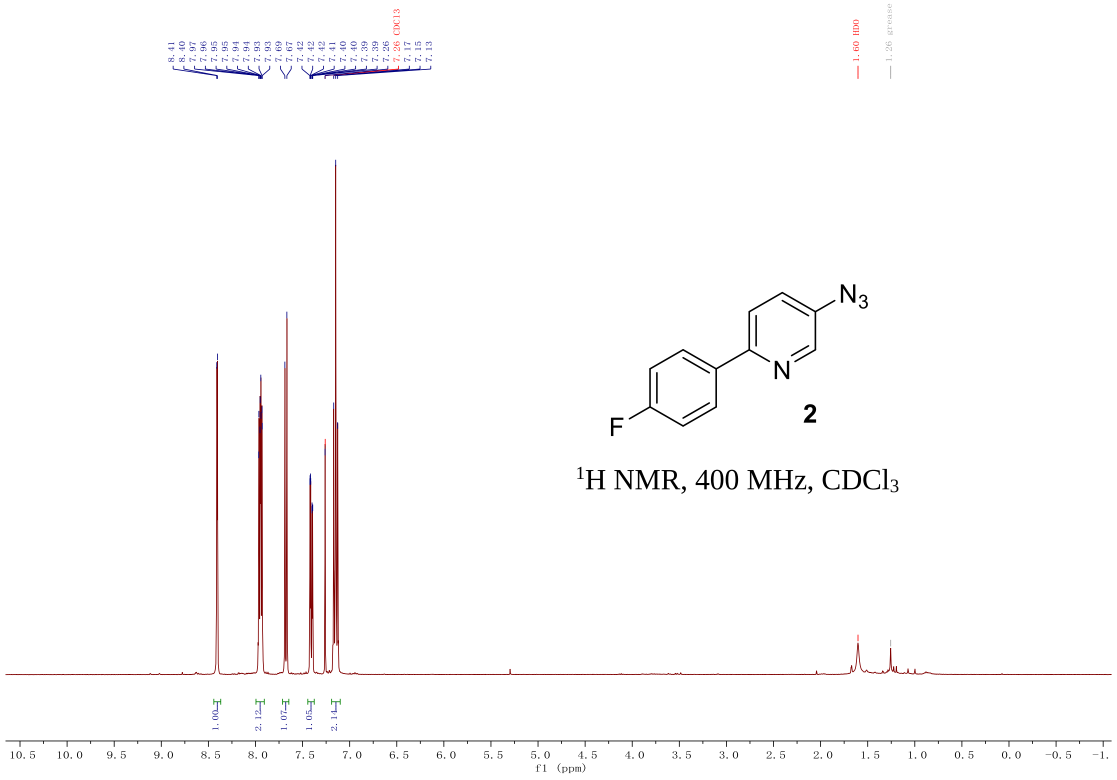
N3
N
F
2
1H NMR, 400 MHz, CDCl3
f1 (ppm)

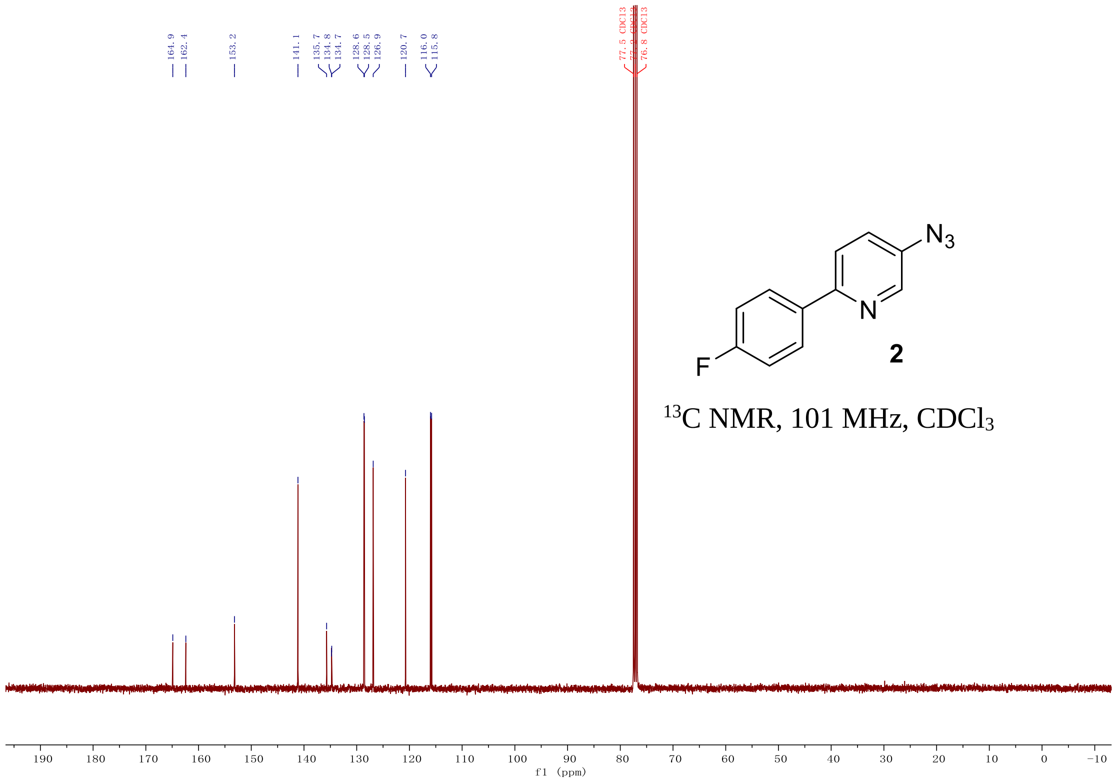
N3
N
F
2
13C NMR, 101 MHz, CDCl3
f1 (ppm)

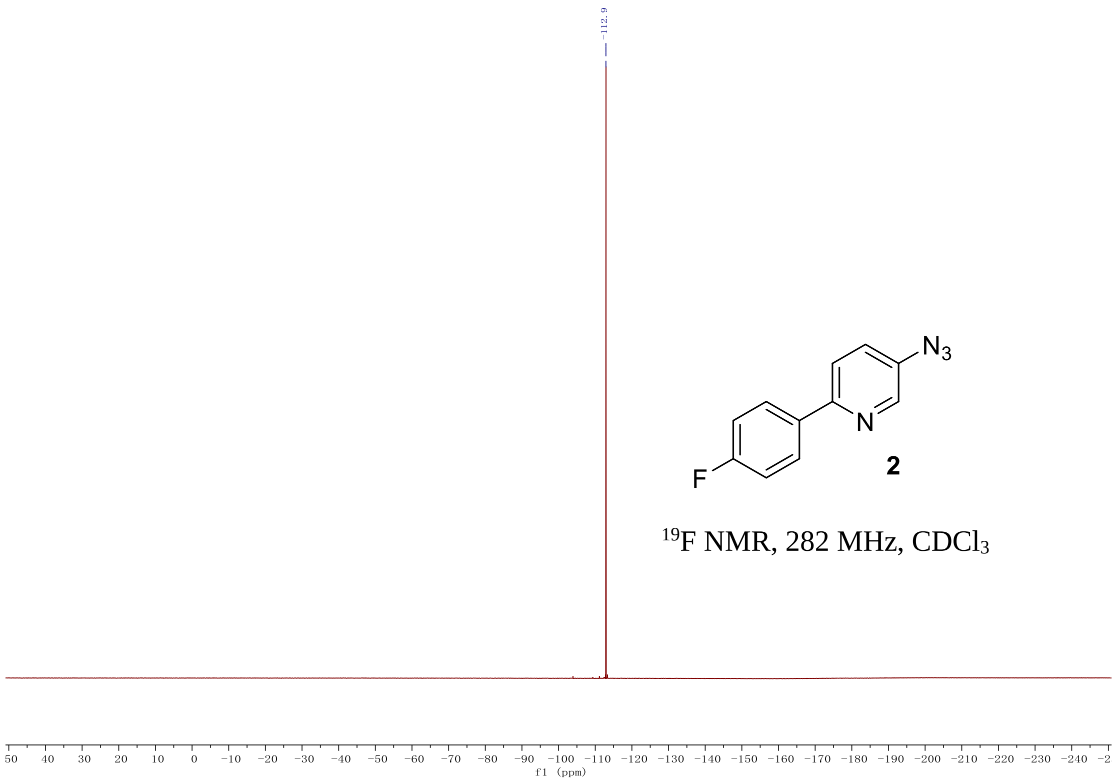

-112.9
N3
N
F
2
19F NMR, 282 MHz, CDCl3
50 40 30 20 10 0 -10 -20 -30 -40 -50 -60 -70 -80 -90 -100 -110 -120 -130 -140 -150 -160 -170 -180 -190 -200 -210 -220 -230 -240 -2
f1 (ppm)

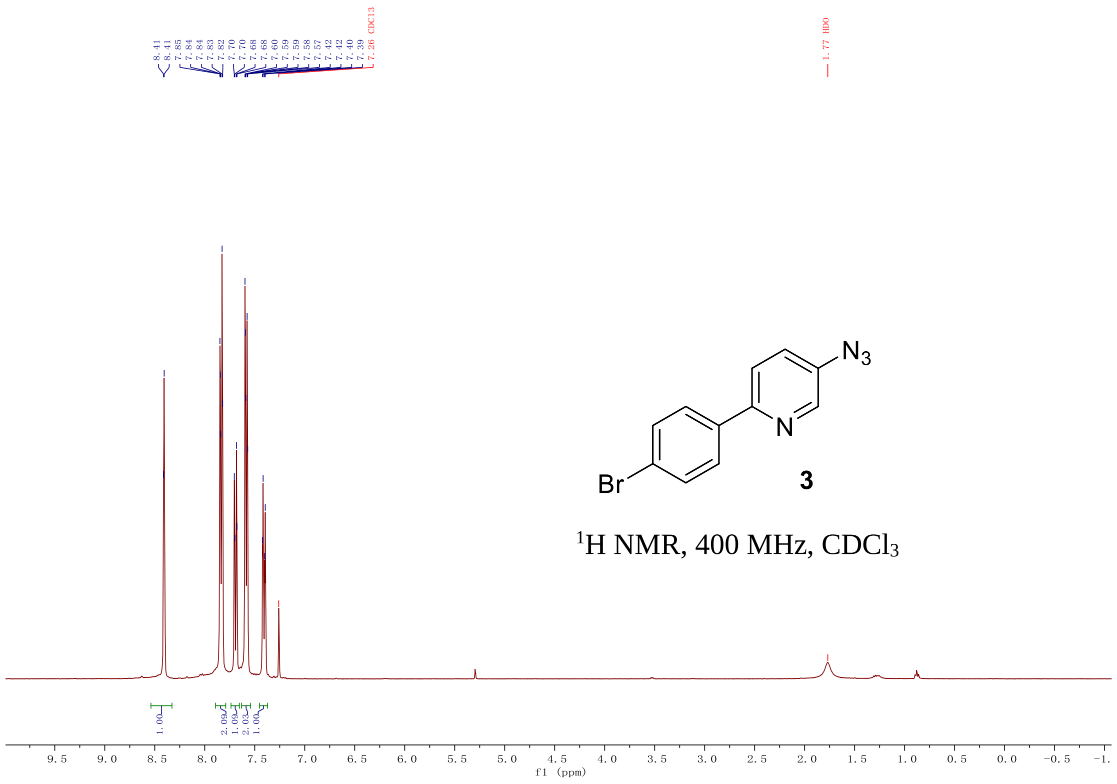

^{1}H NMR, 400 MHz, $CDCl_3$

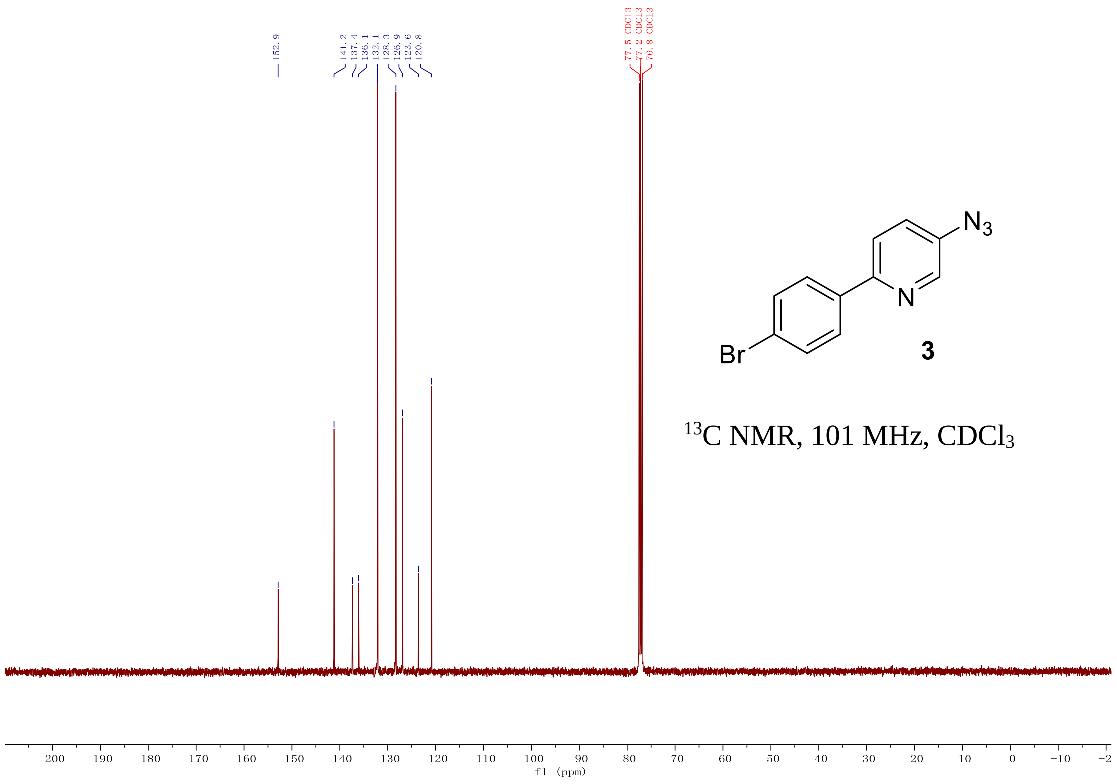

^{13}C NMR, 101 MHz, $CDCl_3$

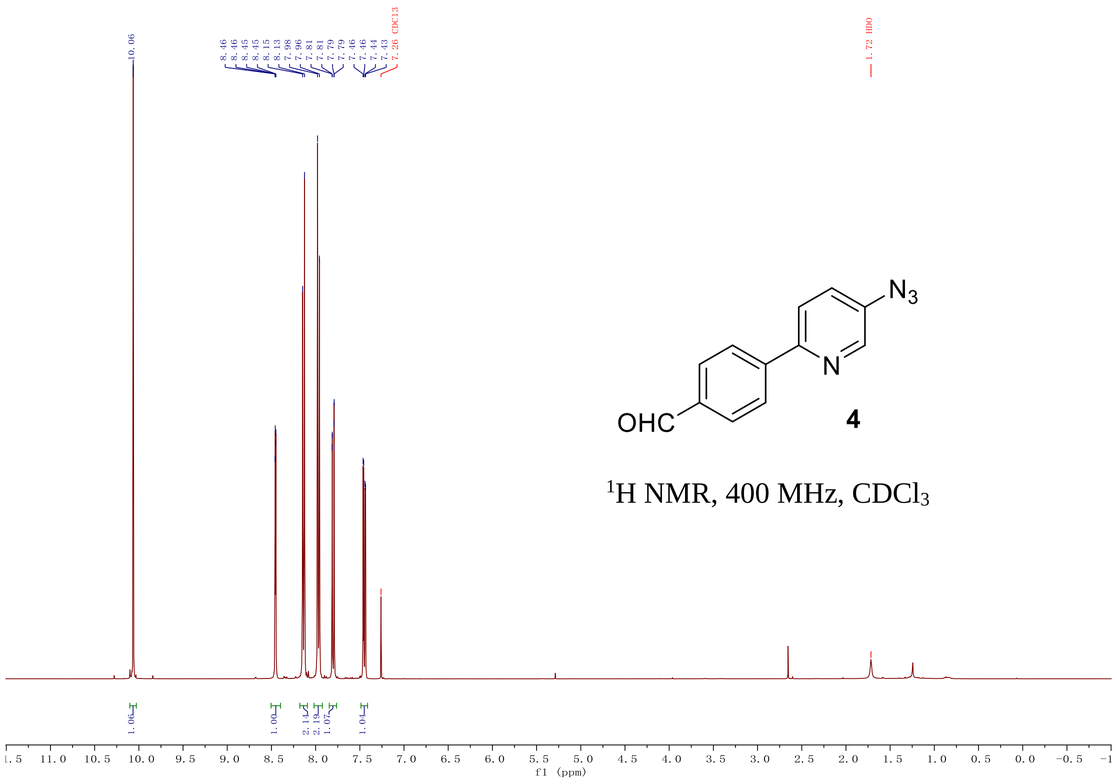
N3
N
OHC
4
1H NMR, 400 MHz, CDCl3
f1 (ppm)

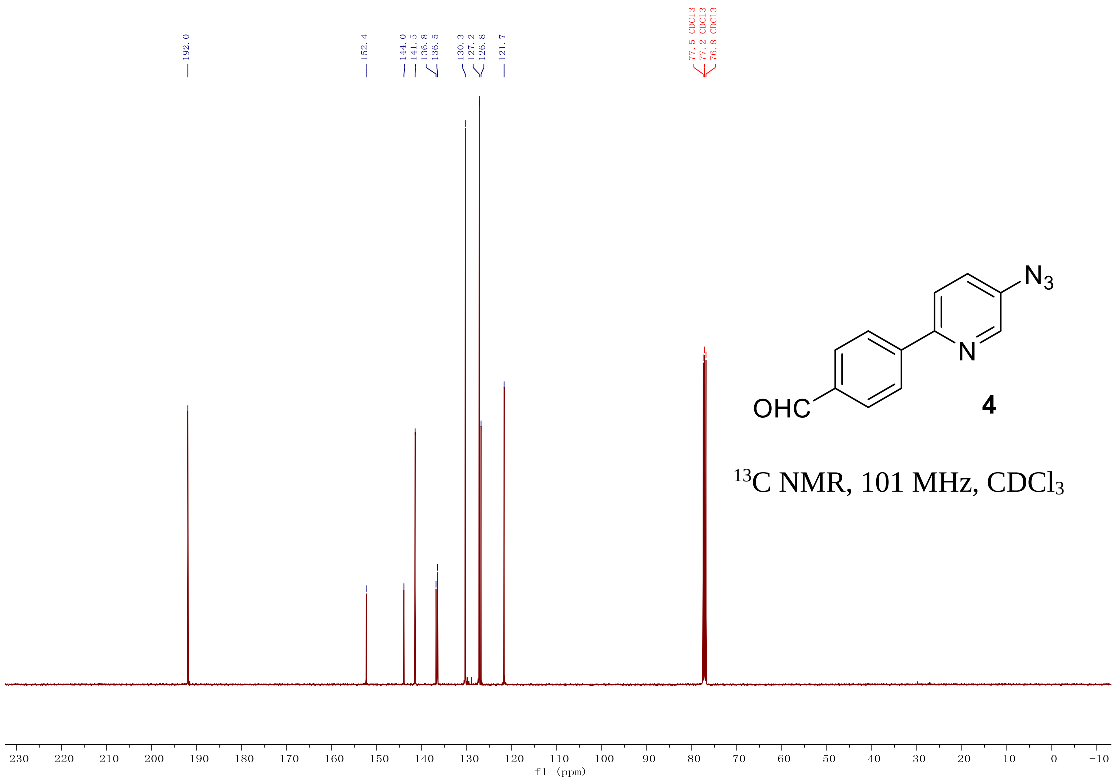
N3
N
OHC
4
13C NMR, 101 MHz, CDCl3
f1 (ppm)

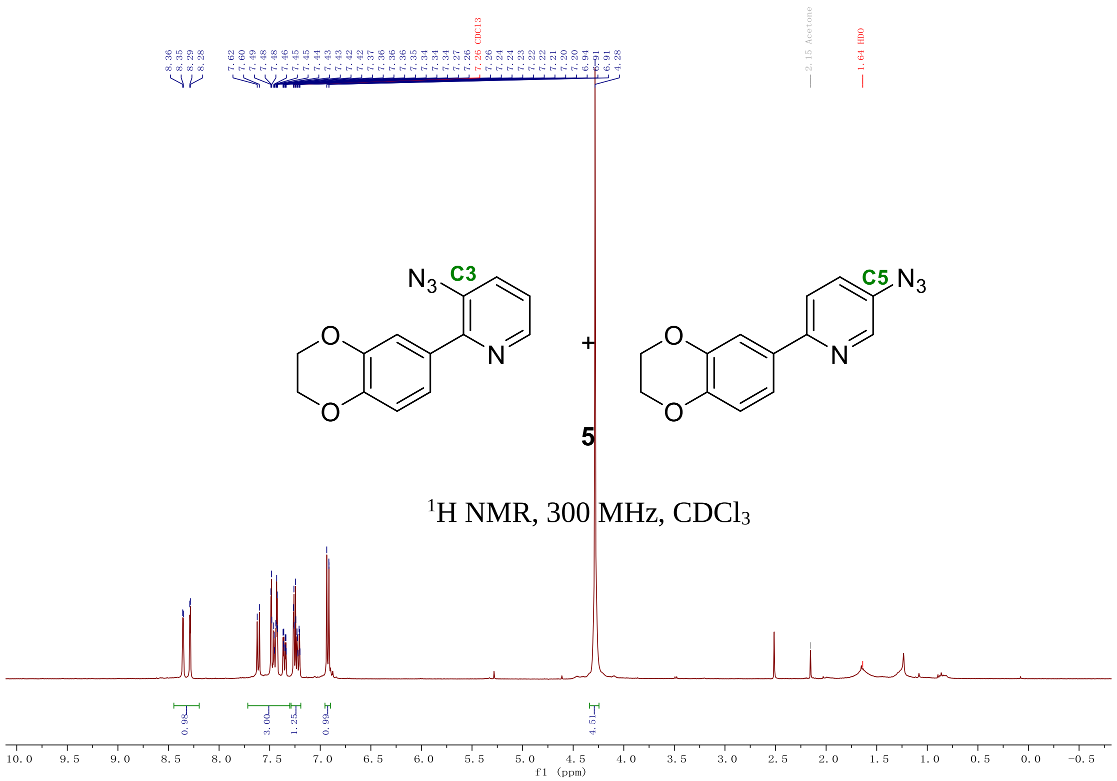

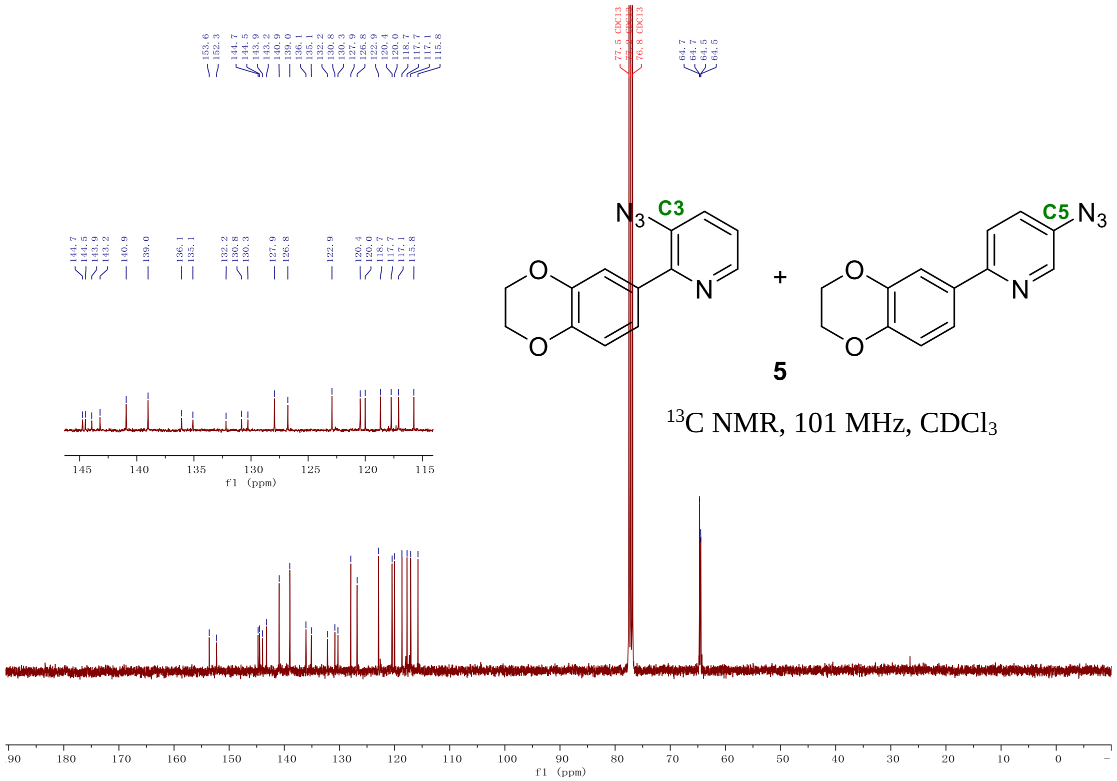

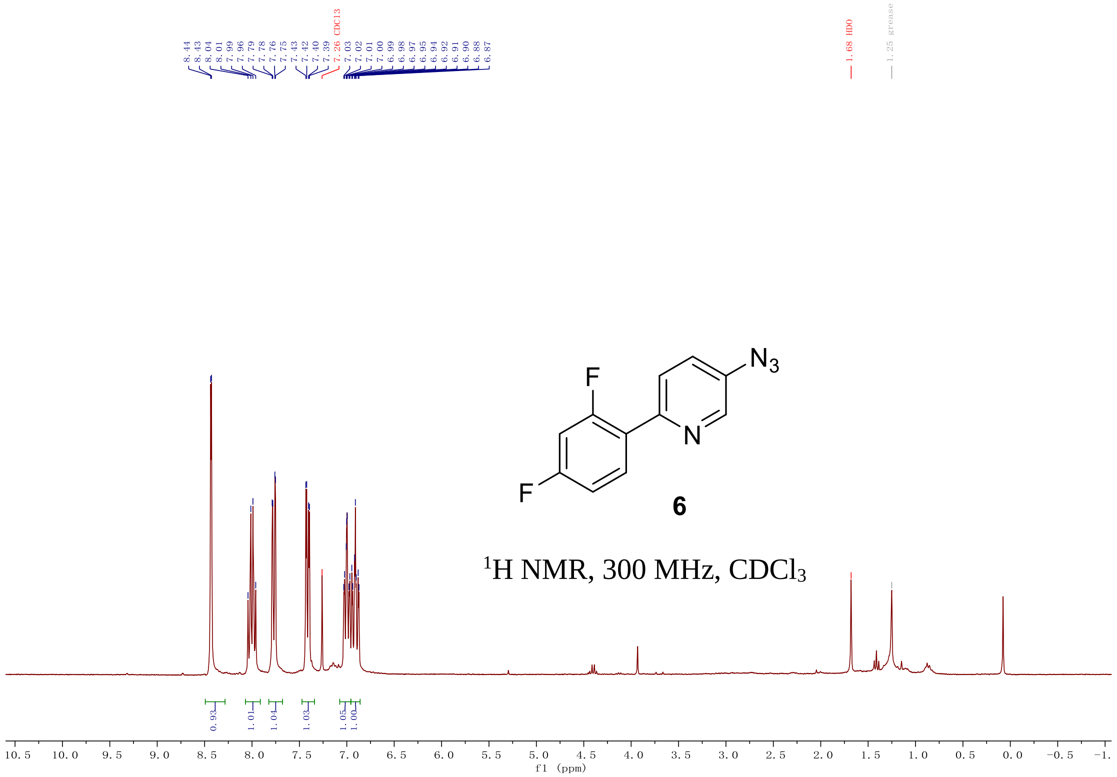

F
F
N
N3
6
1H NMR, 300 MHz, CDCl3
f1 (ppm)

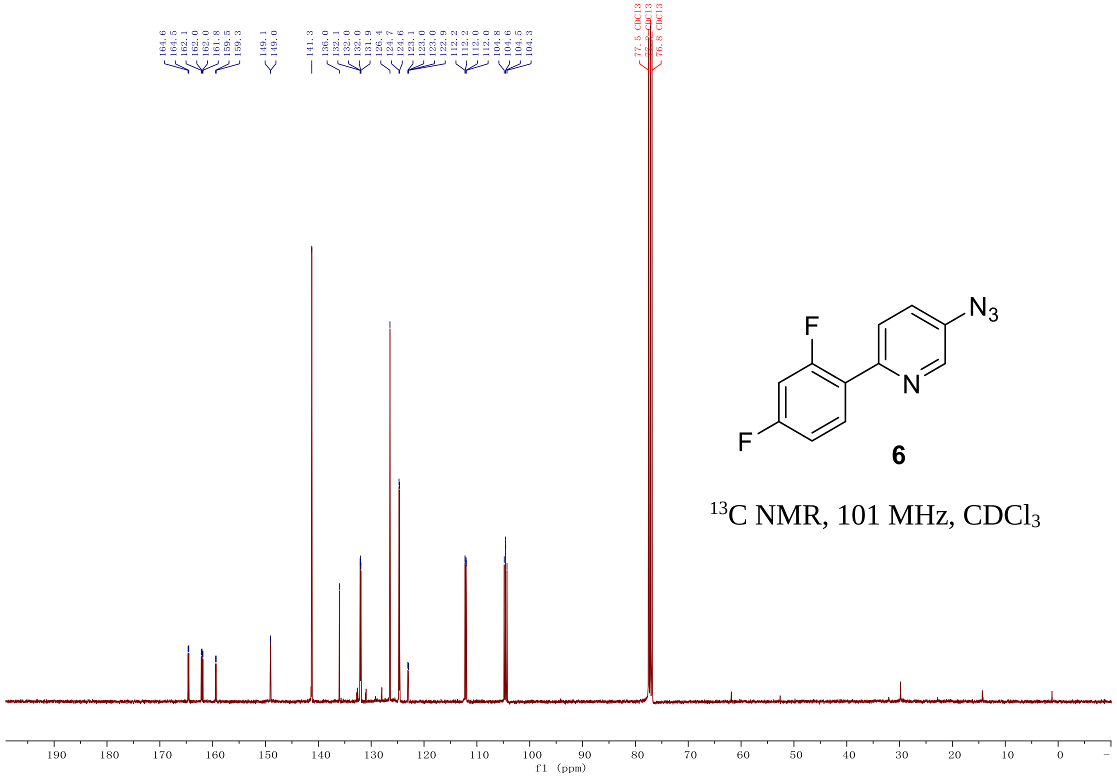

F
F
N
N3
6
13C NMR, 101 MHz, CDCl3
f1 (ppm)

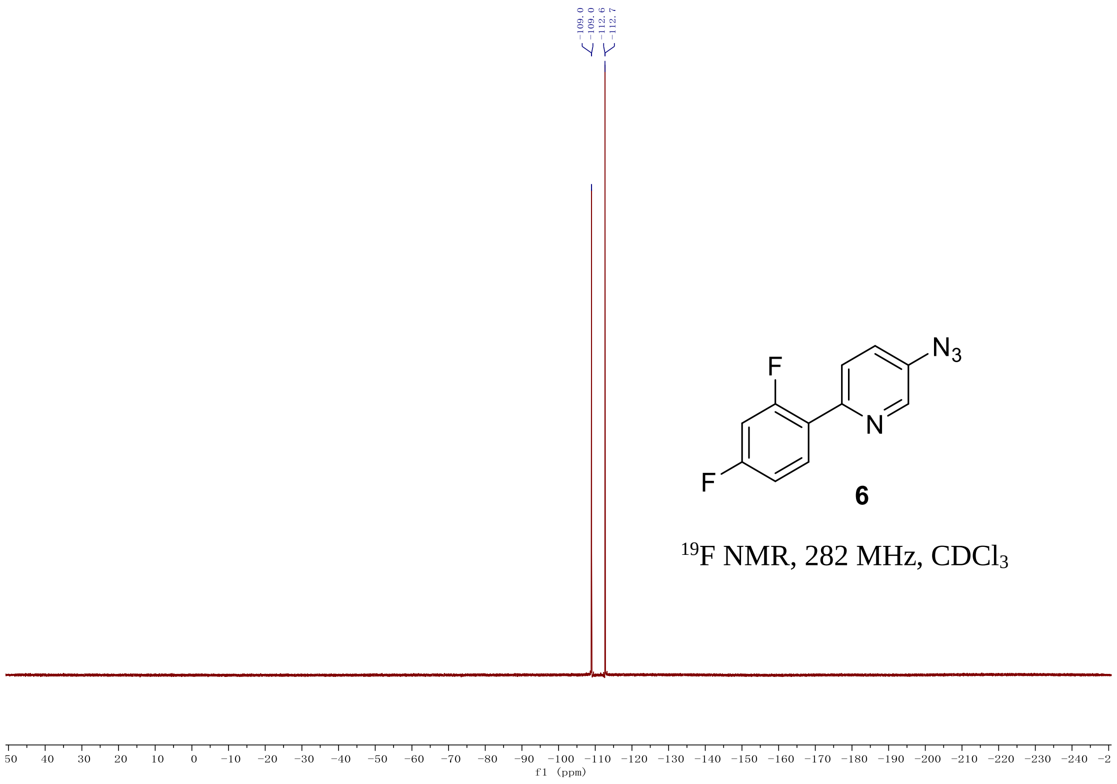

^{19}F NMR, 282 MHz, $CDCl_3$

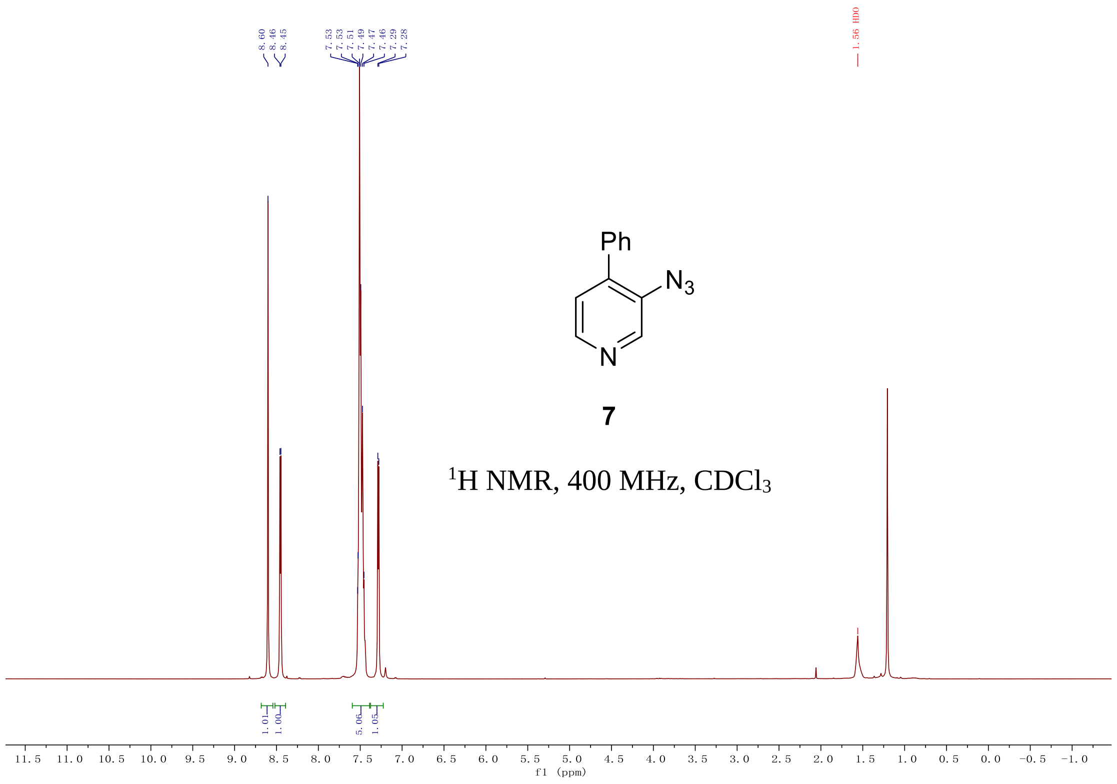

7

^{1}H NMR, 400 MHz, $CDCl_3$

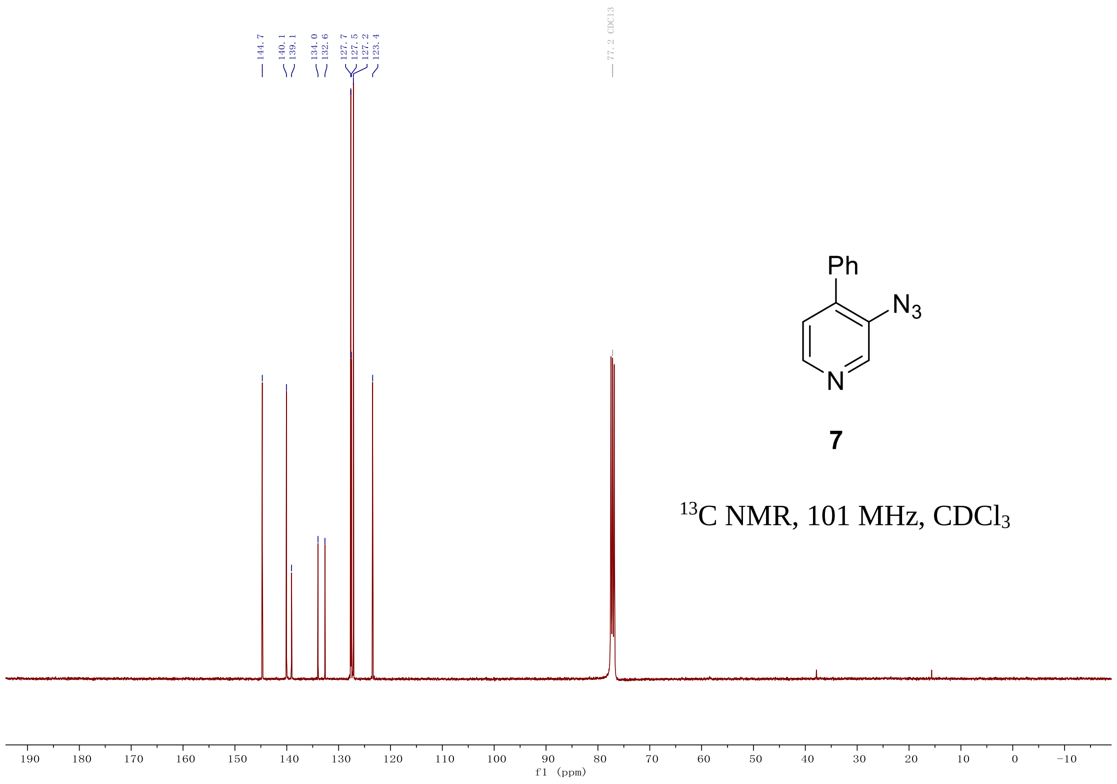

7

^{13}C NMR, 101 MHz, $CDCl_3$

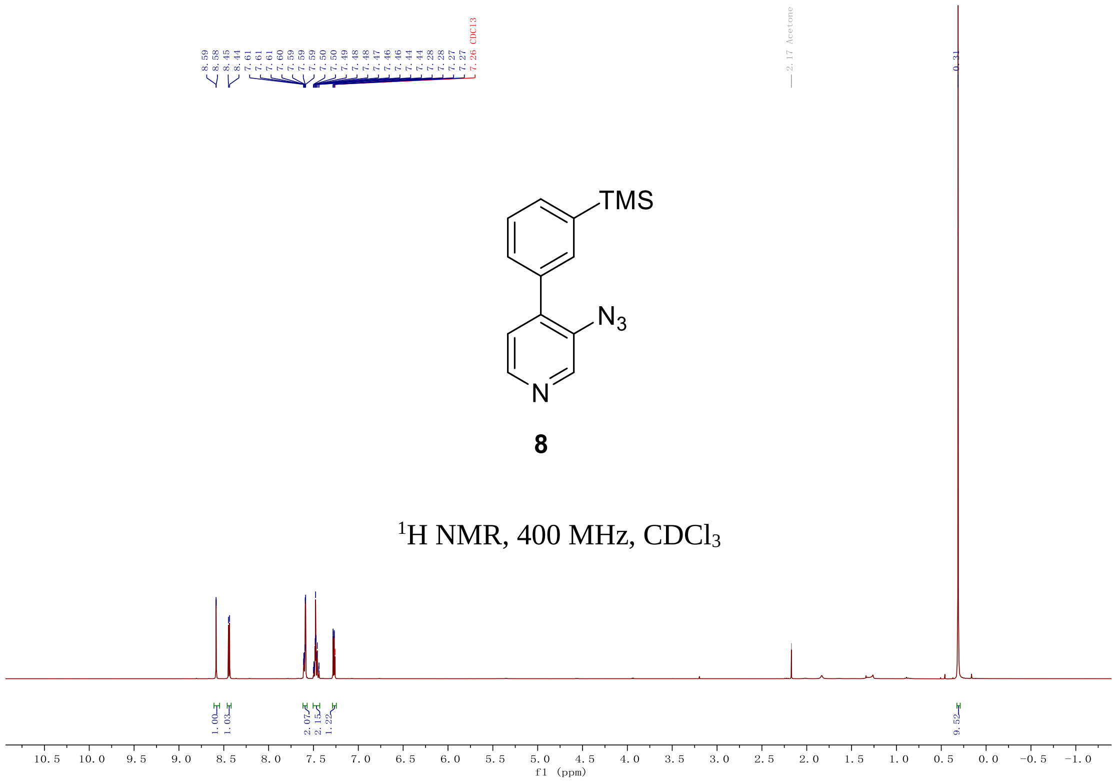

^{1}H NMR, 400 MHz, $CDCl_3$

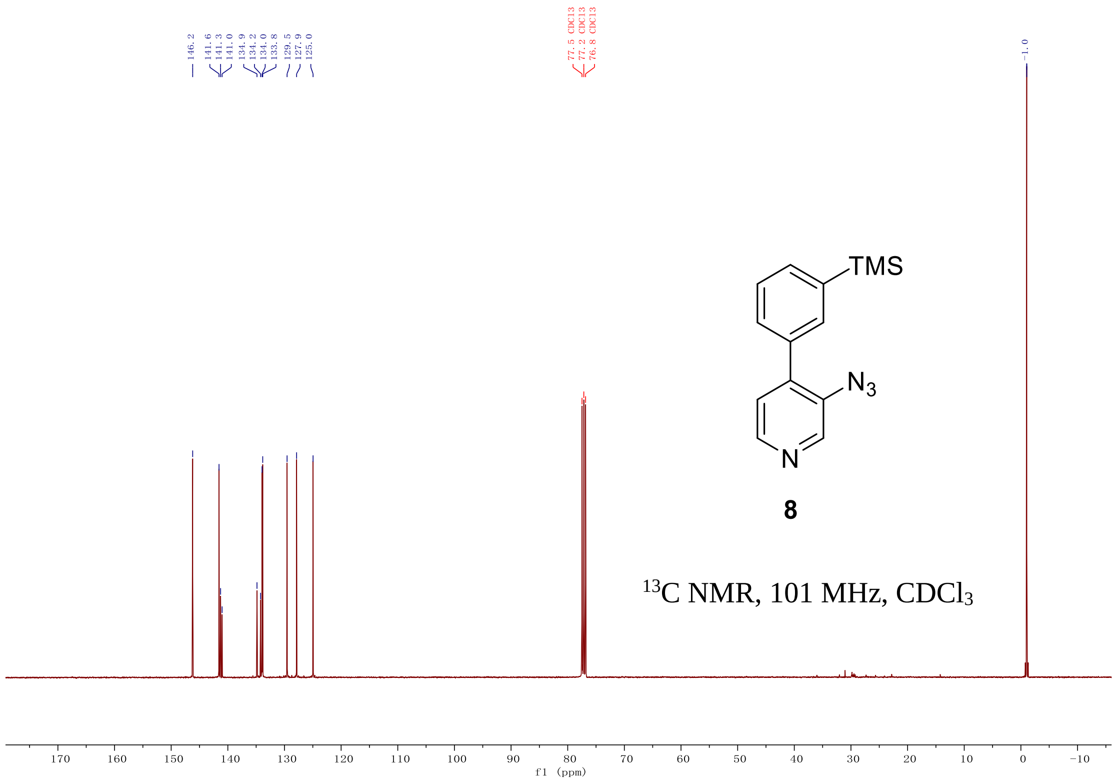

^{13}C NMR, 101 MHz, $CDCl_3$

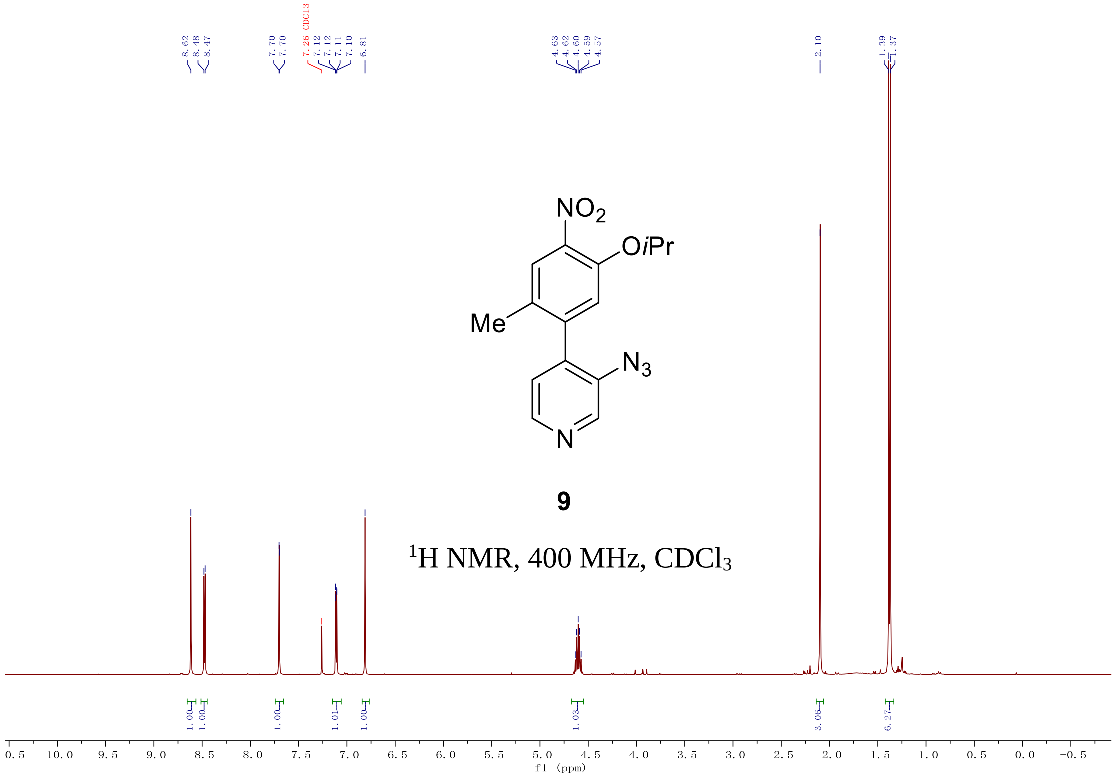
NO2
OiPr
Me
N3
N
9
1H NMR, 400 MHz, CDCl3
f1 (ppm)

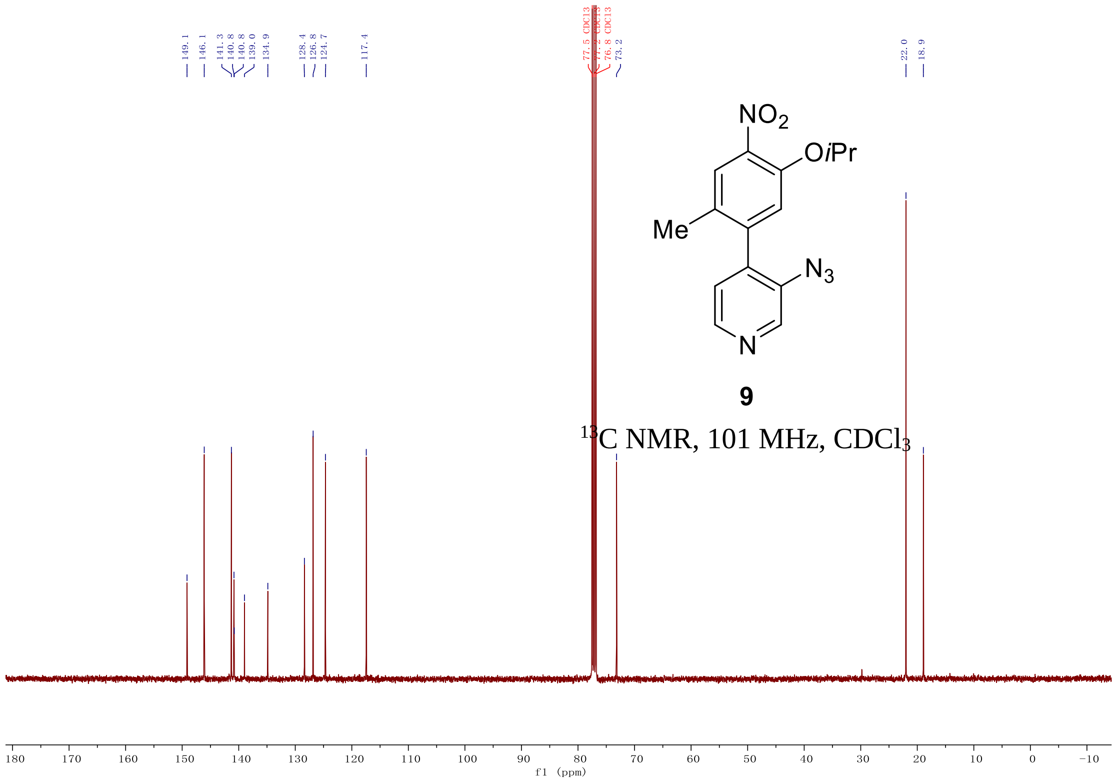
NO2
OiPr
Me
N3
N
9
13C NMR, 101 MHz, CDCl3
f1 (ppm)

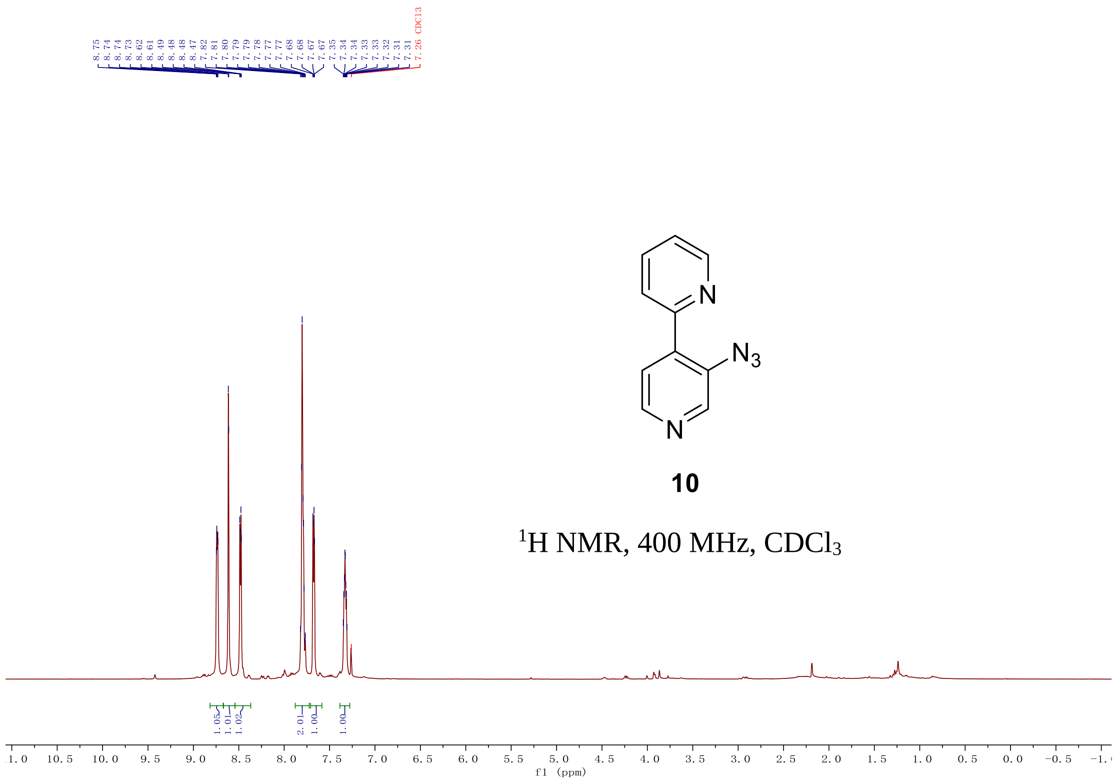

10
1H NMR, 400 MHz, CDCl3
f1 (ppm)

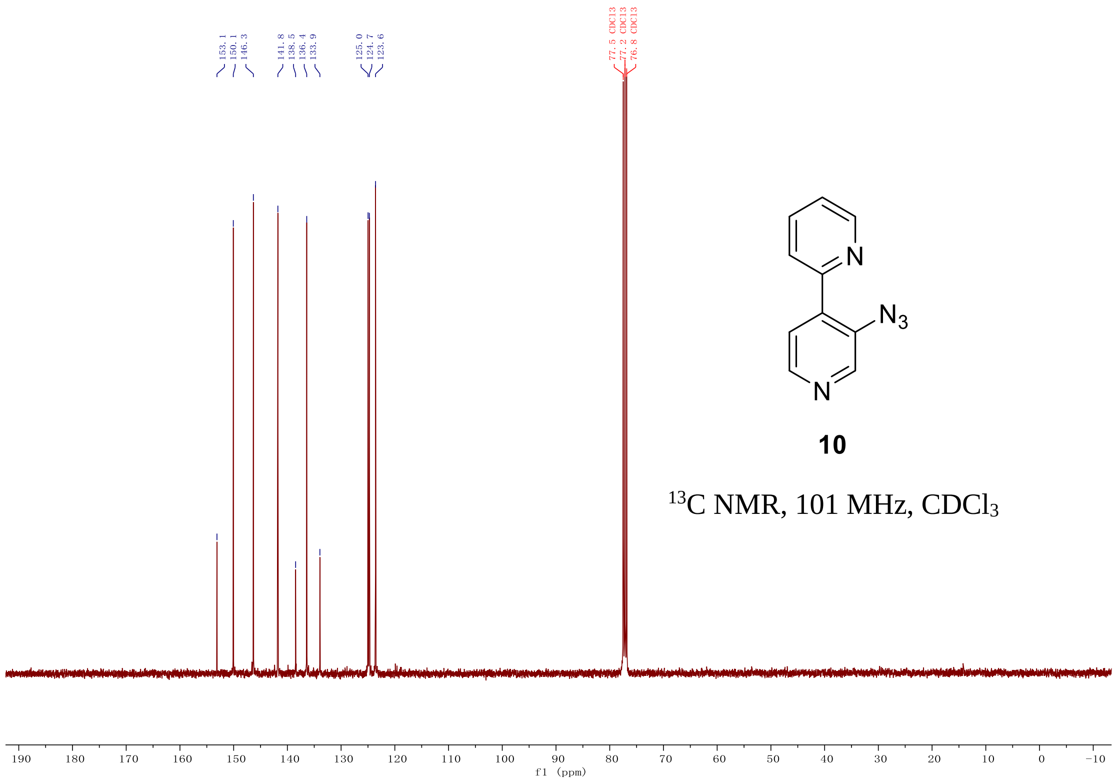

10
13C NMR, 101 MHz, CDCl3
f1 (ppm)

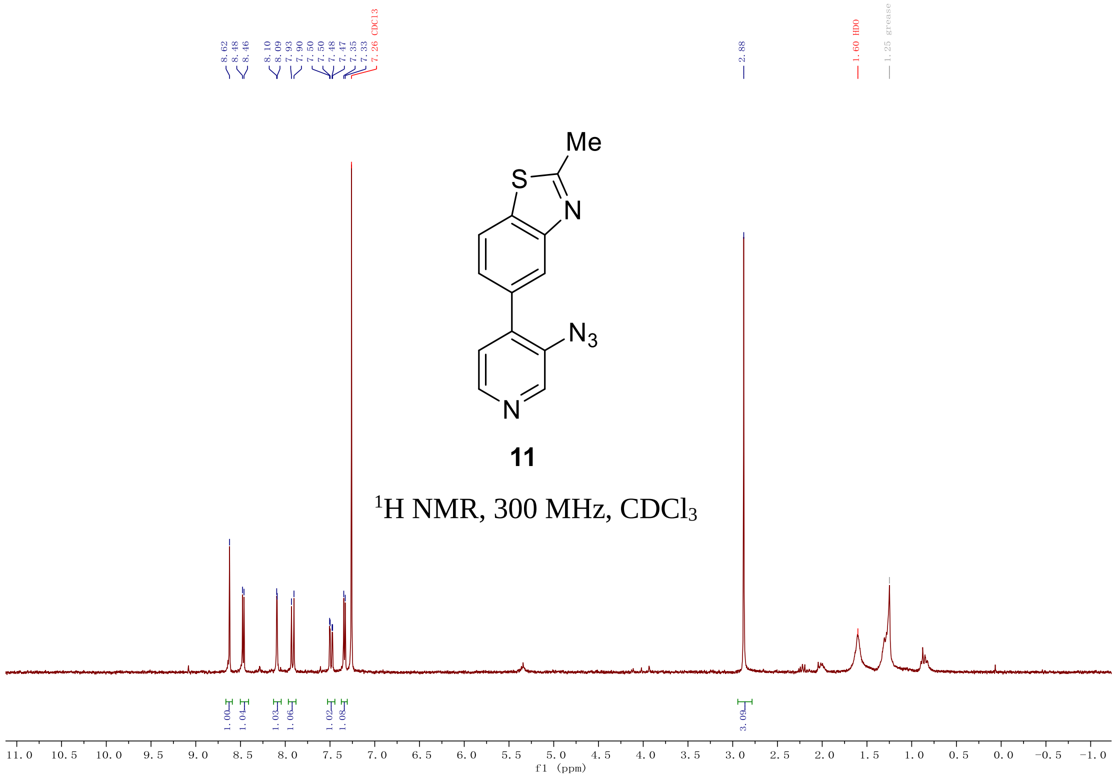
Me
S
N
N3
N
11
1H NMR, 300 MHz, CDCl3

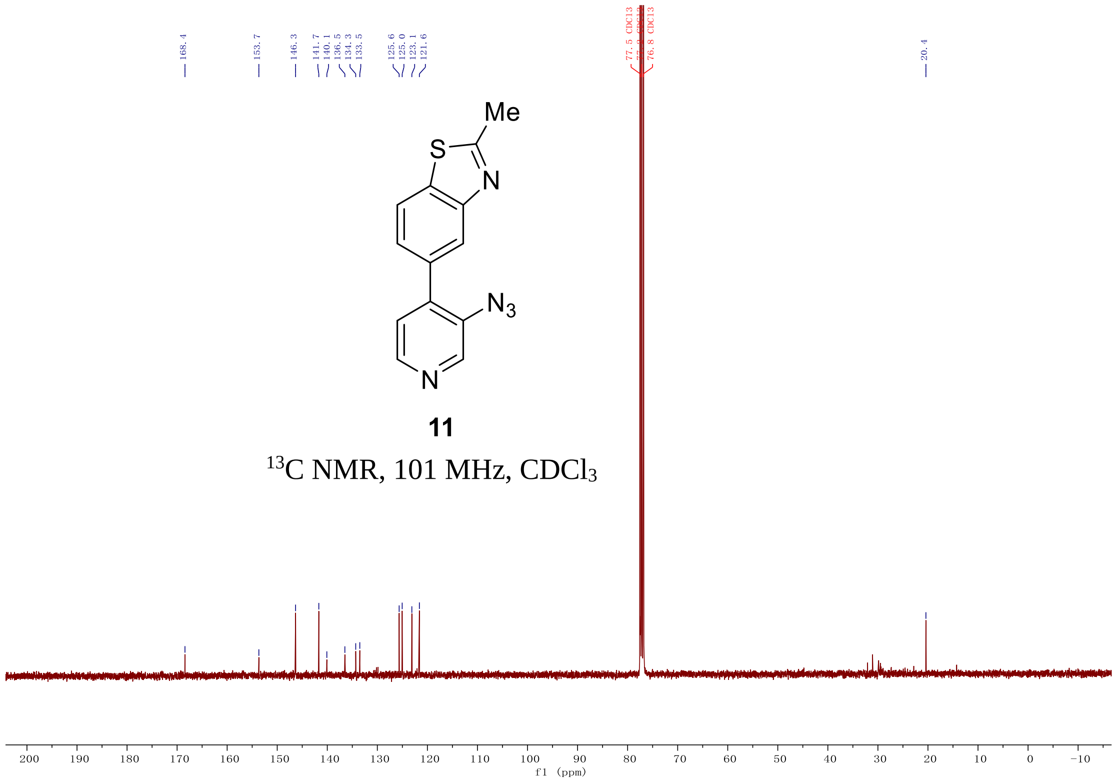
Me
S
N
N3
N
11
13C NMR, 101 MHz, CDCl3

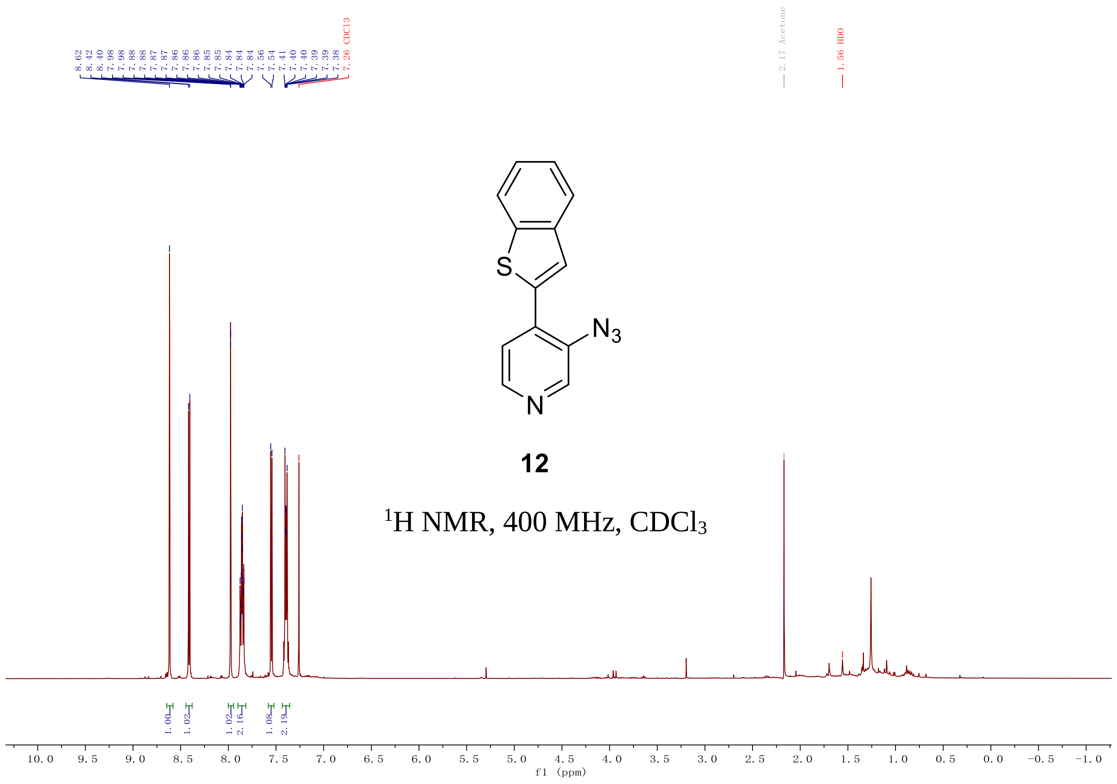

12

^{1}H NMR, 400 MHz, $CDCl_3$

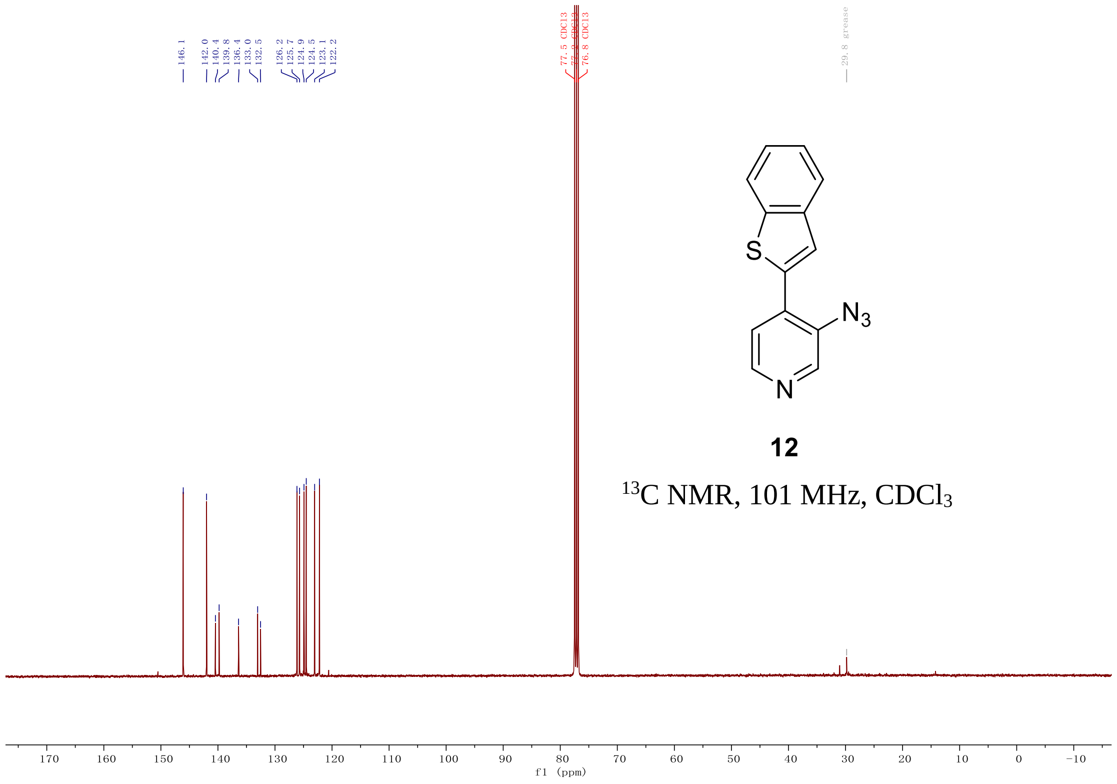

12

^{13}C NMR, 101 MHz, $CDCl_3$

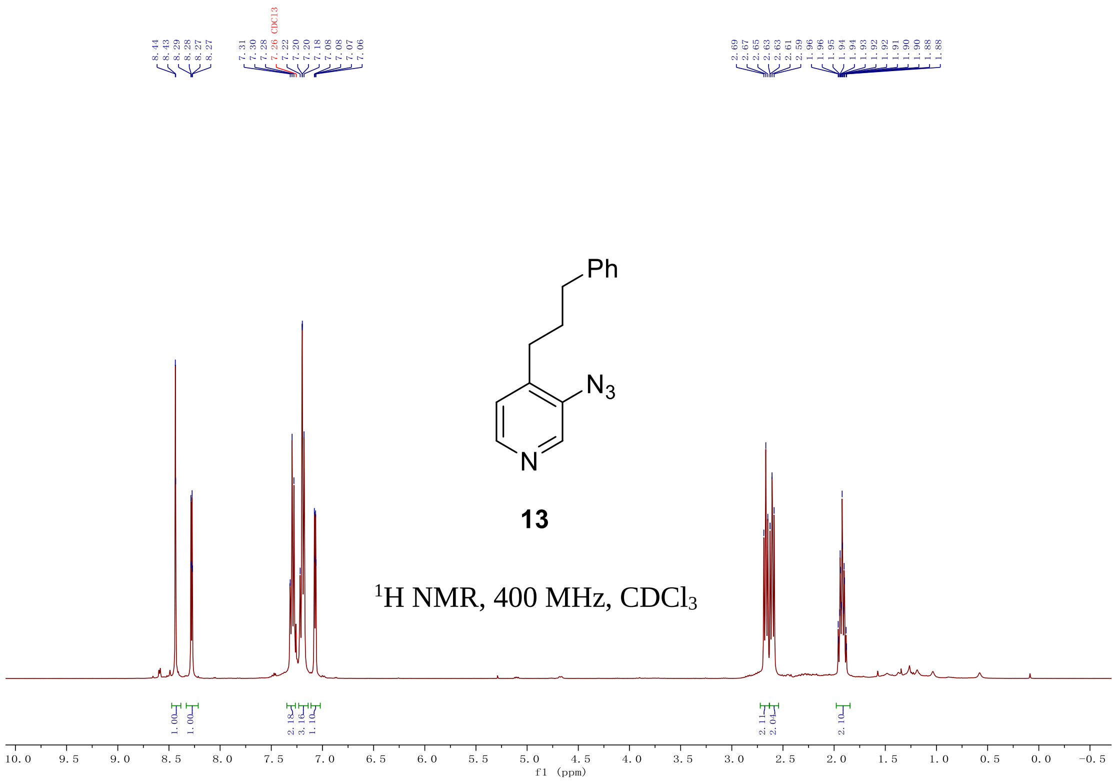
8.44
8.43
8.29
8.28
8.27
8.27
7.31
7.30
7.28
7.26 CDCl3
7.22
7.20
7.20
7.18
7.08
7.07
7.06
2.69
2.67
2.65
2.63
2.63
2.61
2.59
1.96
1.95
1.94
1.94
1.93
1.92
1.92
1.91
1.90
1.90
1.88
1.88
Ph
N3
N
13
1H NMR, 400 MHz, CDCl3
1.00
1.00
2.18
3.16
1.10
2.11
2.04
2.10
f1 (ppm)

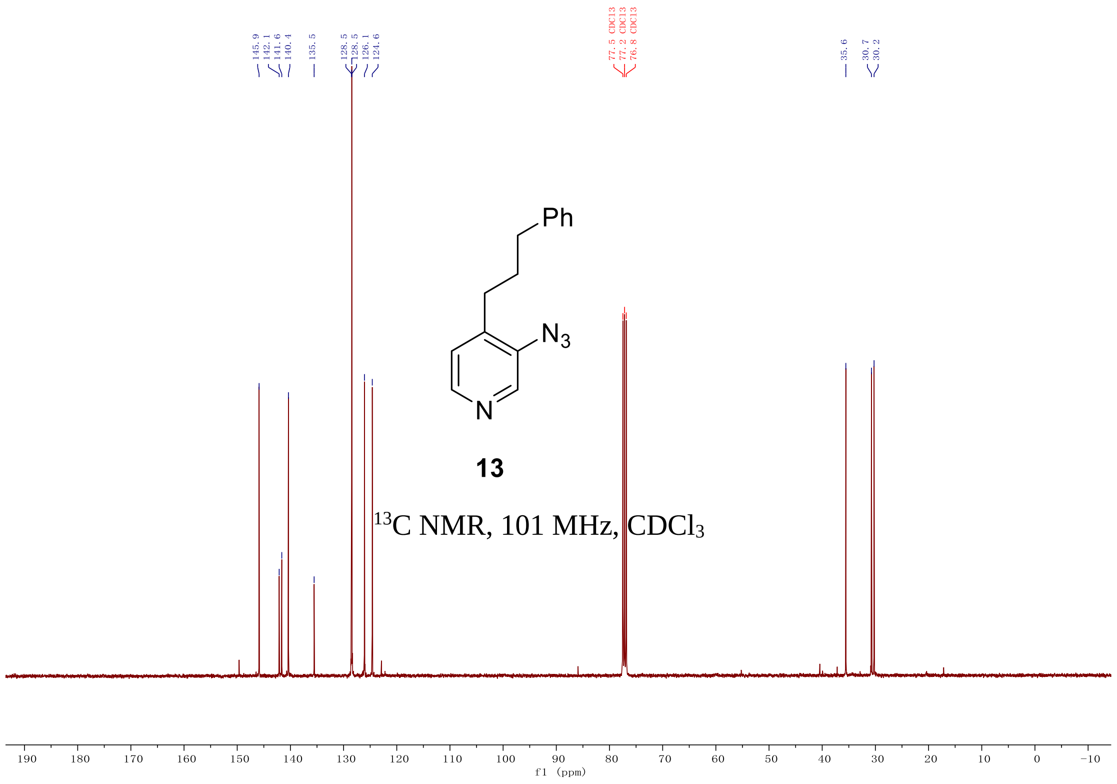
145.9
142.1
141.6
140.4
135.5
128.5
128.5
126.1
124.6
77.5 CDCl3
77.2 CDCl3
76.8 CDCl3
35.6
30.7
30.2
Ph
N3
N
13
13C NMR, 101 MHz, CDCl3
f1 (ppm)

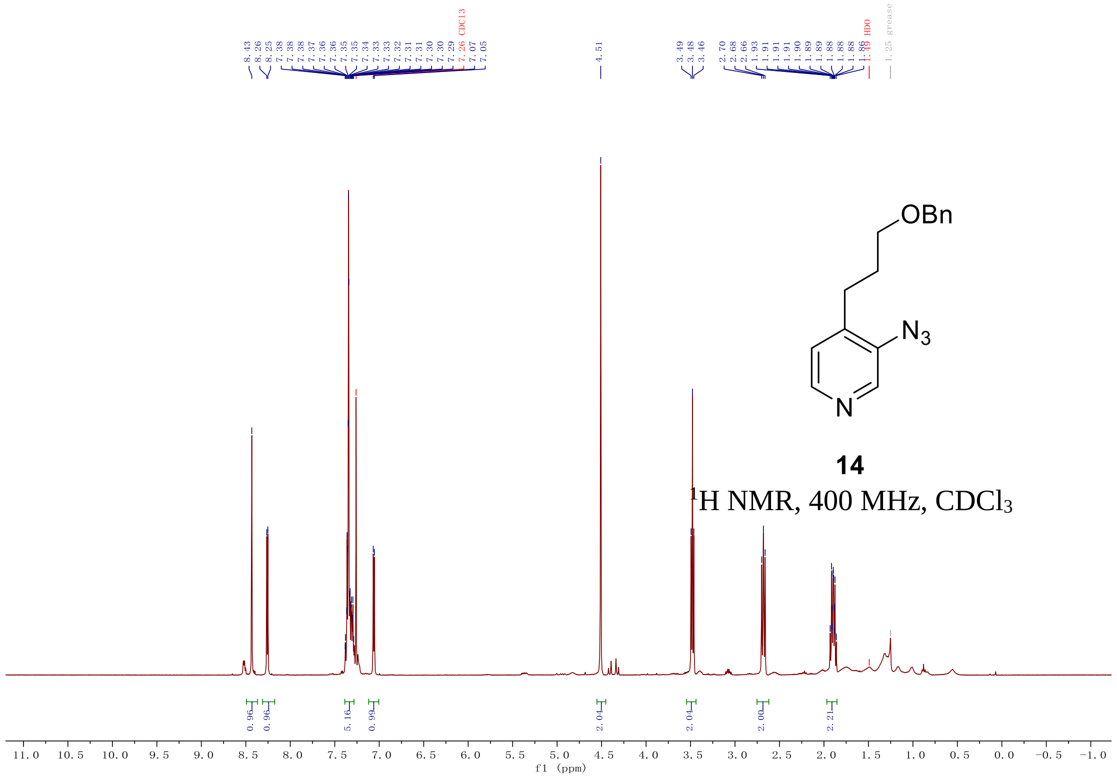
OBn
N3
N
14
1H NMR, 400 MHz, CDCl3
f1 (ppm)

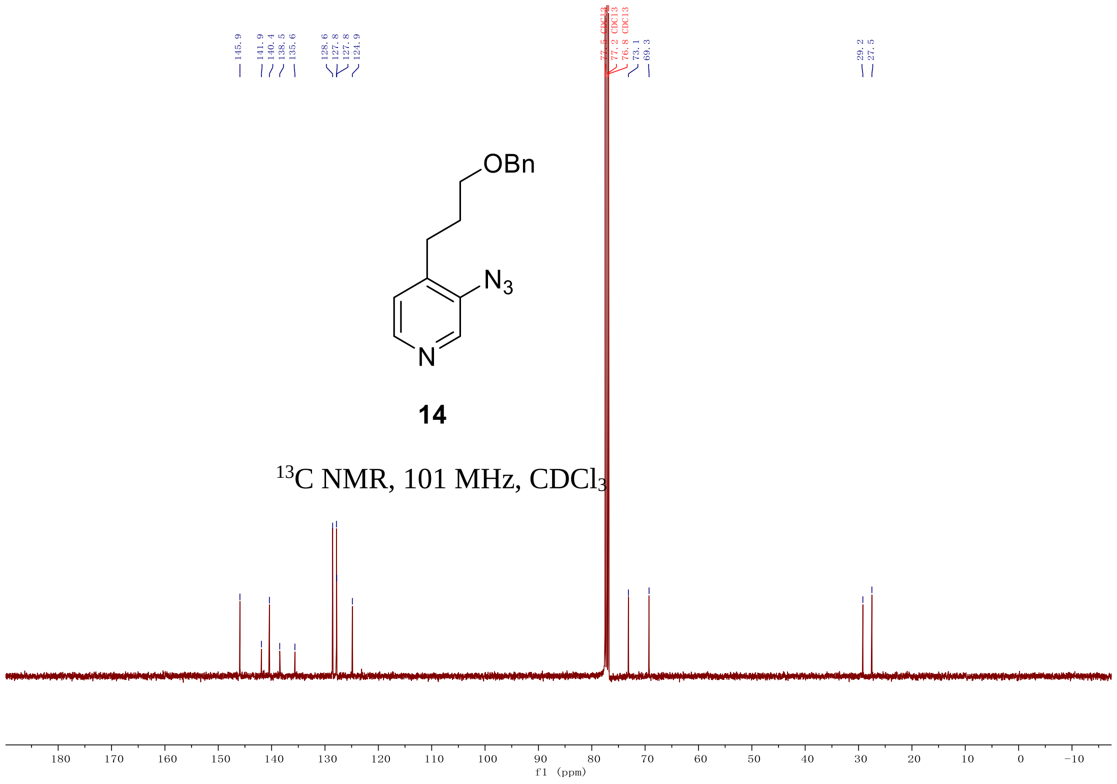
OBn
N3
N
14
13C NMR, 101 MHz, CDCl3
f1 (ppm)

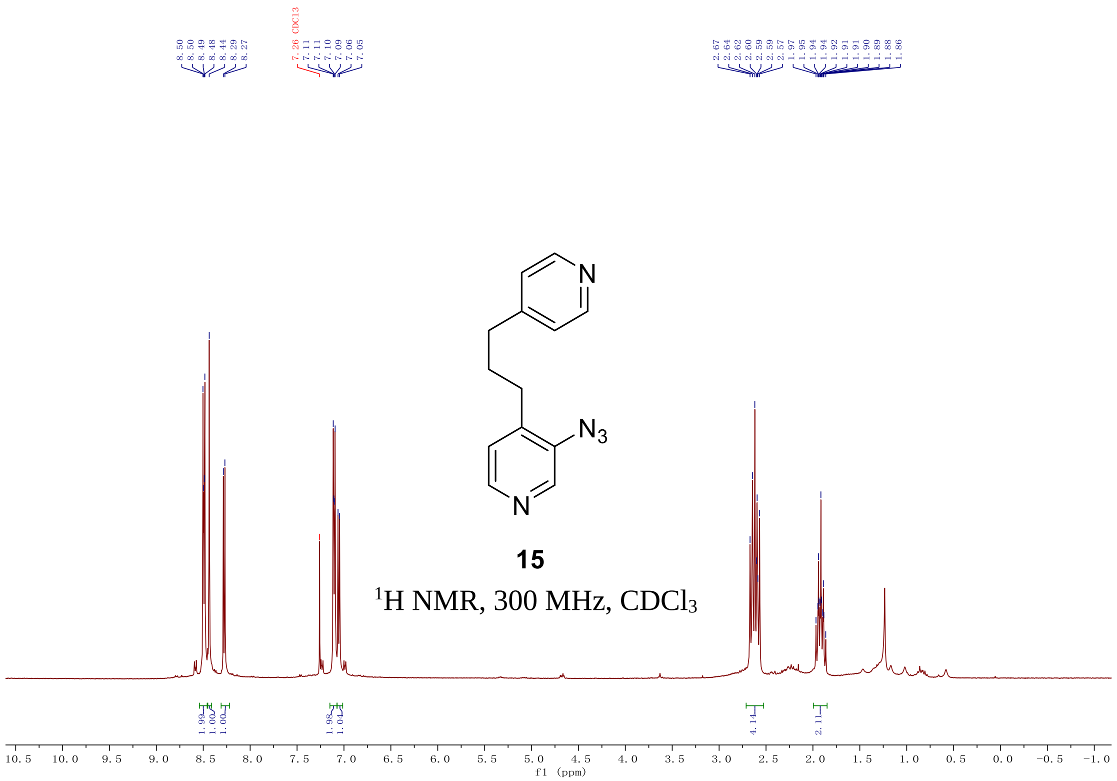

15

^{1}H NMR, 300 MHz, $CDCl_3$

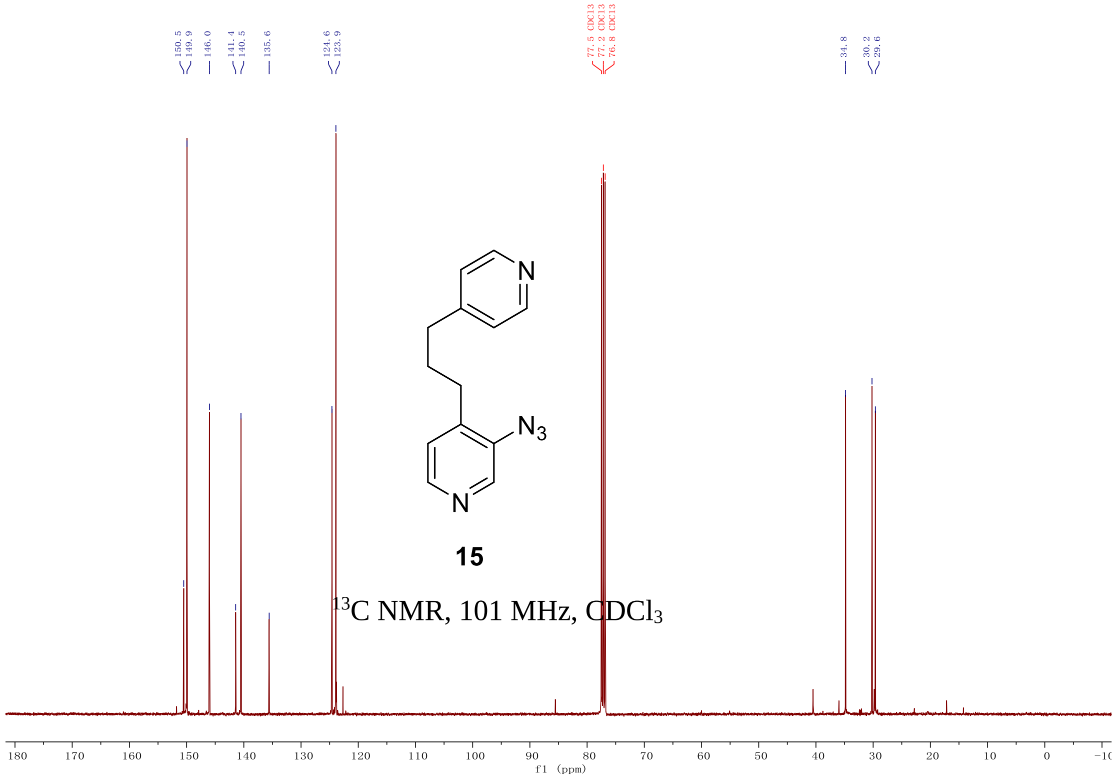

15

^{13}C NMR, 101 MHz, $CDCl_3$

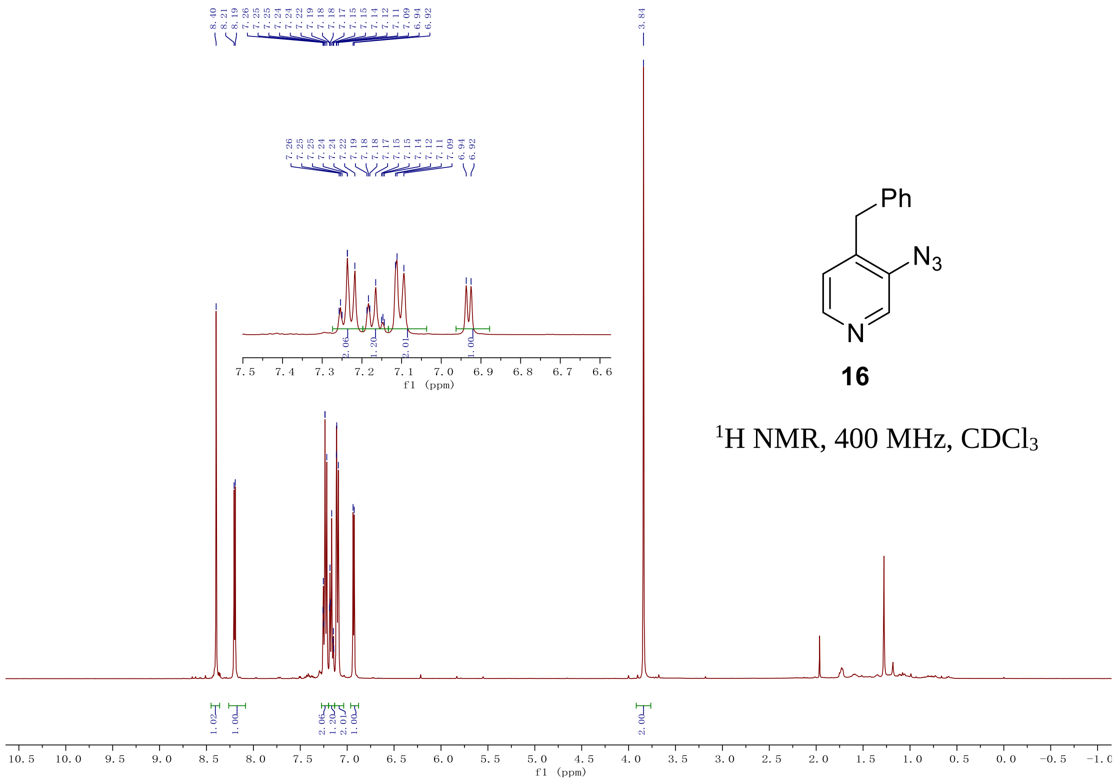
Ph
N3
N
16
1H NMR, 400 MHz, CDCl3
8.40
8.21
8.19
3.84
f1 (ppm)

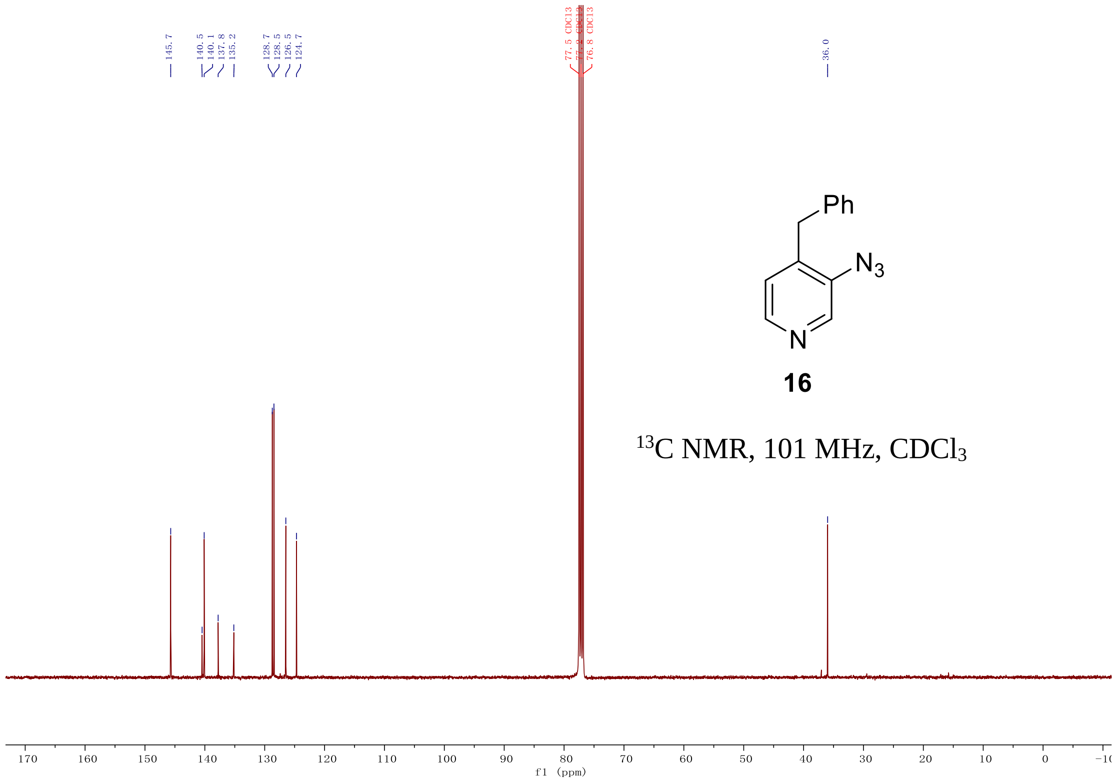
145.7
140.5
140.1
137.8
135.2
128.7
128.5
126.5
124.7
77.5 CDCl3
76.8 CDCl3
36.0
Ph
N3
N
16
13C NMR, 101 MHz, CDCl3
f1 (ppm)

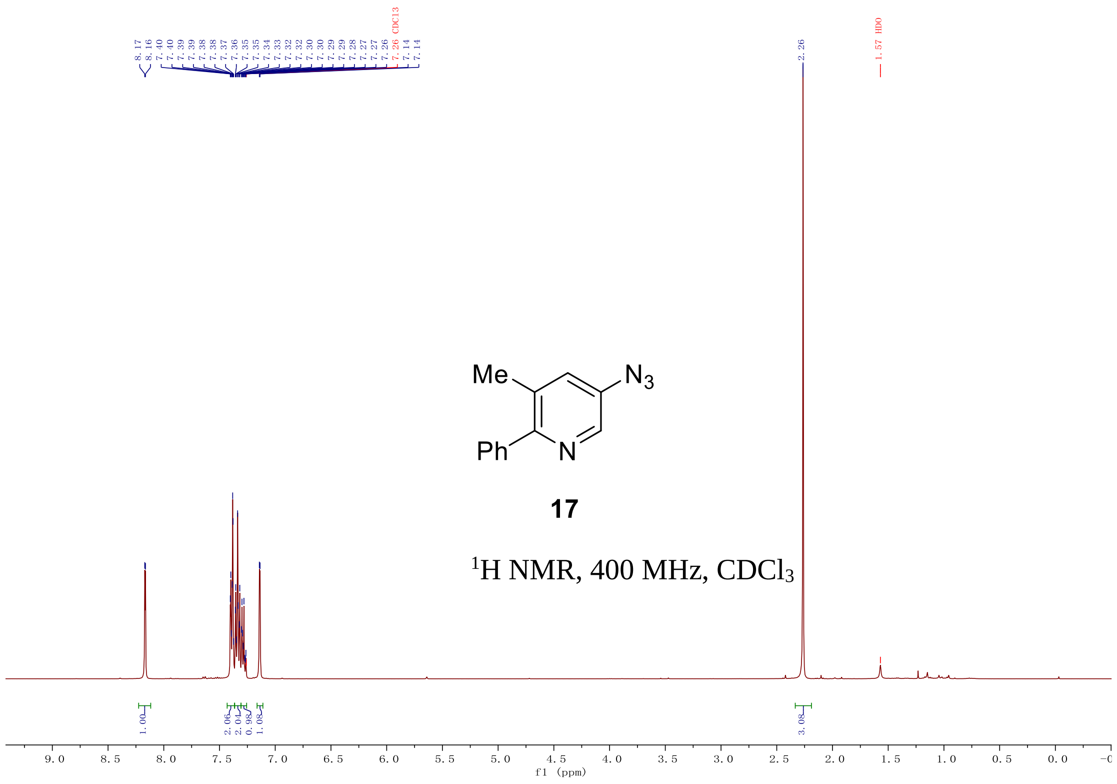

17

^{1}H NMR, 400 MHz, $CDCl_3$

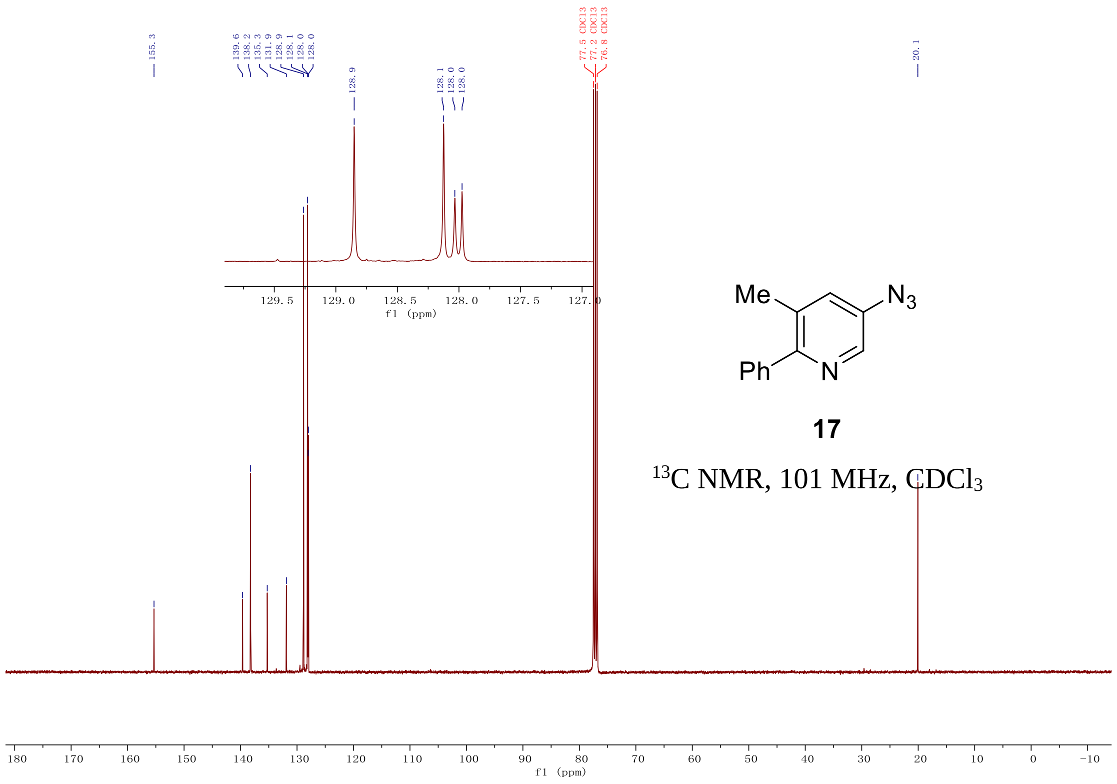

17

^{13}C NMR, 101 MHz, $CDCl_3$

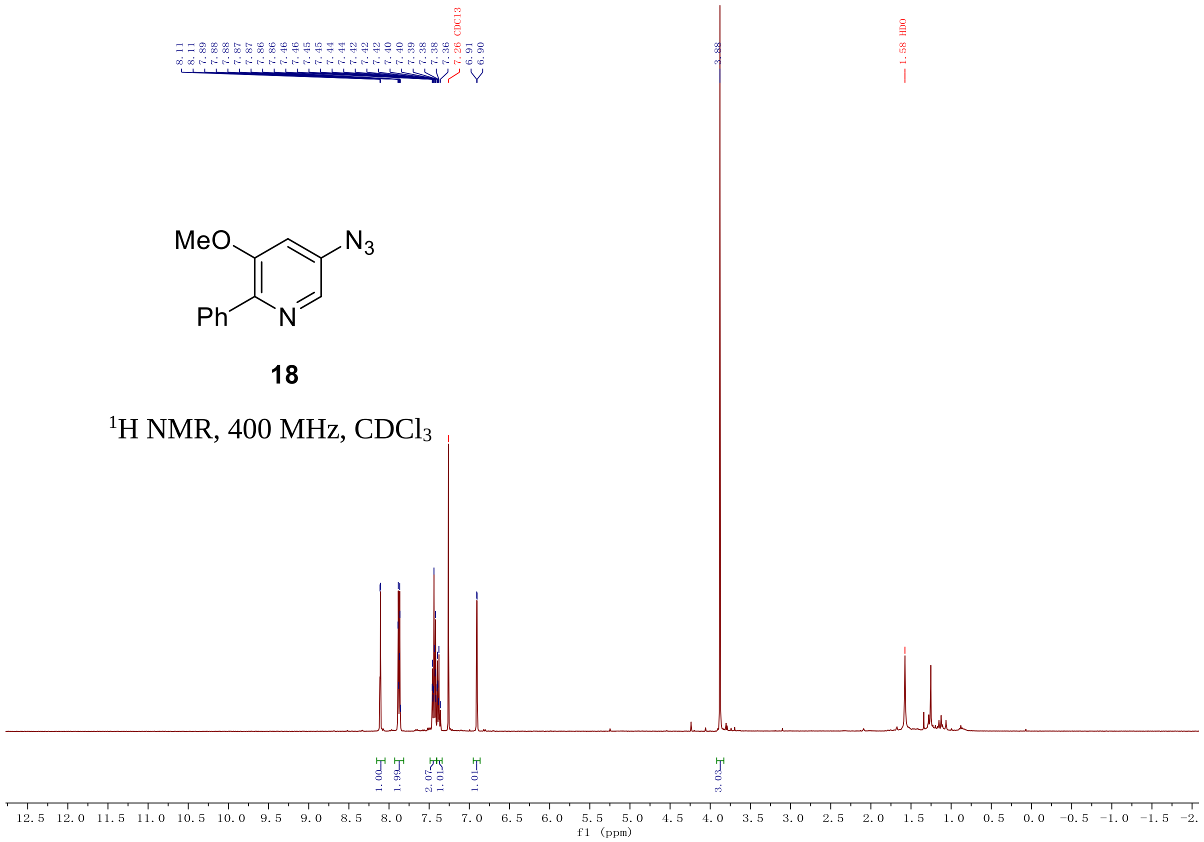

18

^{1}H NMR, 400 MHz, $CDCl_3$

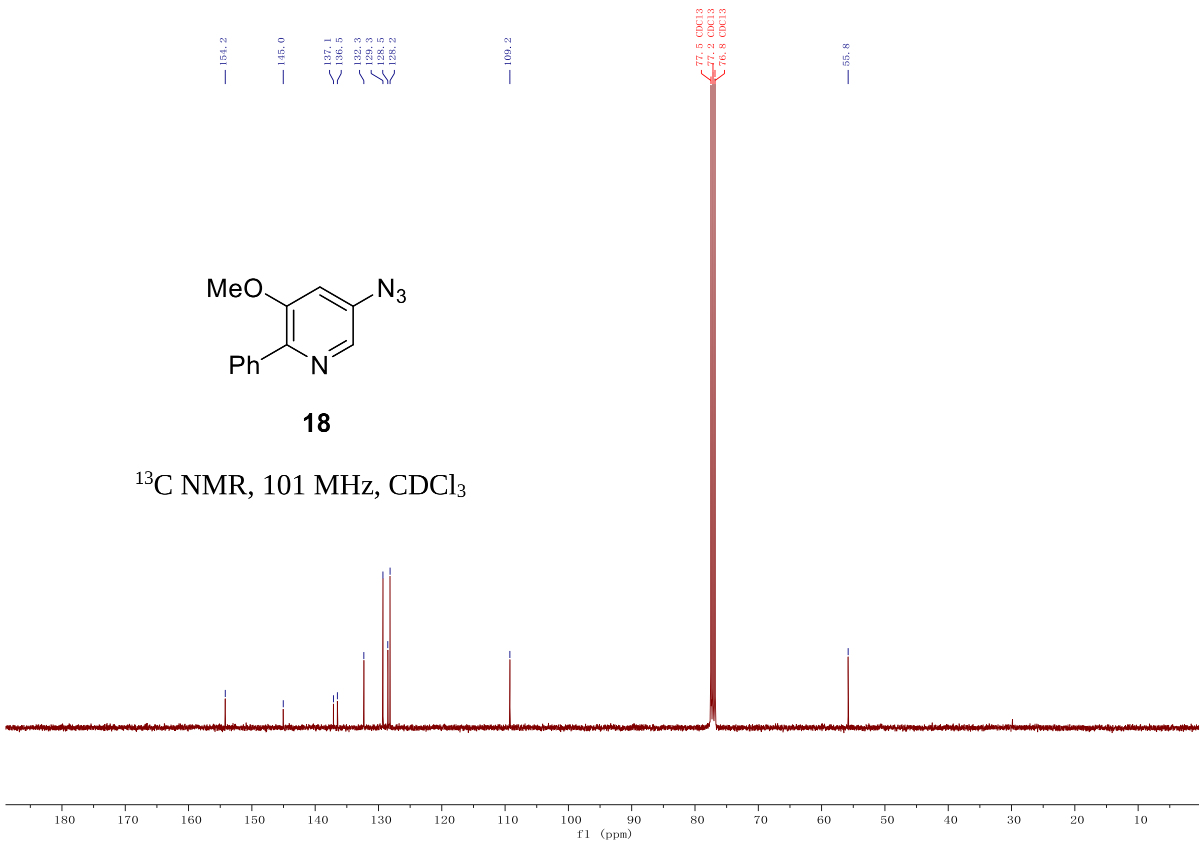

18

^{13}C NMR, 101 MHz, $CDCl_3$

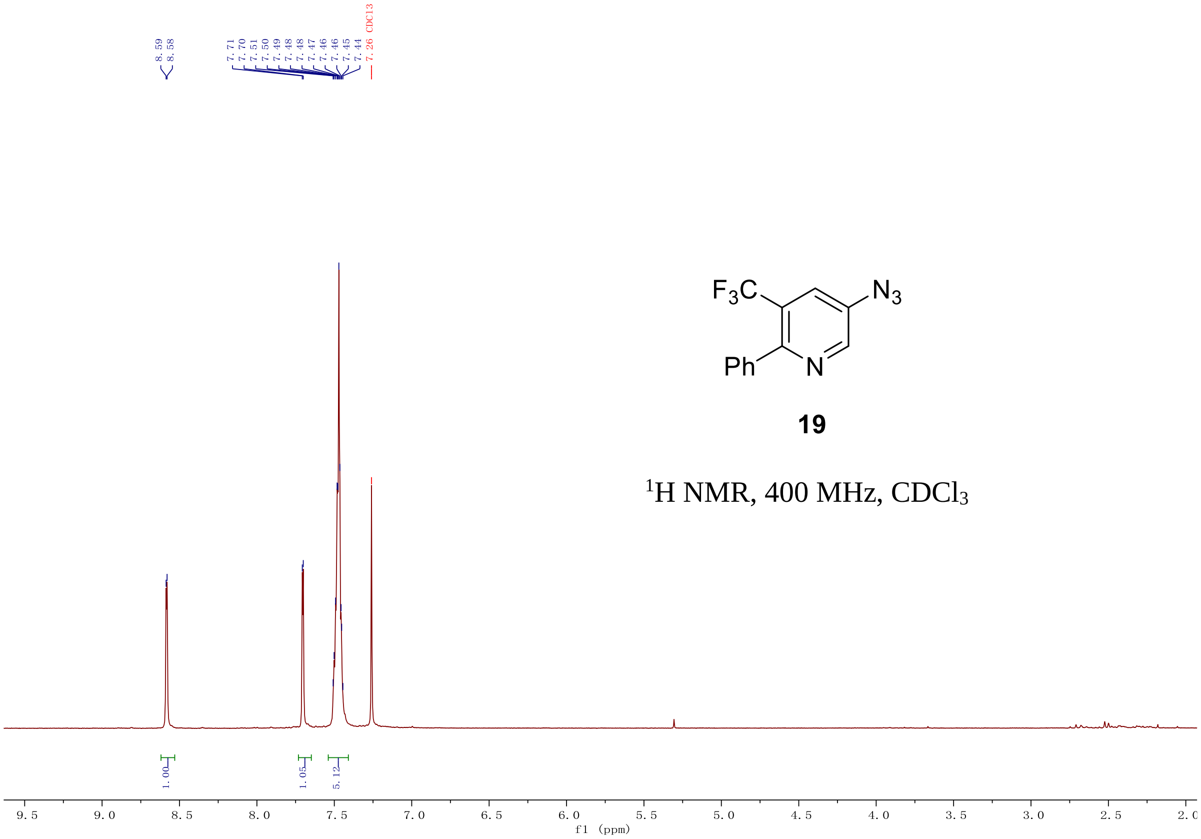
8.59
8.58
7.71
7.70
7.51
7.50
7.49
7.48
7.48
7.47
7.46
7.45
7.44
7.26 CDCl3
F_3C
N_3
Ph
N
19
^{1}H NMR, 400 MHz, $CDCl_3$
1.00
1.05
5.12
9.5
9.0
8.5
8.0
7.5
7.0
6.5
6.0
5.5
5.0
4.5
4.0
3.5
3.0
2.5
f1 (ppm)

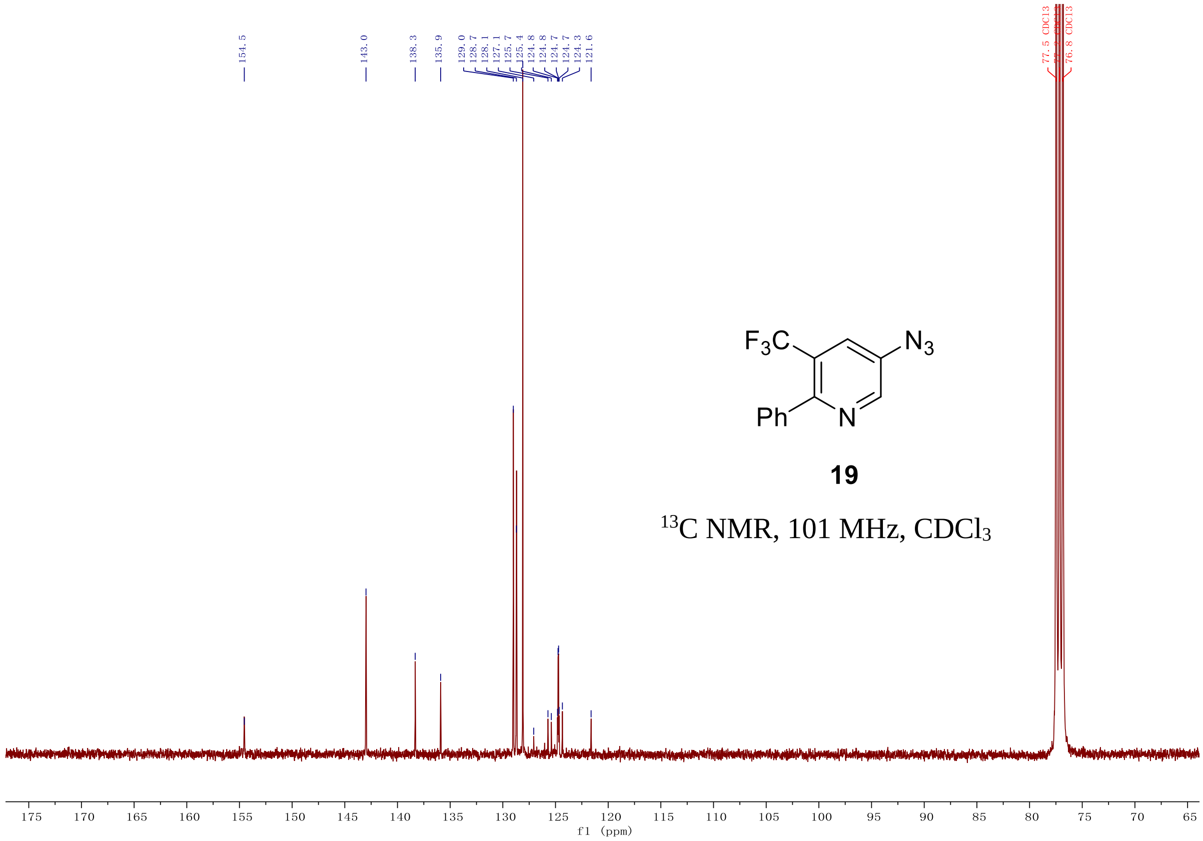
154.5
143.0
138.3
135.9
129.0
128.7
128.1
127.1
125.7
125.4
124.8
124.8
124.7
124.7
124.3
121.6
77.5 CDCl3
76.8 CDCl3
F_3C
N_3
Ph
N
19
^{13}C NMR, 101 MHz, $CDCl_3$
175
170
165
160
155
150
145
140
135
130
125
120
115
110
105
100
95
90
85
80
75
70
65
f1 (ppm)

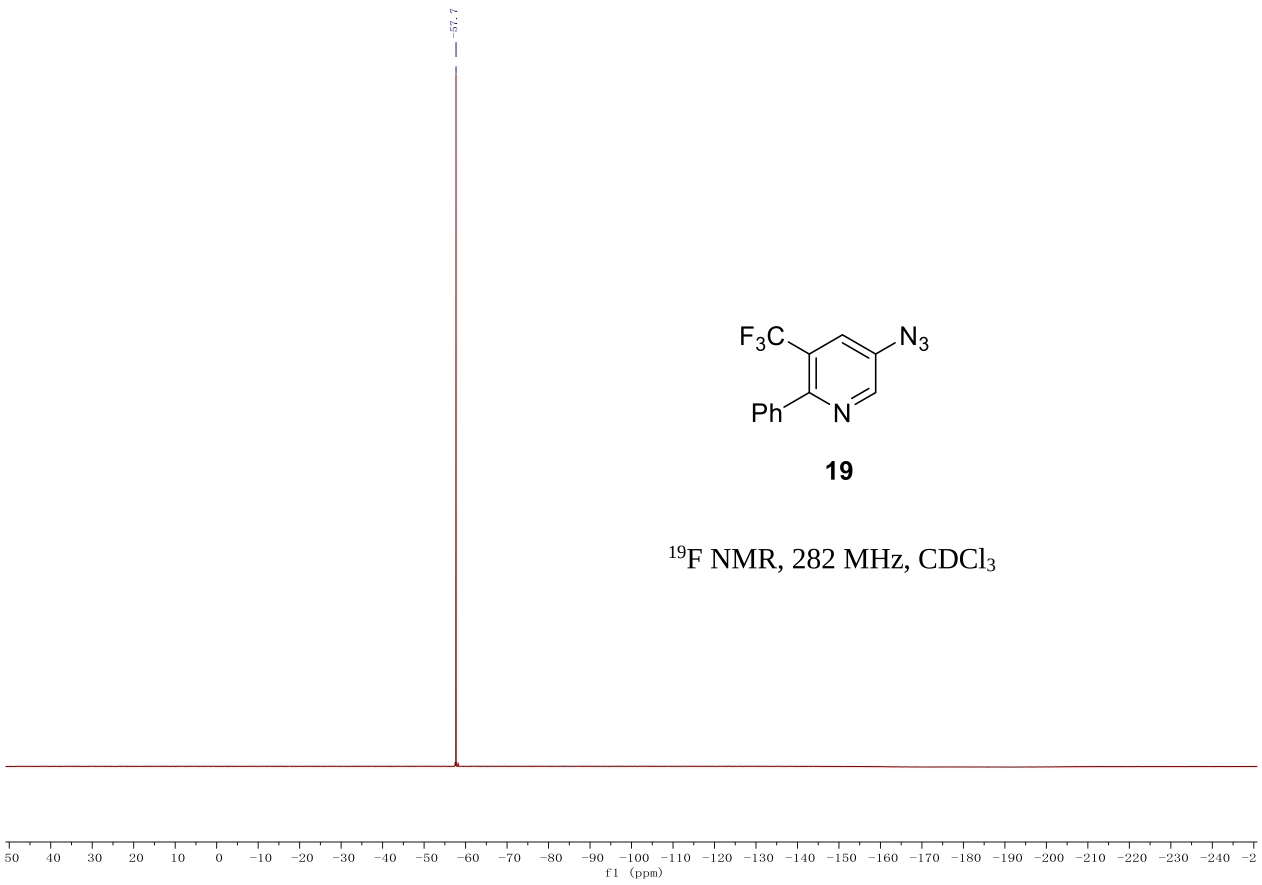

-57.7
F3C
N3
Ph
N
19
19F NMR, 282 MHz, CDCl3
50 40 30 20 10 0 -10 -20 -30 -40 -50 -60 -70 -80 -90 -100 -110 -120 -130 -140 -150 -160 -170 -180 -190 -200 -210 -220 -230 -240 -2
f1 (ppm)

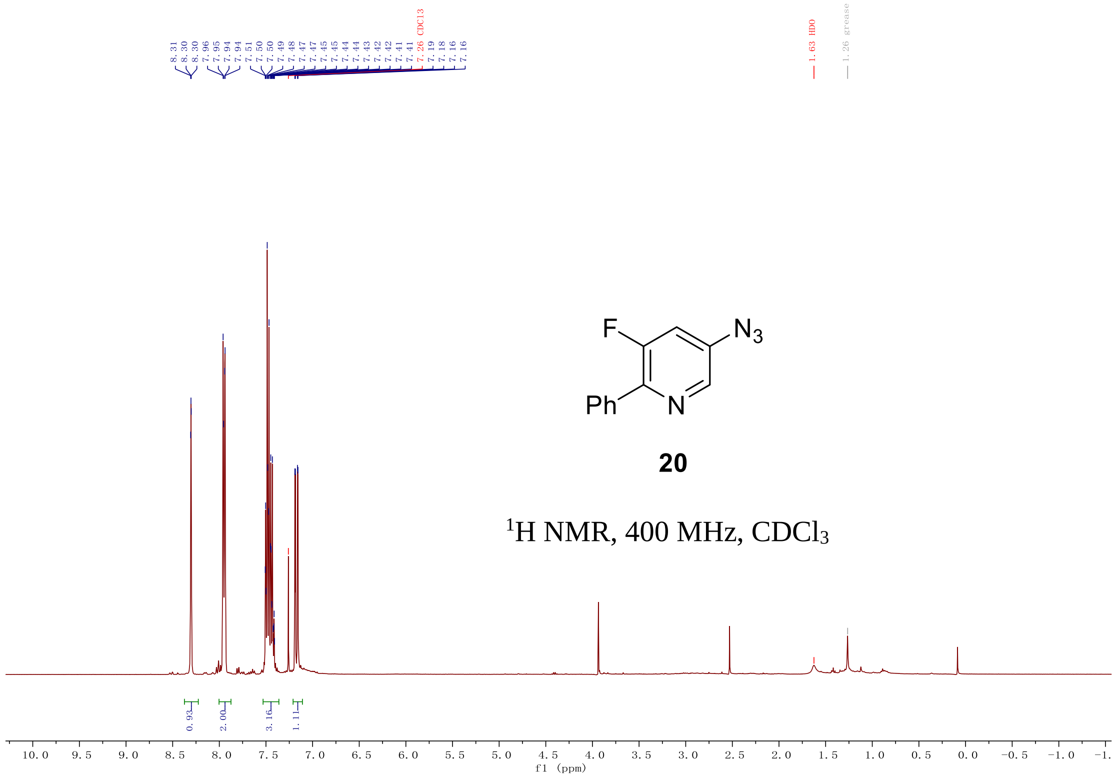
8.31
8.30
8.30
7.96
7.95
7.94
7.94
7.51
7.50
7.50
7.49
7.48
7.47
7.47
7.45
7.45
7.44
7.44
7.43
7.42
7.42
7.41
7.41
7.26 CDCl3
7.19
7.18
7.16
7.16
1.63 HDO
1.26 grease
F
N3
Ph
N
20
1H NMR, 400 MHz, CDCl3
0.93
2.00
3.16
1.11
f1 (ppm)

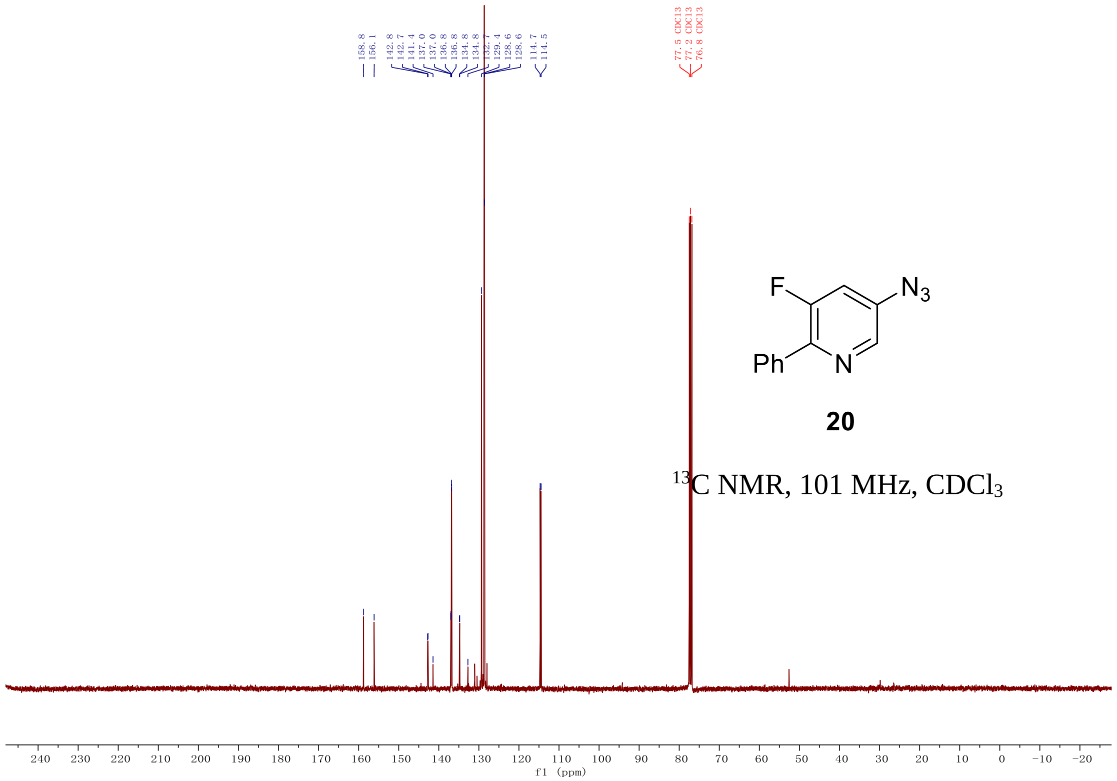
158.8
156.1
142.8
142.7
141.4
137.0
137.0
136.8
136.8
134.8
134.8
132.7
129.4
128.6
128.6
114.7
114.5
77.5 CDCl3
77.2 CDCl3
76.8 CDCl3
F
N3
Ph
N
20
13C NMR, 101 MHz, CDCl3
f1 (ppm)

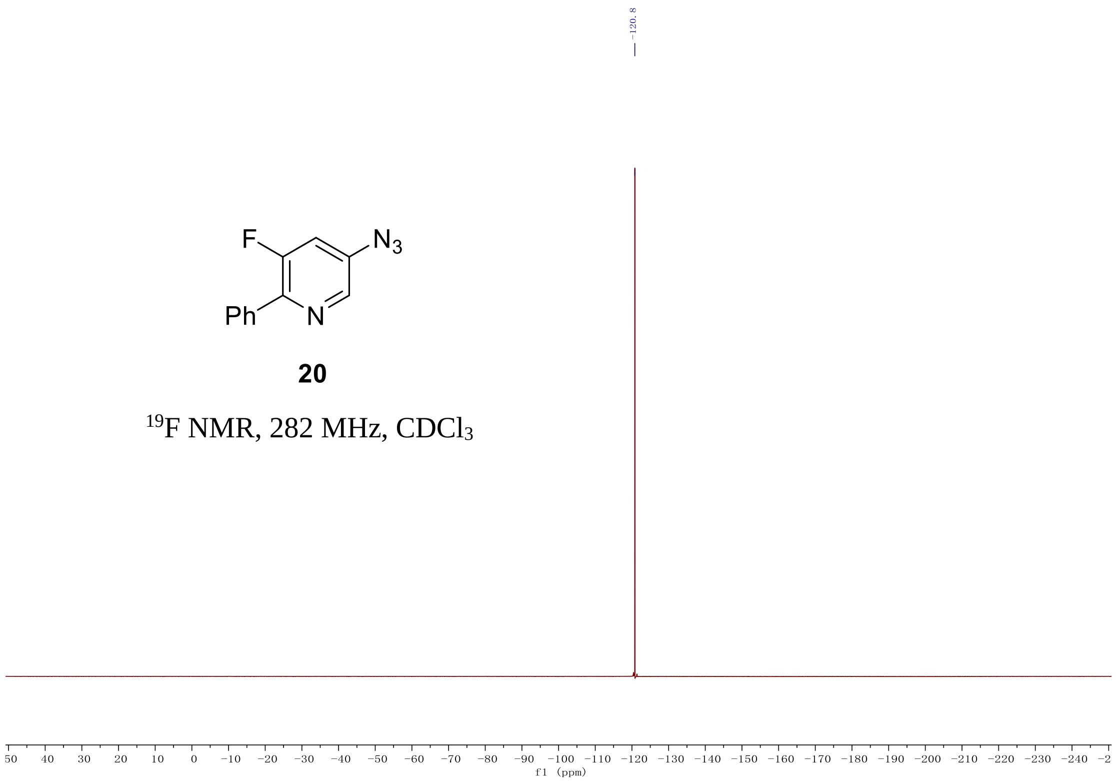

20

^{19}F NMR, 282 MHz, $CDCl_3$

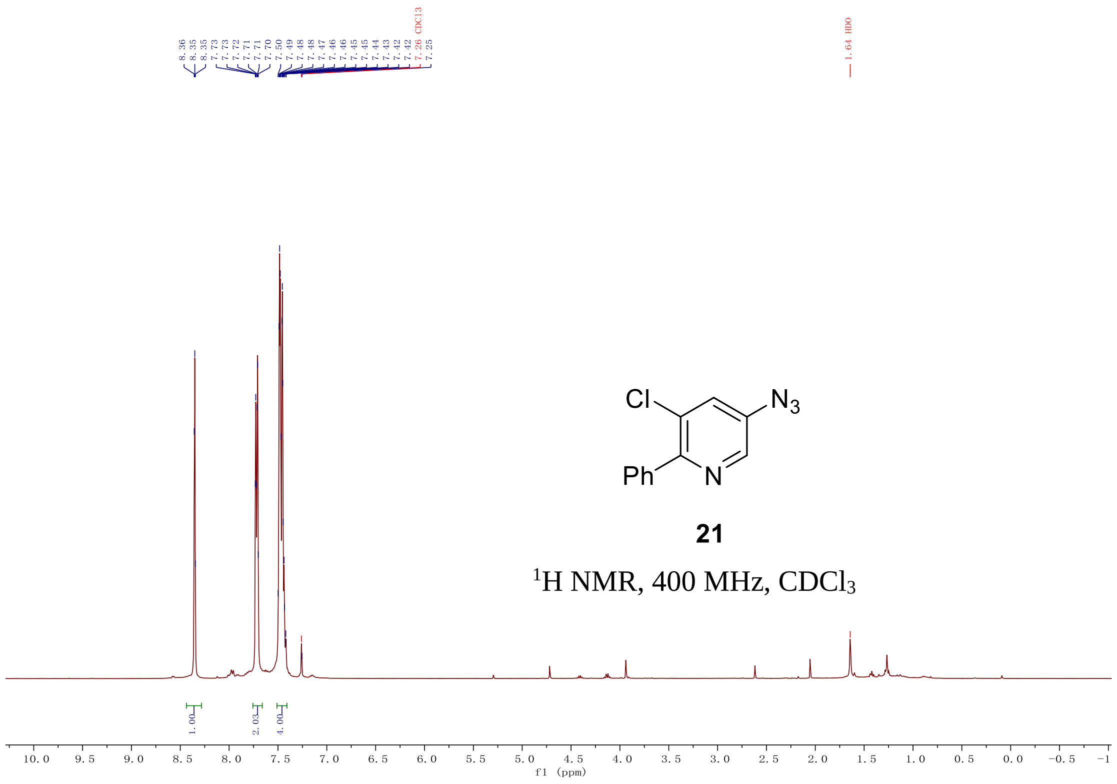

21

^{1}H NMR, 400 MHz, $CDCl_3$

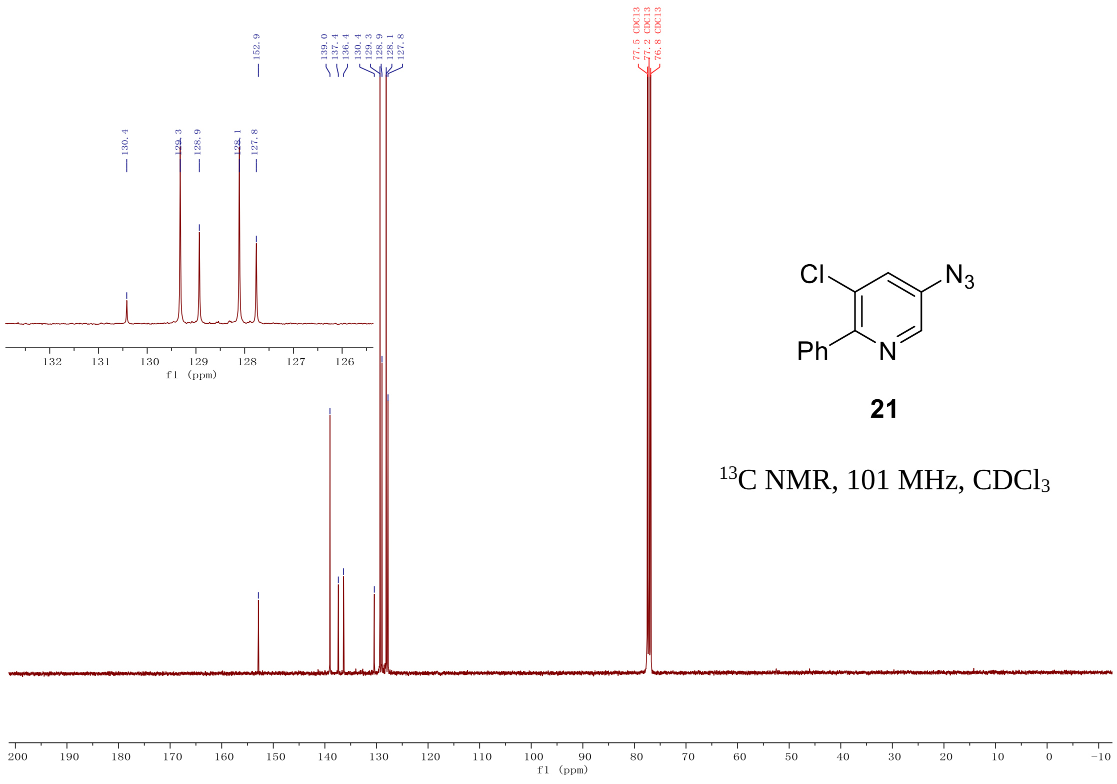

21

^{13}C NMR, 101 MHz, $CDCl_3$

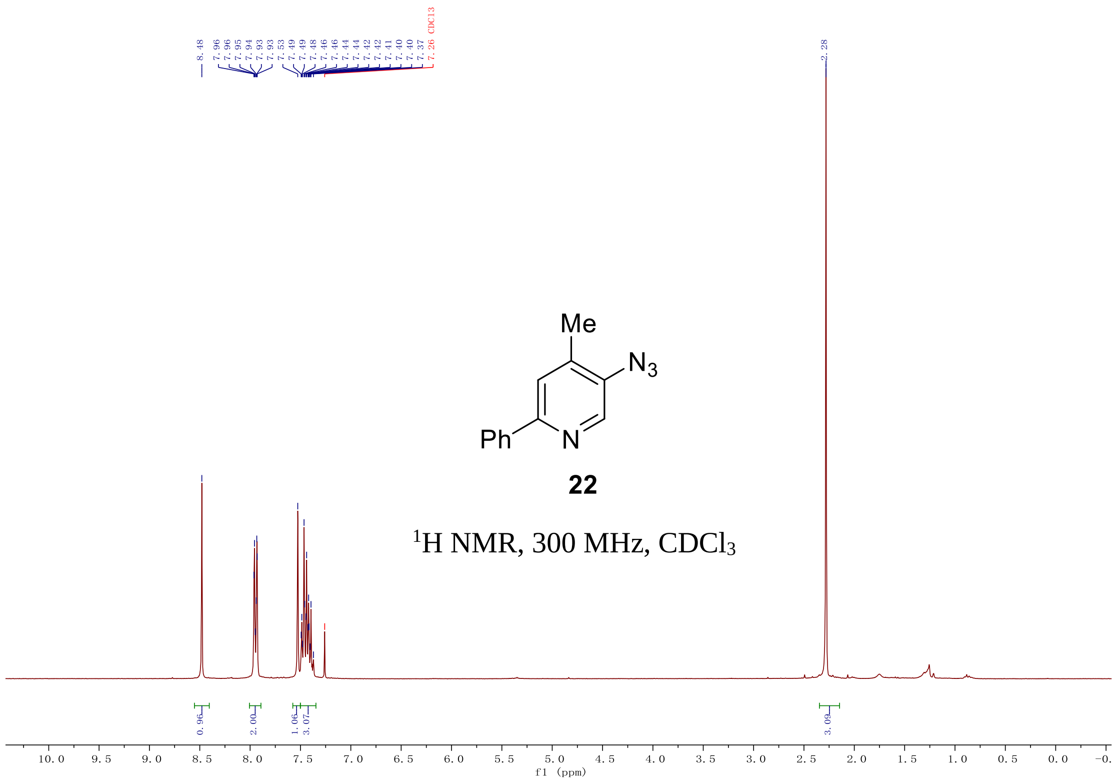

^{1}H NMR, 300 MHz, $CDCl_3$

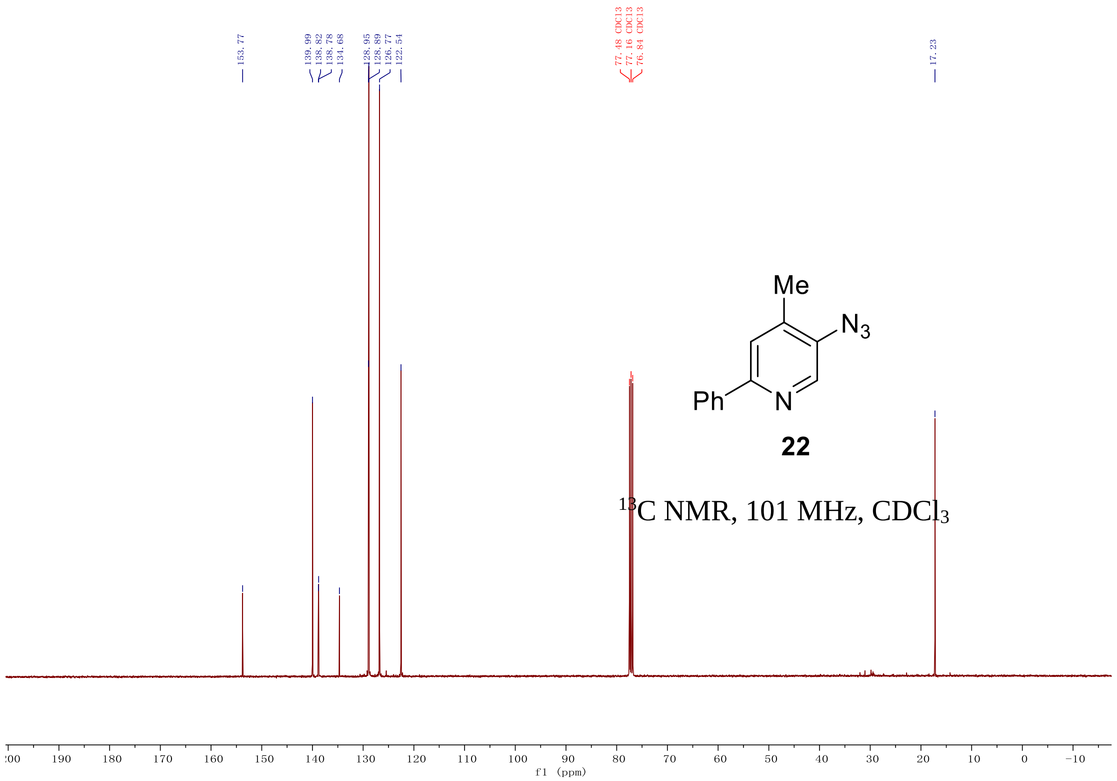

^{13}C NMR, 101 MHz, $CDCl_3$

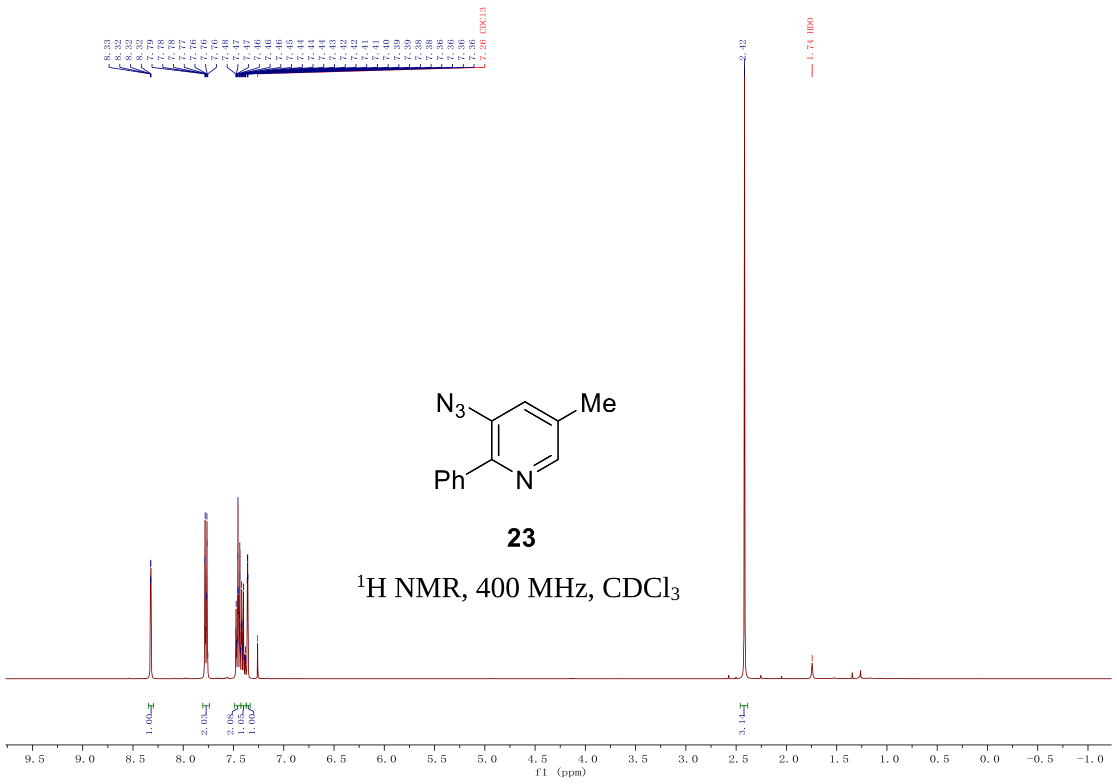

23

^{1}H NMR, 400 MHz, $CDCl_3$

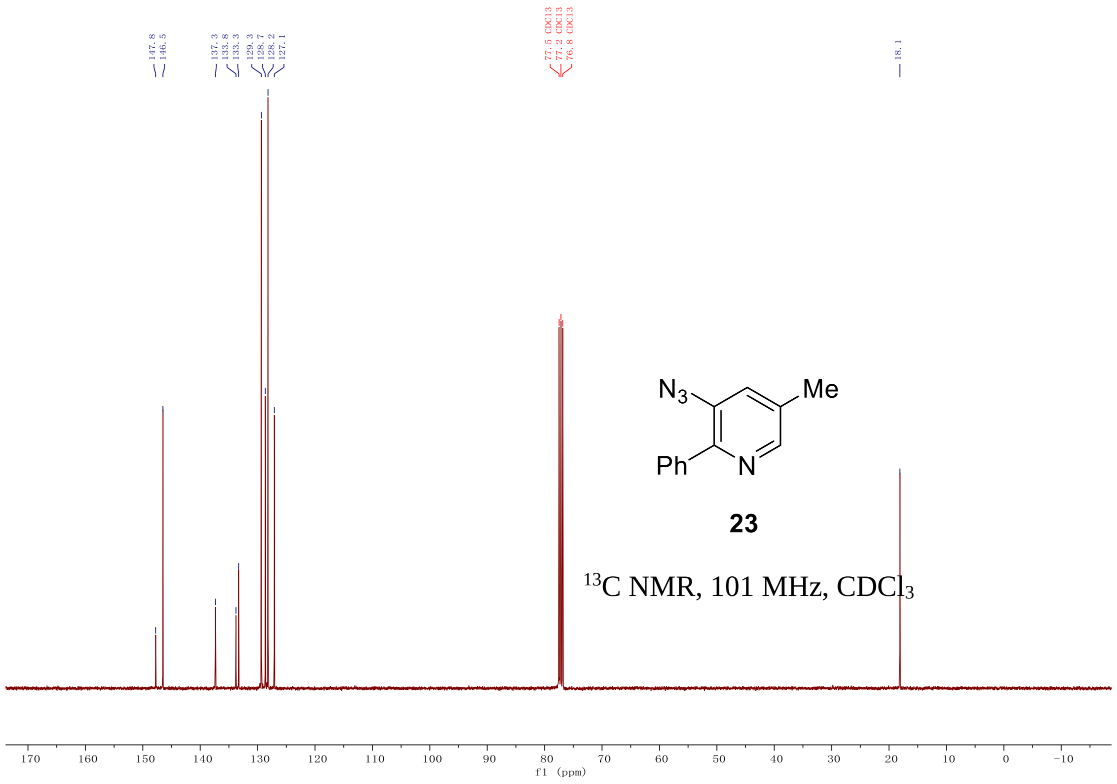

23

^{13}C NMR, 101 MHz, $CDCl_3$

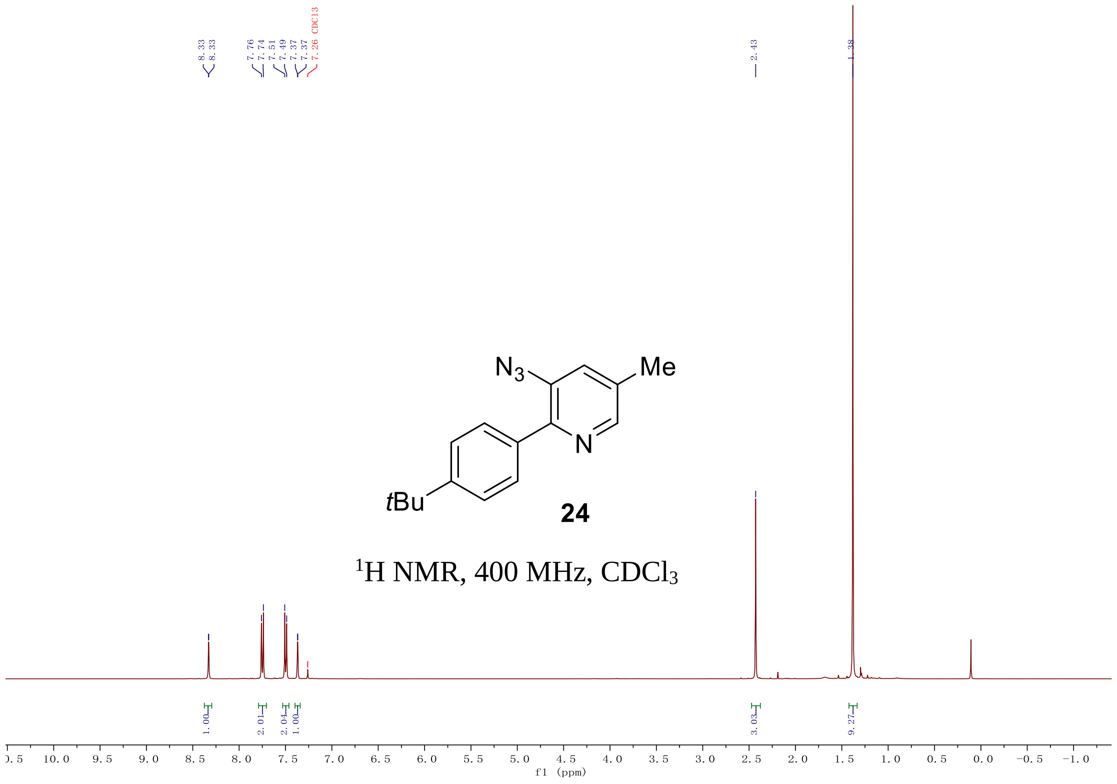

24

^{1}H NMR, 400 MHz, $CDCl_3$

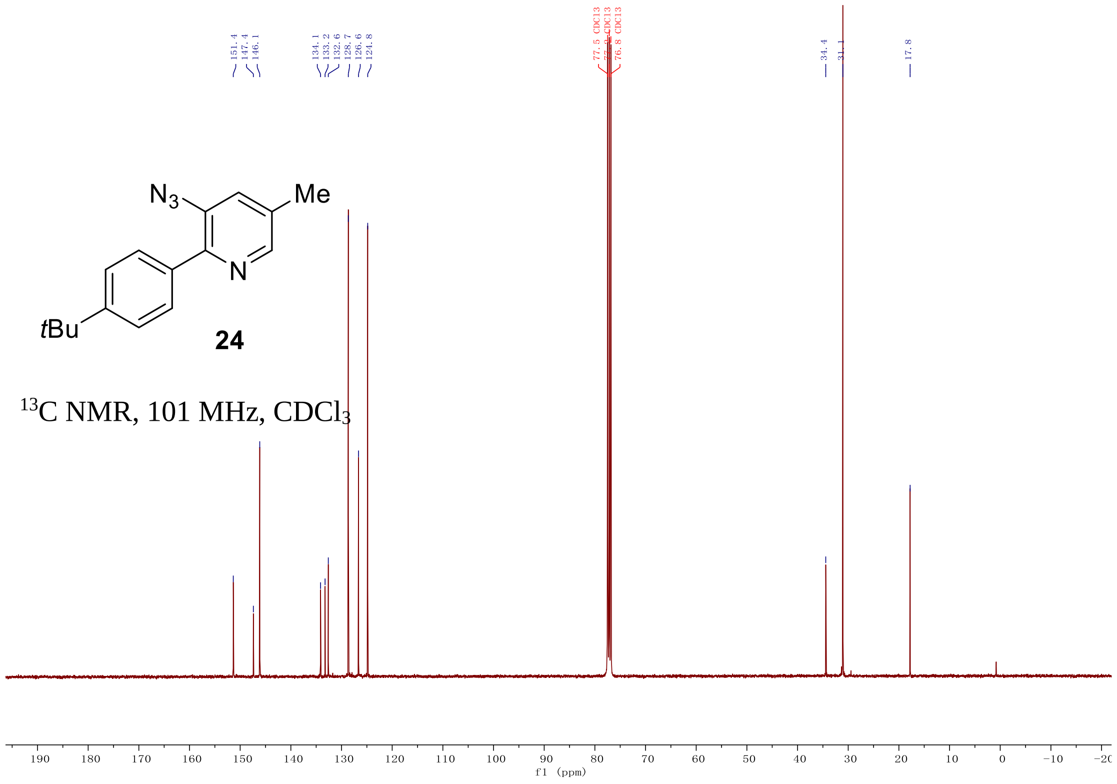

24

^{13}C NMR, 101 MHz, $CDCl_3$

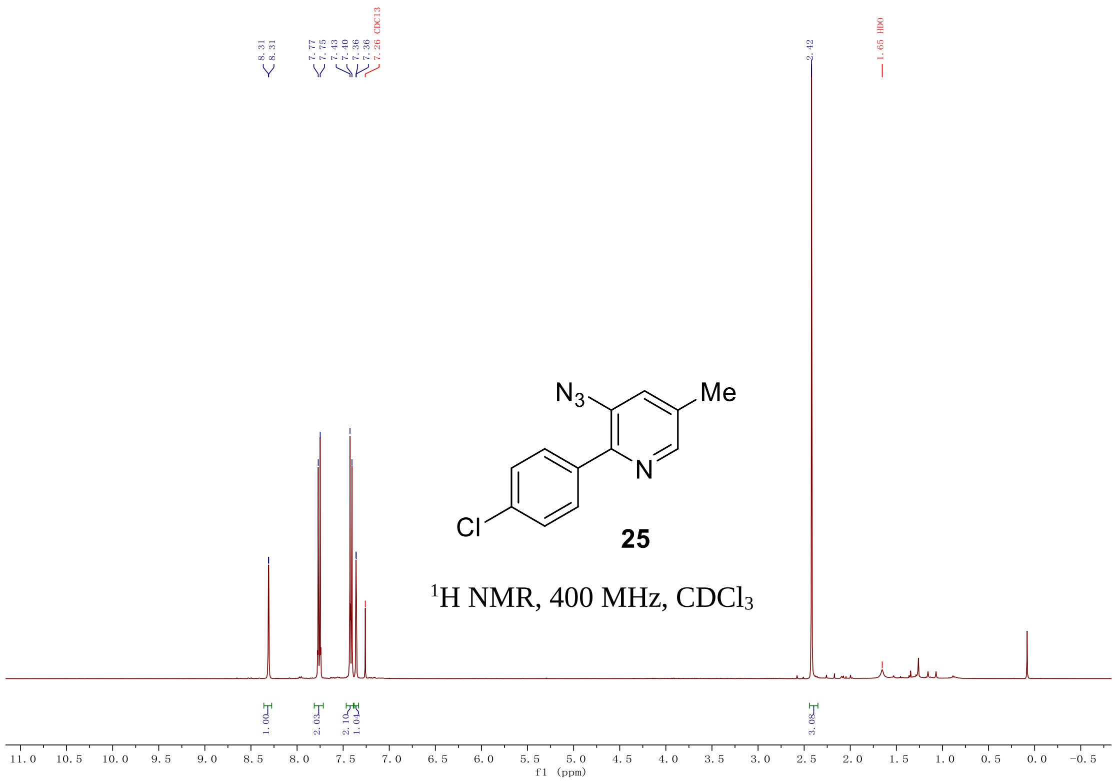
8.31
8.31
7.77
7.75
7.43
7.40
7.36
7.36
7.26 CDCl3
2.42
1.65 HDO
N3
Me
N
Cl
25
1H NMR, 400 MHz, CDCl3
1.00
2.03
2.10
1.04
3.08
f1 (ppm)

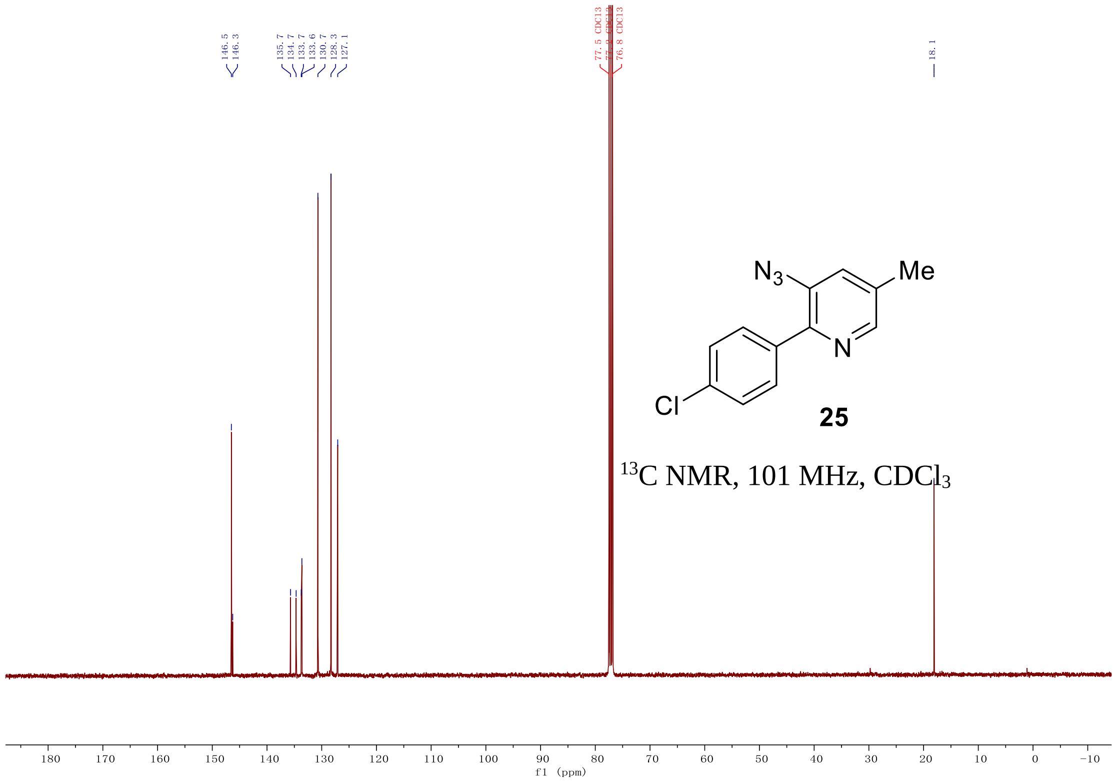
146.5
146.3
135.7
134.7
133.7
133.6
130.7
128.3
127.1
77.5 CDCl3
76.8 CDCl3
18.1
N3
Me
N
Cl
25
13C NMR, 101 MHz, CDCl3
f1 (ppm)

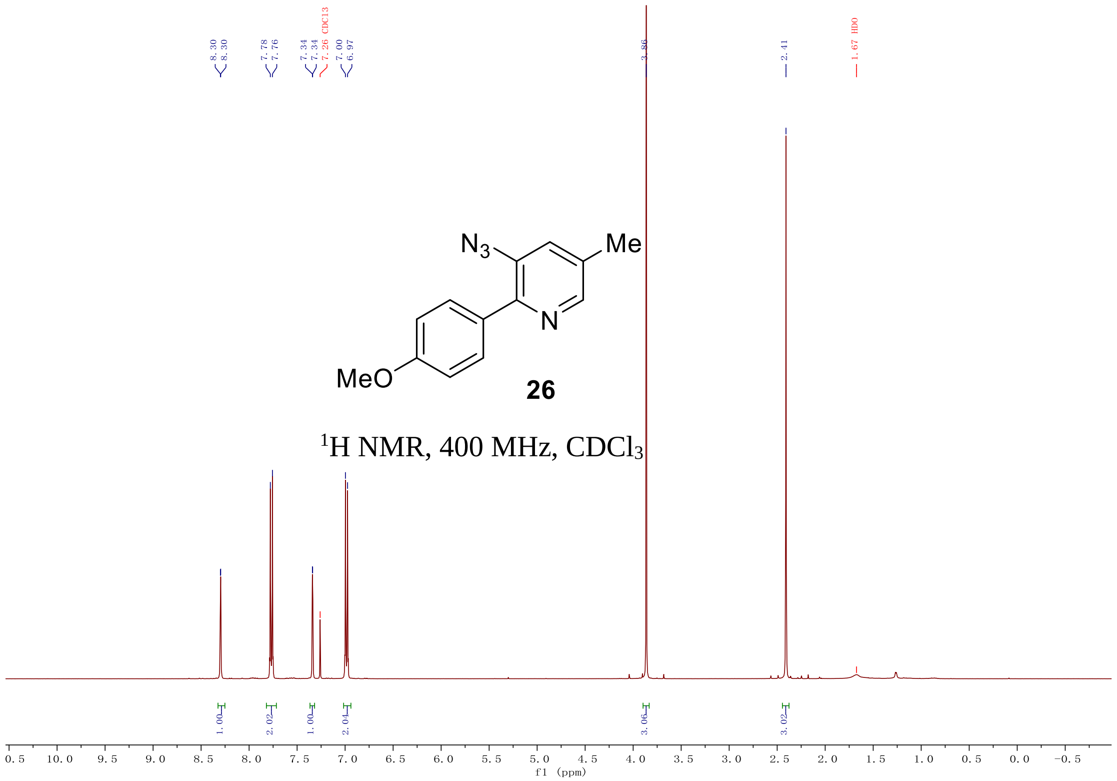
8.30
8.30
7.78
7.76
7.34
7.34
7.26 CDCl3
7.00
6.97
3.86
2.41
1.67 HDO
N3
Me
N
MeO
26
1H NMR, 400 MHz, CDCl3
1.00
2.02
1.00
2.04
3.06
3.02
f1 (ppm)

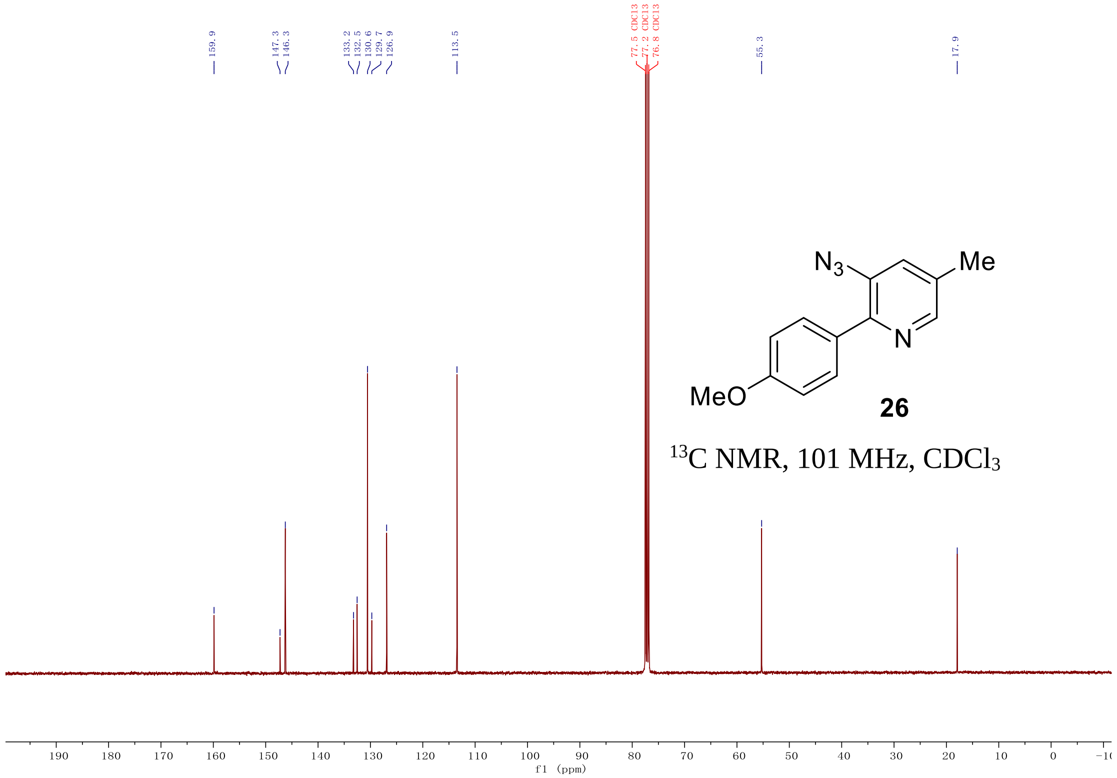
159.9
147.3
146.3
133.2
132.5
130.6
129.7
126.9
113.5
77.5 CDCl3
77.2 CDCl3
76.8 CDCl3
55.3
17.9
N3
Me
N
MeO
26
13C NMR, 101 MHz, CDCl3
f1 (ppm)

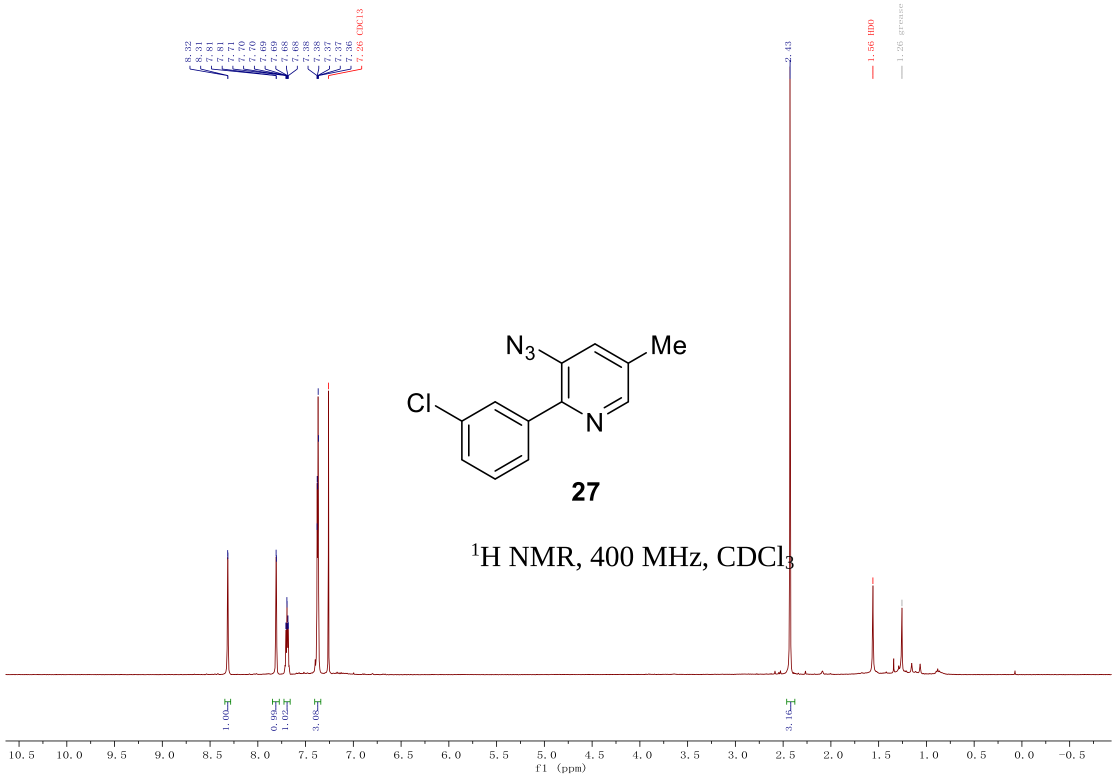

8.32 8.31 7.81 7.81 7.71 7.70 7.70 7.69 7.69 7.68 7.68 7.38 7.38 7.37 7.37 7.36 7.26 CDCl3
2.43
1.56 HDO
1.26 grease
N3
Me
Cl
N
27
1H NMR, 400 MHz, CDCl3
1.00
0.99
1.02
3.08
3.16
f1 (ppm)

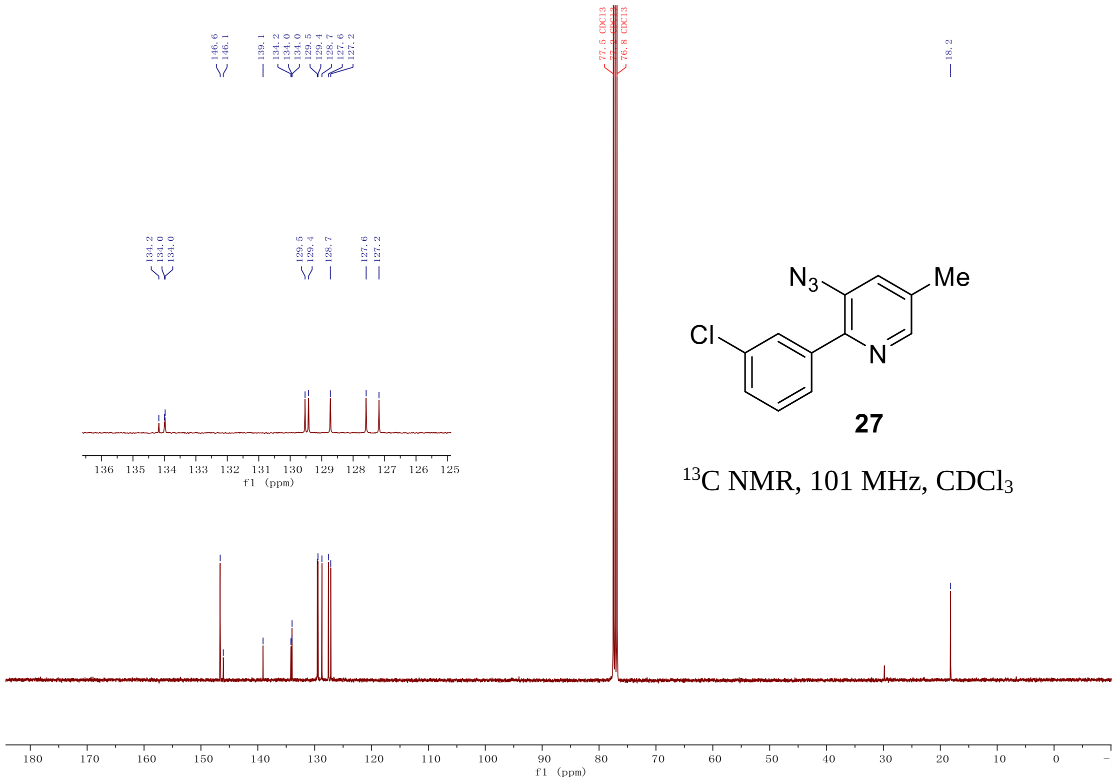

146.6 146.1 139.1 134.2 134.0 134.0 129.5 129.4 128.7 127.6 127.2
77.5 CDCl3 76.8 CDCl3
18.2
134.2 134.0 134.0 129.5 129.4 128.7 127.6 127.2
f1 (ppm)
N3
Me
Cl
N
27
13C NMR, 101 MHz, CDCl3
f1 (ppm)

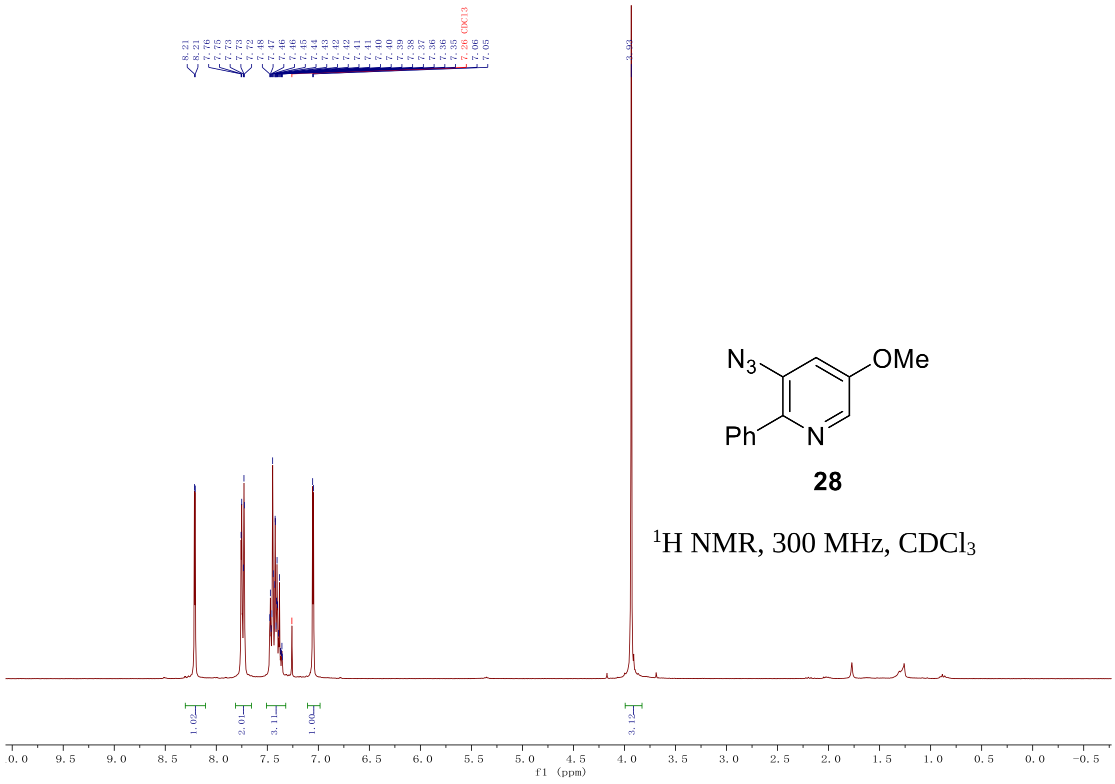

28

^{1}H NMR, 300 MHz, $CDCl_3$

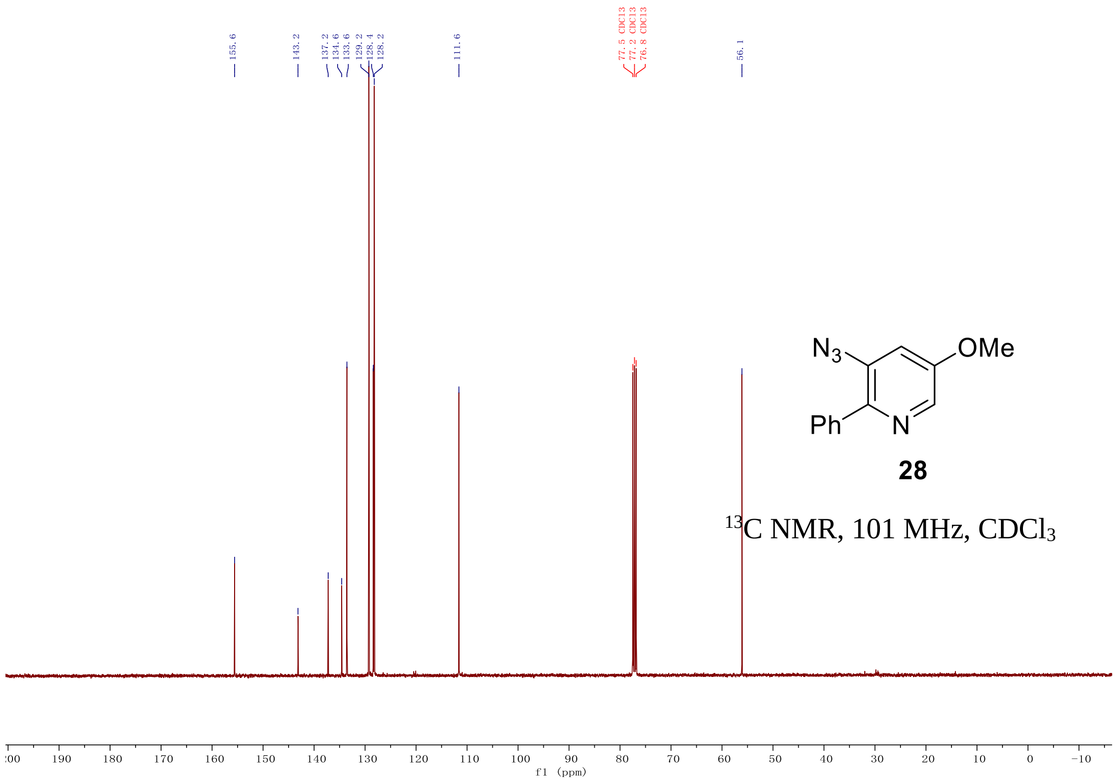

28

^{13}C NMR, 101 MHz, $CDCl_3$

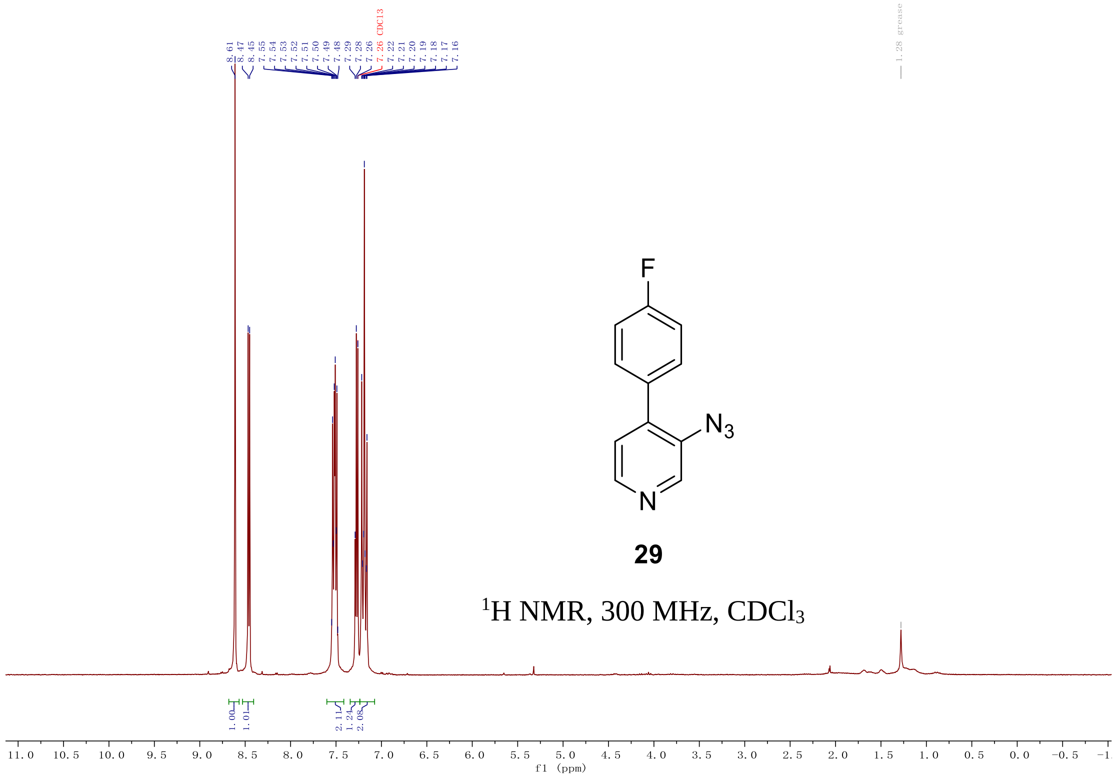

29

^{1}H NMR, 300 MHz, $CDCl_3$

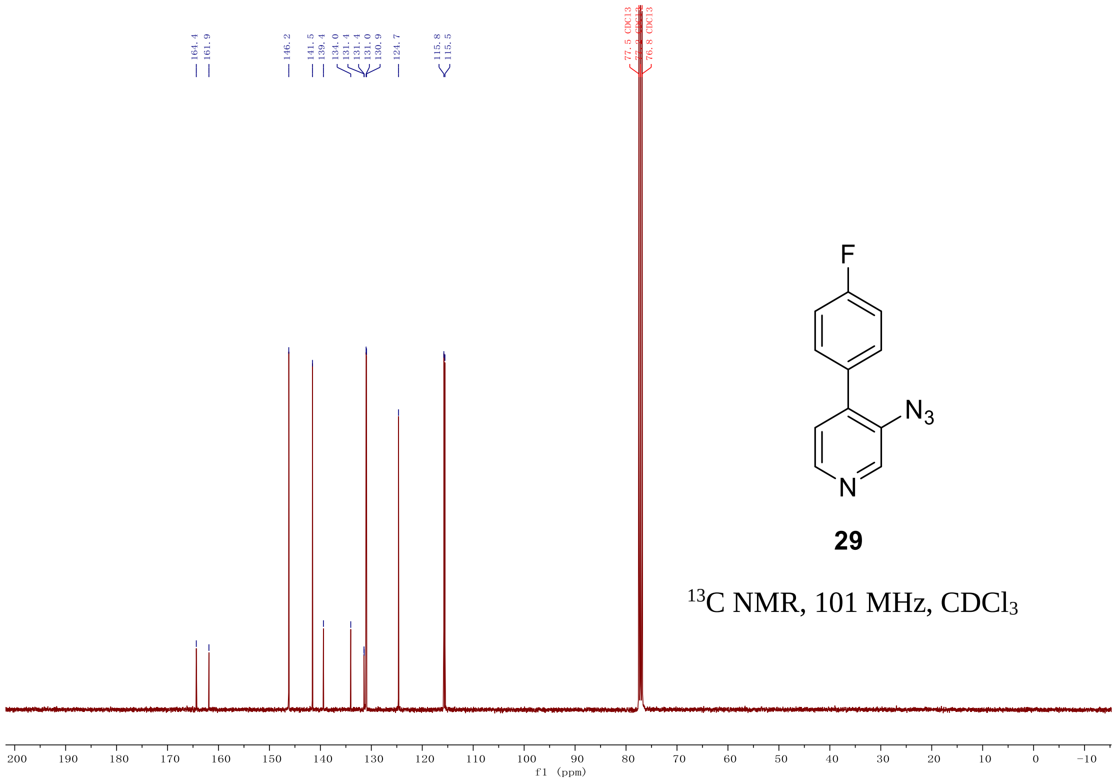

29

^{13}C NMR, 101 MHz, $CDCl_3$

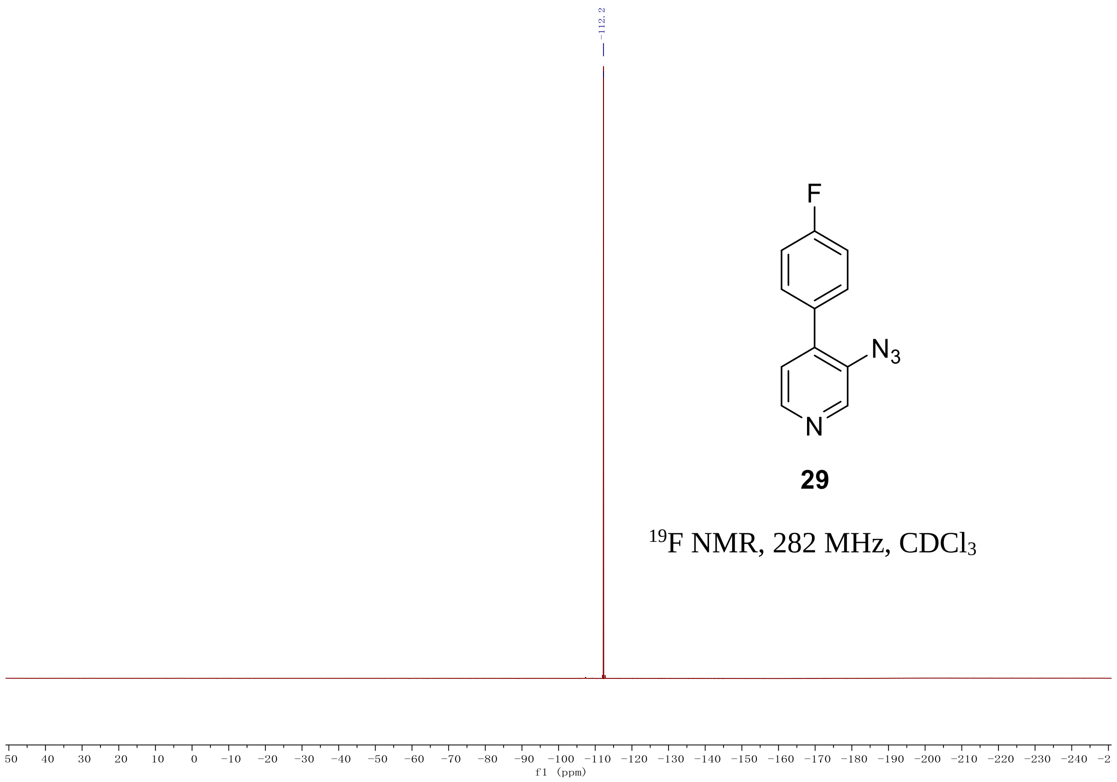

29

^{19}F NMR, 282 MHz, $CDCl_3$

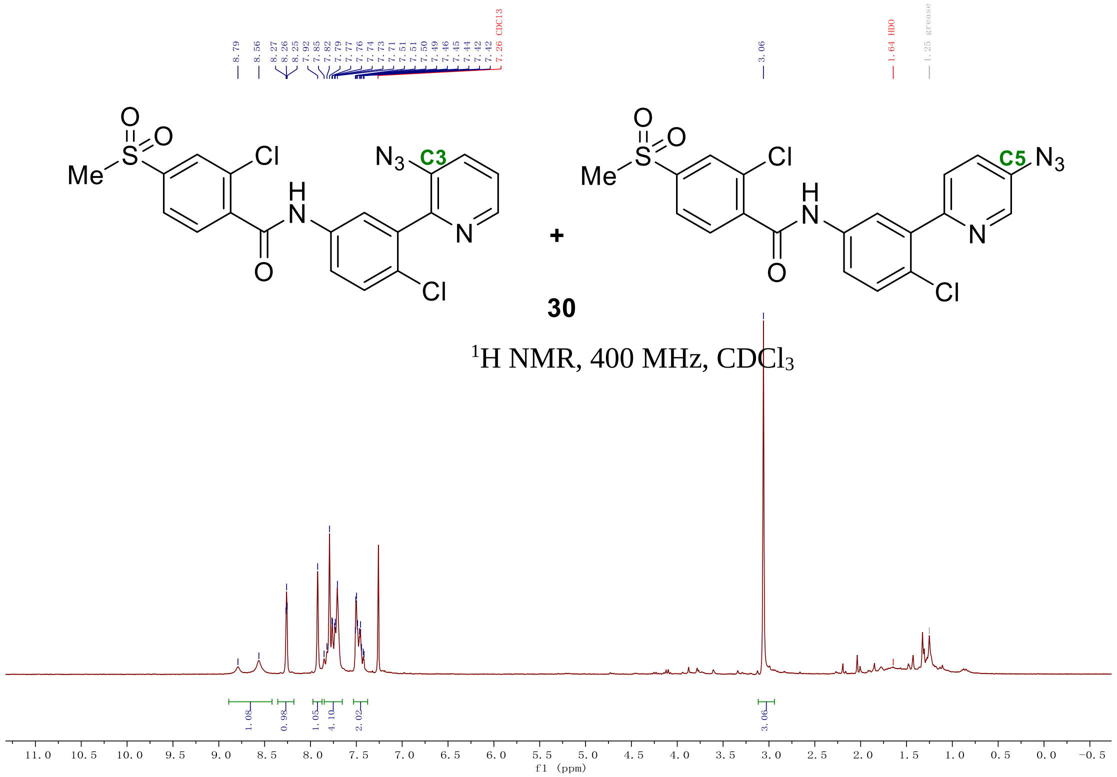

30

^{1}H NMR, 400 MHz, $CDCl_3$

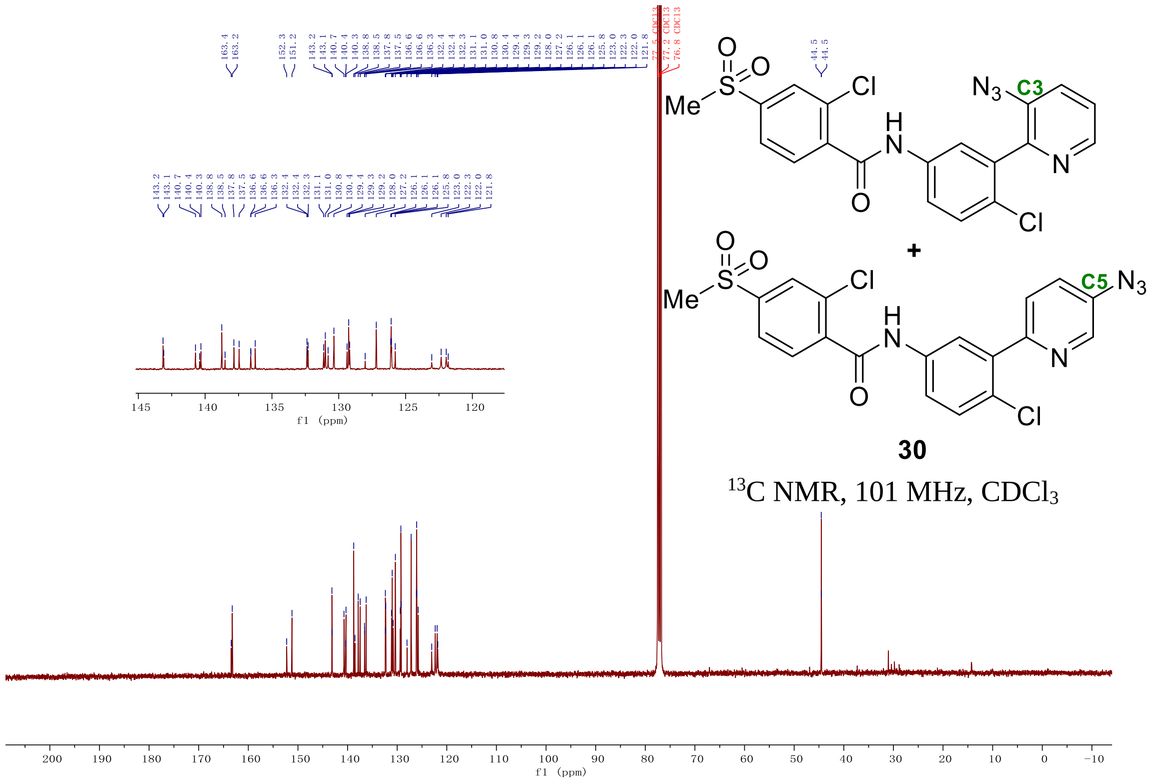

30

^{13}C NMR, 101 MHz, $CDCl_3$

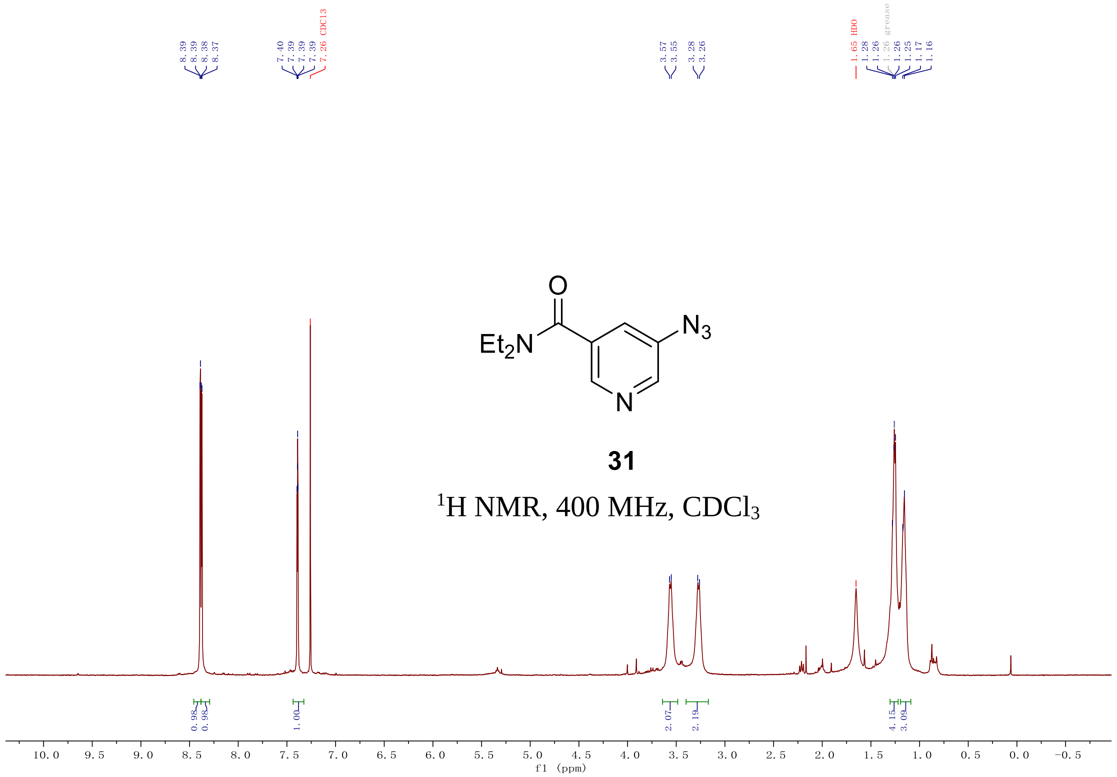

31

^{1}H NMR, 400 MHz, $CDCl_3$

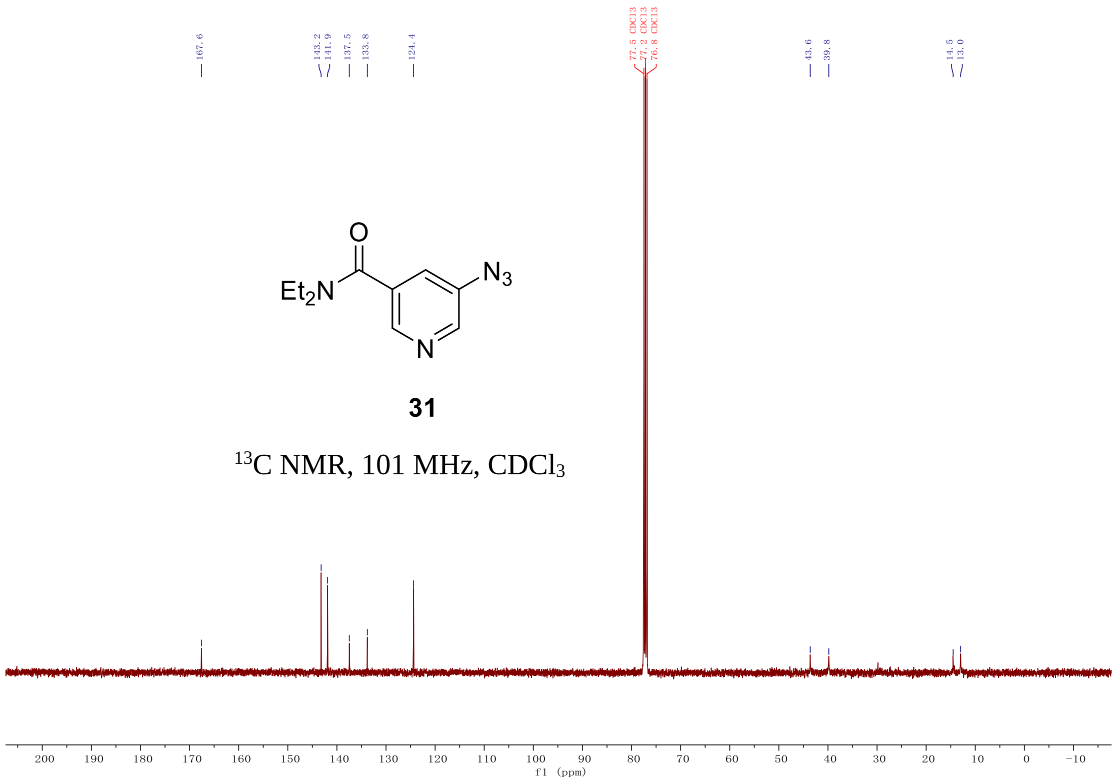

31

^{13}C NMR, 101 MHz, $CDCl_3$

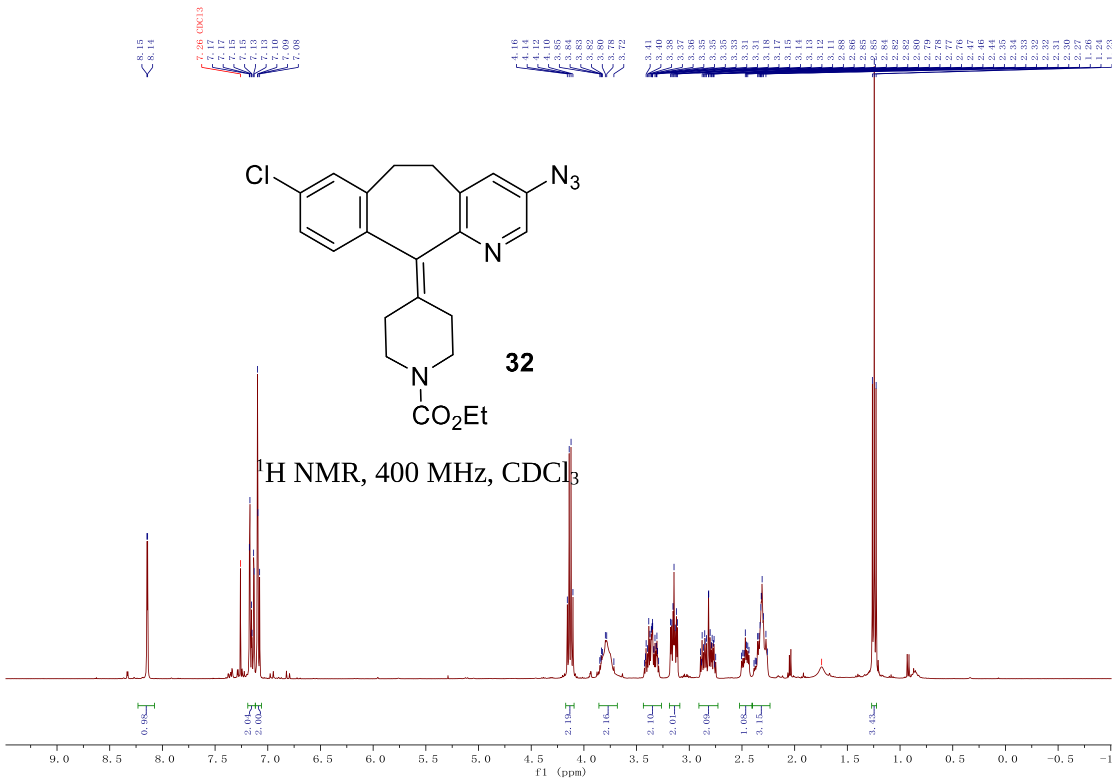
Cl
N3
N
N
CO2Et
32
1H NMR, 400 MHz, CDCl3
f1 (ppm)

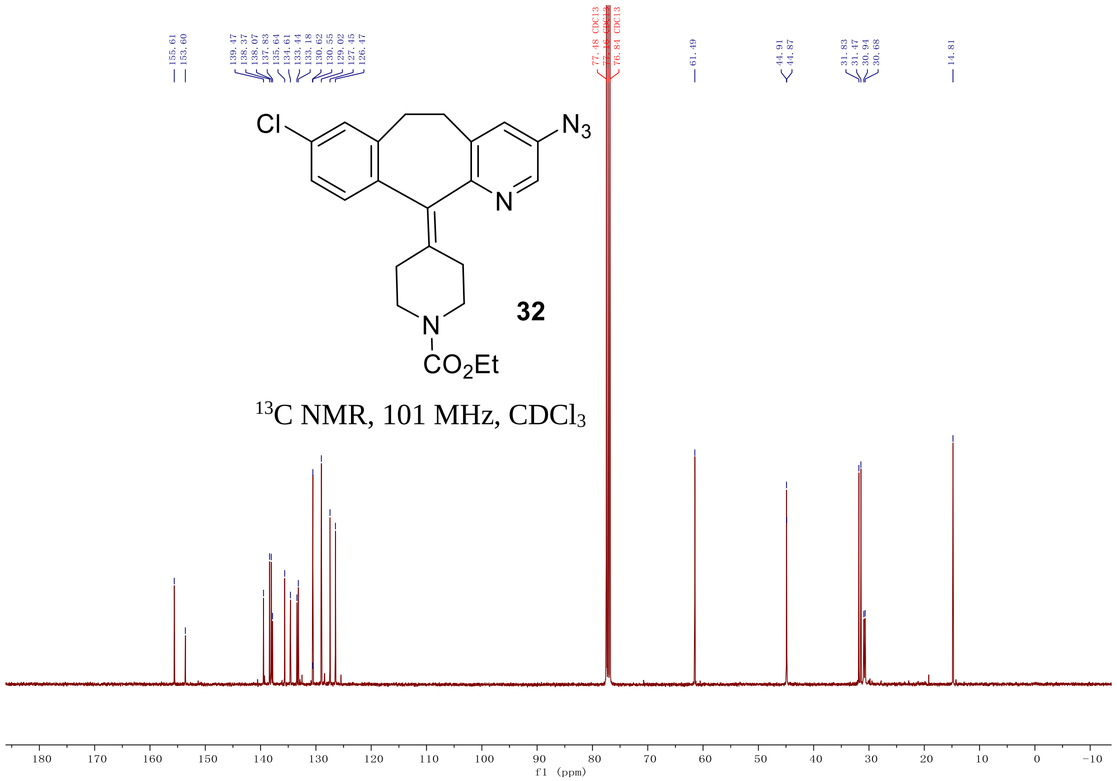
Cl
N3
N
N
CO2Et
32
13C NMR, 101 MHz, CDCl3
f1 (ppm)

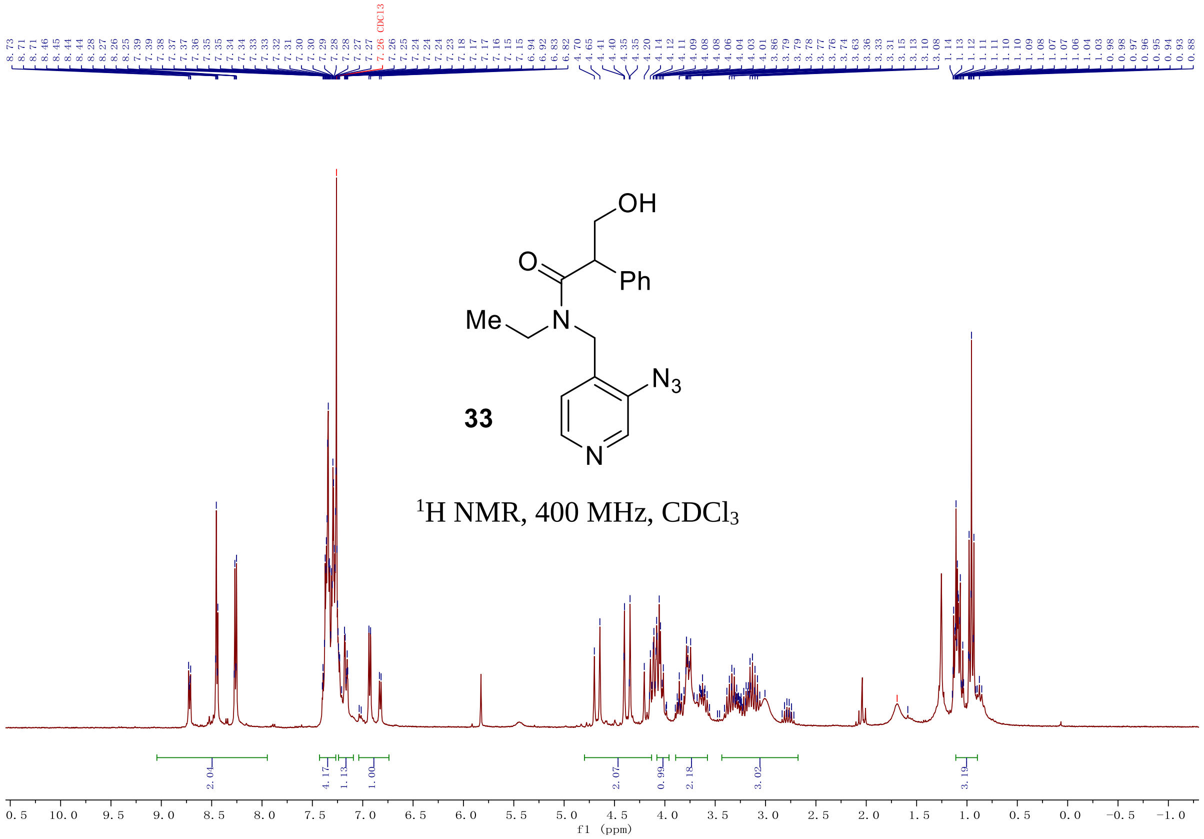
OH
O
Ph
Me
N
N3
33
N
1H NMR, 400 MHz, CDCl3
f1 (ppm)

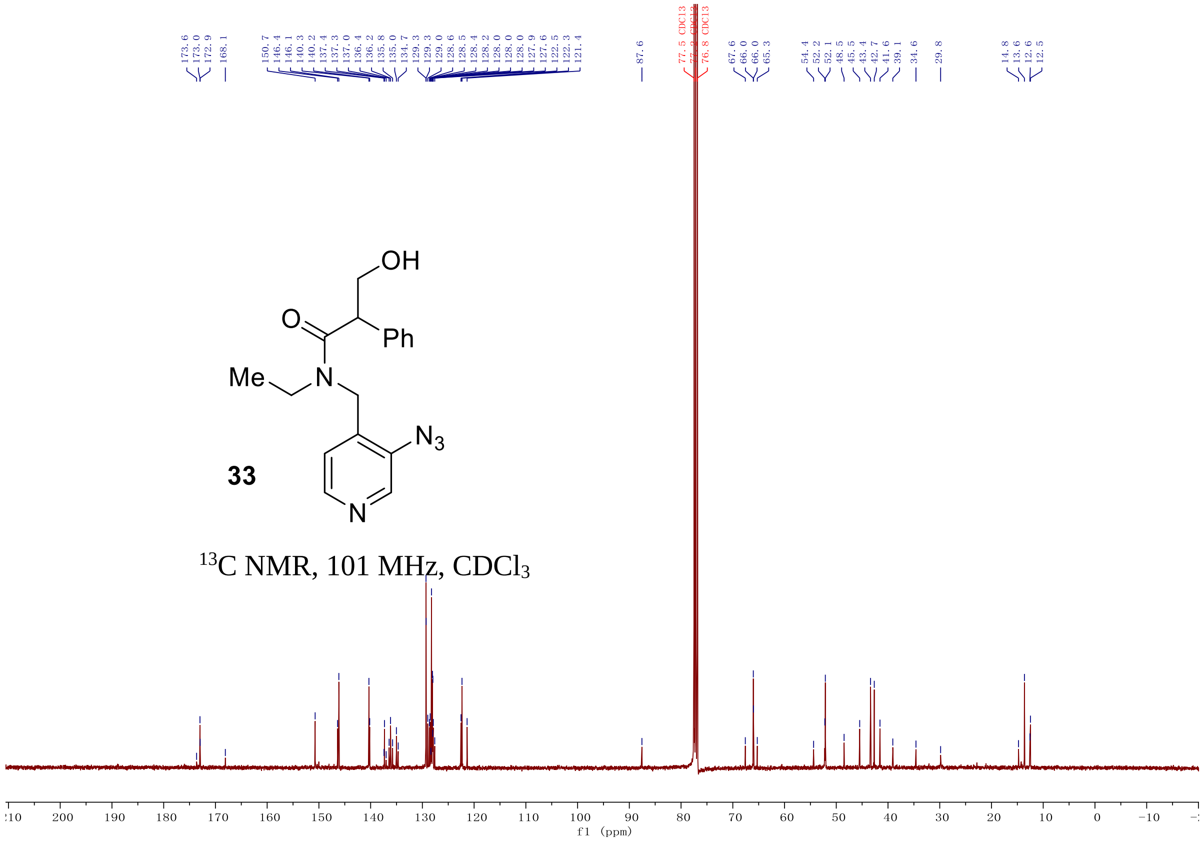
OH
O
Ph
Me
N
N3
33
N
13C NMR, 101 MHz, CDCl3
f1 (ppm)

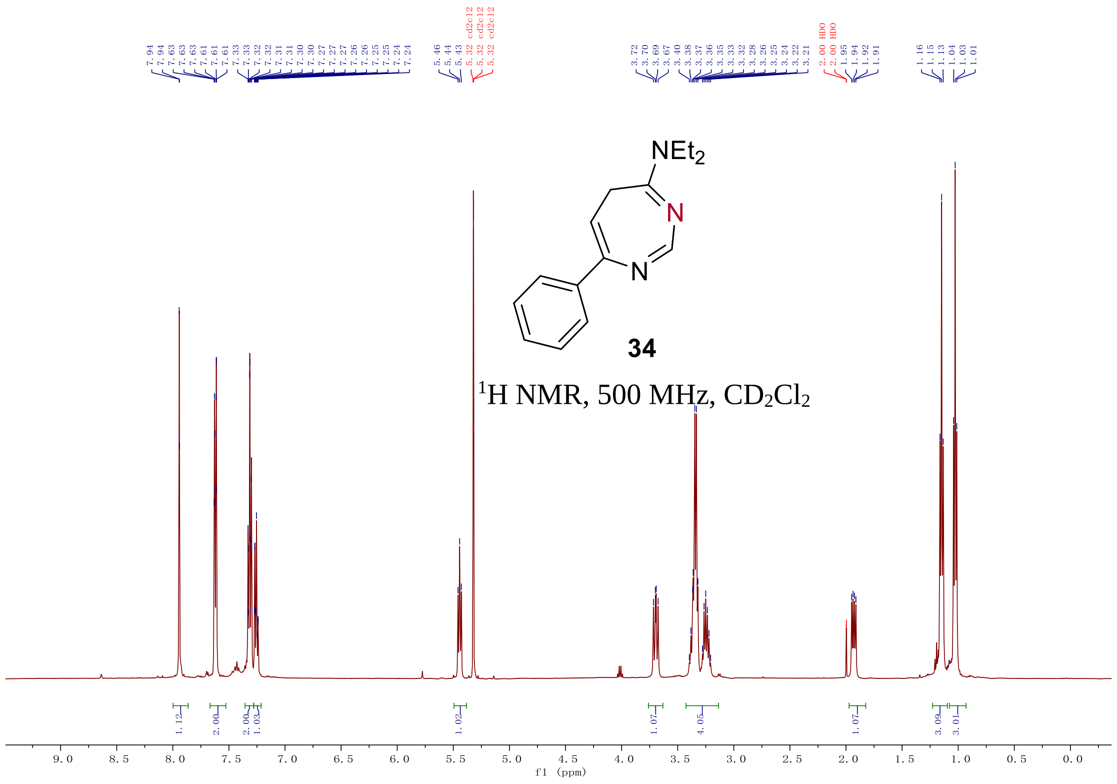
NEt2
N
N
34
1H NMR, 500 MHz, CD2Cl2
f1 (ppm)

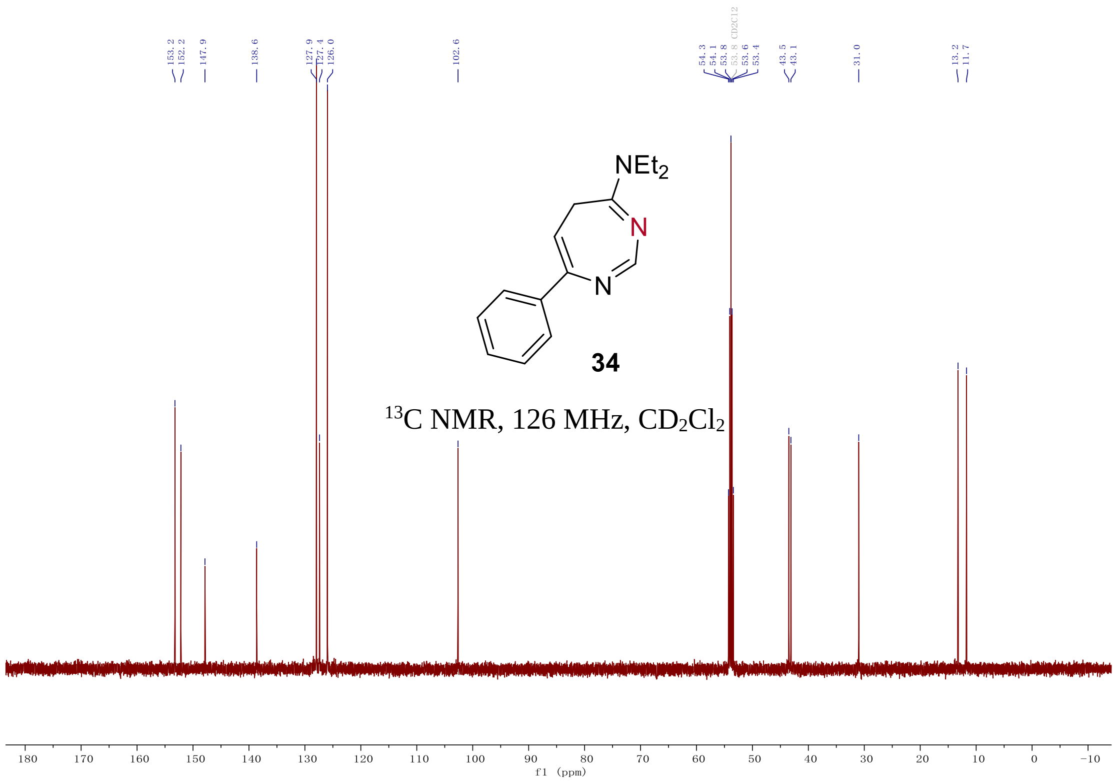
NEt2
N
N
34
13C NMR, 126 MHz, CD2Cl2
f1 (ppm)

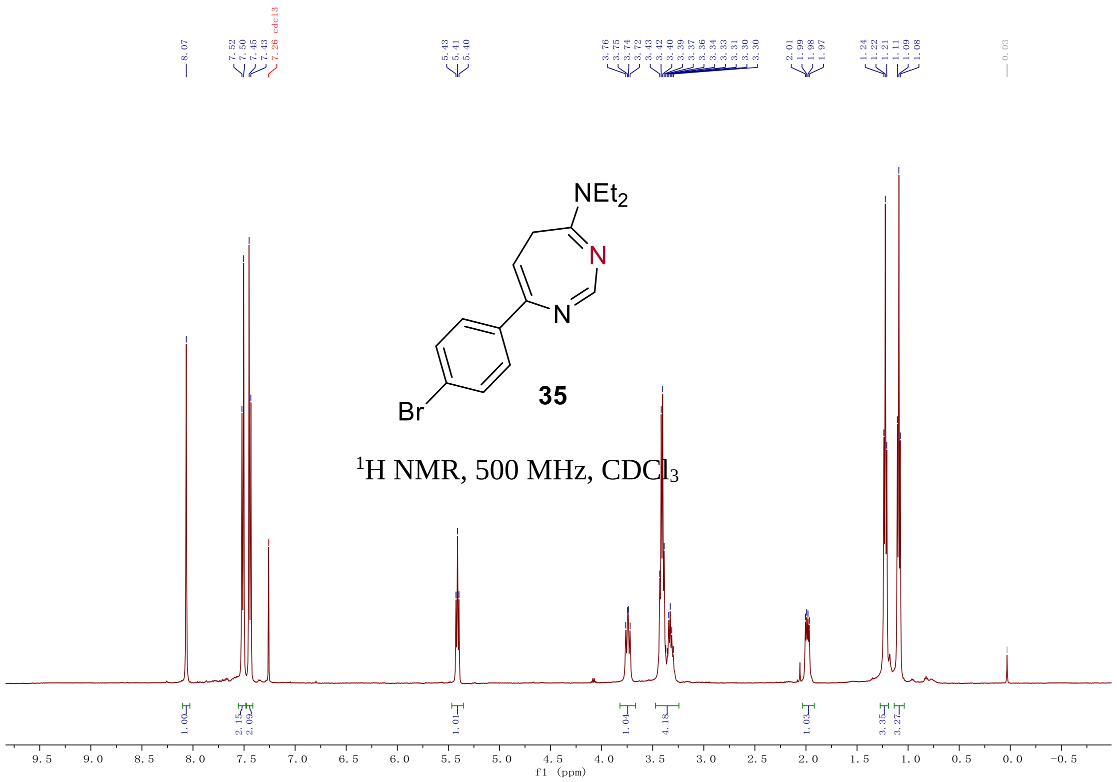
NEt2
N
N
Br
35
1H NMR, 500 MHz, CDCl3
f1 (ppm)

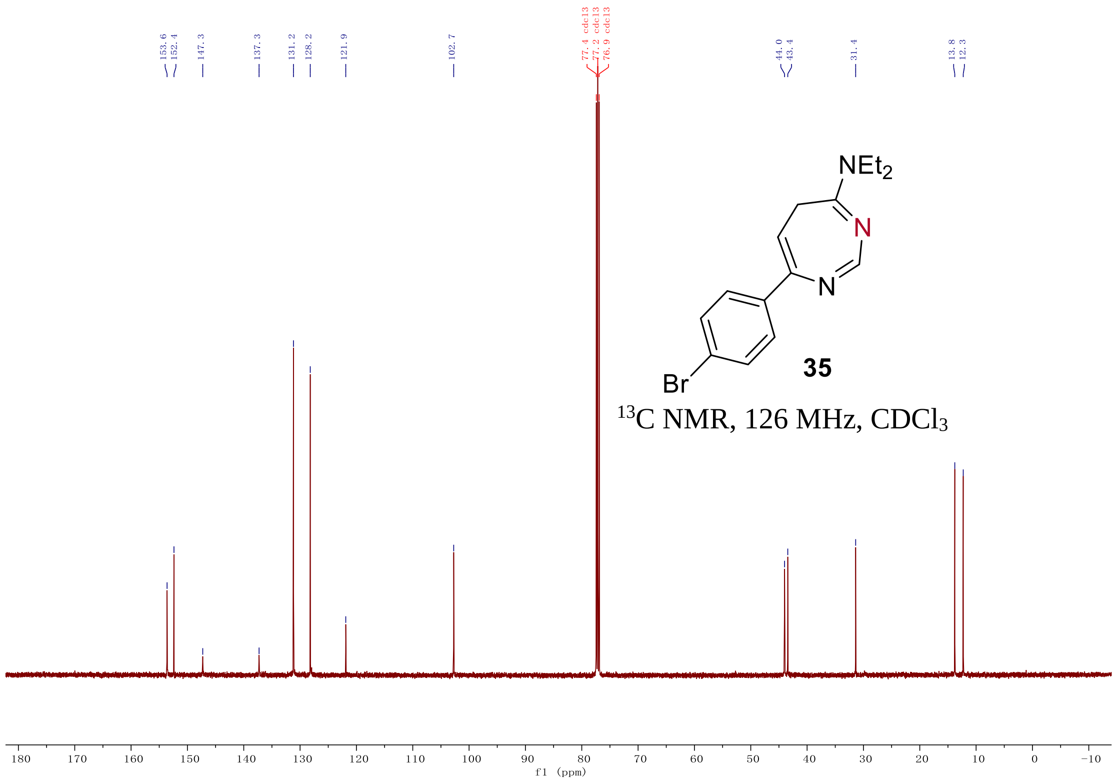
NEt2
N
N
Br
35
13C NMR, 126 MHz, CDCl3
f1 (ppm)

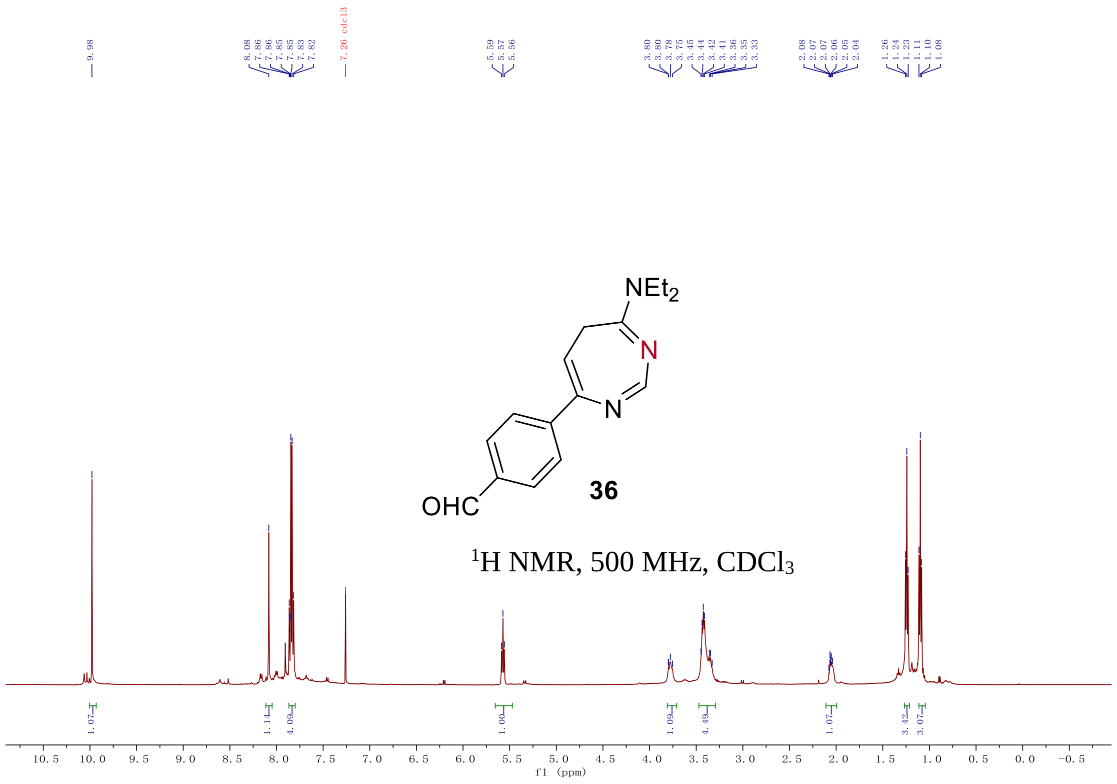

NEt2
N
N
OHC
36
1H NMR, 500 MHz, CDCl3
f1 (ppm)

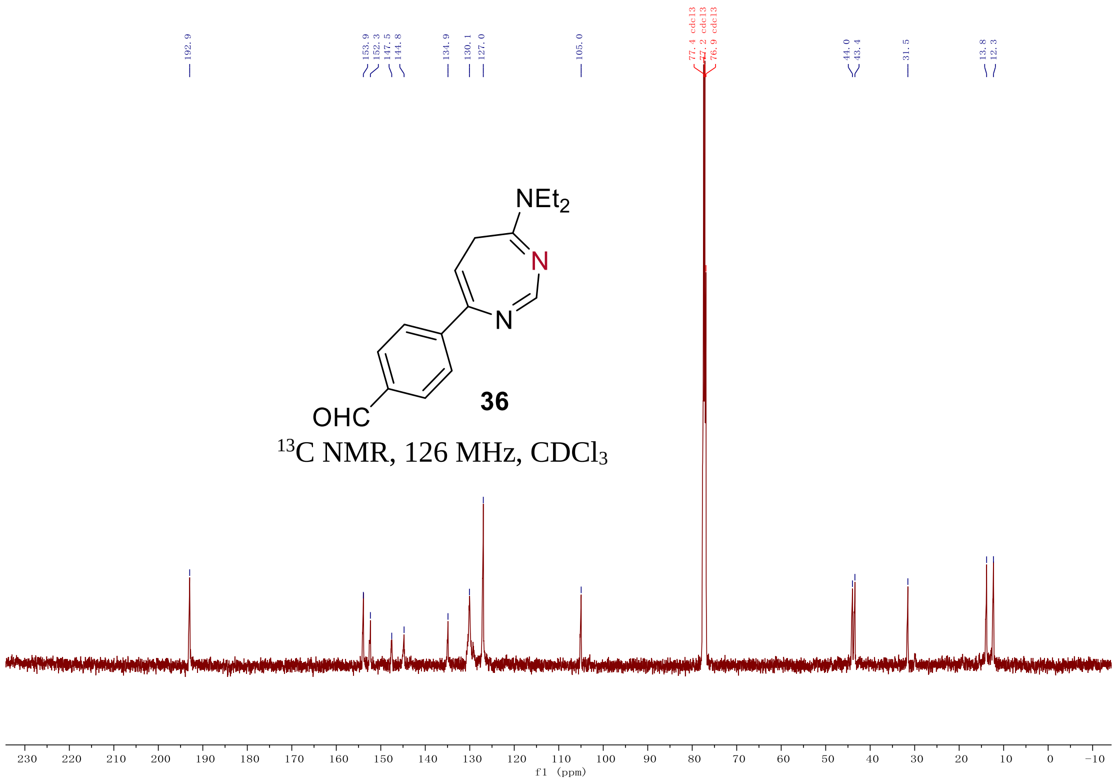

NEt2
N
N
OHC
36
13C NMR, 126 MHz, CDCl3
f1 (ppm)

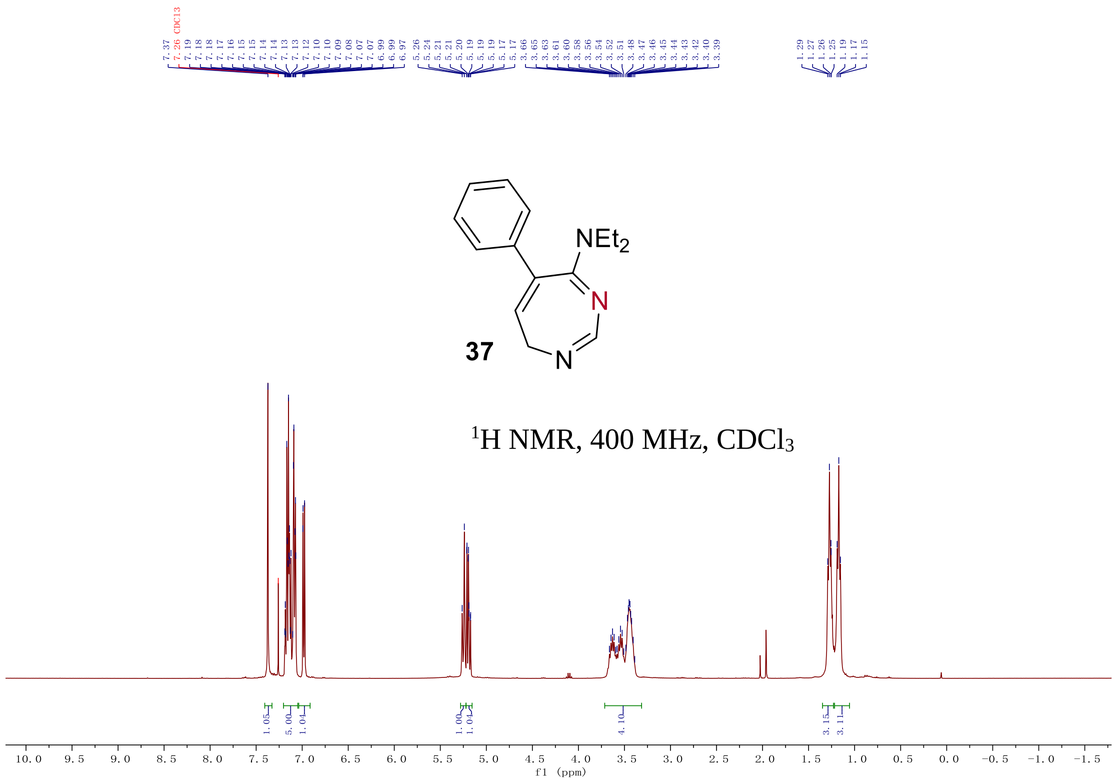
NEt2
N
37
N
1H NMR, 400 MHz, CDCl3
f1 (ppm)

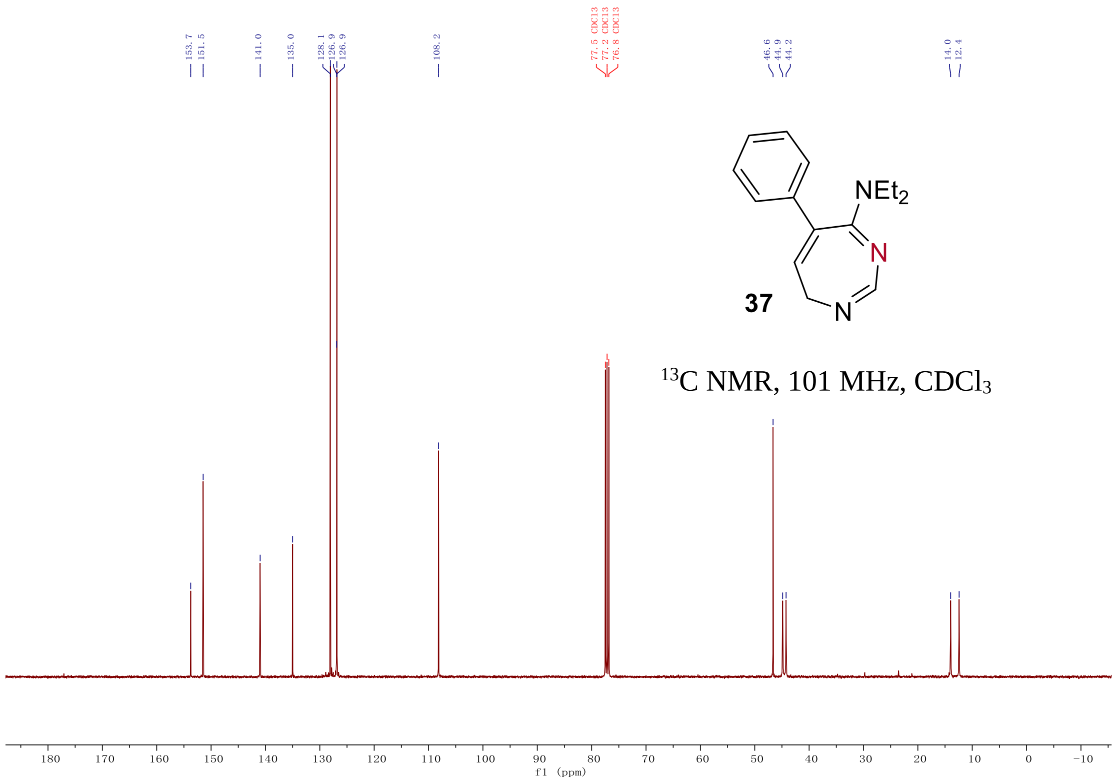
153.7
151.5
141.0
135.0
128.1
126.9
126.9
108.2
77.5 CDCl3
77.2 CDCl3
76.8 CDCl3
46.6
44.9
44.2
14.0
12.4
NEt2
N
37
N
13C NMR, 101 MHz, CDCl3
f1 (ppm)

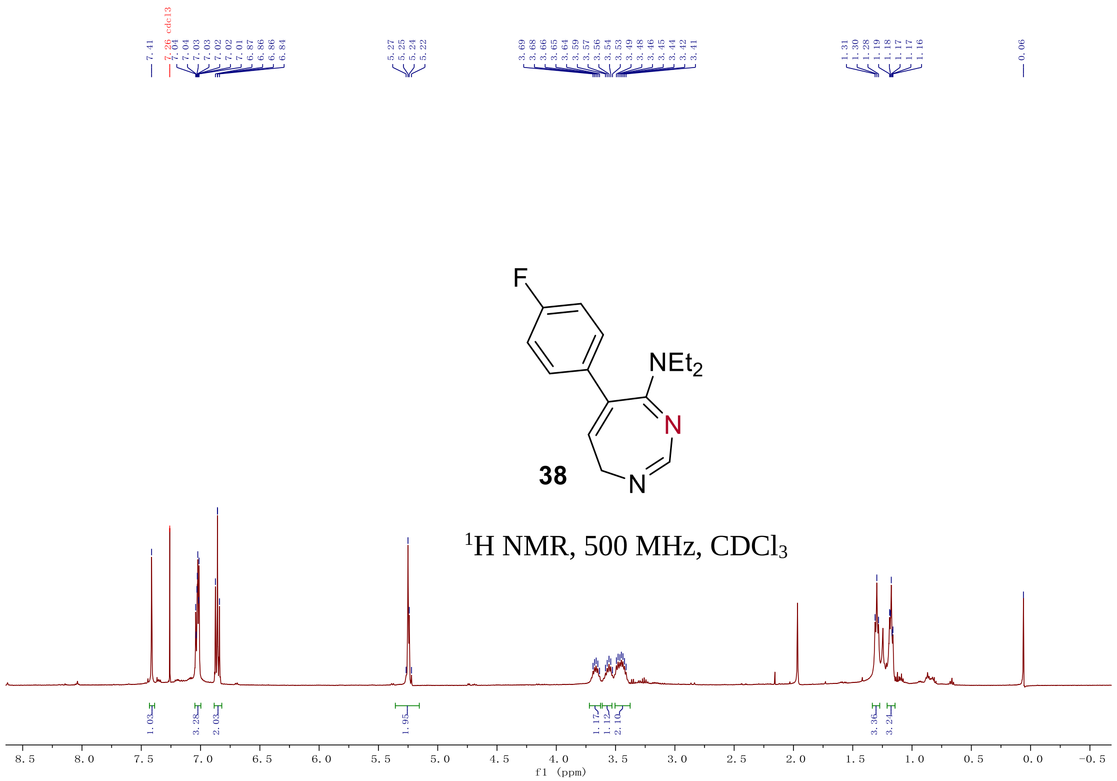

^{1}H NMR, 500 MHz, CDCl$_3$

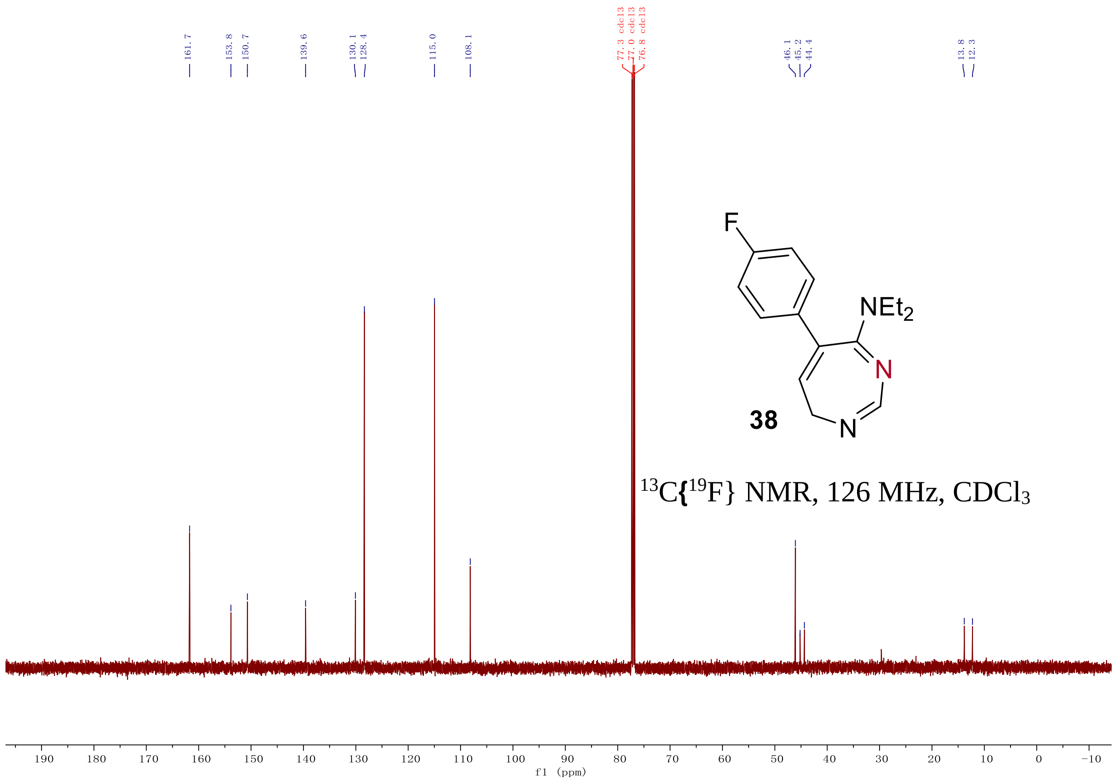

^{13}C{^{19}F} NMR, 126 MHz, CDCl$_3$

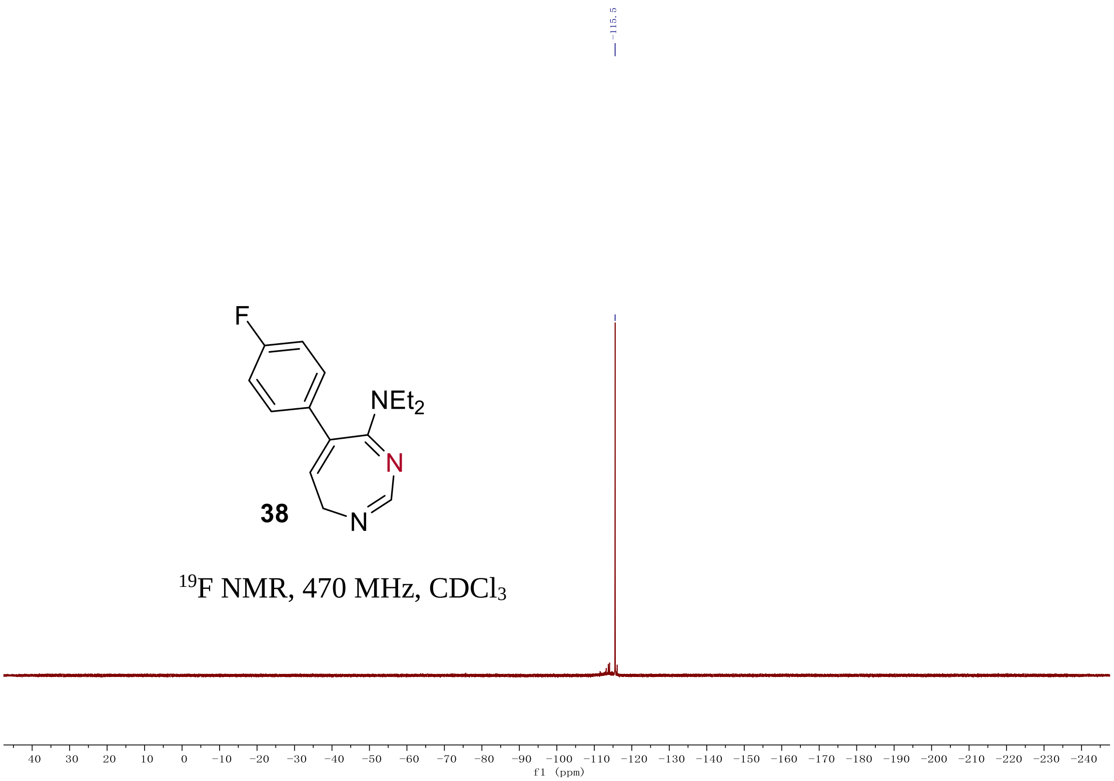

-115.5
F
NEt2
N
N
38
19F NMR, 470 MHz, CDCl3
40 30 20 10 0 -10 -20 -30 -40 -50 -60 -70 -80 -90 -100 -110 -120 -130 -140 -150 -160 -170 -180 -190 -200 -210 -220 -230 -240
f1 (ppm)

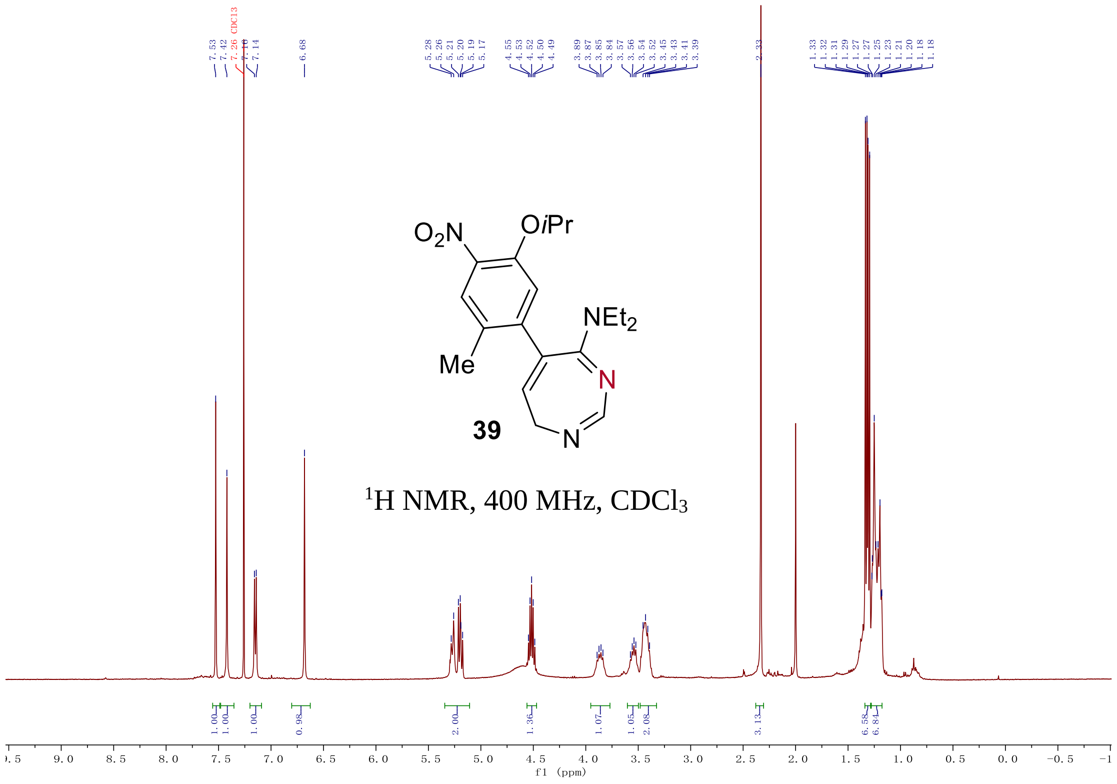

^1H NMR, 400 MHz, $CDCl_3$

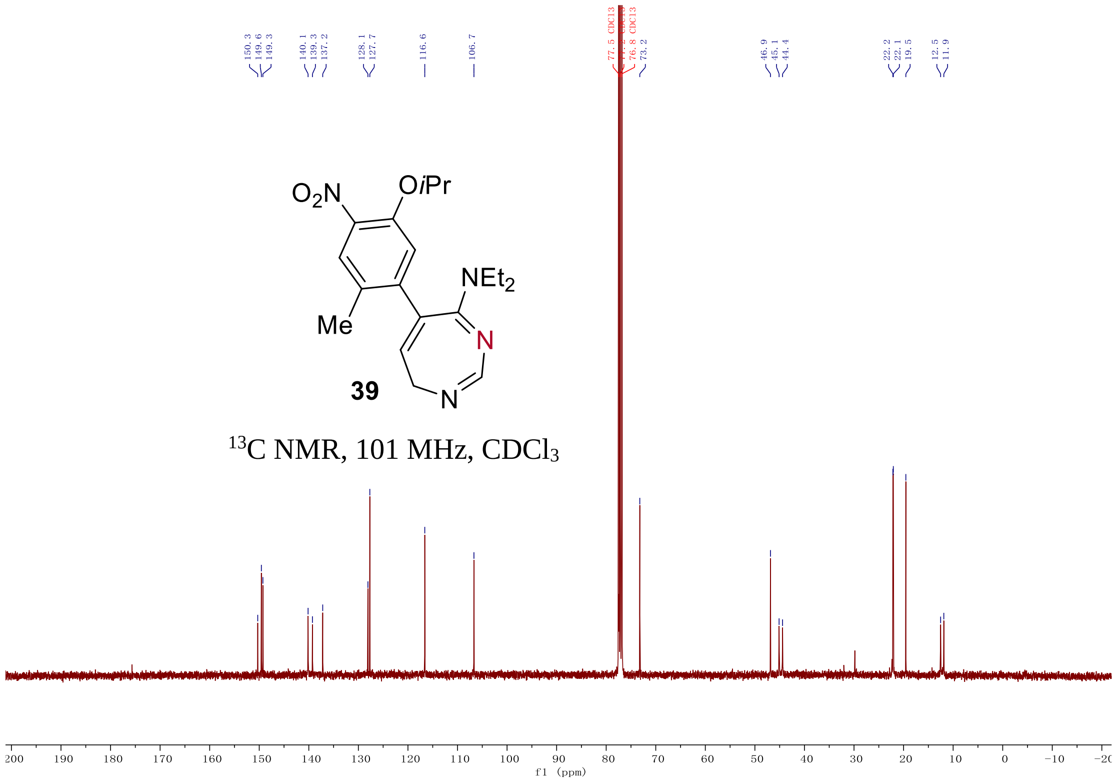

^{13}C NMR, 101 MHz, $CDCl_3$

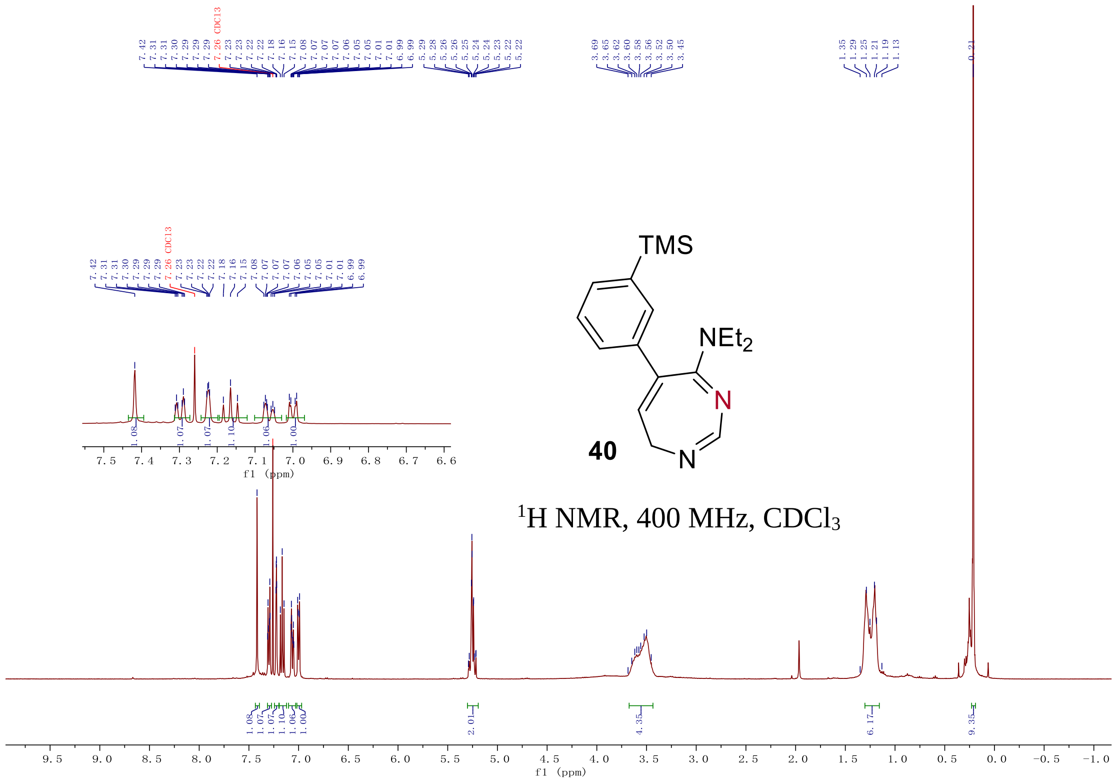

^{1}H NMR, 400 MHz, $CDCl_3$

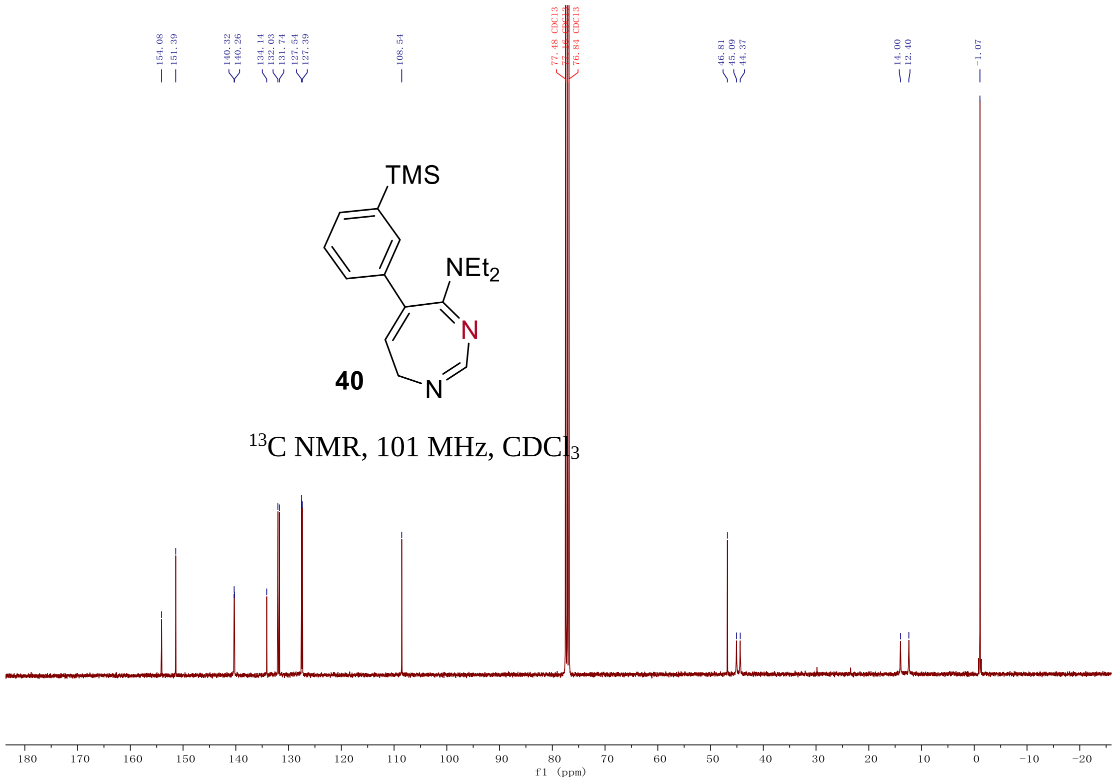

^{13}C NMR, 101 MHz, $CDCl_3$

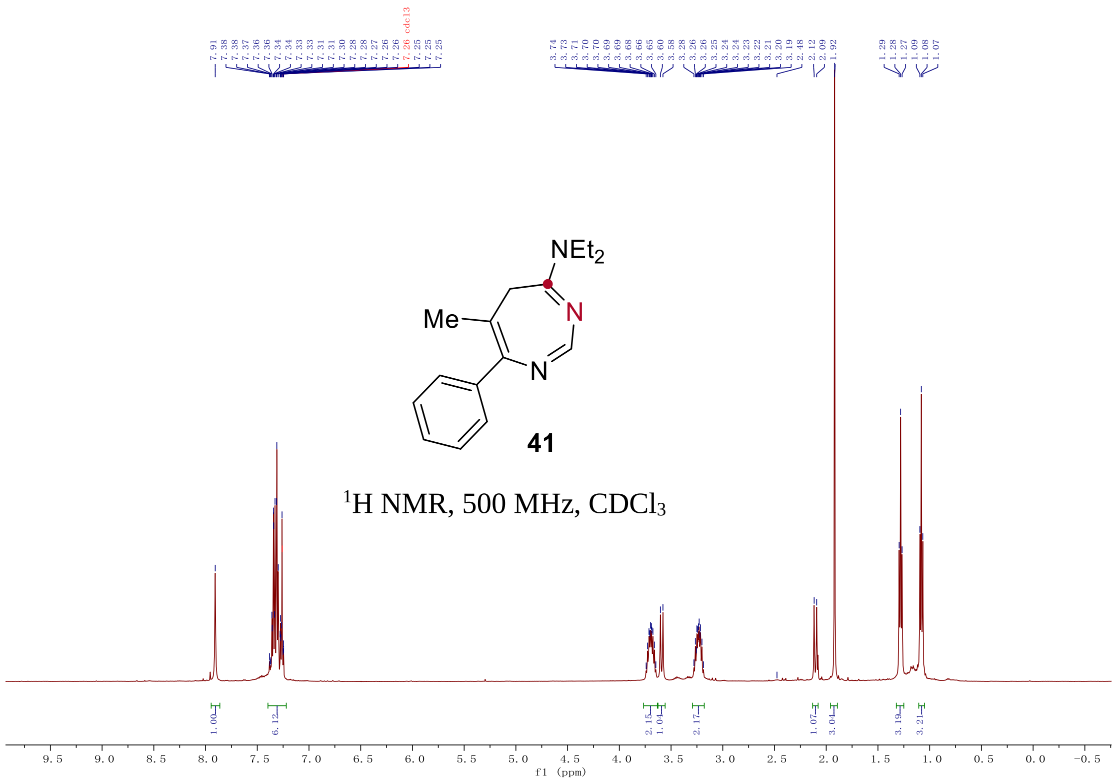
7.91
7.38
7.38
7.37
7.36
7.36
7.34
7.34
7.33
7.33
7.31
7.31
7.30
7.28
7.28
7.27
7.27
7.26
7.26
7.26 cdcl3
7.25
7.25
7.25
3.74
3.73
3.71
3.70
3.70
3.69
3.69
3.68
3.66
3.65
3.60
3.58
3.28
3.26
3.26
3.25
3.24
3.24
3.23
3.22
3.21
3.20
3.19
2.48
2.12
2.09
1.92
1.29
1.28
1.27
1.09
1.08
1.07
NEt2
Me
N
N
41
1H NMR, 500 MHz, CDCl3
1.00
6.12
2.15
1.04
2.17
1.07
3.04
3.19
3.21
f1 (ppm)

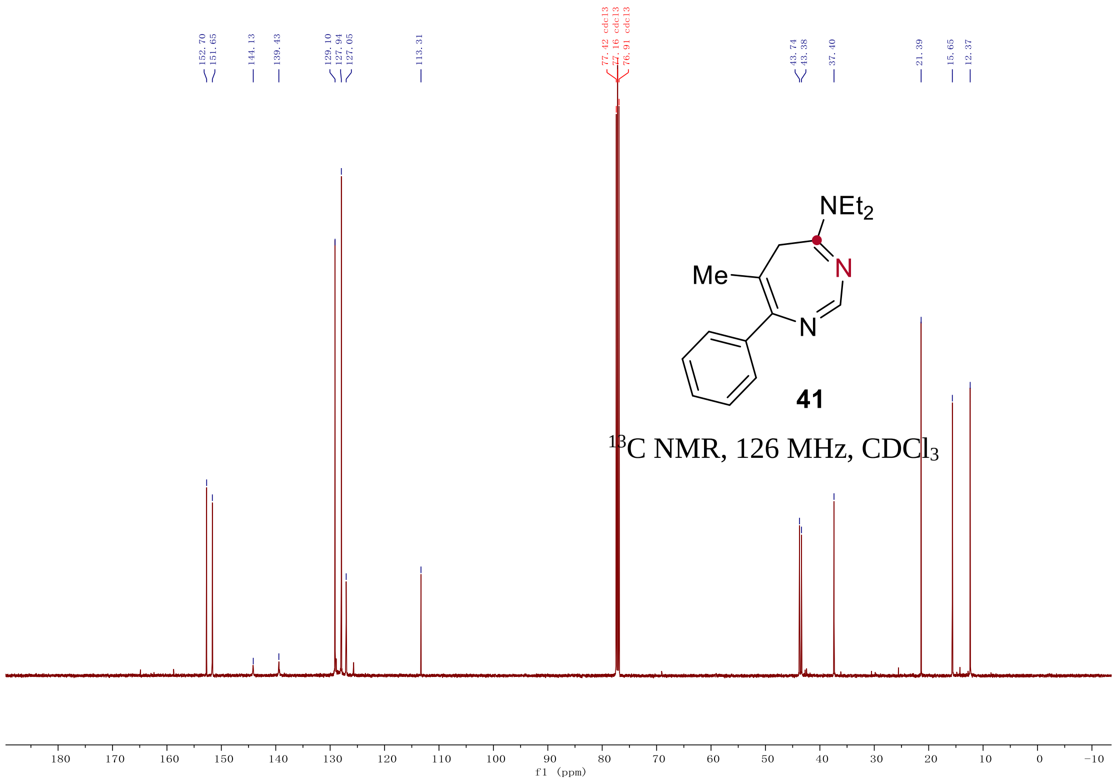
152.70
151.65
144.13
139.43
129.10
127.94
127.05
113.31
77.42 cdcl3
77.16 cdcl3
76.91 cdcl3
43.74
43.38
37.40
21.39
15.65
12.37
NEt2
Me
N
N
41
13C NMR, 126 MHz, CDCl3
f1 (ppm)

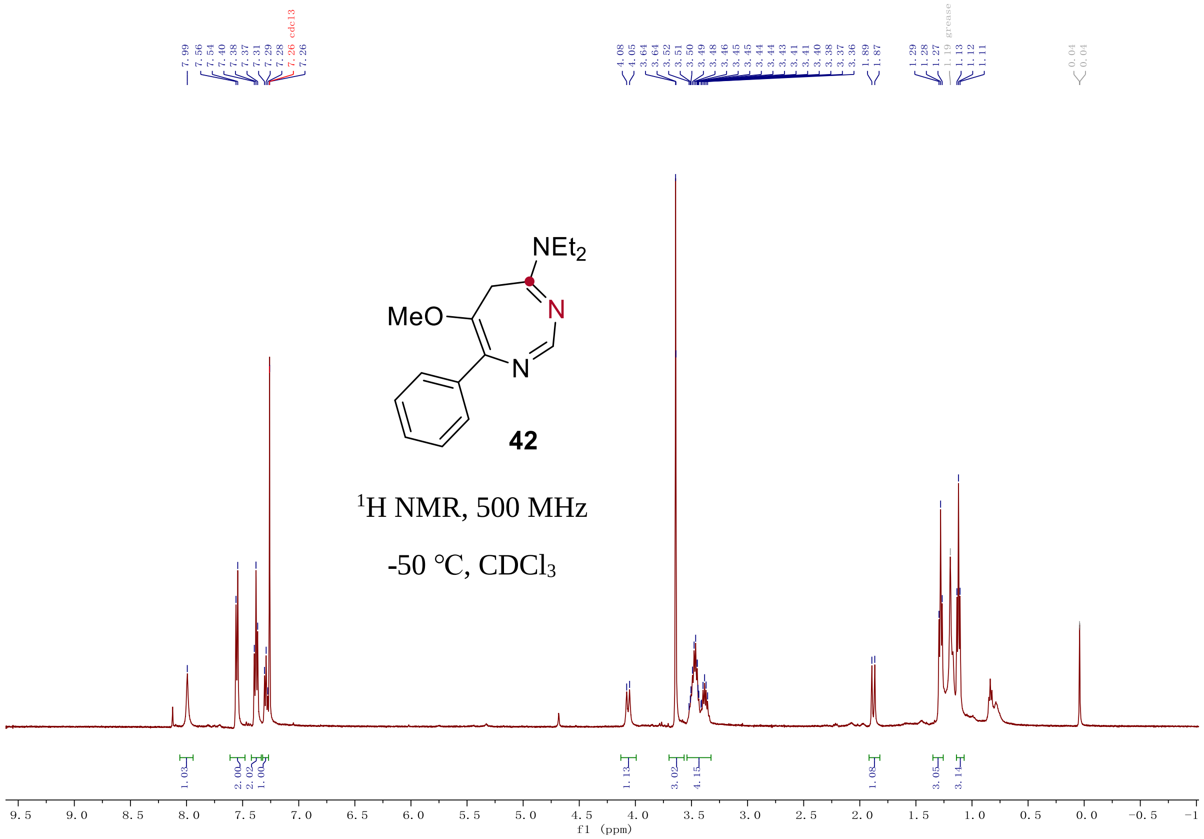
NEt2
MeO
N
N
42
1H NMR, 500 MHz
-50 ℃, CDCl3
f1 (ppm)

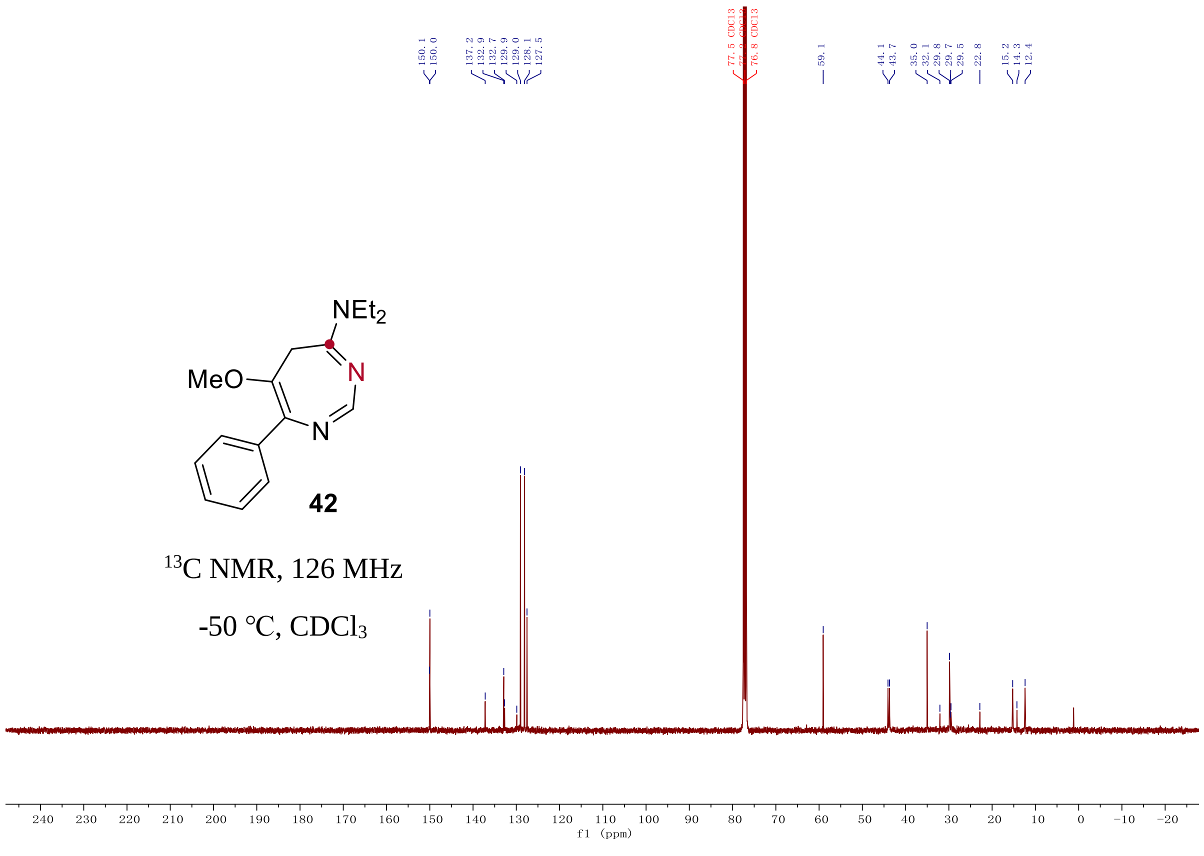
NEt2
MeO
N
N
42
13C NMR, 126 MHz
-50 ℃, CDCl3
f1 (ppm)

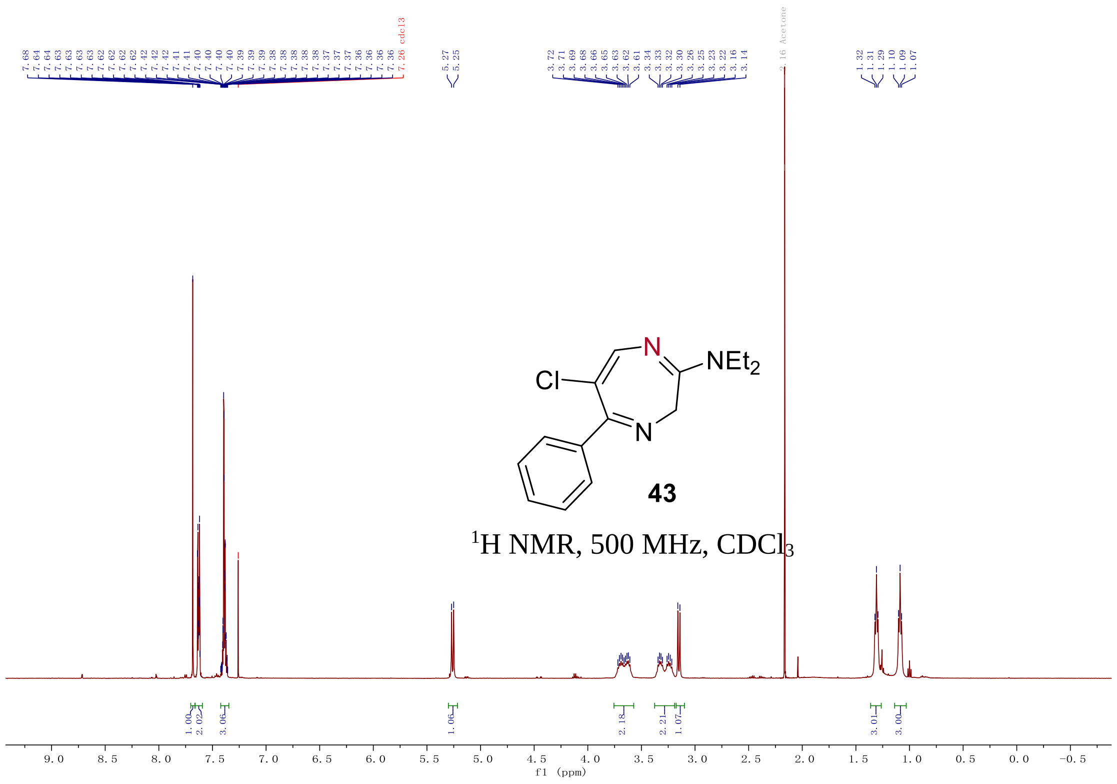
Cl
N
NEt2
N
43
1H NMR, 500 MHz, CDCl3
f1 (ppm)

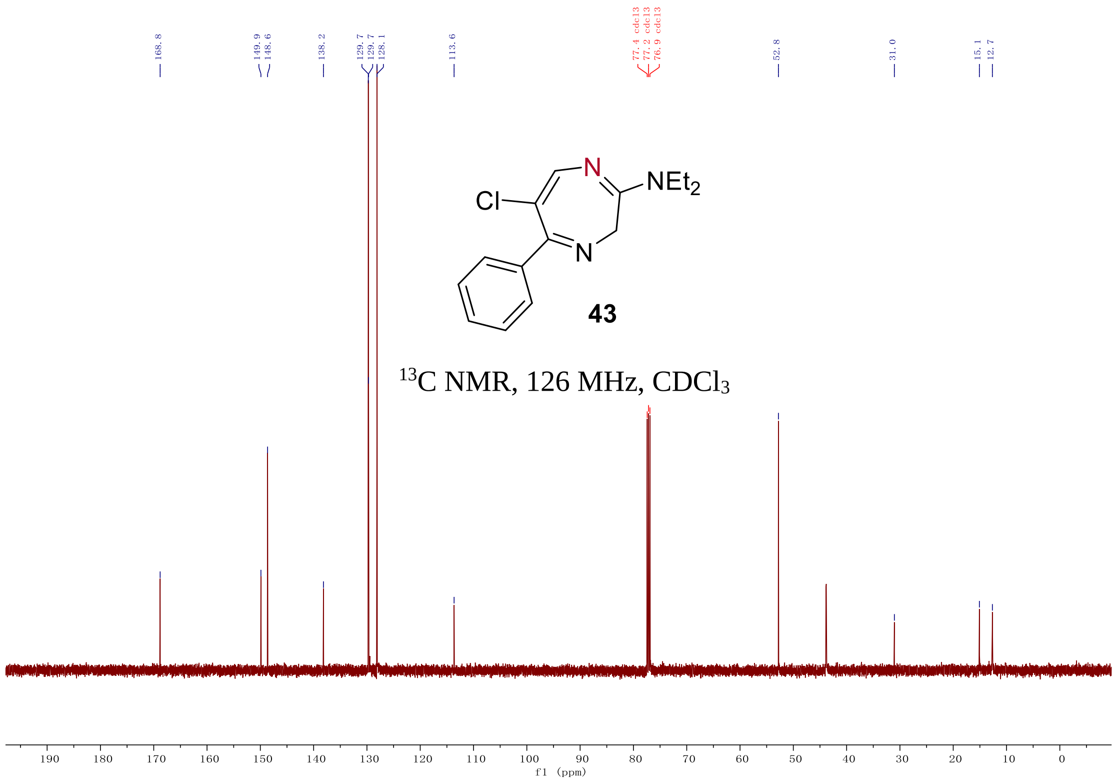
Cl
N
NEt2
N
43
13C NMR, 126 MHz, CDCl3
f1 (ppm)

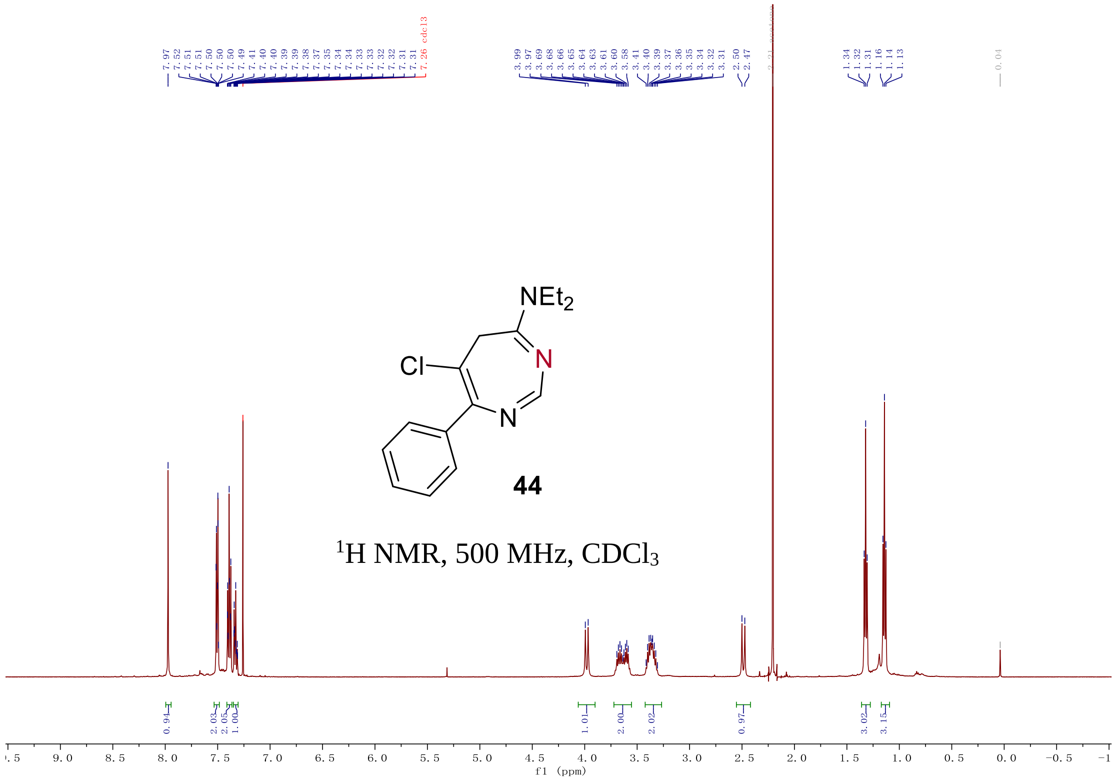

^{1}H NMR, 500 MHz, $CDCl_3$

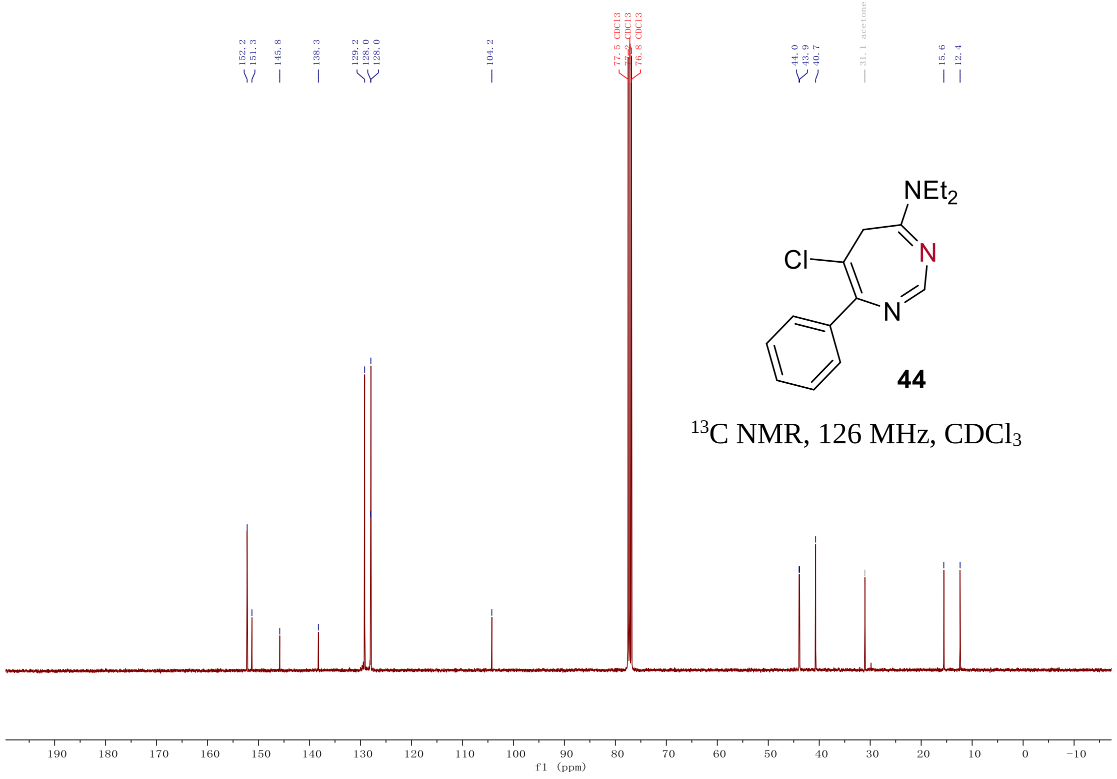

^{13}C NMR, 126 MHz, $CDCl_3$

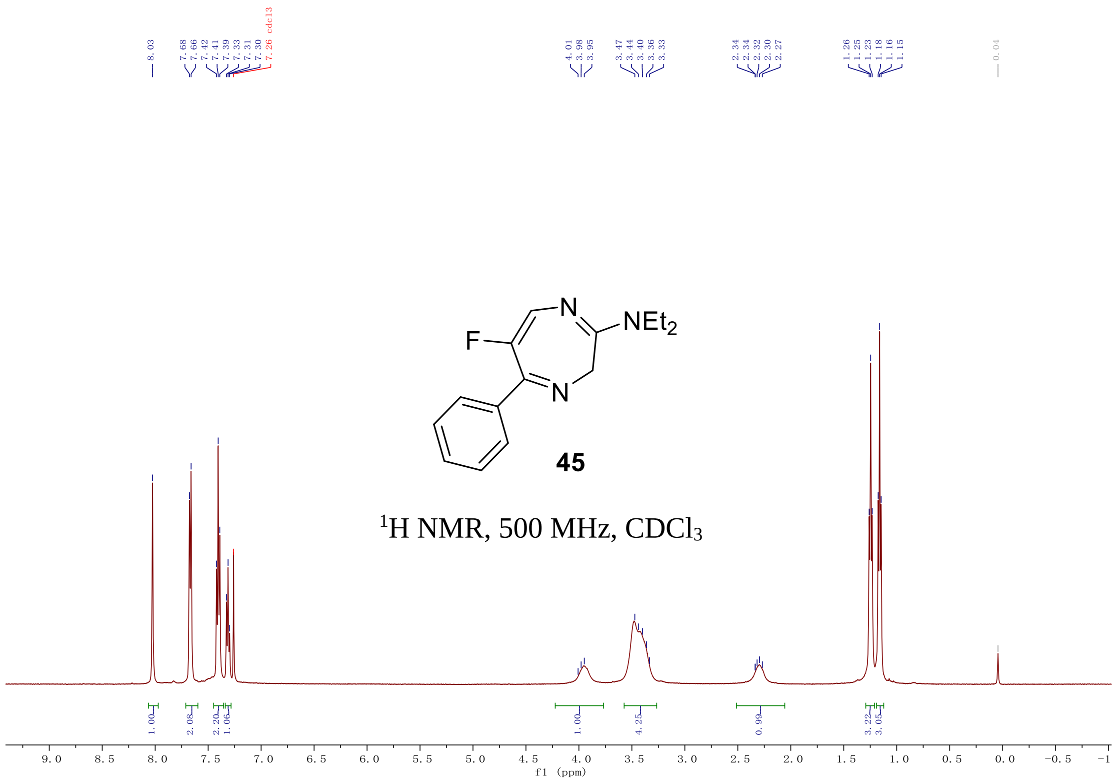
8.03
7.68
7.66
7.42
7.41
7.39
7.33
7.31
7.30
7.26 cdcl3
4.01
3.98
3.95
3.47
3.44
3.40
3.36
3.33
2.34
2.34
2.32
2.30
2.27
1.26
1.25
1.23
1.18
1.16
1.15
NEt2
F
N
45
1H NMR, 500 MHz, CDCl3
1.00
2.08
2.20
1.06
1.00
4.25
0.99
3.22
3.05
f1 (ppm)

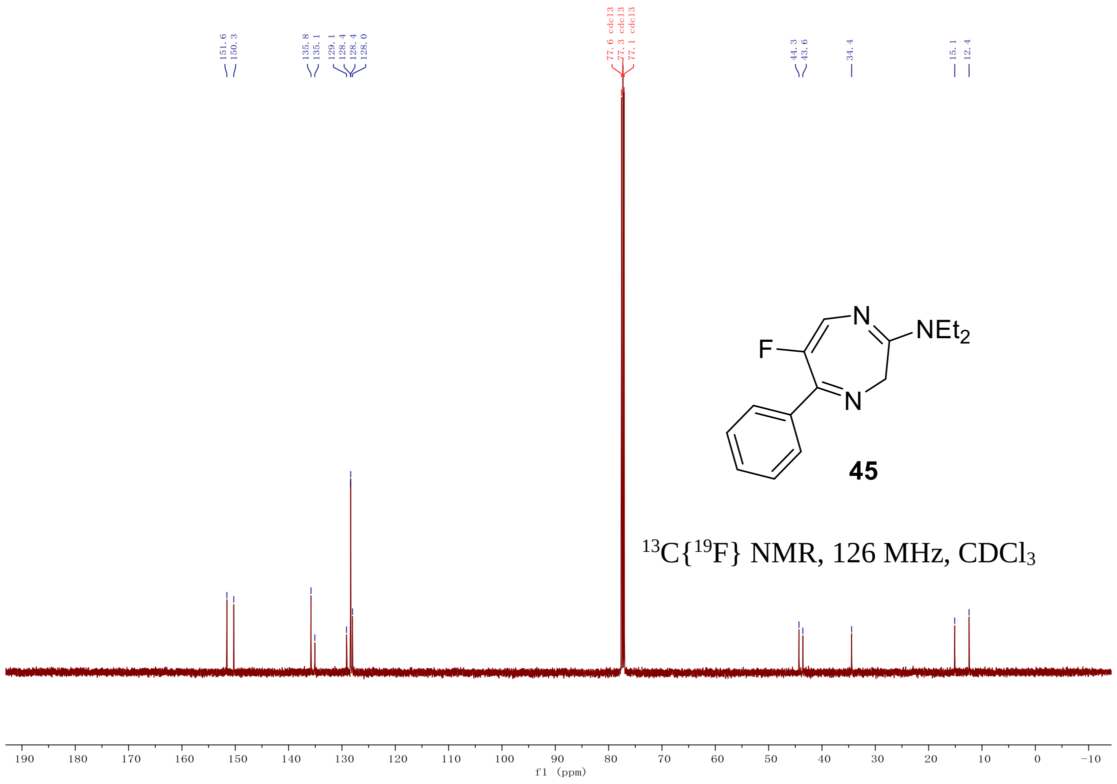
151.6
150.3
135.8
135.1
129.1
128.4
128.4
128.0
77.6 cdcl3
77.3 cdcl3
77.1 cdcl3
44.3
43.6
34.4
15.1
12.4
NEt2
F
N
45
13C{19F} NMR, 126 MHz, CDCl3
f1 (ppm)

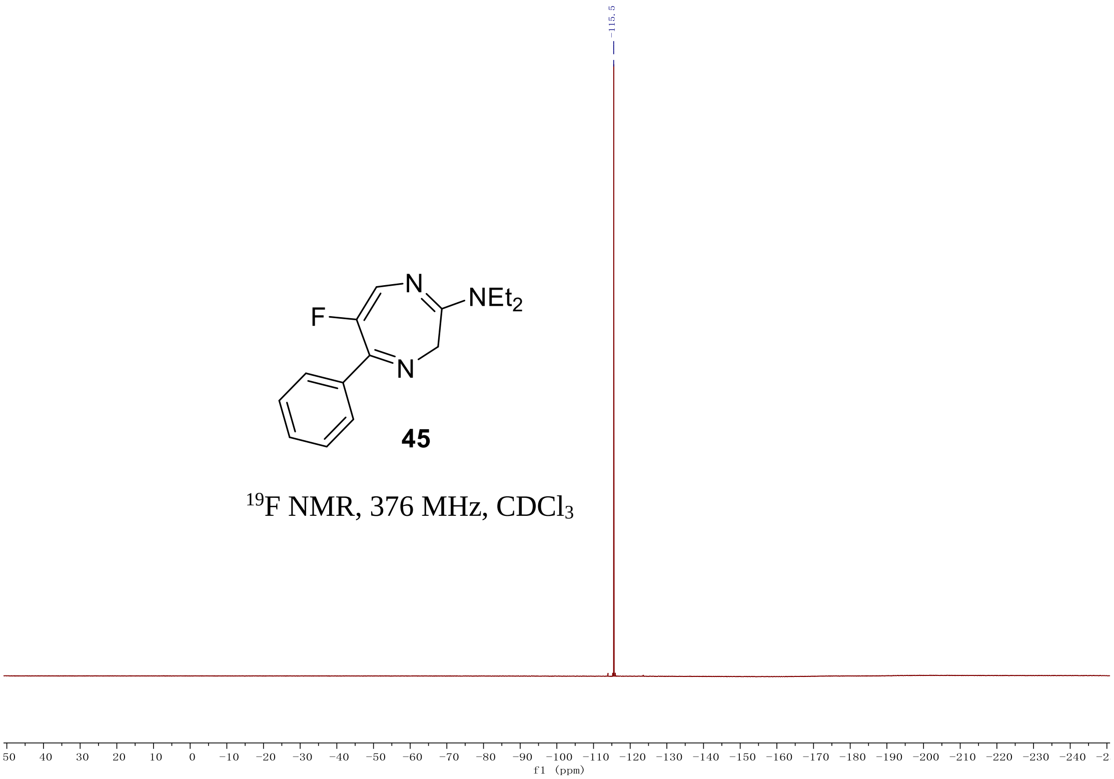
−115.5
F
N
NEt2
N
45
19F NMR, 376 MHz, CDCl3
50 40 30 20 10 0 −10 −20 −30 −40 −50 −60 −70 −80 −90 −100 −110 −120 −130 −140 −150 −160 −170 −180 −190 −200 −210 −220 −230 −240 −2
f1 (ppm)

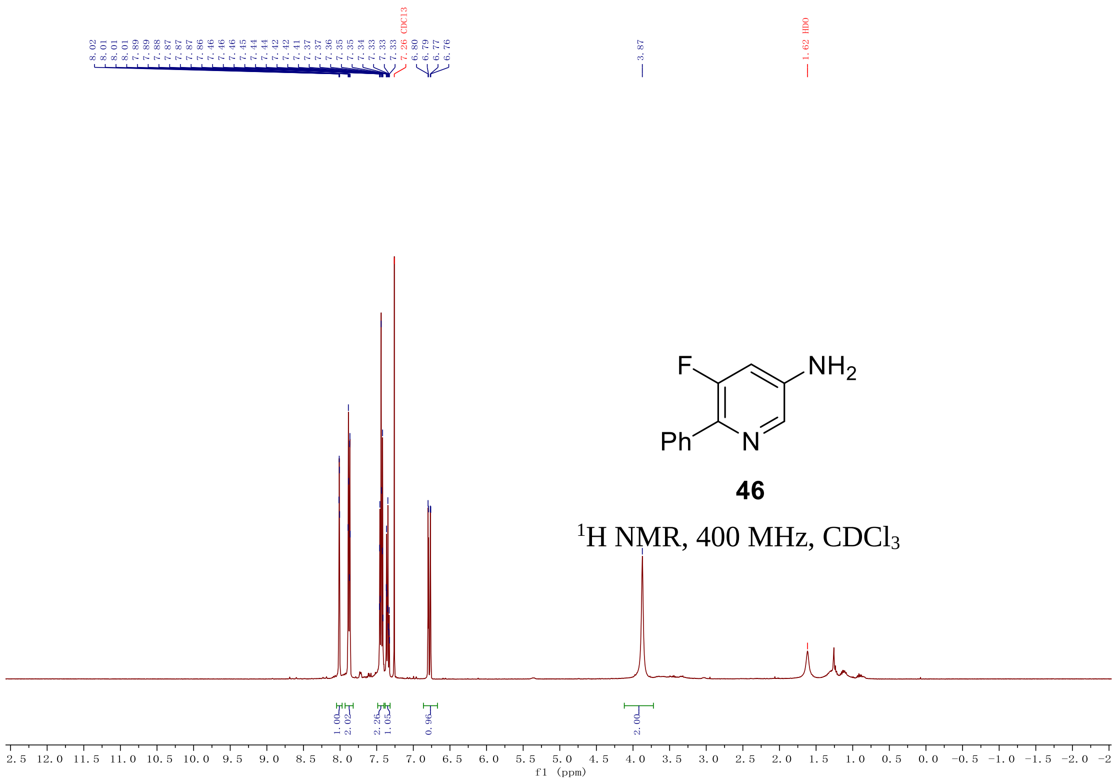
8.02
8.01
8.01
8.01
7.89
7.89
7.88
7.87
7.87
7.87
7.86
7.46
7.46
7.46
7.45
7.44
7.44
7.42
7.42
7.41
7.37
7.37
7.36
7.35
7.35
7.35
7.34
7.33
7.33
7.33
7.26 CDCl3
6.80
6.79
6.77
6.76
3.87
1.62 HDO
F
NH2
Ph
N
46
1H NMR, 400 MHz, CDCl3
1.00
2.02
2.26
1.03
0.96
2.00
f1 (ppm)

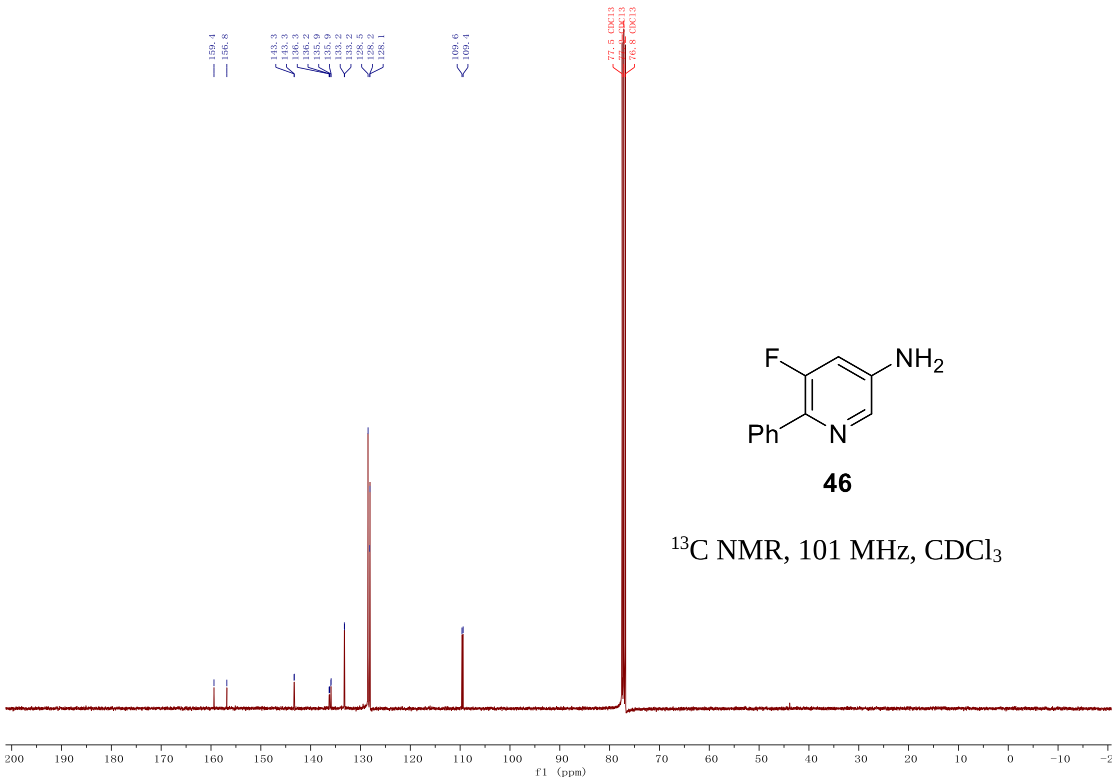
159.4
156.8
143.3
143.3
136.2
135.9
133.2
133.2
128.5
128.2
128.1
109.6
109.4
77.5 CDCl3
76.8 CDCl3
F
NH2
Ph
N
46
13C NMR, 101 MHz, CDCl3
f1 (ppm)

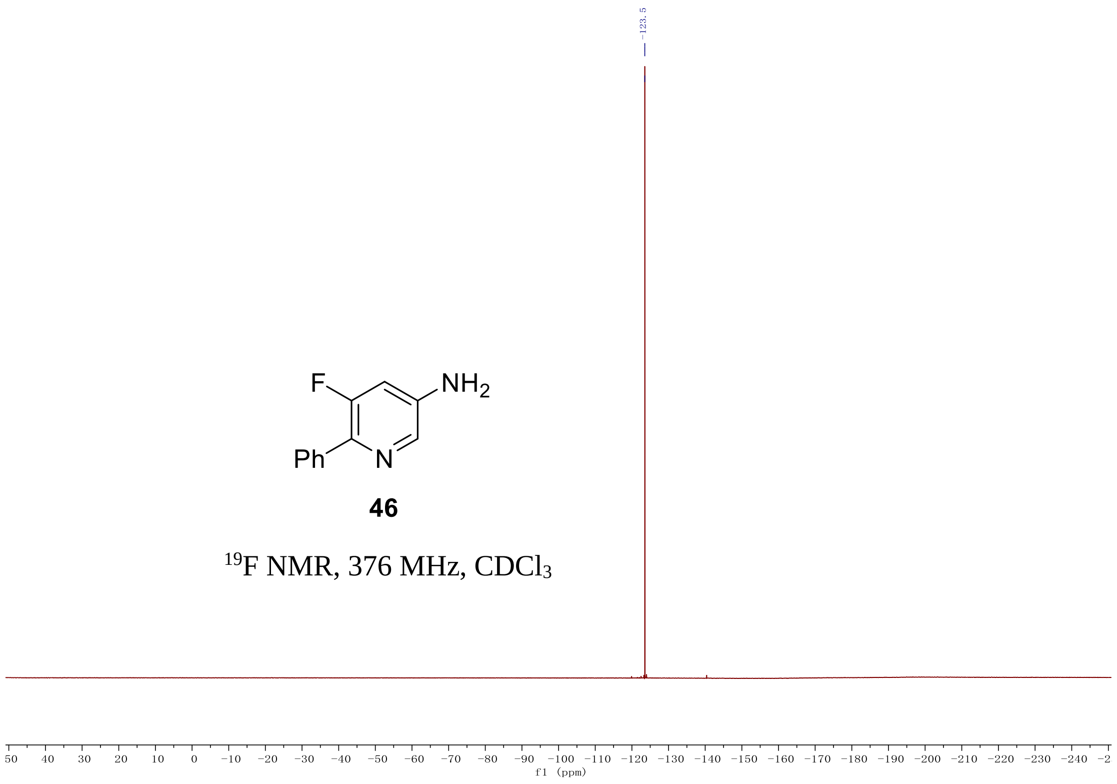

46

^{19}F NMR, 376 MHz, $CDCl_3$

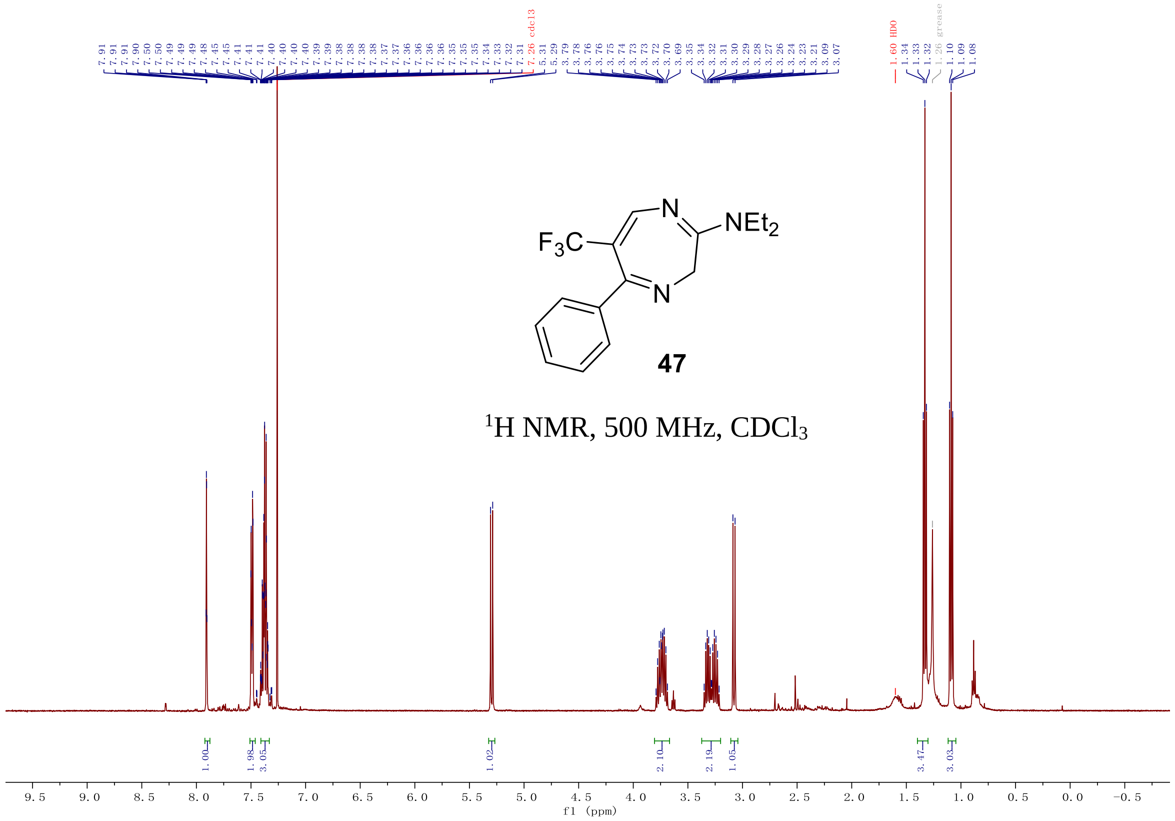

47

^{1}H NMR, 500 MHz, $CDCl_3$

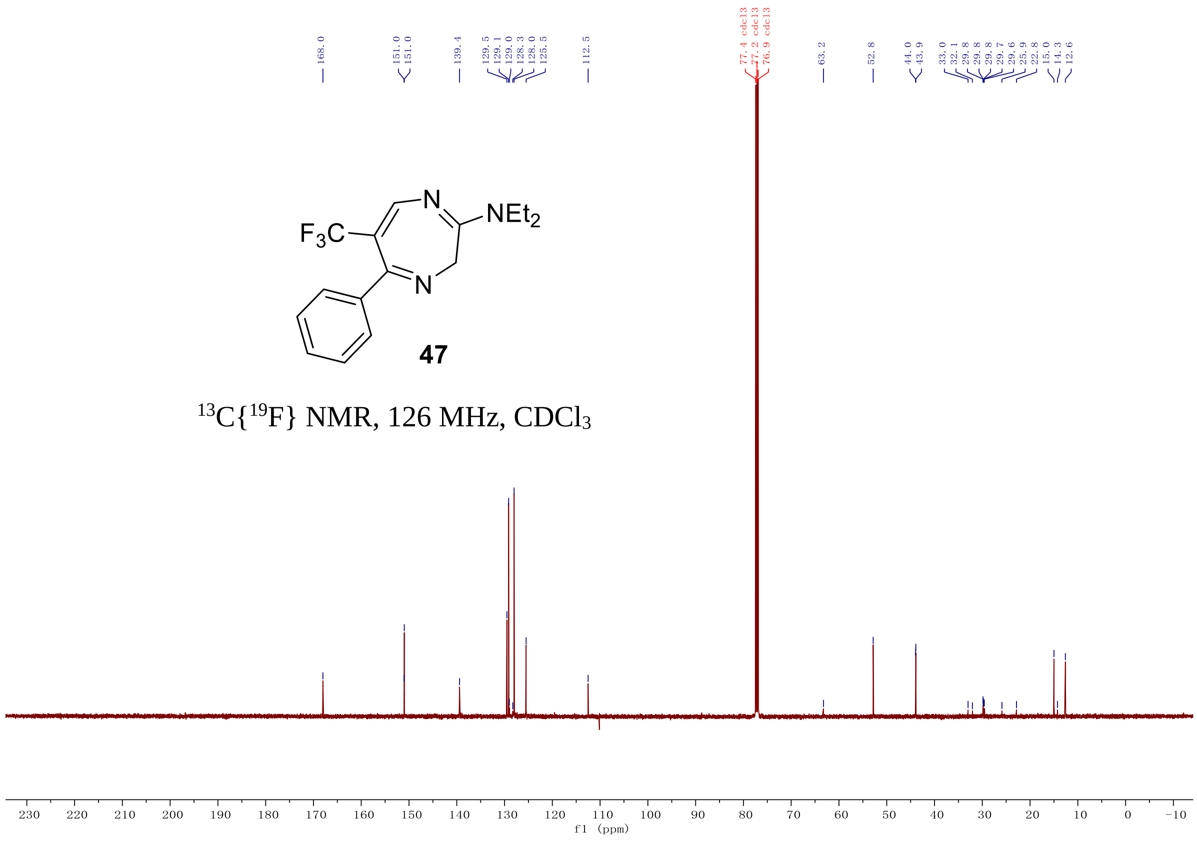

47

$^{13}C\{^{19}F\}$ NMR, 126 MHz, $CDCl_3$

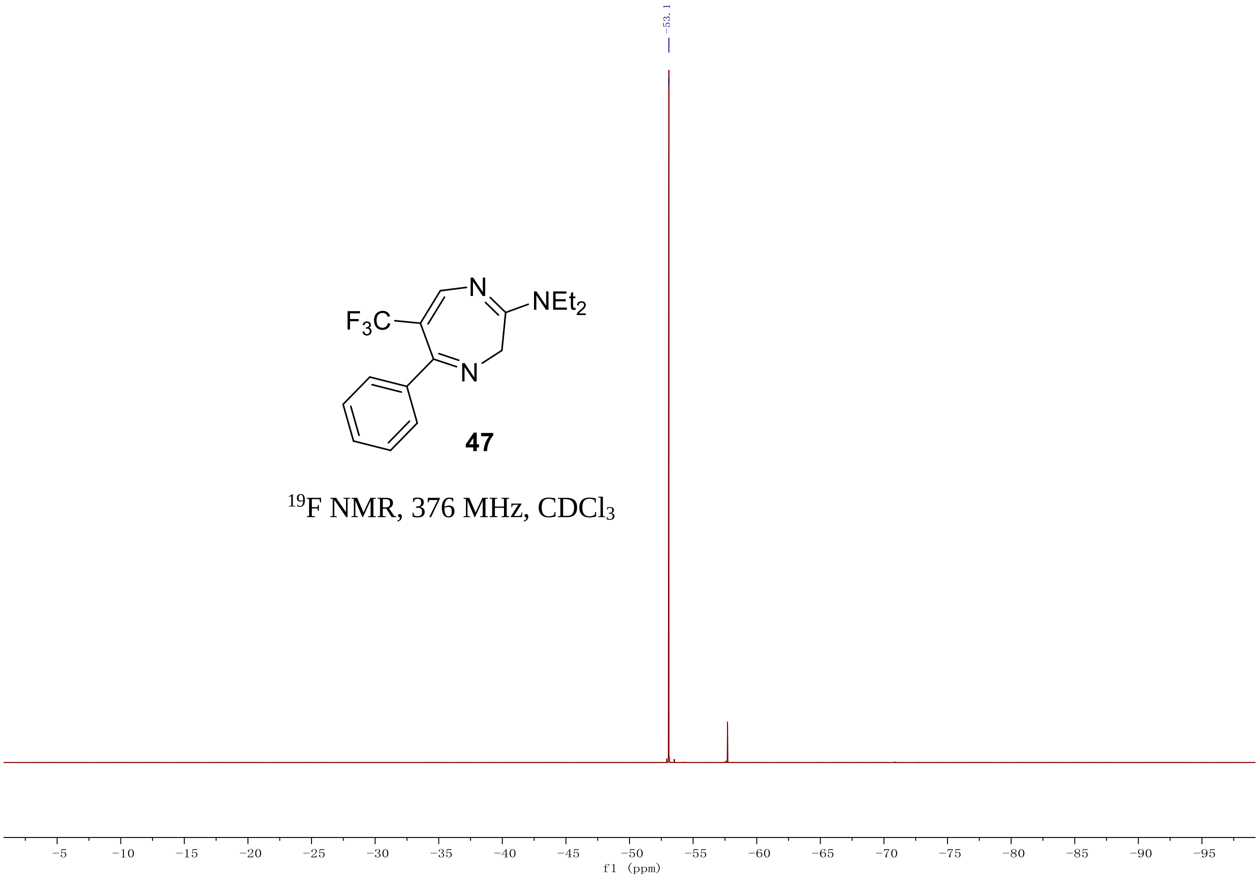

47

^{19}F NMR, 376 MHz, $CDCl_3$

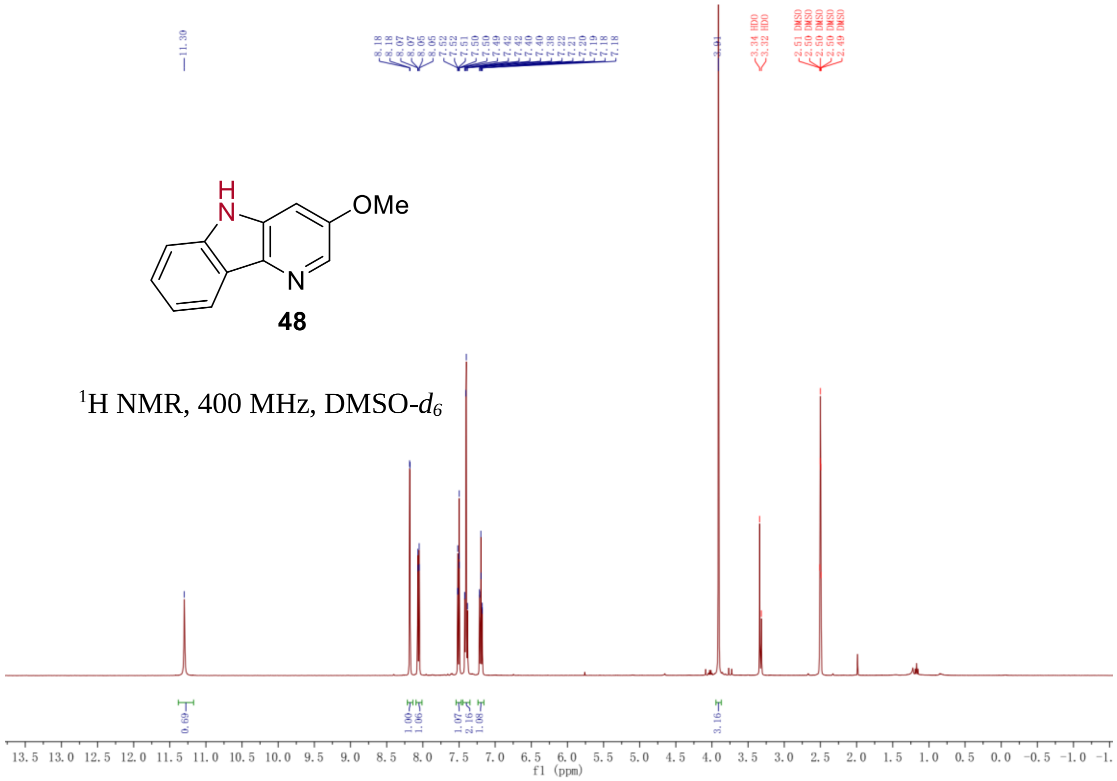

^{1}H NMR, 400 MHz, DMSO-d_6

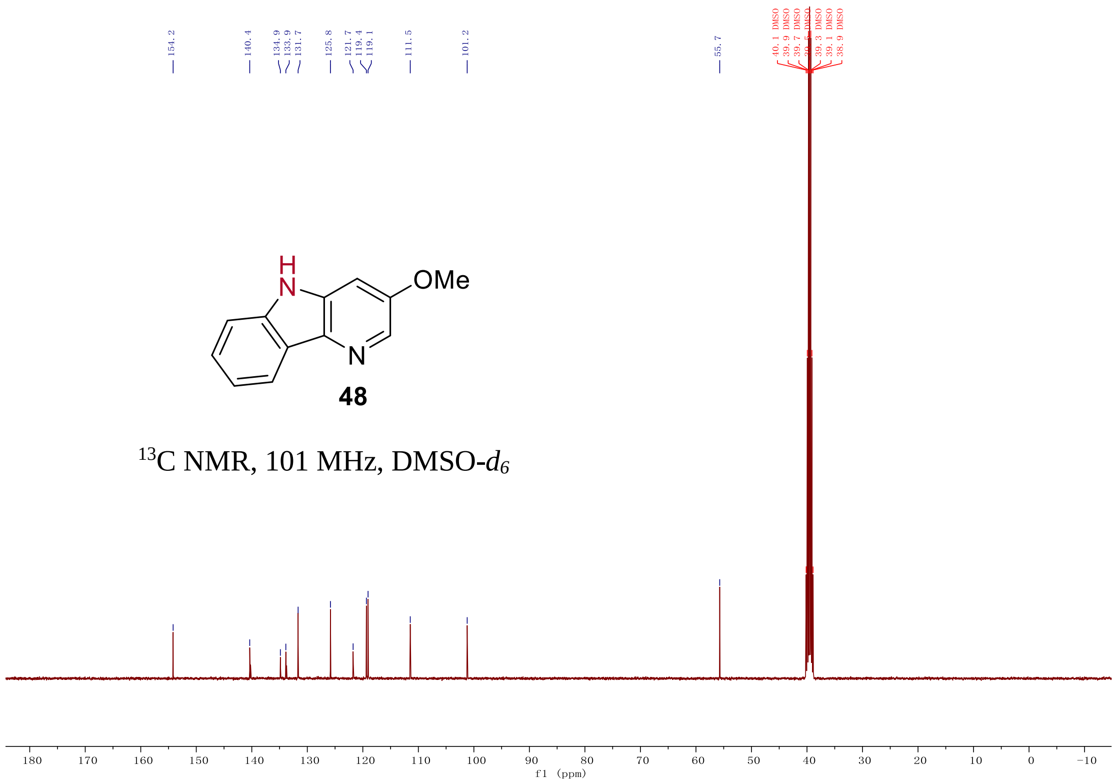

^{13}C NMR, 101 MHz, DMSO-d_6

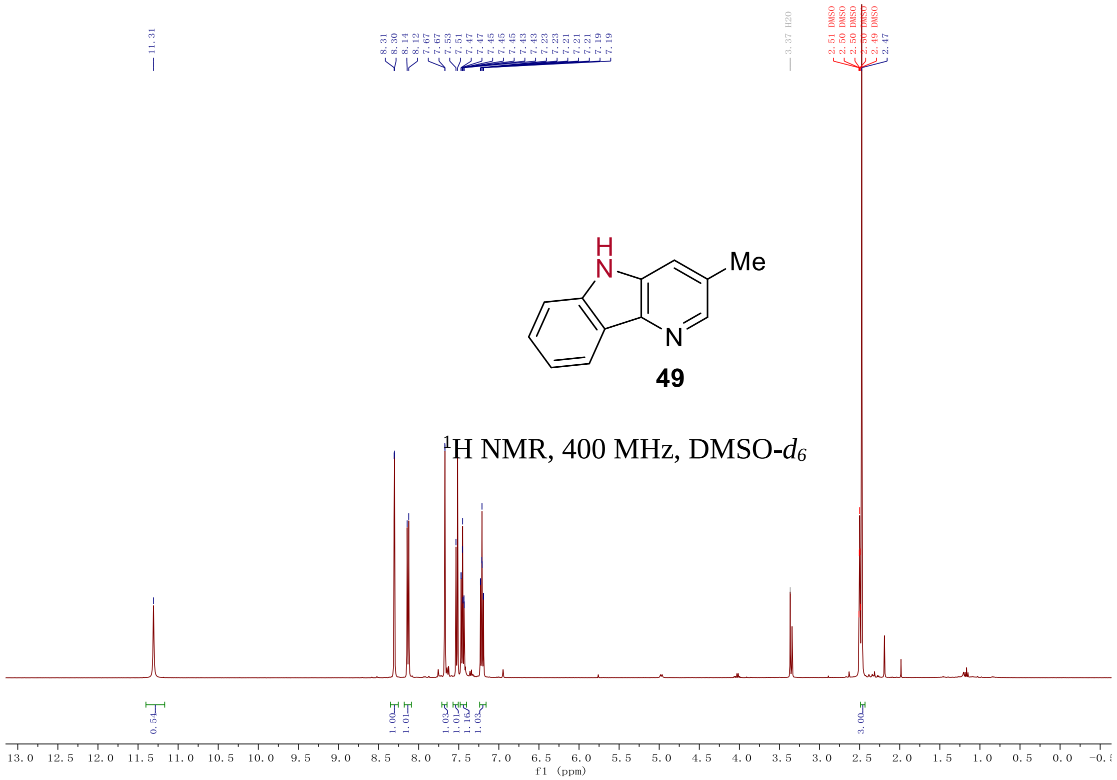
11.31
8.31
8.30
8.14
8.12
7.67
7.67
7.53
7.51
7.47
7.47
7.45
7.45
7.43
7.43
7.23
7.23
7.21
7.21
7.19
7.19
3.37 H2O
2.51 DMSO
2.50 DMSO
2.50 DMSO
2.50 DMSO
2.49 DMSO
2.47
Me
49
1H NMR, 400 MHz, DMSO-d6
0.54
1.00
1.01
1.03
1.01
1.16
1.03
3.00
f1 (ppm)

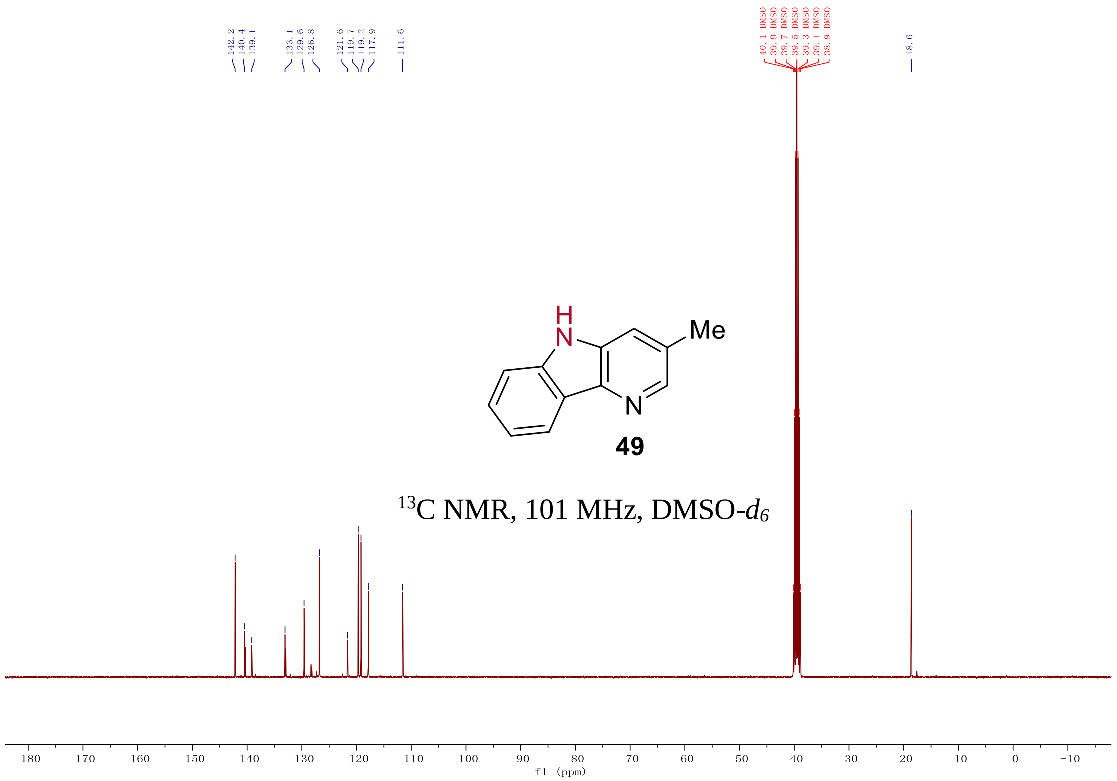
142.2
140.4
139.1
133.1
129.6
126.8
121.6
119.7
119.2
117.9
111.6
40.1 DMSO
39.9 DMSO
39.7 DMSO
39.5 DMSO
39.3 DMSO
39.1 DMSO
38.9 DMSO
18.6
Me
49
13C NMR, 101 MHz, DMSO-d6
f1 (ppm)

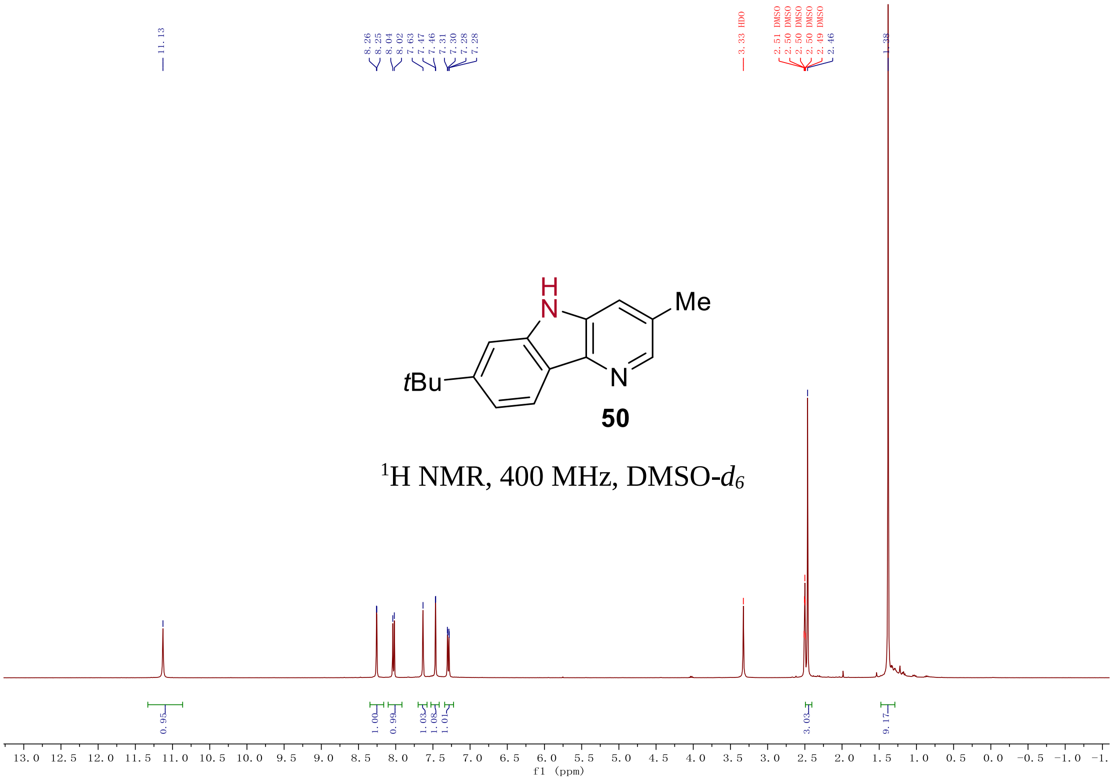
11.13
8.26 8.25 8.04 8.02 7.63 7.47 7.46 7.31 7.30 7.28 7.28
3.33 HDO
2.51 DMSO 2.50 DMSO 2.50 DMSO 2.50 DMSO 2.49 DMSO 2.46
1.38
H
N
Me
N
tBu
50
^{1}H NMR, 400 MHz, DMSO-d_6
0.95 1.00 0.99 1.03 1.08 1.01 3.03 9.17
f1 (ppm)

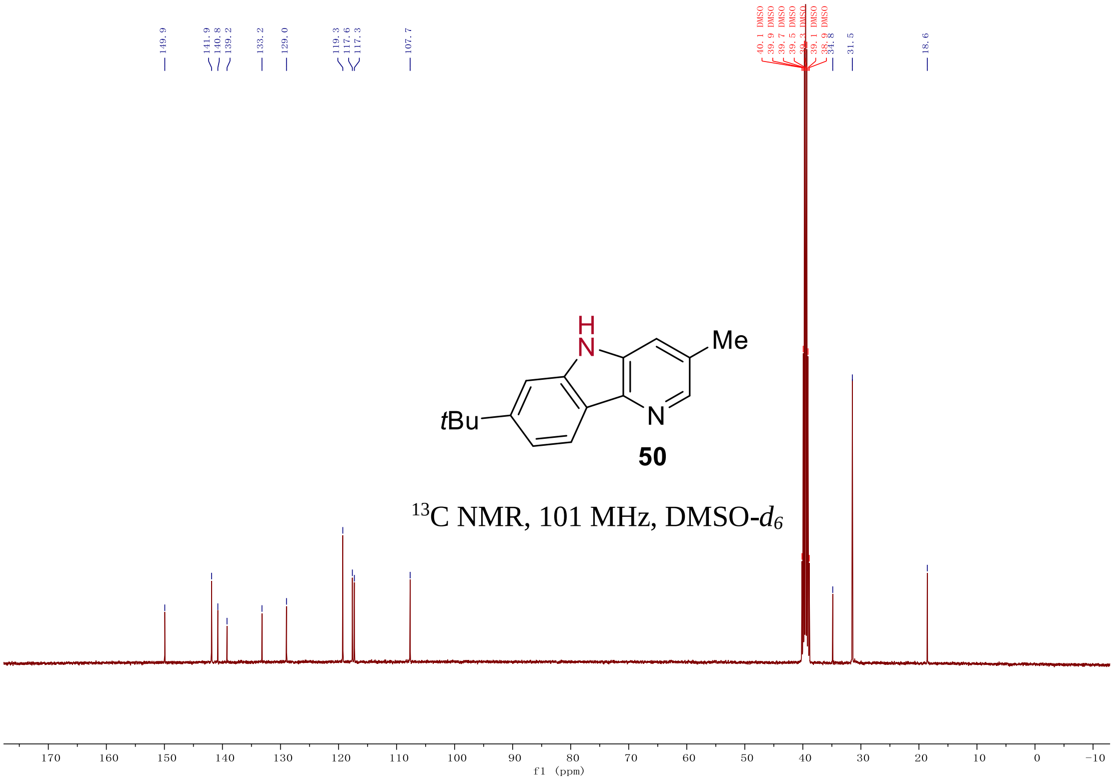
149.9
141.9 140.8 139.2
133.2
129.0
119.3 117.6 117.3
107.7
40.1 DMSO 39.9 DMSO 39.7 DMSO 39.5 DMSO 39.3 DMSO 39.1 DMSO 38.9 DMSO
34.8
31.5
18.6
H
N
Me
N
tBu
50
^{13}C NMR, 101 MHz, DMSO-d_6
f1 (ppm)

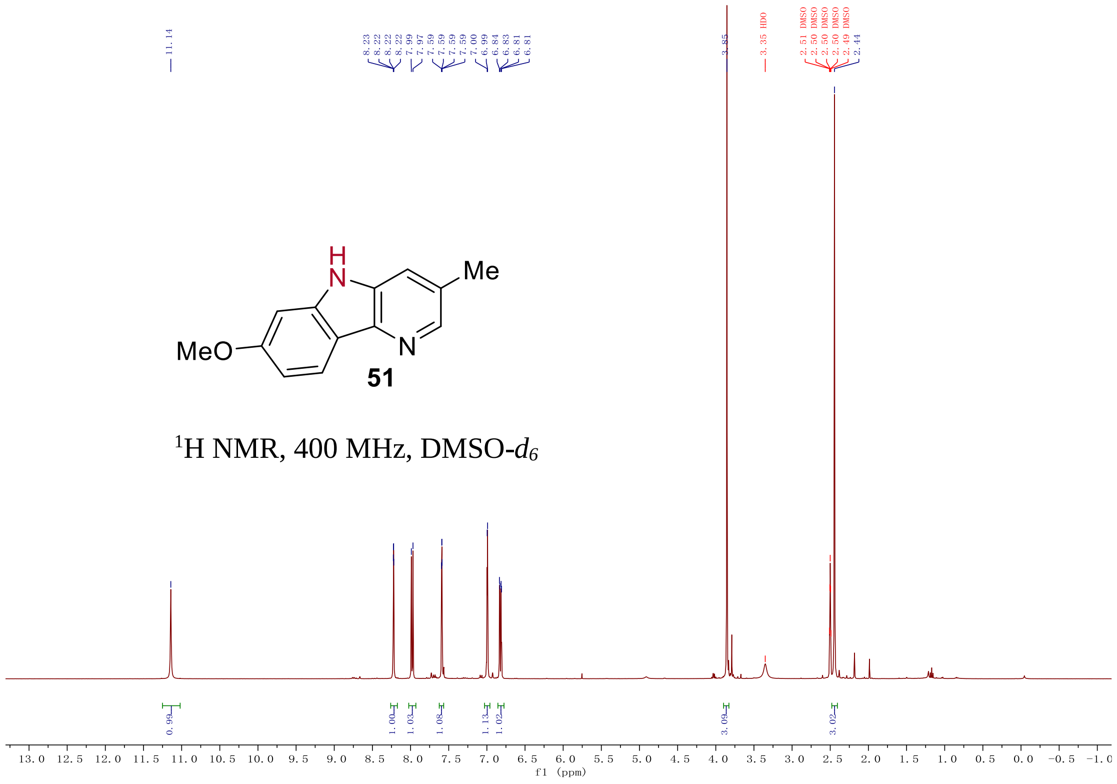

^{1}H NMR, 400 MHz, DMSO-d_6

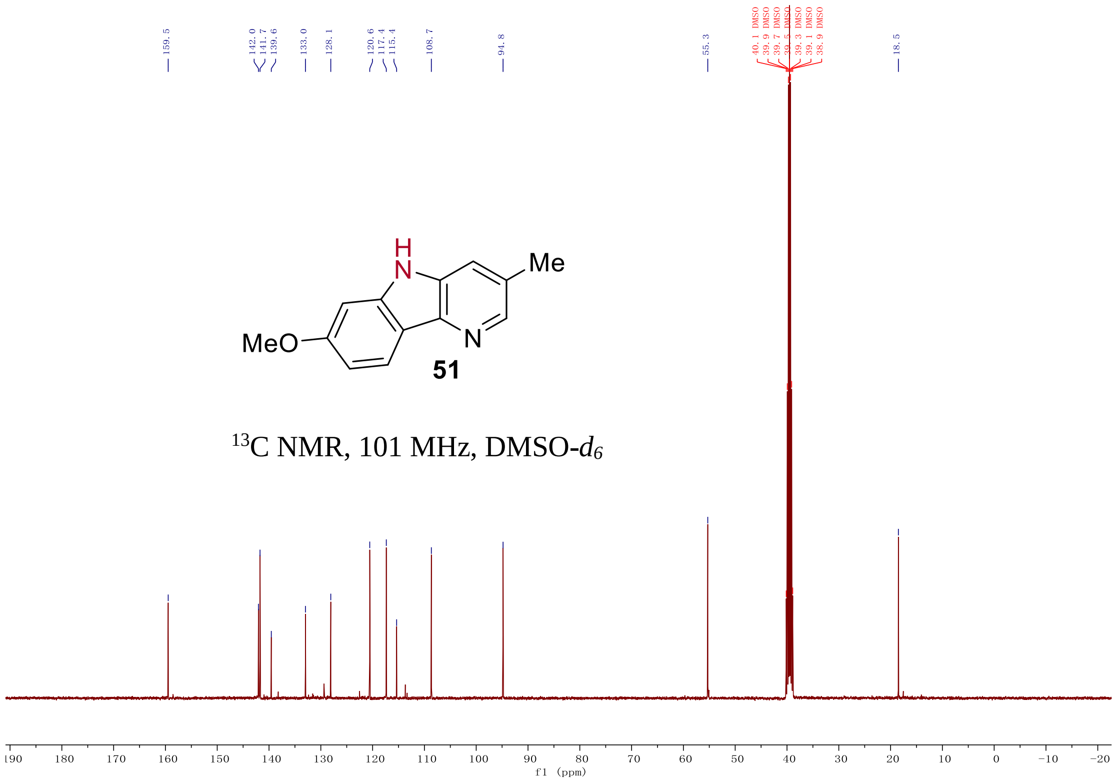

^{13}C NMR, 101 MHz, DMSO-d_6

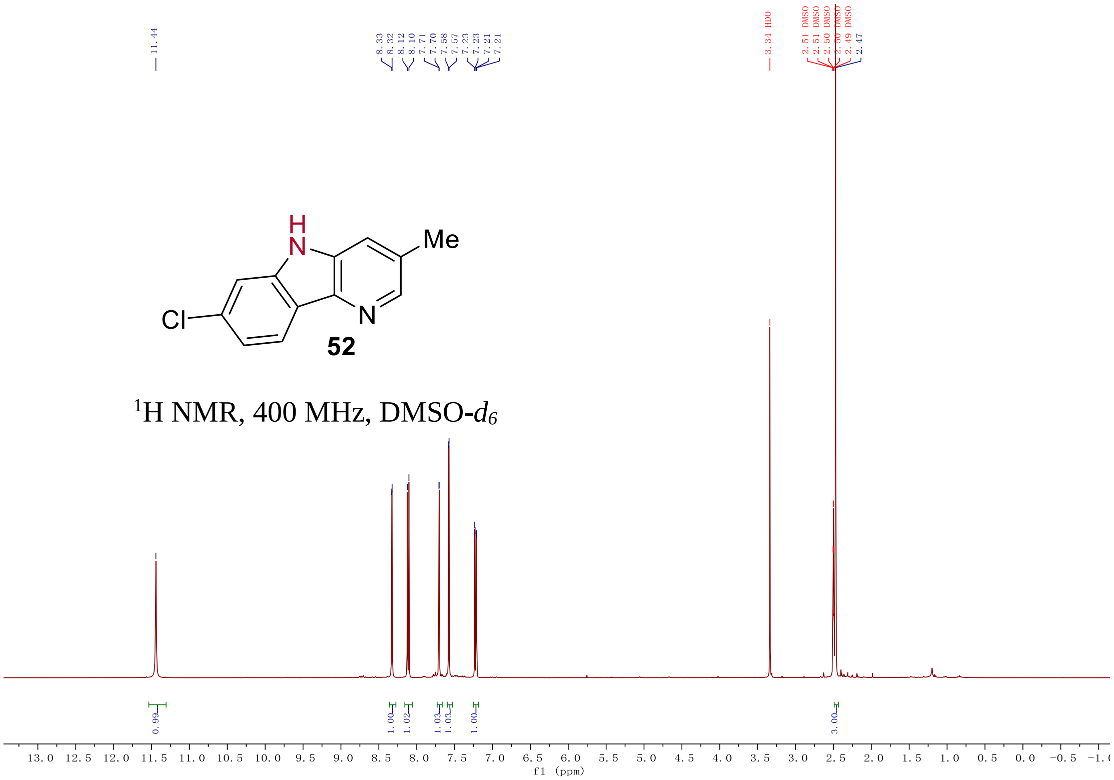

^{1}H NMR, 400 MHz, DMSO-d_6

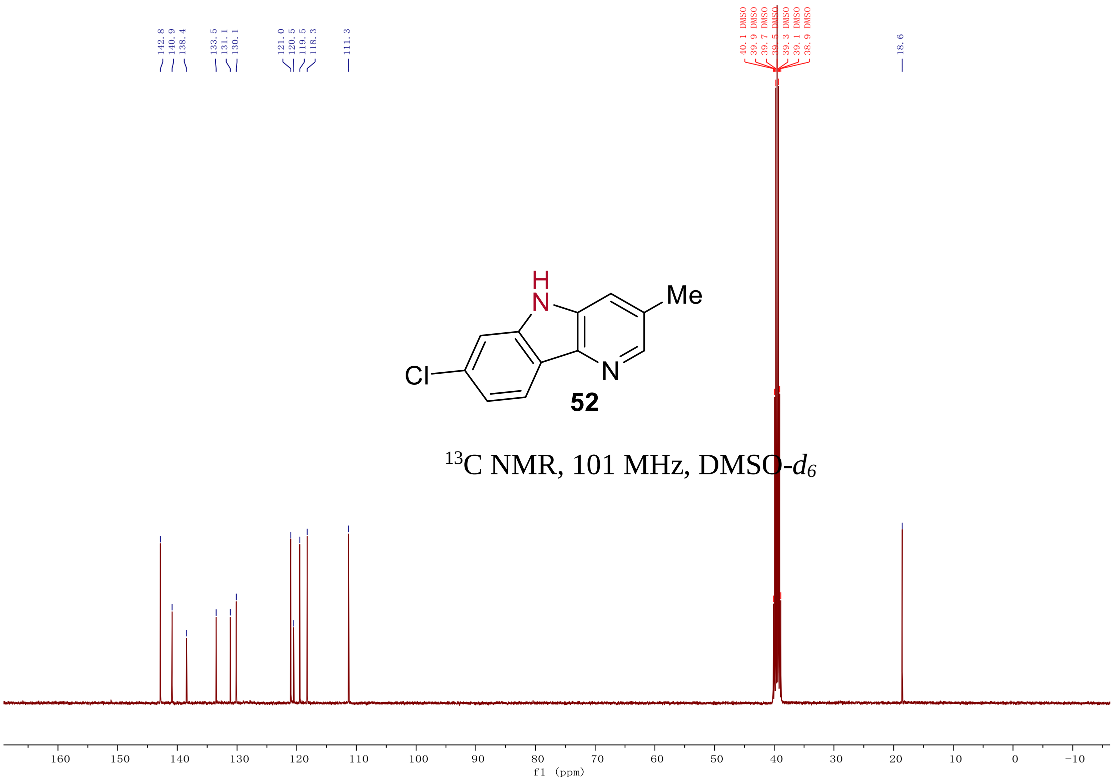

^{13}C NMR, 101 MHz, DMSO-d_6

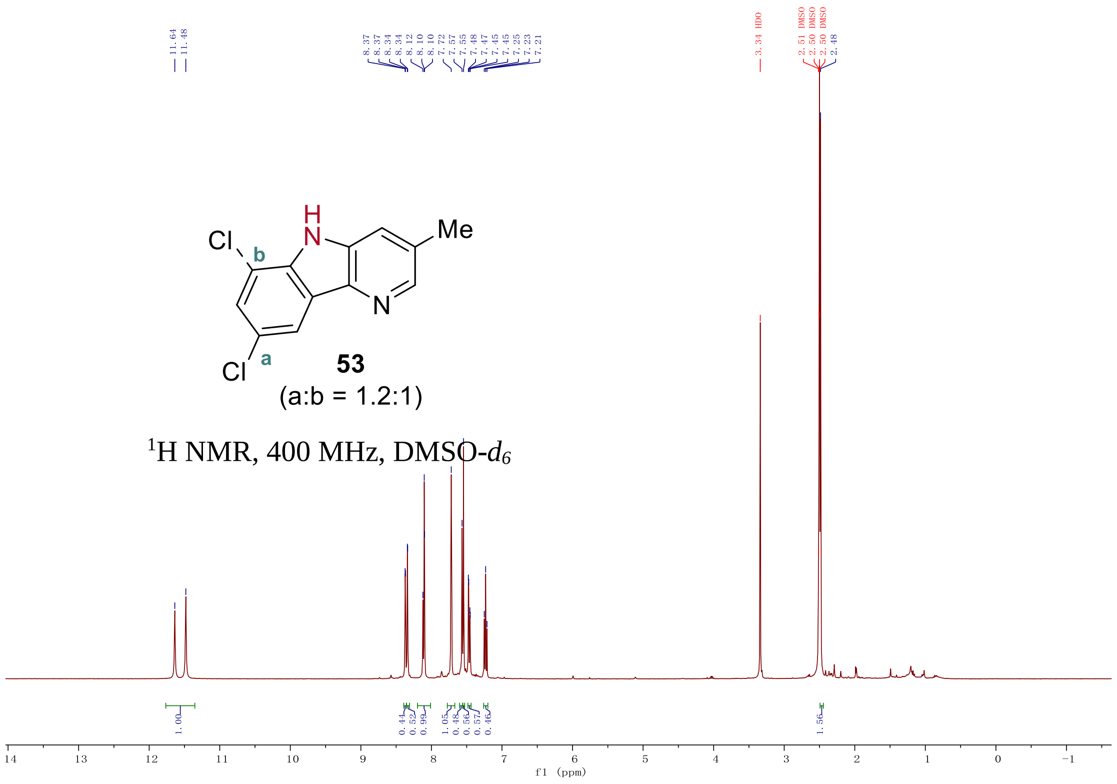

53
(a:b = 1.2:1)
^{1}H NMR, 400 MHz, DMSO-d_6
f1 (ppm)

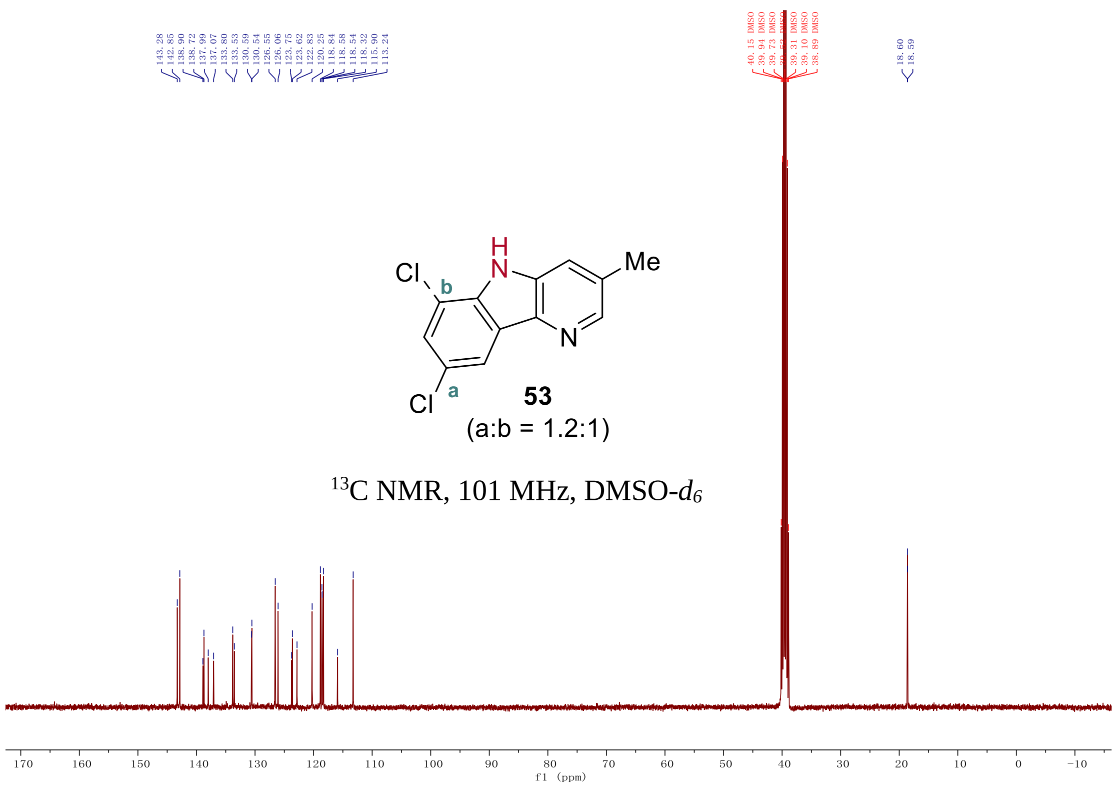

53
(a:b = 1.2:1)
^{13}C NMR, 101 MHz, DMSO-d_6
f1 (ppm)

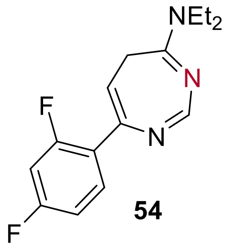

54

^1H NMR, 500 MHz, $CDCl_3$

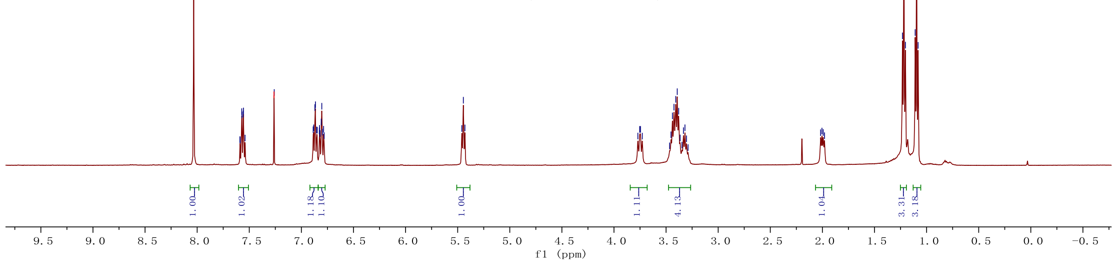

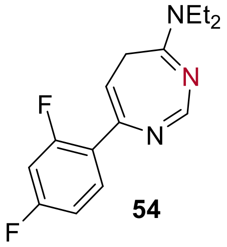

54

^{13}C NMR, 126 MHz, $CDCl_3$

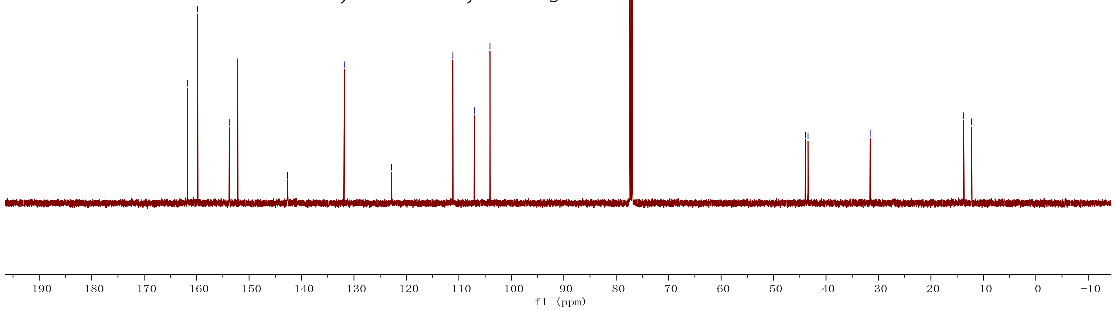

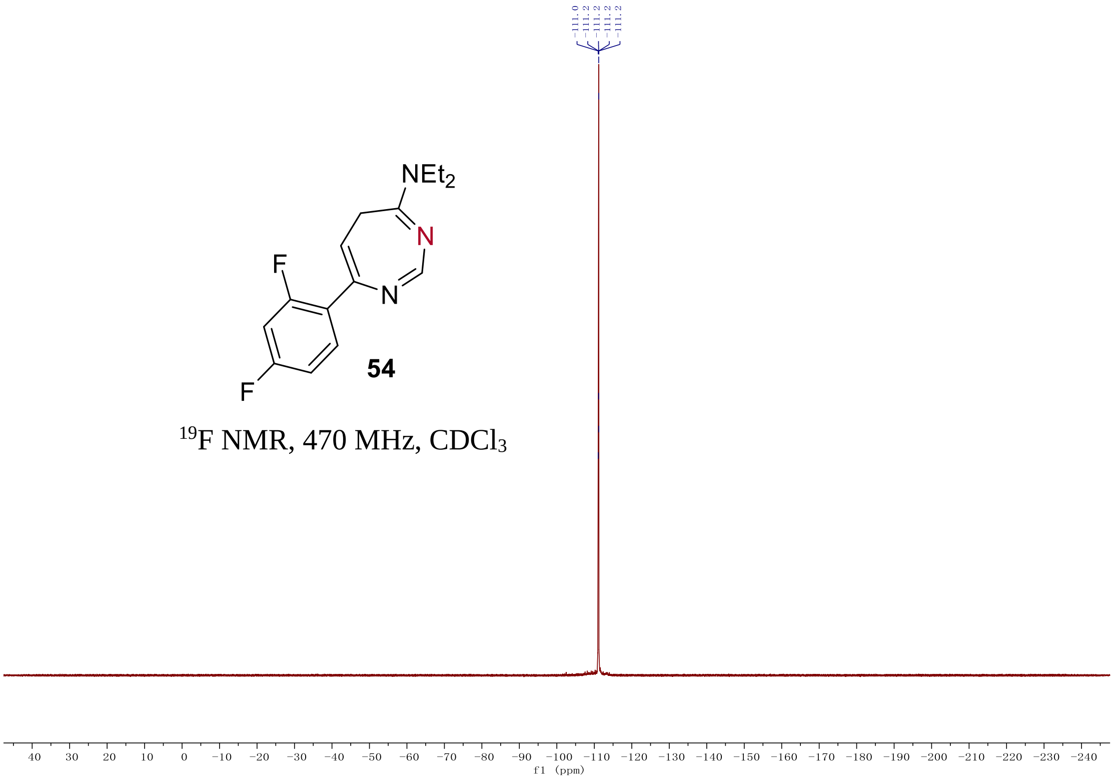

54

^{19}F NMR, 470 MHz, $CDCl_3$

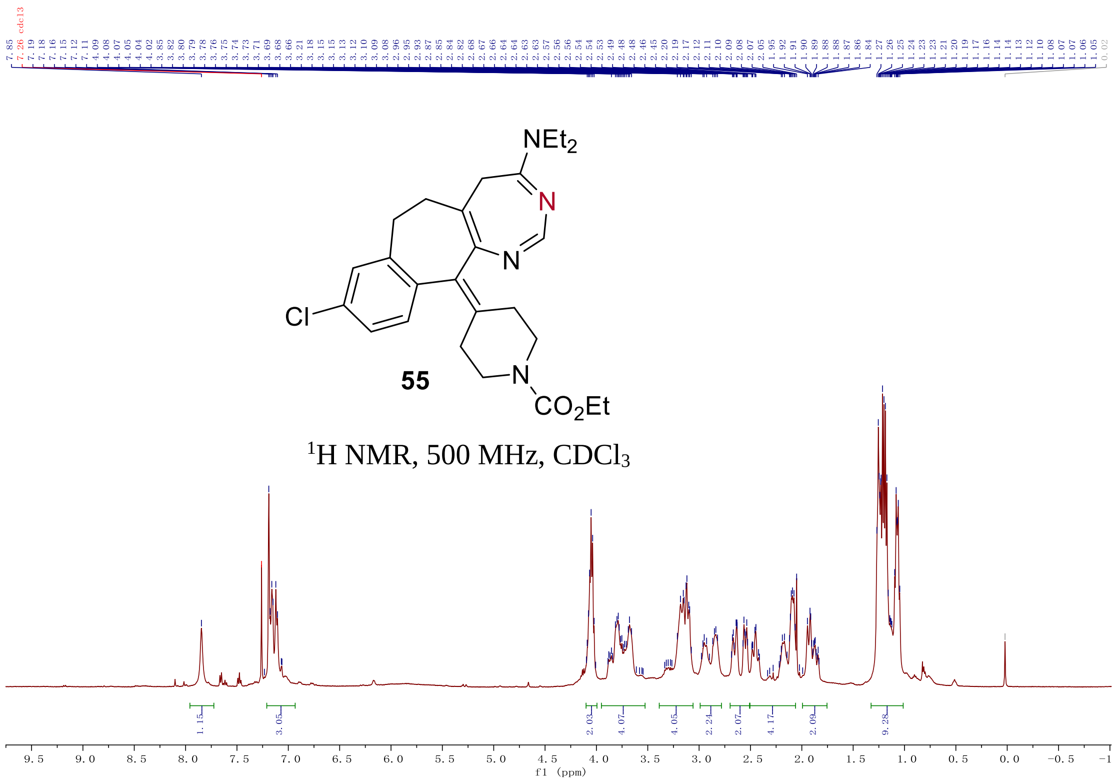

55

^{1}H NMR, 500 MHz, $CDCl_3$

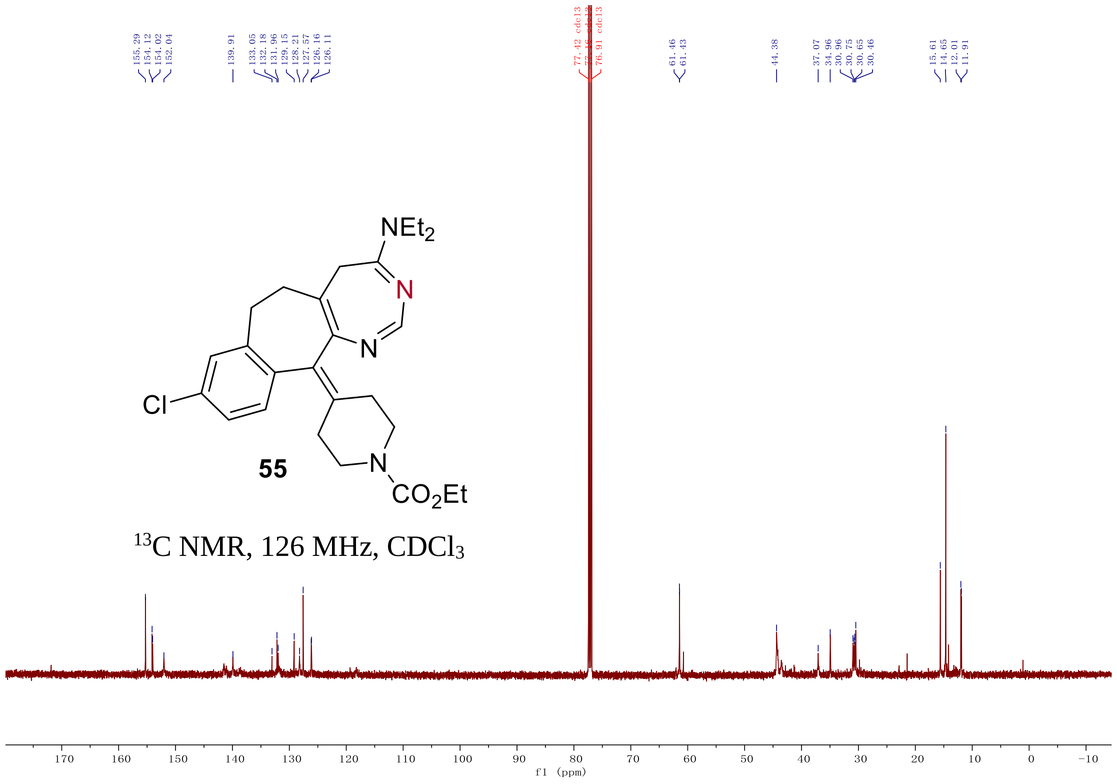

55

^{13}C NMR, 126 MHz, $CDCl_3$

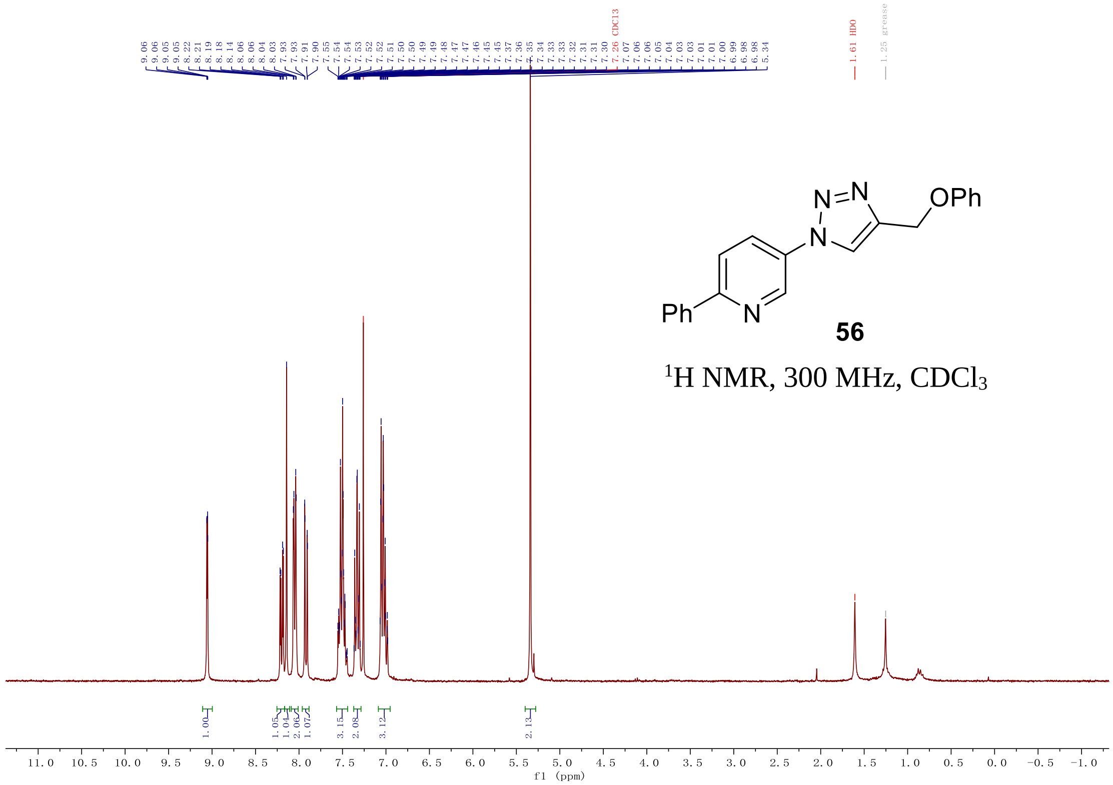

56
^{1}H NMR, 300 MHz, CDCl$_3$

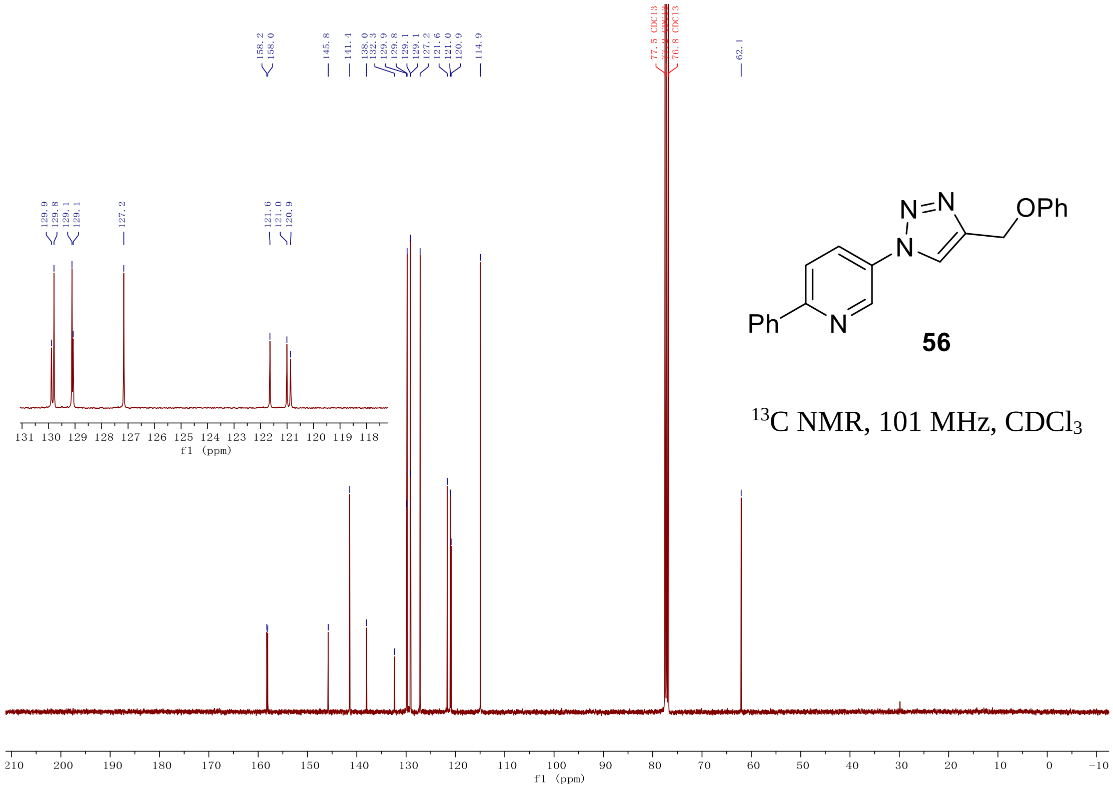

56
^{13}C NMR, 101 MHz, CDCl$_3$

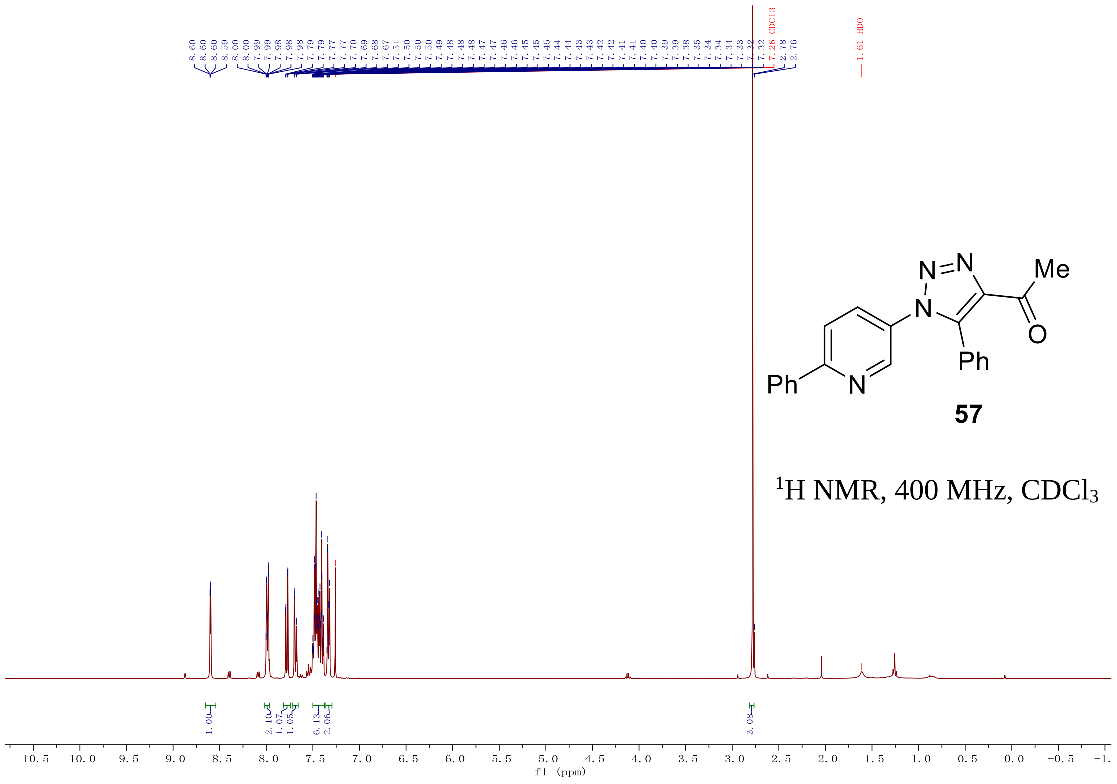
1H NMR, 400 MHz, CDCl3
57
Ph
N
N
N=N
Me
O
Ph
f1 (ppm)

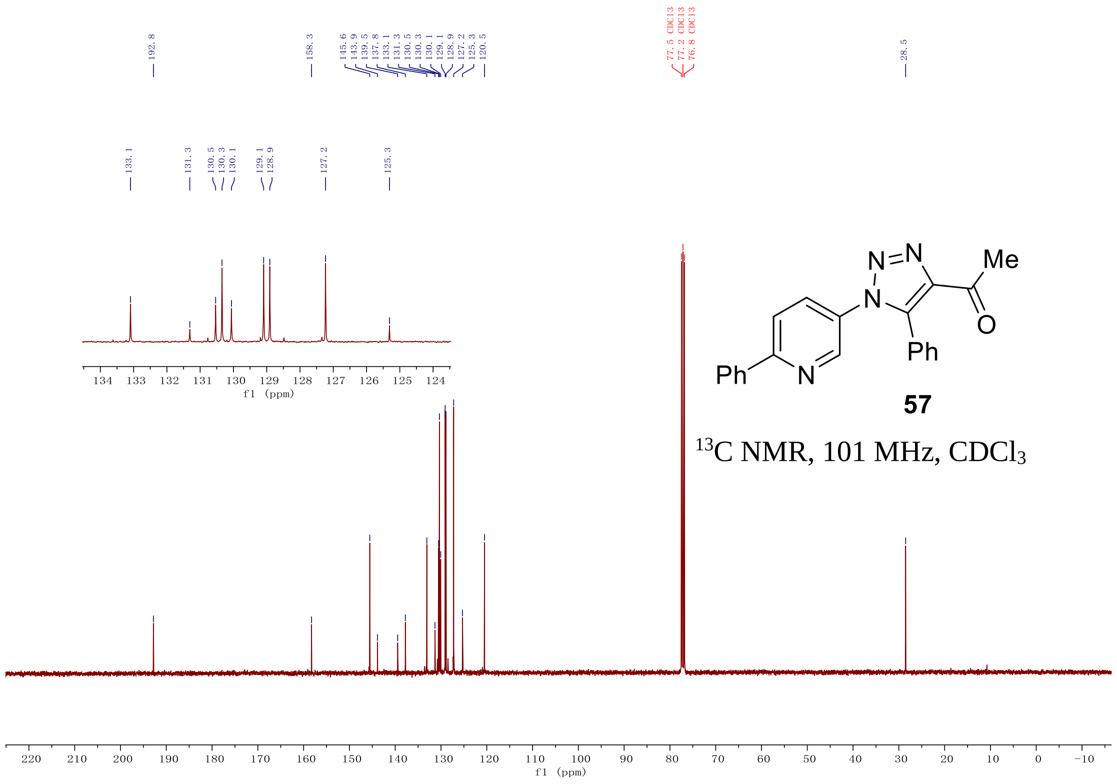
13C NMR, 101 MHz, CDCl3
57
192.8
158.3
28.5
f1 (ppm)

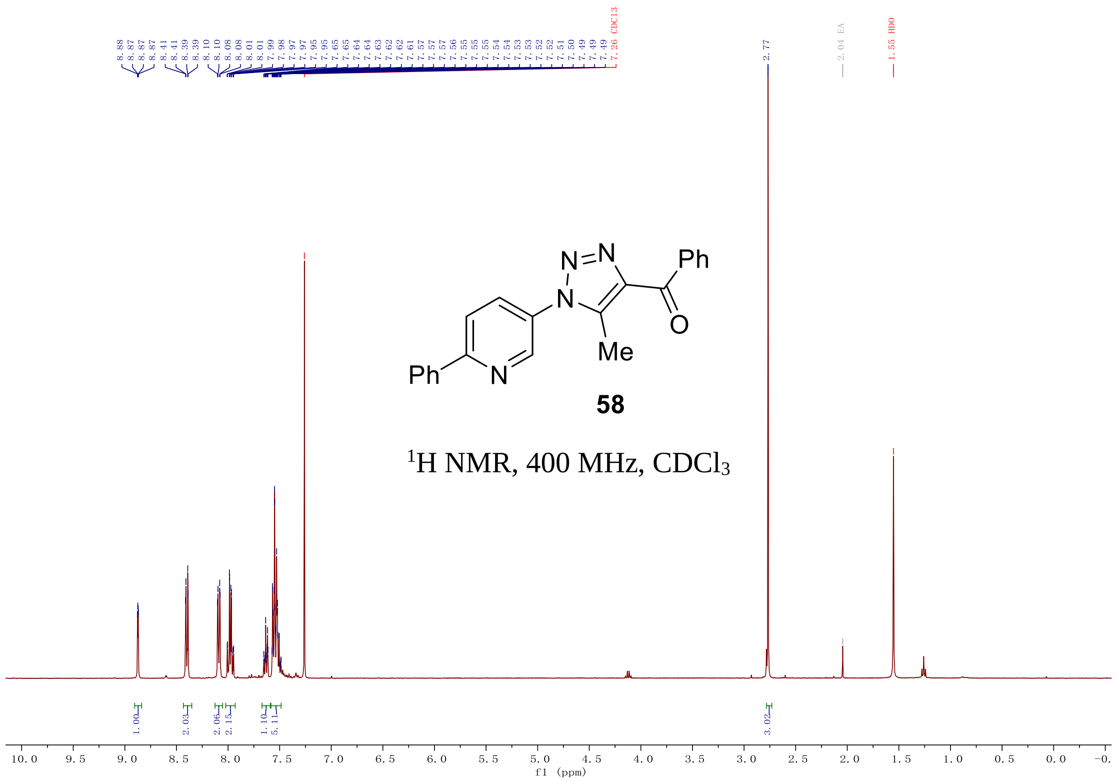

58

^{1}H NMR, 400 MHz, $CDCl_3$

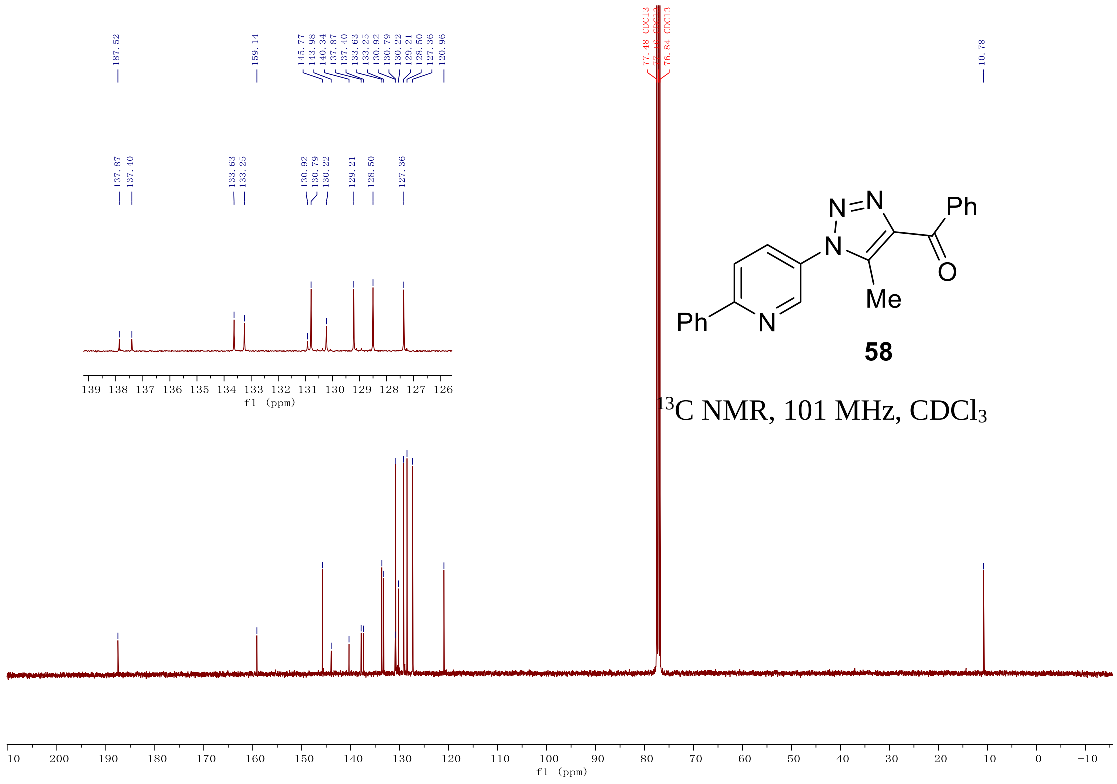

58

^{13}C NMR, 101 MHz, $CDCl_3$

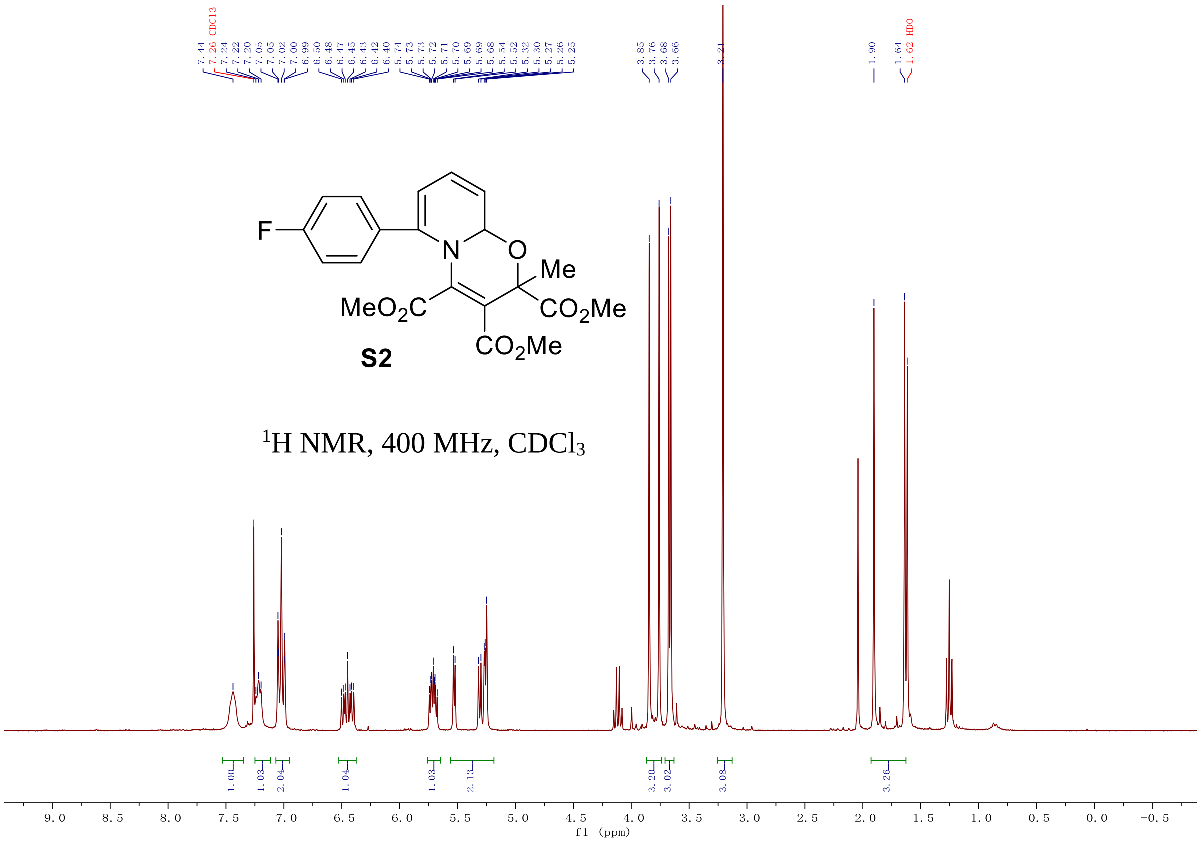
F
N
O
Me
MeO2C
CO2Me
CO2Me
S2
1H NMR, 400 MHz, CDCl3
f1 (ppm)

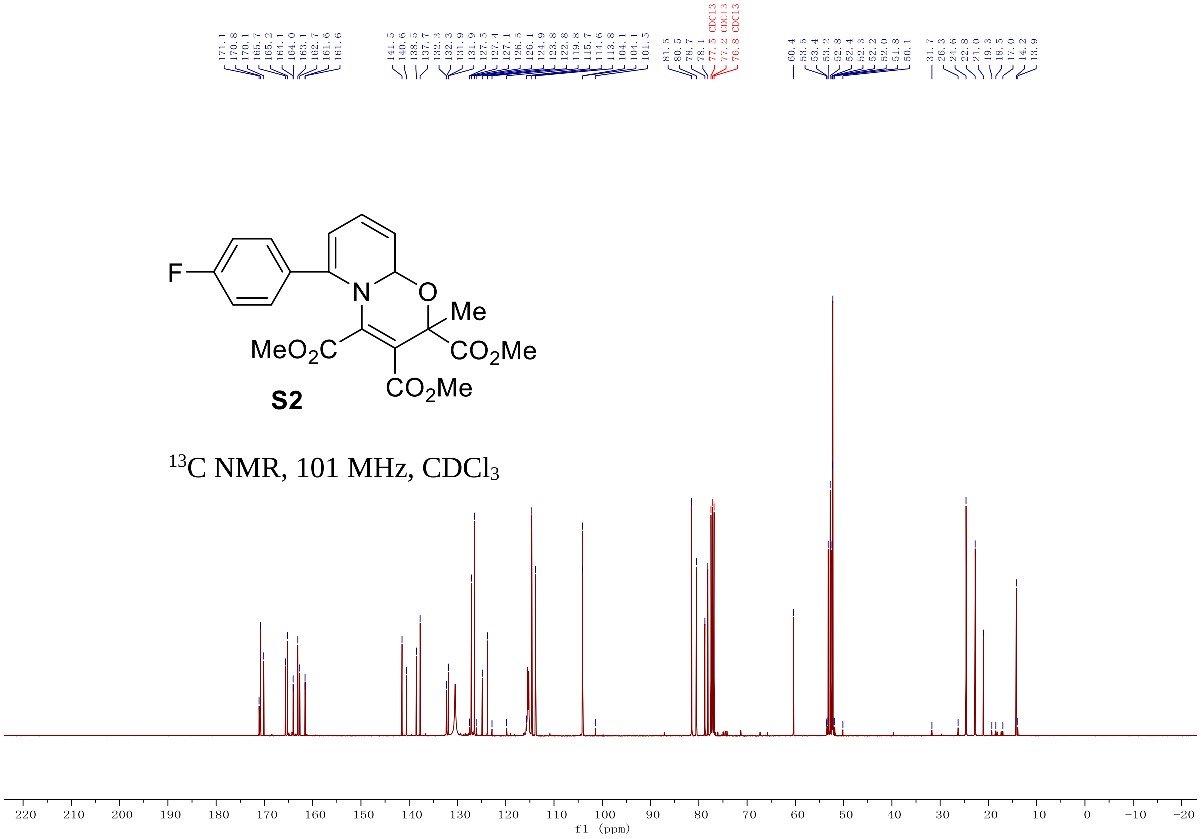
F
N
O
Me
MeO2C
CO2Me
CO2Me
S2
13C NMR, 101 MHz, CDCl3
f1 (ppm)

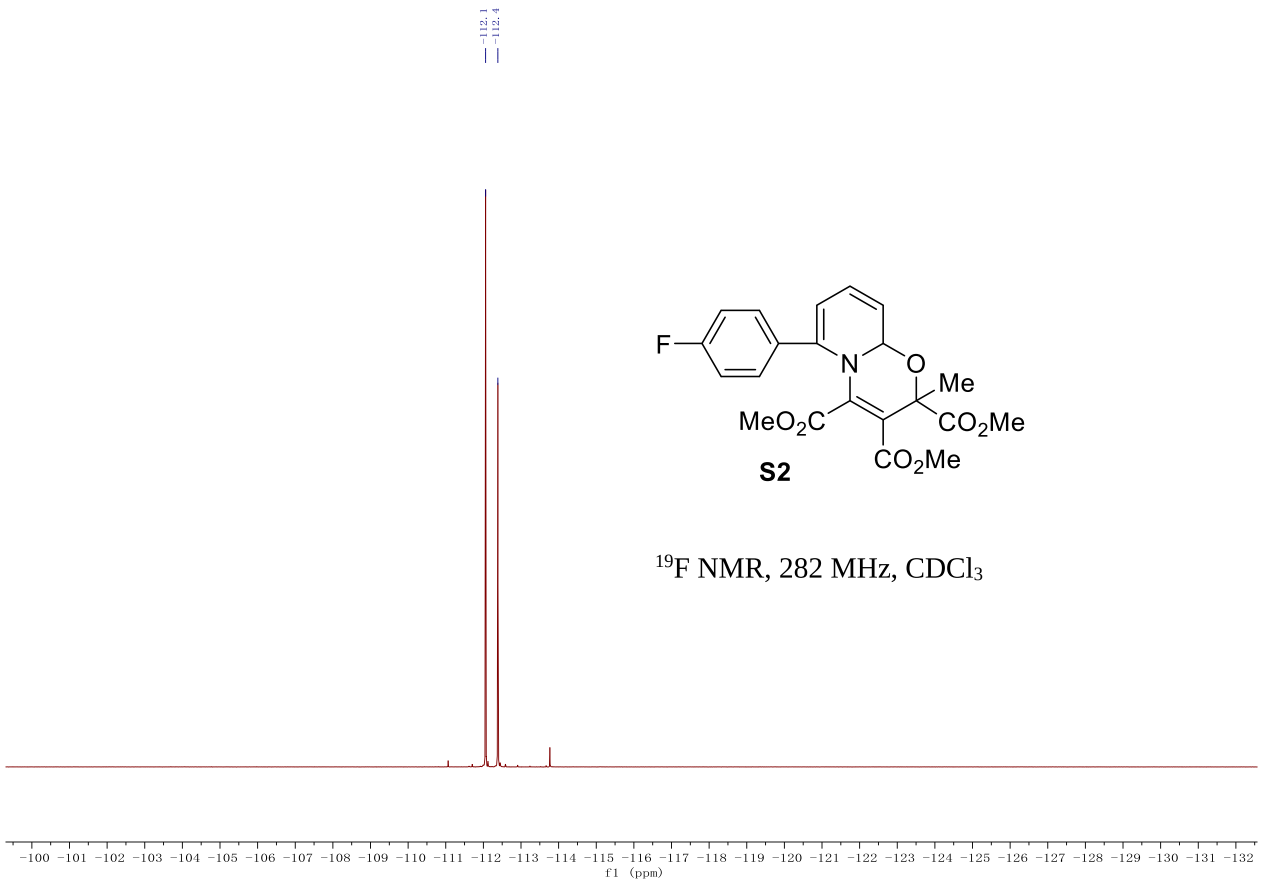

S2

^{19}F NMR, 282 MHz, $CDCl_3$

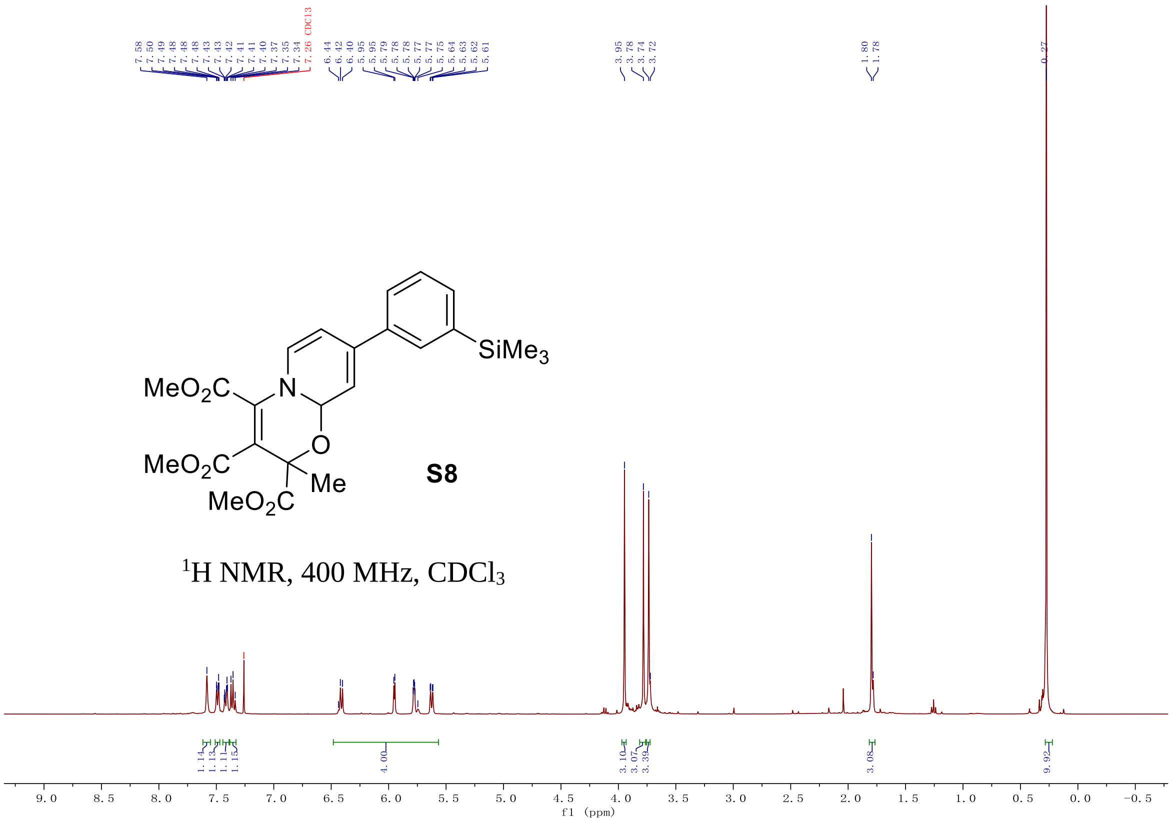

^{1}H NMR, 400 MHz, $CDCl_3$

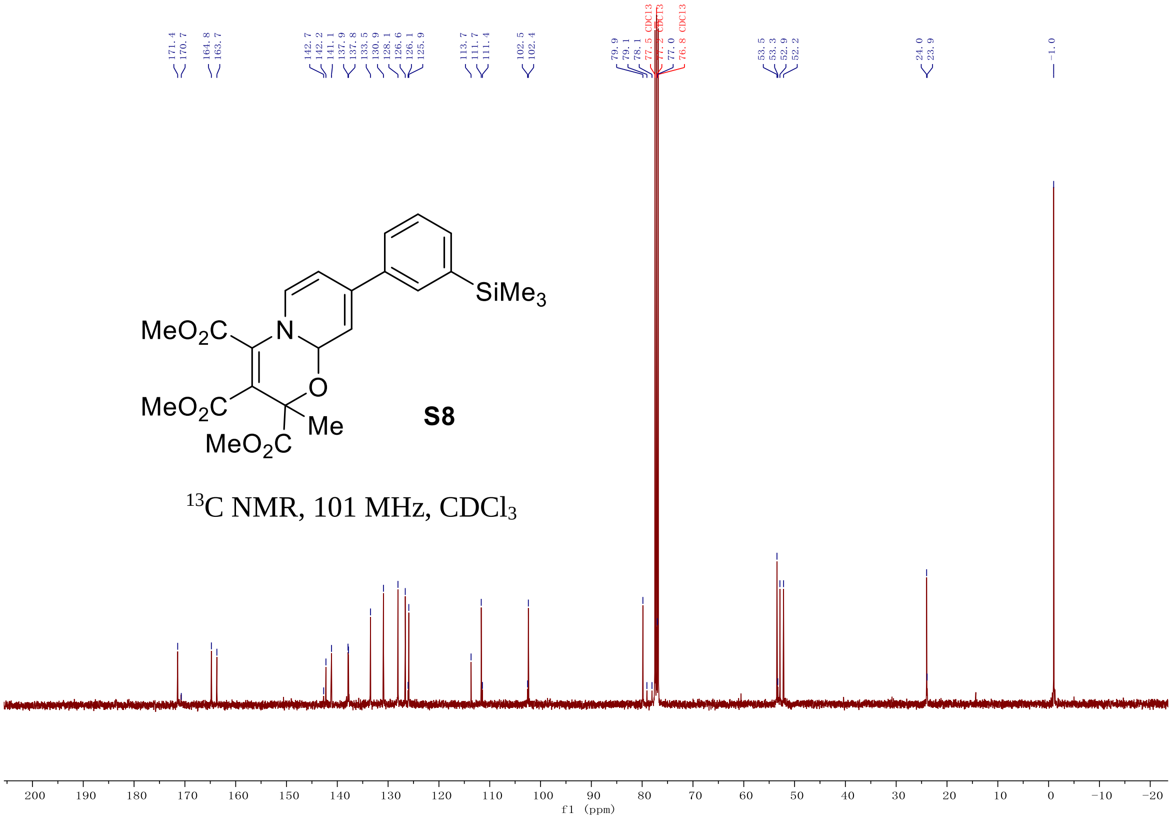

^{13}C NMR, 101 MHz, $CDCl_3$

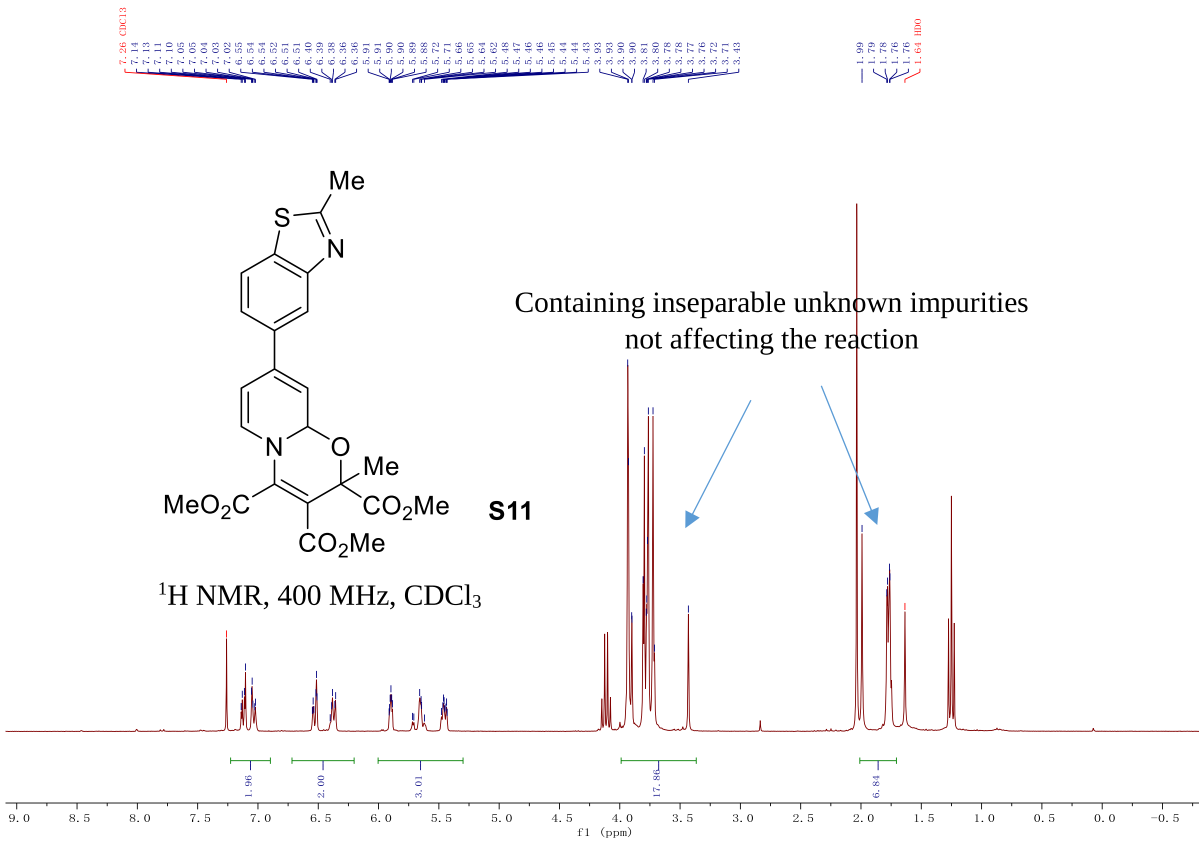
S11
¹H NMR, 400 MHz, CDCl₃
Containing inseparable unknown impurities
not affecting the reaction
f1 (ppm)

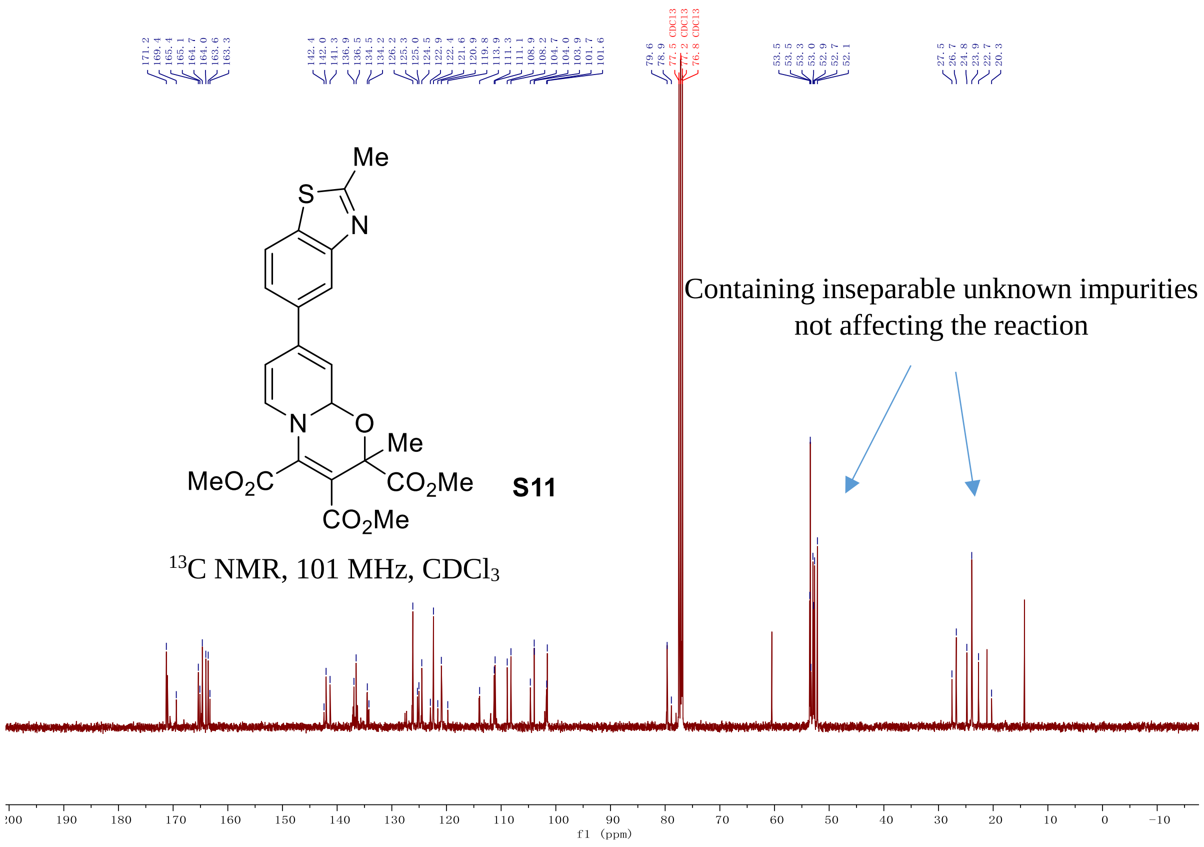
S11
¹³C NMR, 101 MHz, CDCl₃
Containing inseparable unknown impurities
not affecting the reaction
f1 (ppm)

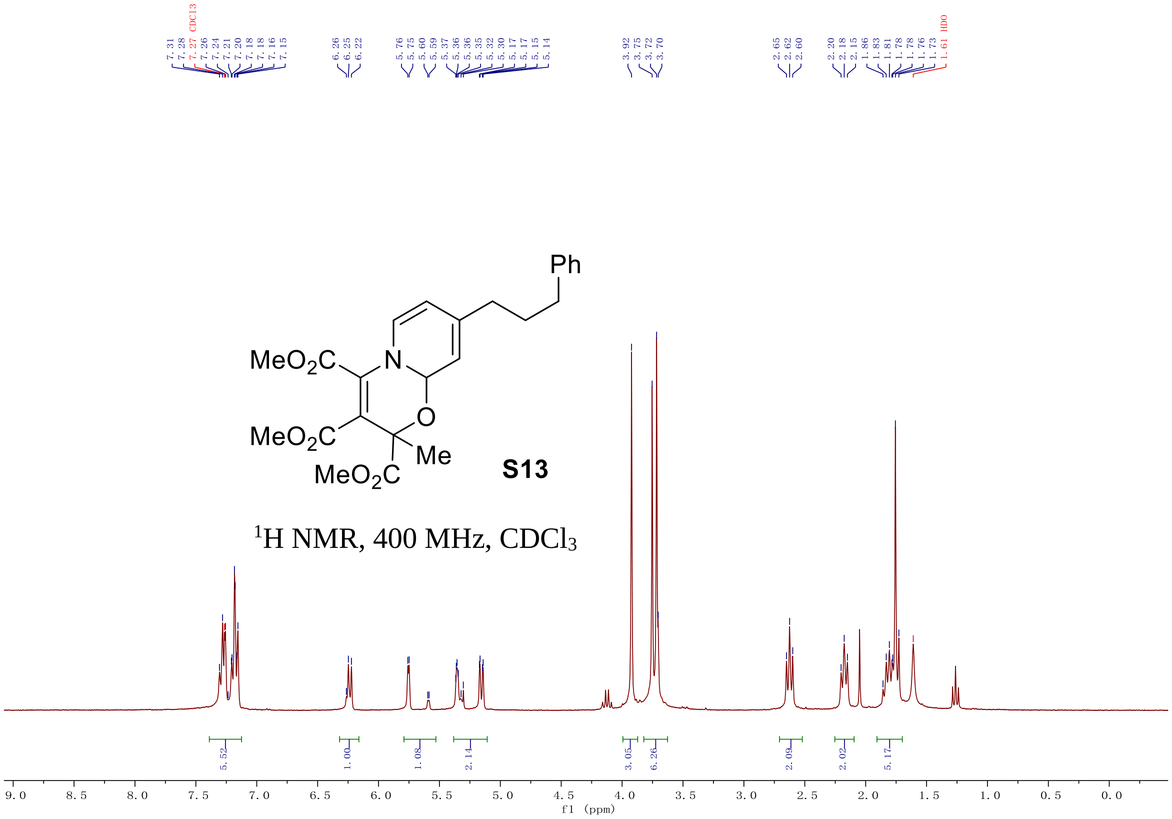

^{1}H NMR, 400 MHz, $CDCl_3$

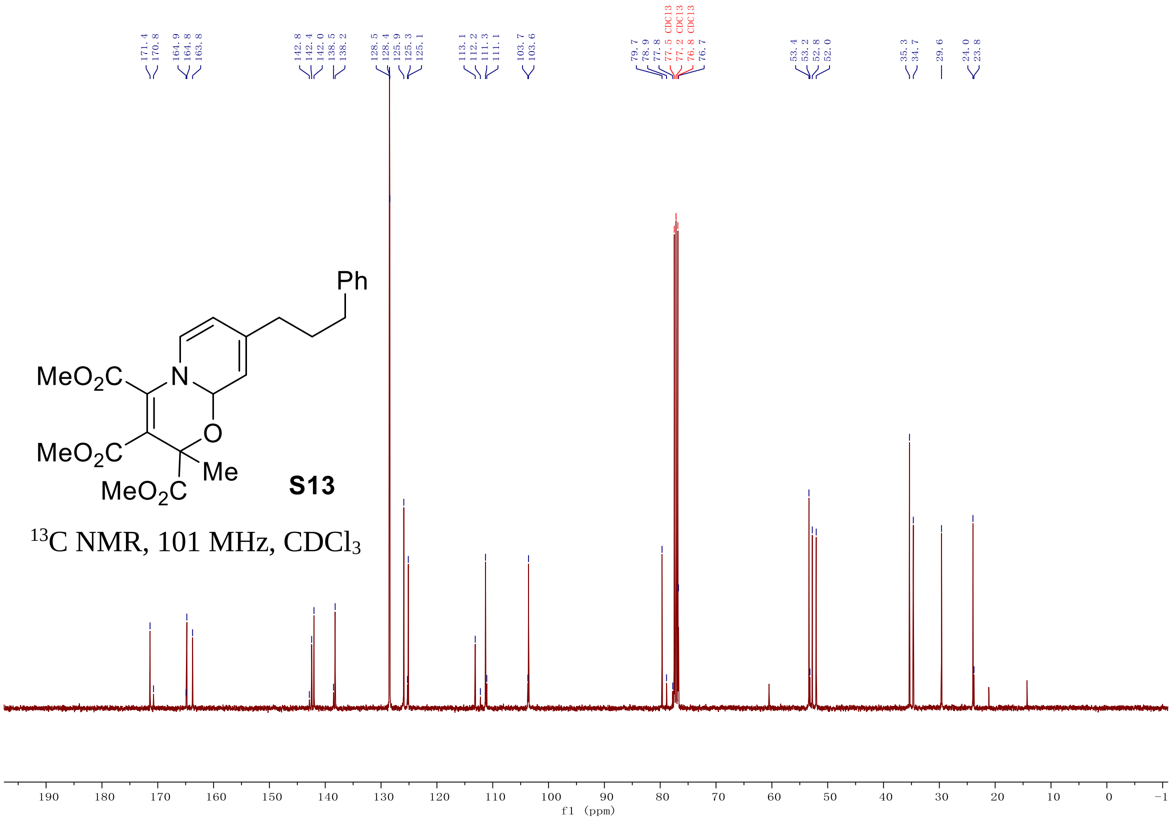

^{13}C NMR, 101 MHz, $CDCl_3$

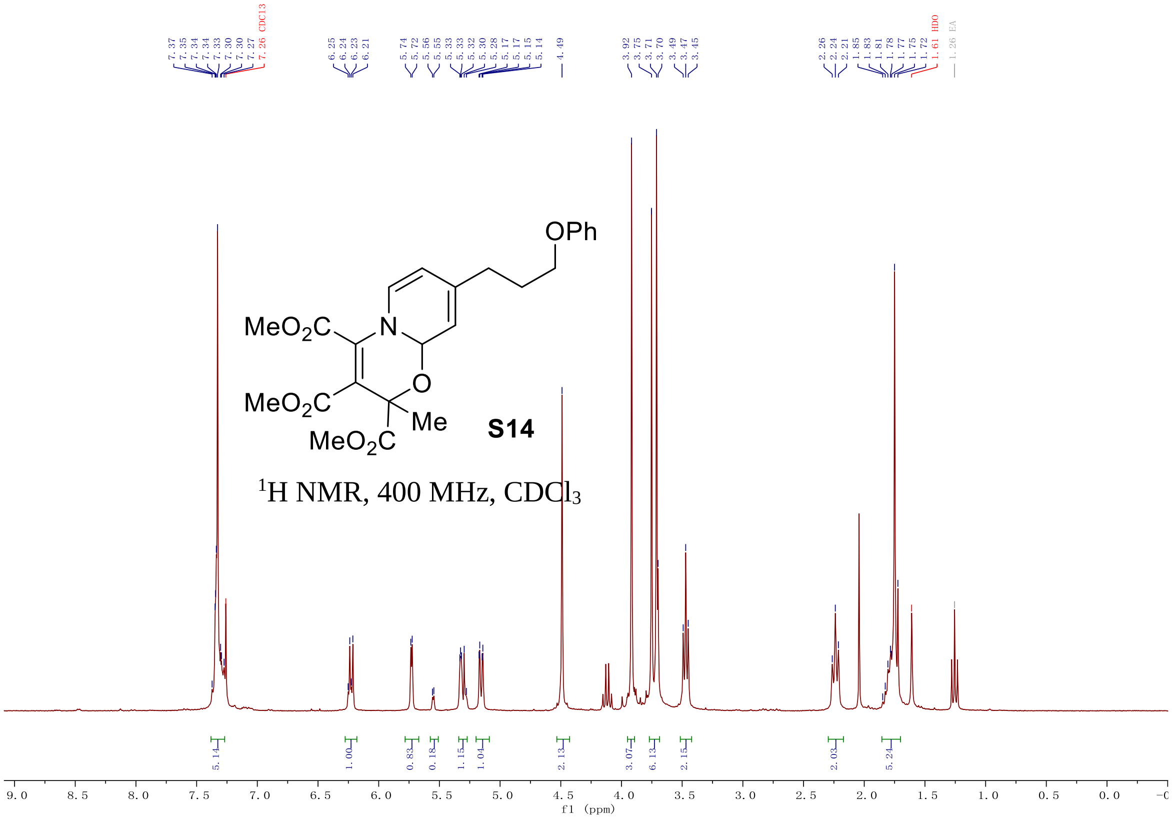
7.37 7.35 7.34 7.34 7.33 7.30 7.30 7.27 7.26 CDCl3
6.25 6.24 6.23 6.21
5.74 5.72 5.56 5.55 5.33 5.33 5.32 5.30 5.28 5.17 5.17 5.15 5.14
4.49
3.92 3.75 3.71 3.70 3.49 3.47 3.45
2.26 2.24 2.21 1.85 1.83 1.81 1.78 1.77 1.75 1.72 1.61 HDO 1.26 EA
OPh
MeO2C
N
MeO2C
O
Me
MeO2C
S14
1H NMR, 400 MHz, CDCl3
5.14 1.00 0.83 0.18 1.15 1.04 2.13 3.07 6.13 2.15 2.03 5.24
9.0 8.5 8.0 7.5 7.0 6.5 6.0 5.5 5.0 4.5 4.0 3.5 3.0 2.5 2.0 1.5 1.0 0.5 0.0
f1 (ppm)

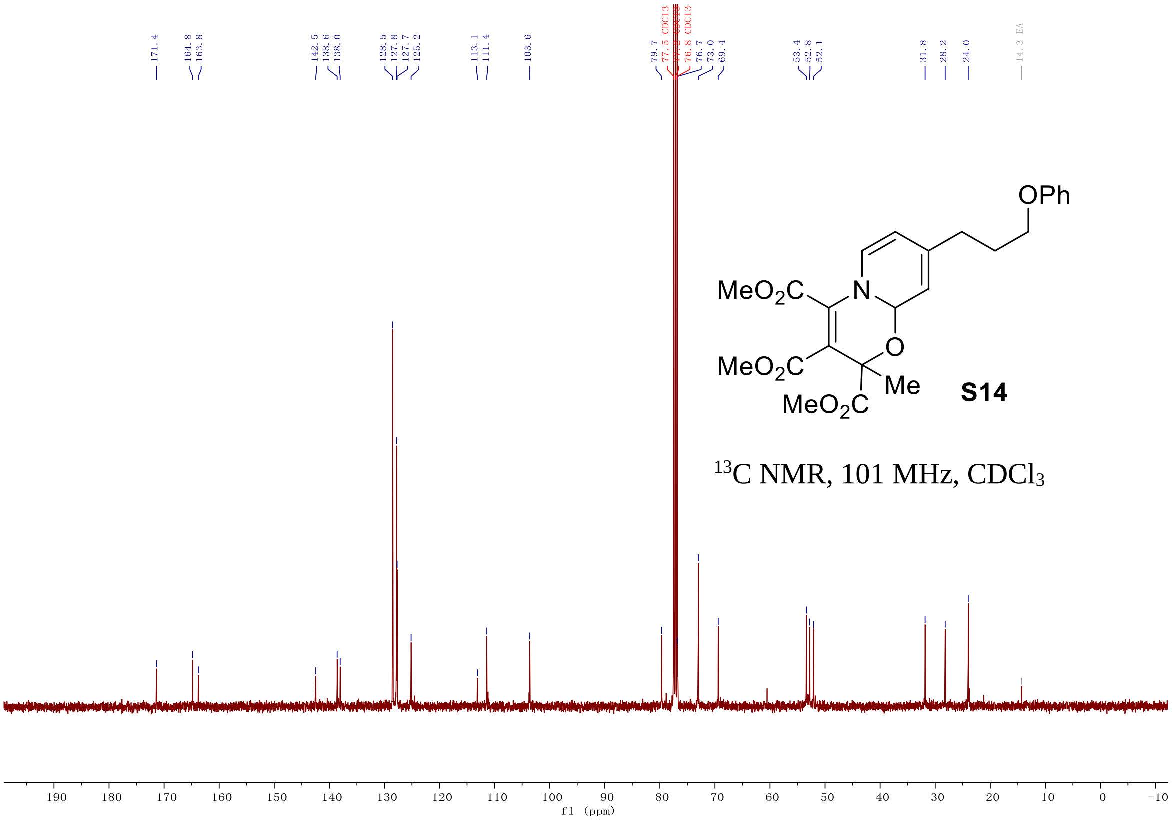
171.4 164.8 163.8 142.5 138.6 138.0 128.5 127.8 127.7 125.2 113.1 111.4 103.6
79.7 77.5 CDCl3 76.8 CDCl3 76.7 73.0 69.4 53.4 52.8 52.1 31.8 28.2 24.0 14.3 EA
OPh
MeO2C
N
MeO2C
O
Me
MeO2C
S14
13C NMR, 101 MHz, CDCl3
190 180 170 160 150 140 130 120 110 100 90 80 70 60 50 40 30 20 10 0 −10
f1 (ppm)

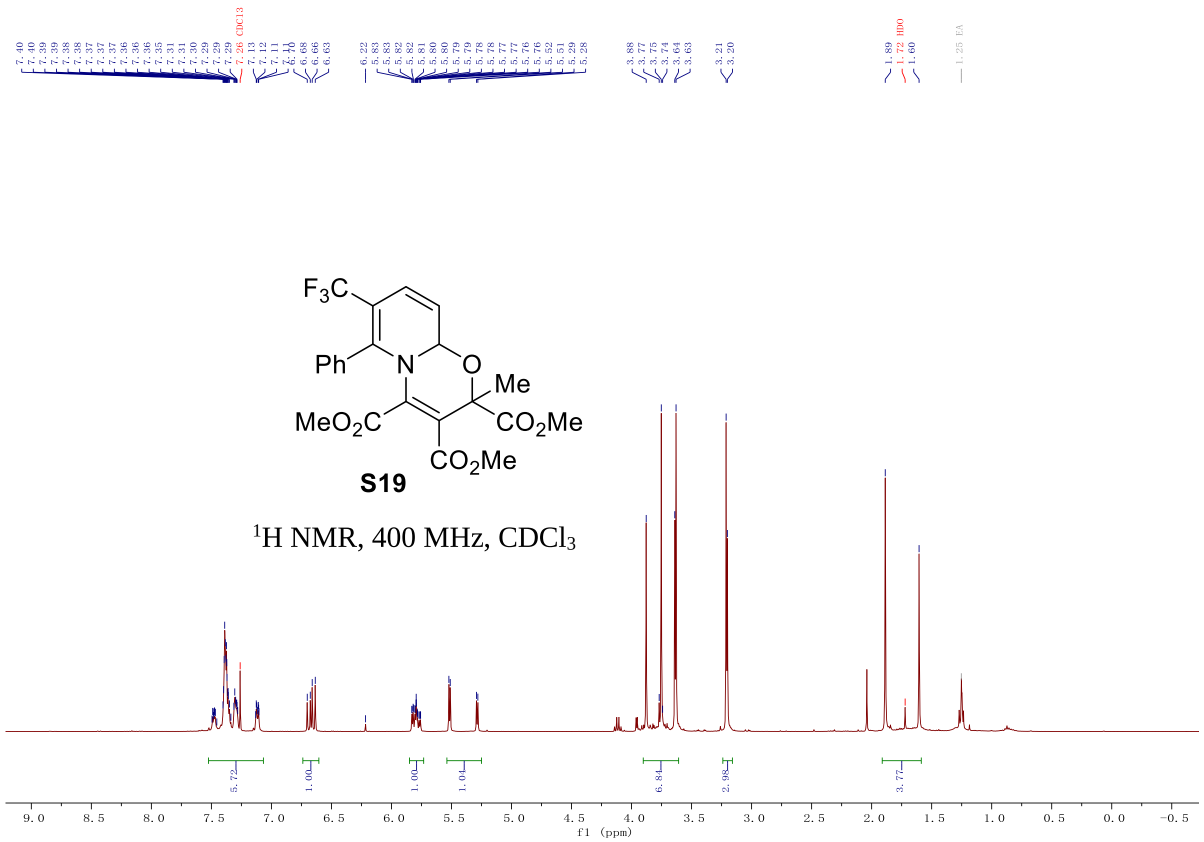

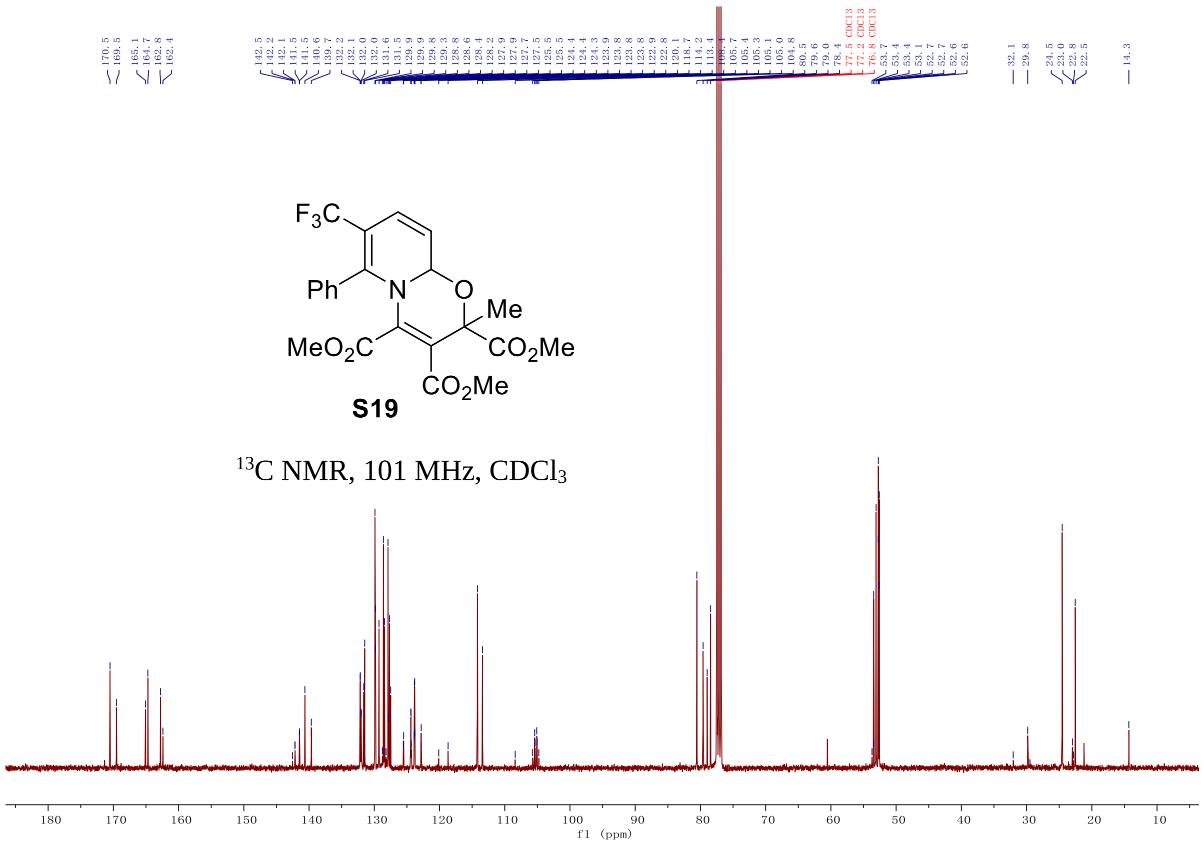

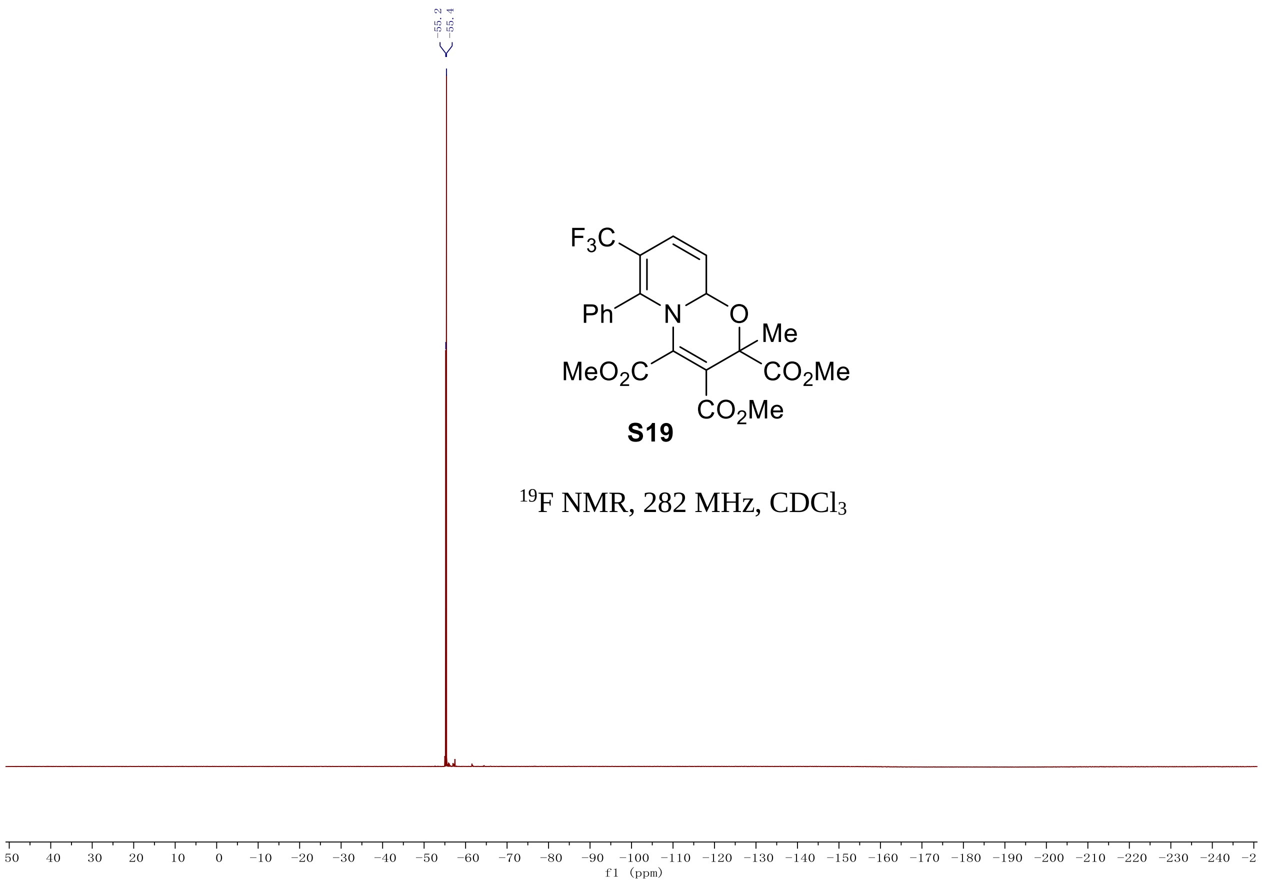
-55.2
-55.4
F3C
Ph
N
O
Me
MeO2C
CO2Me
CO2Me
S19
19F NMR, 282 MHz, CDCl3
50 40 30 20 10 0 -10 -20 -30 -40 -50 -60 -70 -80 -90 -100 -110 -120 -130 -140 -150 -160 -170 -180 -190 -200 -210 -220 -230 -240 -2
f1 (ppm)

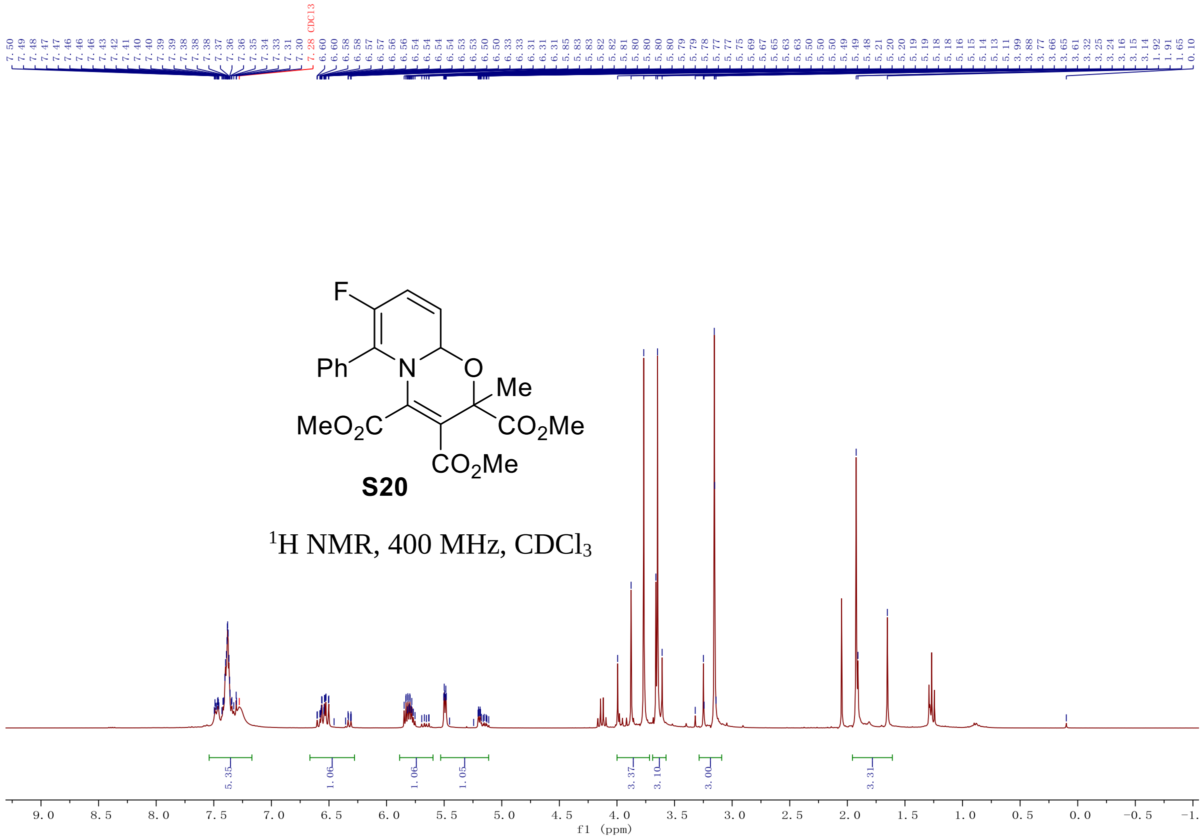
F
Ph
N
O
Me
MeO_2C
CO_2Me
CO_2Me
S20
^1H NMR, 400 MHz, $CDCl_3$
f1 (ppm)

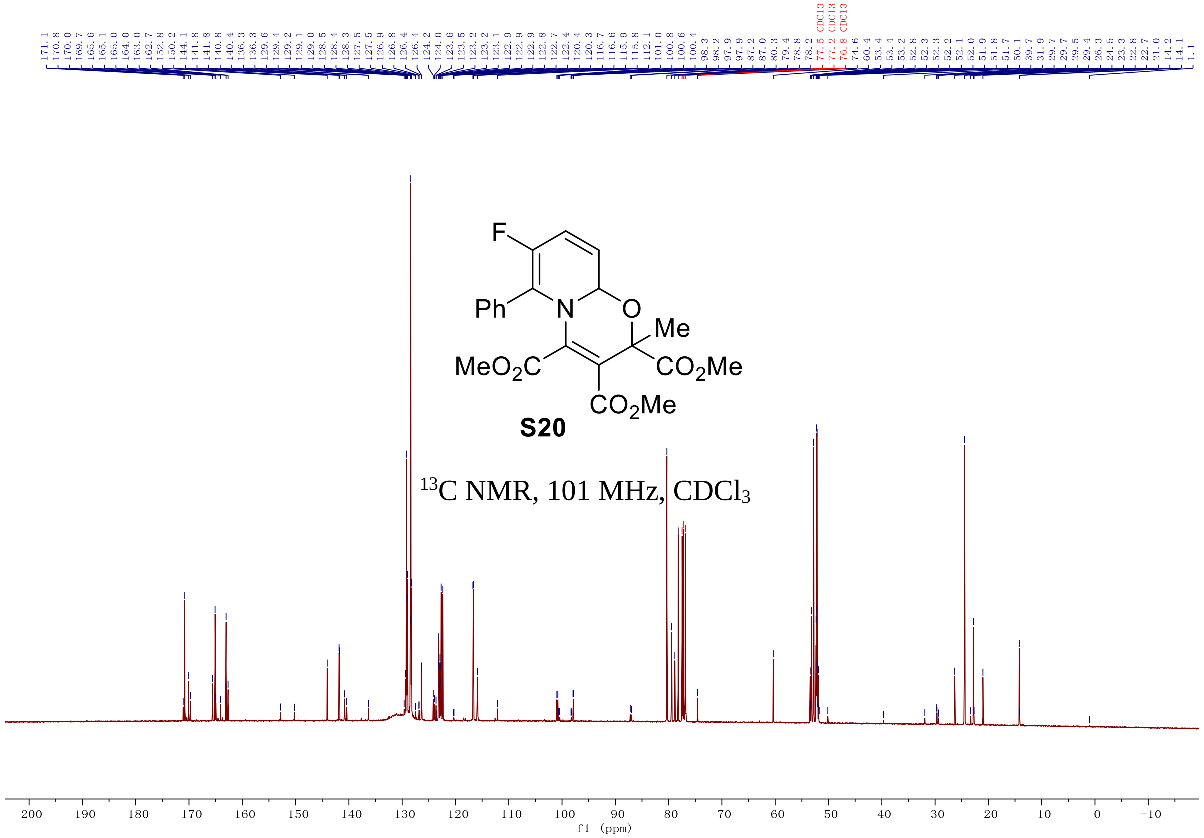
F
Ph
N
O
Me
MeO_2C
CO_2Me
CO_2Me
S20
^{13}C NMR, 101 MHz, $CDCl_3$
f1 (ppm)

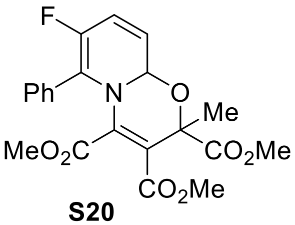

S20

^{19}F NMR, 282 MHz, $CDCl_3$

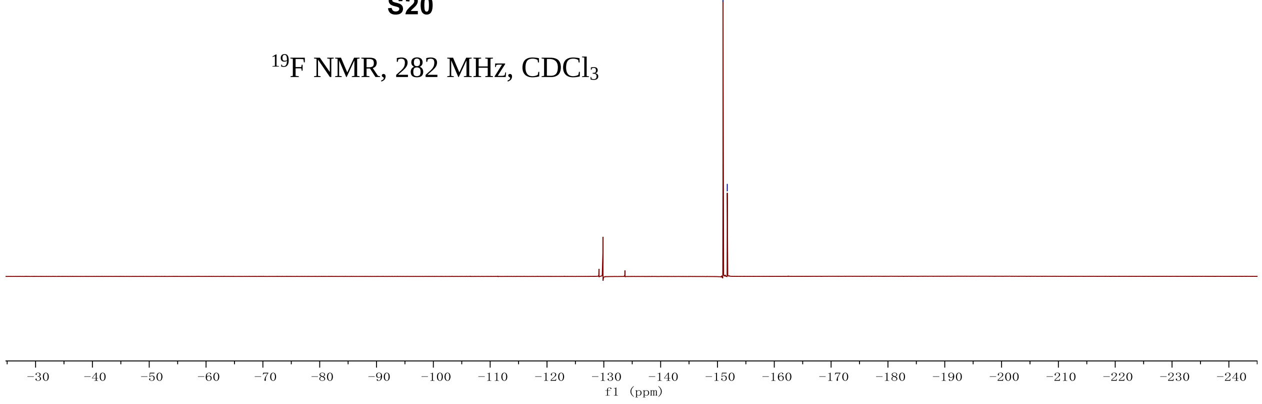

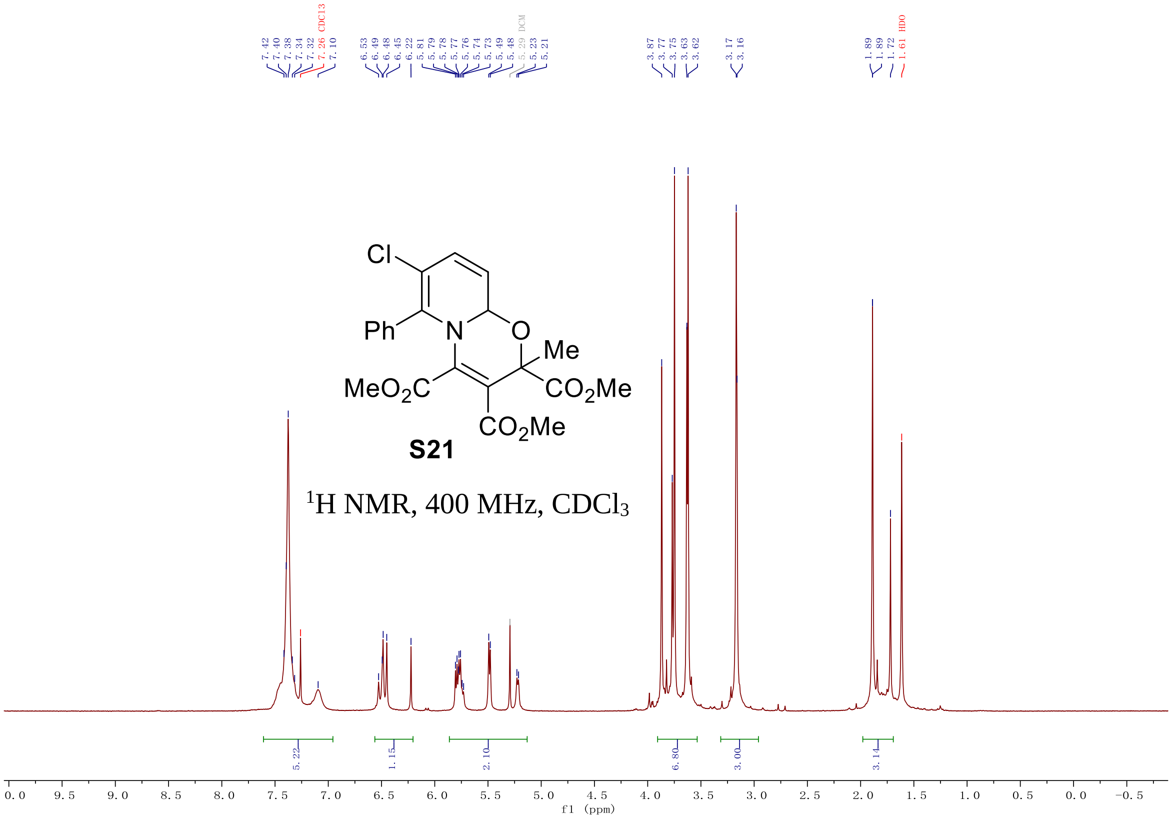
Cl
Ph
N
O
Me
MeO2C
CO2Me
CO2Me
S21
1H NMR, 400 MHz, CDCl3
7.42
7.40
7.38
7.34
7.32
7.26 CDCl3
7.10
6.53
6.49
6.48
6.45
6.22
5.81
5.79
5.78
5.77
5.76
5.74
5.73
5.49
5.48
5.29 DCM
5.23
5.21
3.87
3.77
3.75
3.63
3.62
3.17
3.16
1.89
1.89
1.72
1.61 HDO
5.22
1.15
2.10
6.80
3.00
3.14
f1 (ppm)

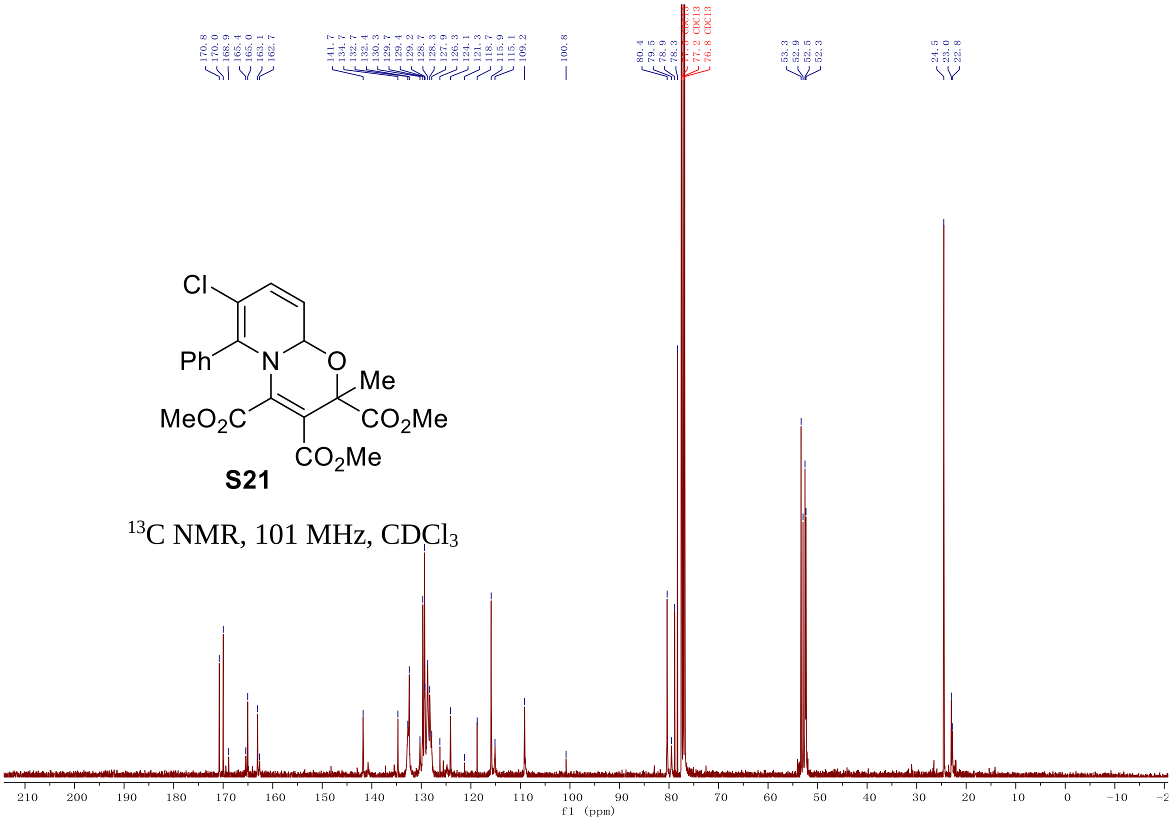
Cl
Ph
N
O
Me
MeO2C
CO2Me
CO2Me
S21
13C NMR, 101 MHz, CDCl3
170.8
170.0
168.9
165.4
165.0
163.1
162.7
141.7
134.7
132.7
132.4
130.3
129.7
129.4
129.2
128.7
128.3
127.9
126.3
124.1
121.3
118.7
115.9
115.1
109.2
100.8
80.4
79.5
78.9
78.3
77.2 CDCl3
76.8 CDCl3
53.3
52.9
52.5
52.3
24.5
23.0
22.8
f1 (ppm)

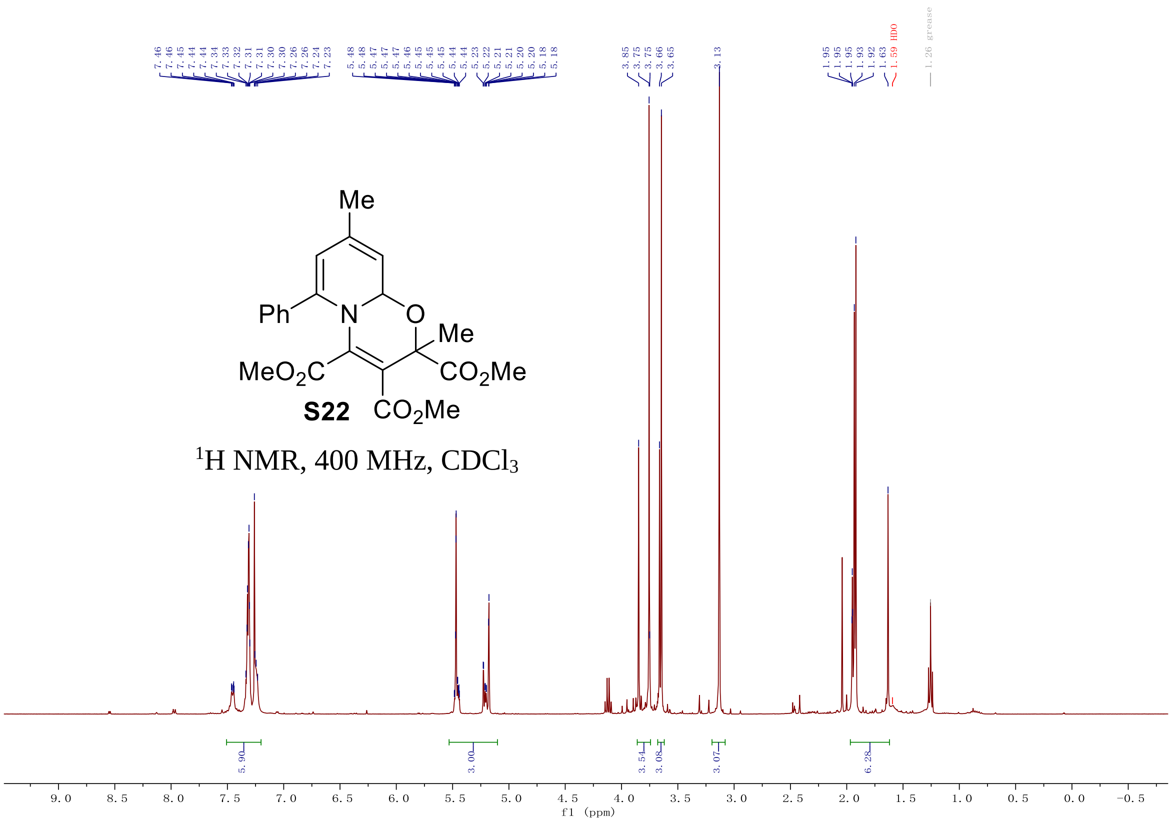

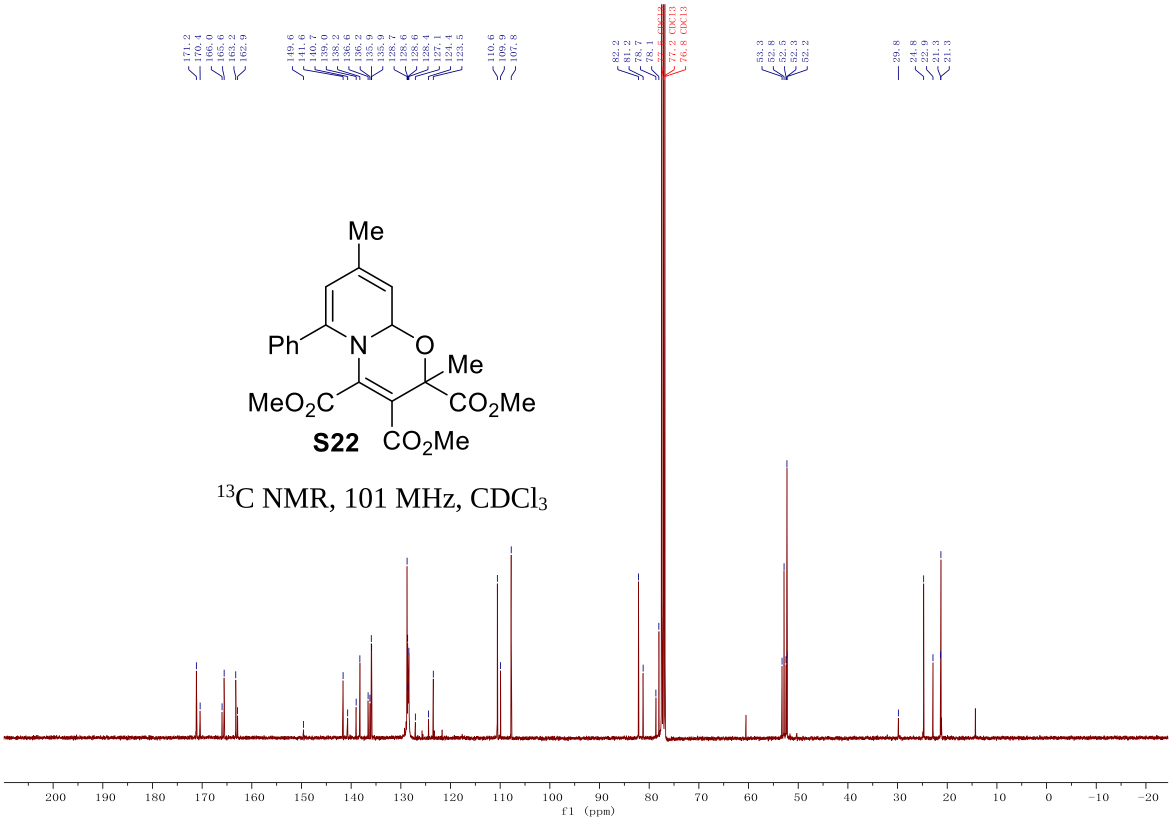

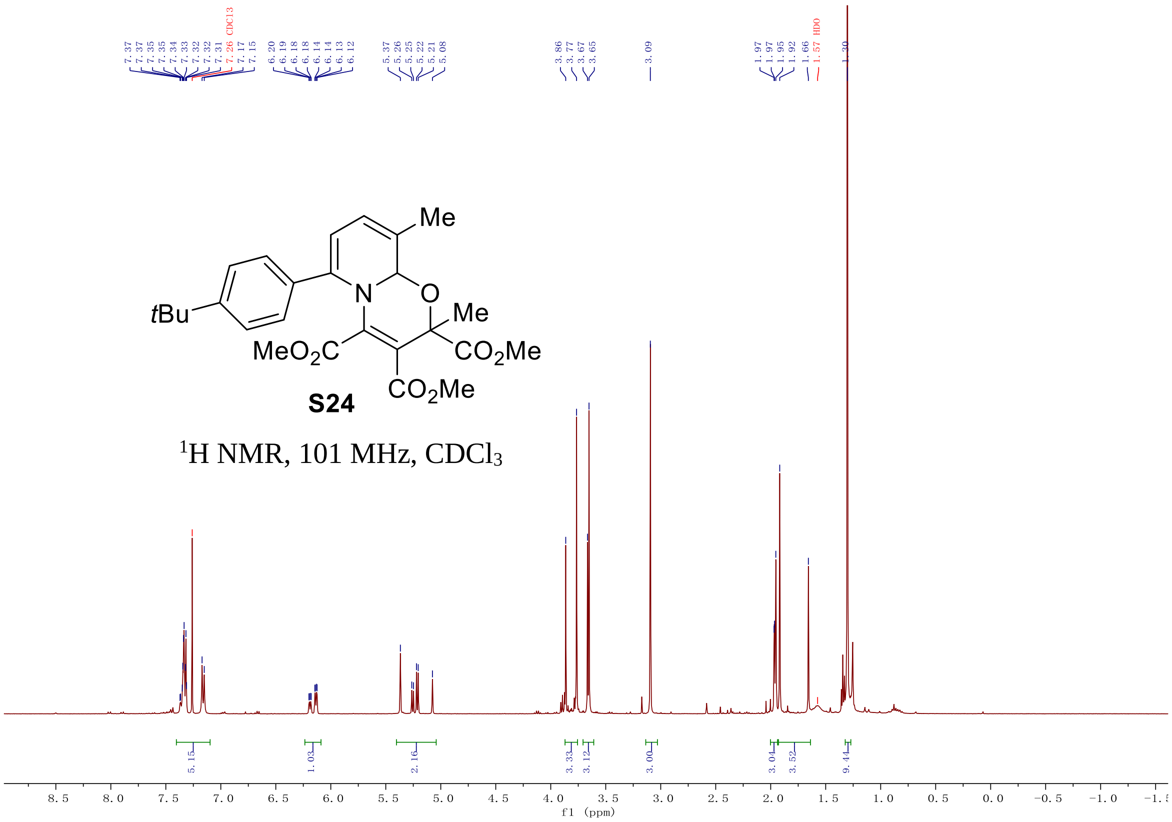
S24
^{1}H NMR, 101 MHz, CDCl$_3$

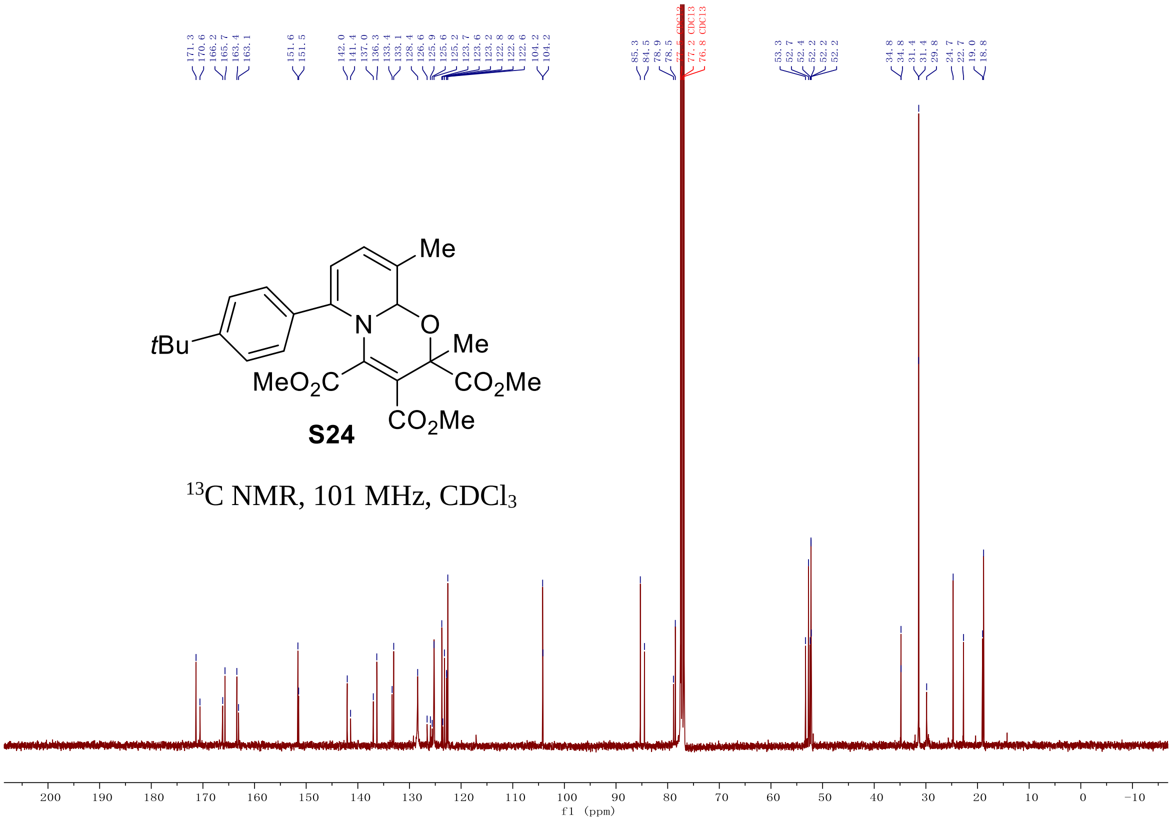
S24
^{13}C NMR, 101 MHz, CDCl$_3$

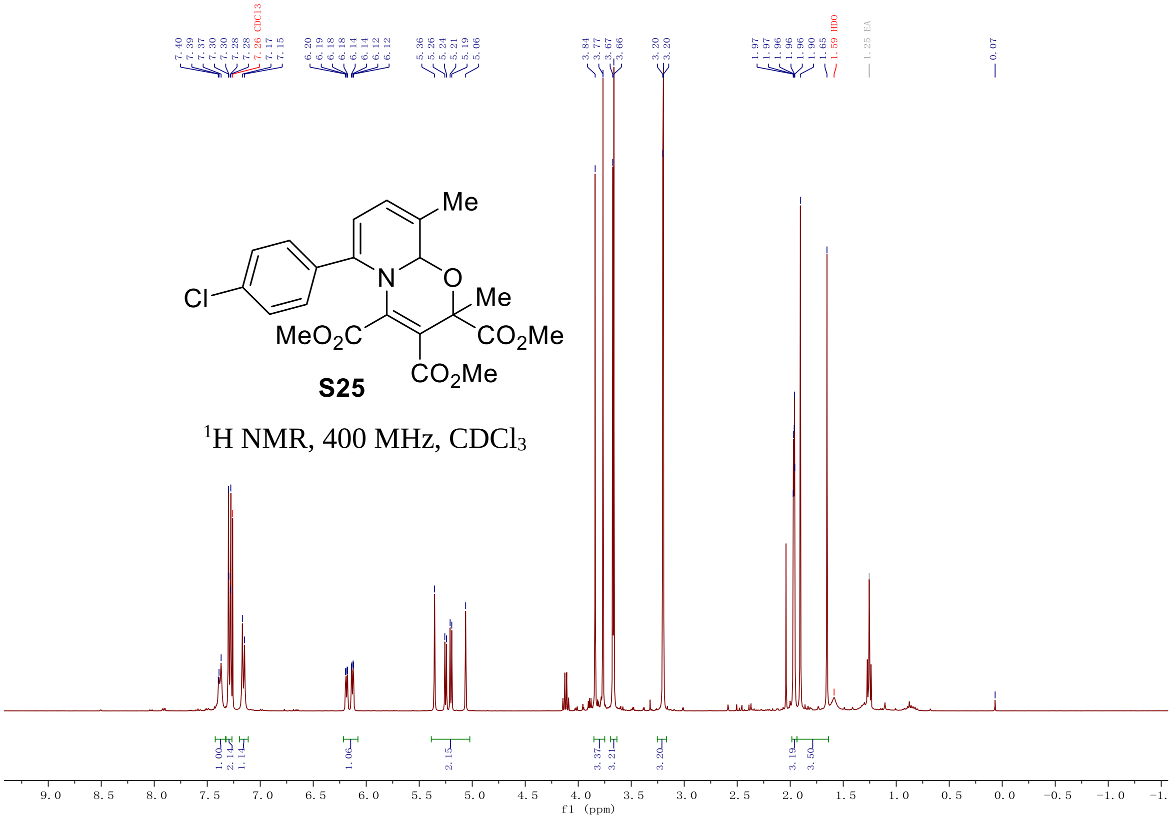

S25

^{1}H NMR, 400 MHz, $CDCl_3$

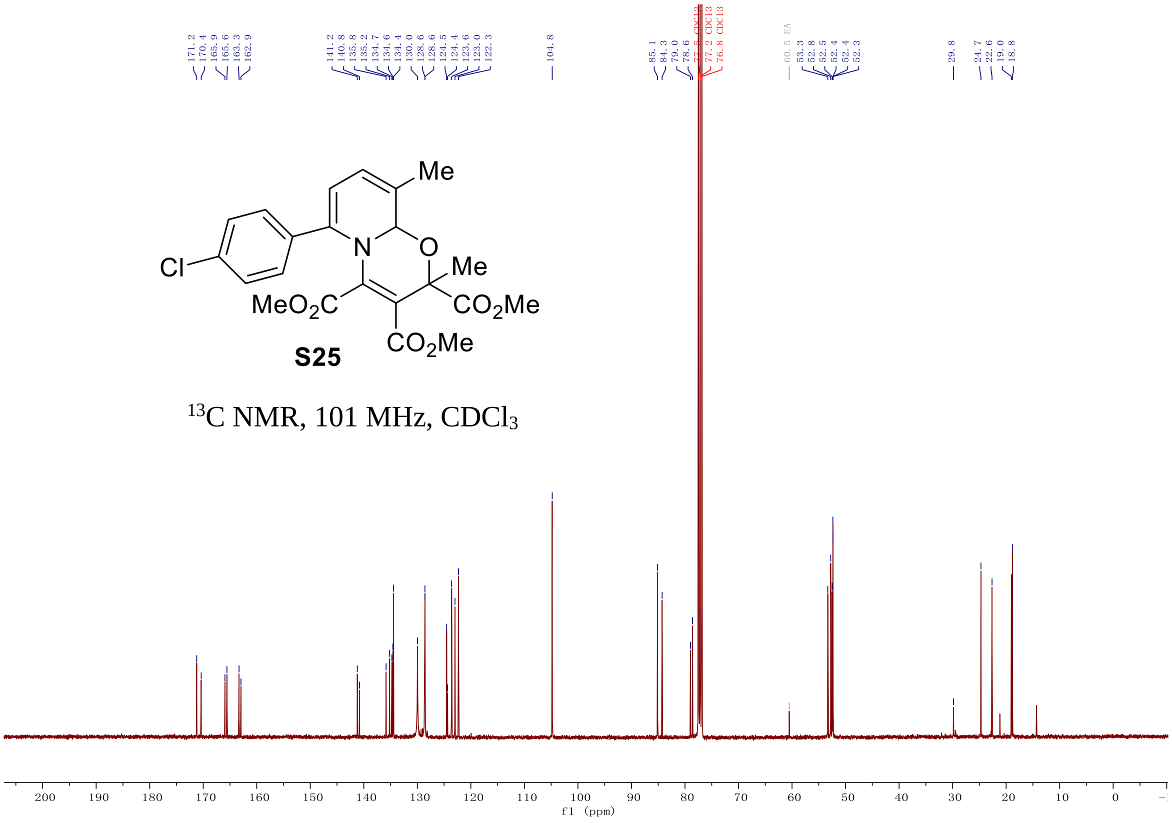

S25

^{13}C NMR, 101 MHz, $CDCl_3$

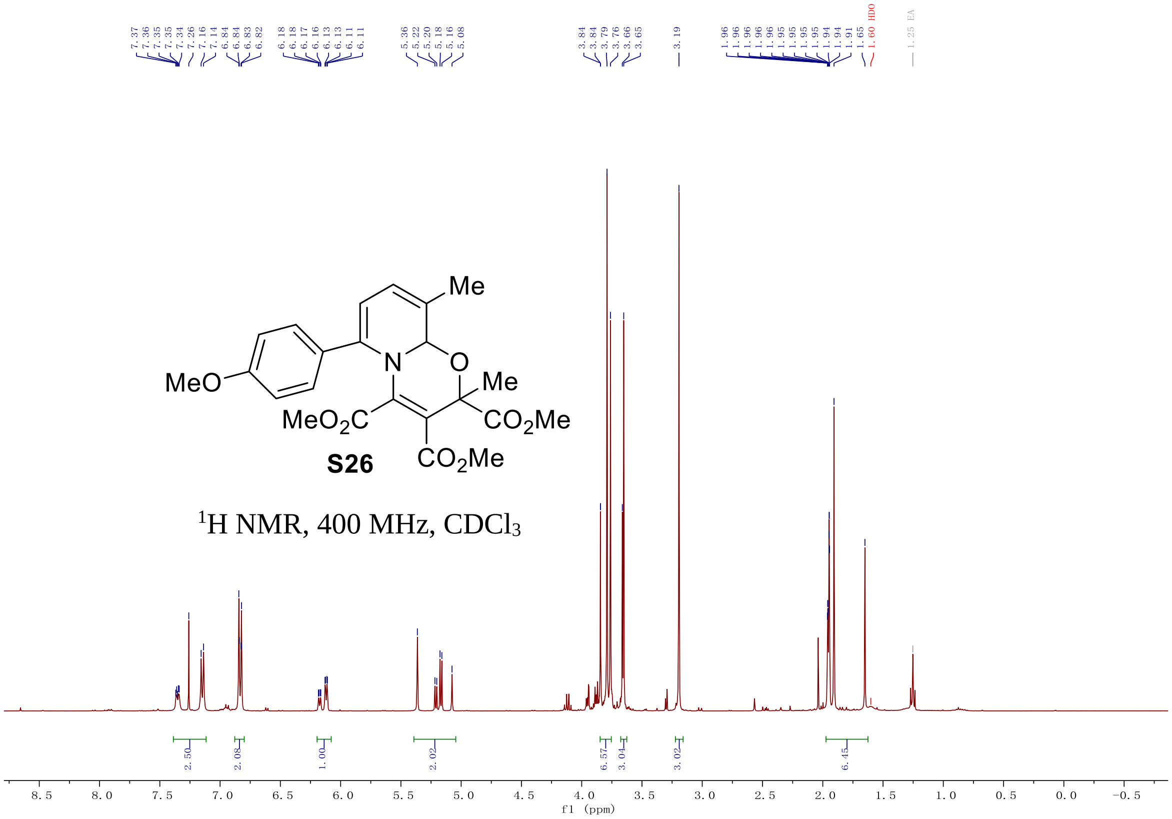
Me
MeO
N
O
Me
MeO_2C
CO_2Me
S26
CO_2Me
^{1}H NMR, 400 MHz, $CDCl_3$
f1 (ppm)

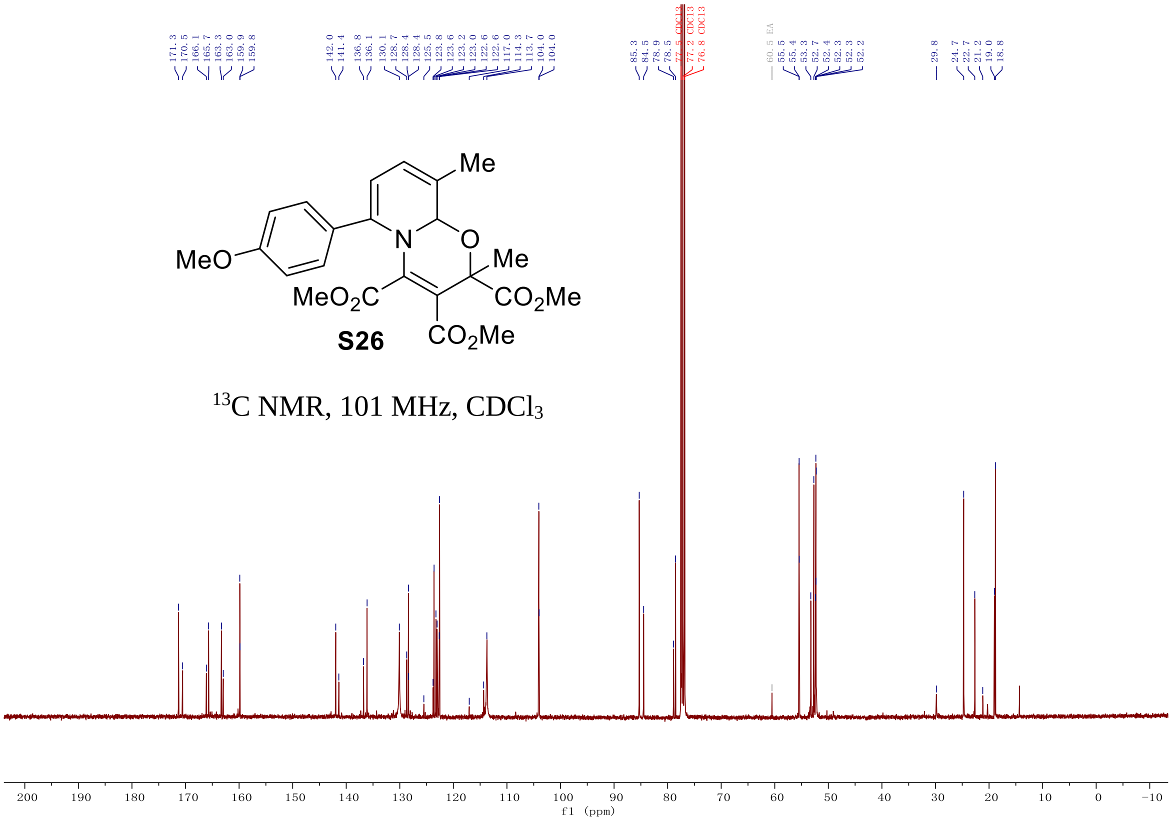
Me
MeO
N
O
Me
MeO_2C
CO_2Me
S26
CO_2Me
^{13}C NMR, 101 MHz, $CDCl_3$
f1 (ppm)

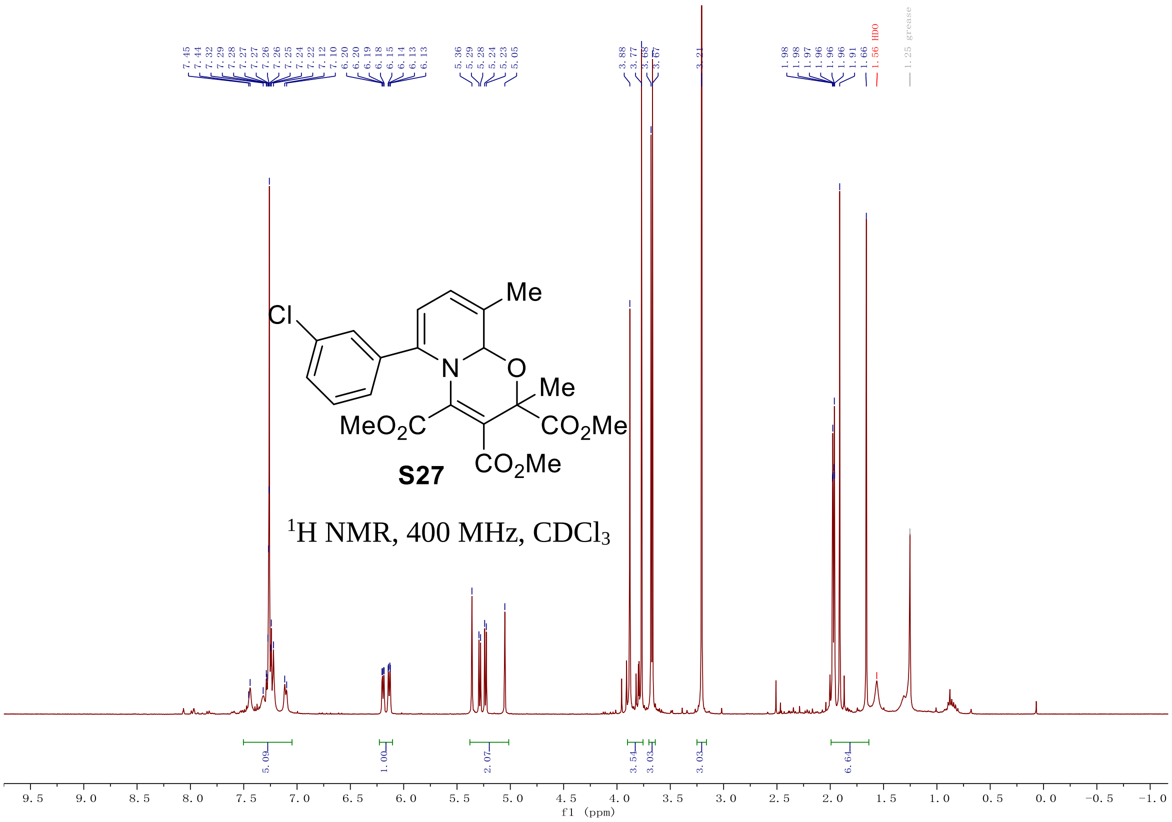

Me
Cl
N
O
Me
MeO2C
CO2Me
CO2Me
S27
1H NMR, 400 MHz, CDCl3
f1 (ppm)

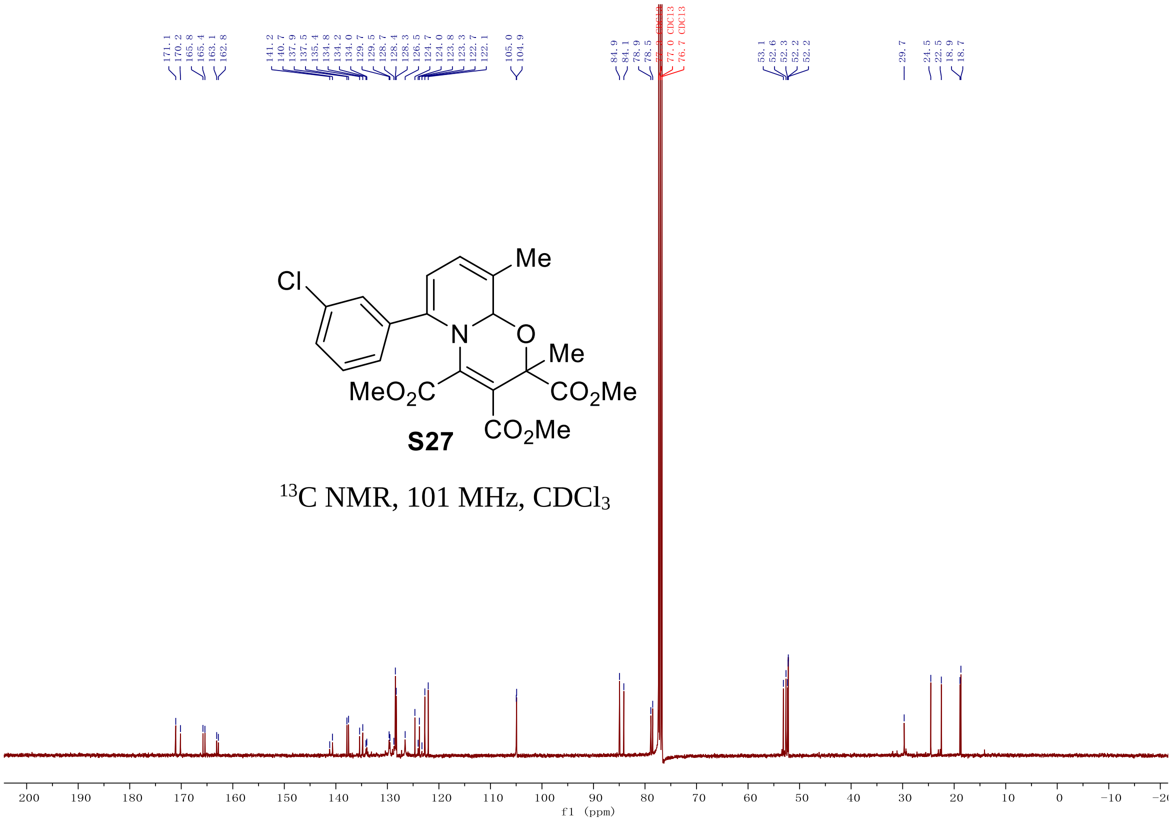

Me
Cl
N
O
Me
MeO2C
CO2Me
CO2Me
S27
13C NMR, 101 MHz, CDCl3
f1 (ppm)

References

1. Cao, H., Cheng, Q. & Studer, A. Radical and ionic *meta*-C–H functionalization of pyridines, quinolines, and isoquinolines. *Science* **378**, 779-785 (2022).
2. Cao, H., Bhattacharya, D., Cheng, Q. & Studer, A. C–H Functionalization of Pyridines via Oxazino Pyridine Intermediates: Switching to para -Selectivity under Acidic Conditions. *J. Am. Chem. Soc*. **145**, 15581-15588 (2023).
3. Xu, P., Wang, Z., Guo, S.-M. & Studer, A. Introduction of the difluoromethyl group at the *meta*- or *para*-position of pyridines through regioselectivity switch. *Nat. Commun.* **15**, 4121 (2024).
4. Haring, M., Balanna, K., Cheng, Q., Lammert, J. & Studer, A. Formal *meta*-C–H-Fluorination of Pyridines and Isoquinolines through Dearomatized Oxazinopyridine Intermediates. *J. Am. Chem. Soc.* **146**, 30758-30763 (2024).
5. Balanna, K. & Studer, A. *meta*-Nitration of Pyridines and Quinolines through Oxazino Azines. *J. Am. Chem. Soc.* **147**, 7485-7495 (2025).
6. Li, Y. & Studer, A. Transition-Metal-Free Trifluoromethylaminoxylation of Alkenes. *Angew. Chem. Int. Ed.* **51**, 8221-8224 (2012).
7. Waser, J. *et al*. Cyclic Hypervalent Iodine Reagents for Azidation: Safer Reagents and Photoredox-Catalyzed Ring Expansion. *J. Org. Chem*. **83**, 12334-12356 (2018).
8. Fukukawa, J. *et al*. Synthesis of *dl*-6-protolludene. *Chem. Pharm. Bull*. **33**, 440-443 (1985).
9. Shafi, S. *et al*. Synthesis of novel 2-mercapto benzothiazole and 1,2,3-triazole based bis-heterocycles: Their anti-inflammatory and anti-nociceptive activities. *Eur. J. of Med. Chem.***49**, 324-333 (2012).
